# Primary metabolomics and transcriptomic techniques were used to explore the regulatory mechanisms that may influence the flavor characteristics of fresh *Corylus heterophylla* × *Corylus avellana*

**DOI:** 10.3389/fpls.2024.1475242

**Published:** 2025-01-30

**Authors:** Minmin Lu, Tiantian Xie, Yaru Wang, Jieyan Yang, Yan Bai, Shuang Gao, Xiaofan Wu, Xiuqing Yang

**Affiliations:** College of Forestry, Shanxi Agriculture University, Taigu, China

**Keywords:** fresh *C. heterophylla* × *C. avellana*, flavor, sugars, organic acids, amino acids and their derivatives, lipids

## Abstract

To explore the flavor related regulatory mechanisms of fresh *Corylus heterophylla* × *Corylus avellana*, a joint analysis of metabolome and transcriptome were utilized to compare the two typical *C. heterophylla* × *C. avellana* varieties with different flavors (‘yuzhui’ and ‘pingou21’) in this paper. The results showed that the genes including *E2.4.1.67-1*, *E2.4.1.67-2*, *SUS-1*, *SUS-2*, *SUS-4*, *SUS-5*, *SUS-7*, *SUS-8*, *SUS-9*, *UGP2-2* were identified as responsible for regulating the levels of stachyose, manninotriose and raffinose in hazelnuts. CS and OGDH were deemed as the genes involved in the citric acid cycle, which was a central metabolic pathway that generated energy through the oxidation of carbohydrates, fats and proteins in hazelnuts. The genes *trpD*, *ALDO*, *PK-1*, *PK-2*, *ilvH*, *argE-1*, *argE-4*, *argE-5*, *argD*, *PDAH*, *GLTI* were regarded as involved in the biosynthesis of various amino acids like tryptophan, valine, alanine, and arginine. These amino acids determined the taste of *C. heterophylla* × *C. avellana* and were important precursors of other flavor-related compounds. The genes *LOX2S-2*, *LOX2S-3*, *LOX2S-4* and *LCAT3* were viewed as involved in the regulation of lipid biosynthesis, specifically involving 13(S)-HPODE, 9,10,13-trihome and 13(S)-HOTrE in *C. heterophylla* × *C. avellana*. These findings highlight the significance of genes and metabolites and internal regulatory mechanisms in shaping the flavor of fresh *C. heterophylla* × *C. avellana* cultivated in temperate continents. This study provides the theoretical basis for breeding excellent food functional hazelnut varieties.

## 1 Introduction

Hazelnut is one of the four major nuts in the world (Ma et al., 2021). In 2022, for the first time, Hazelnut was listed as an important woody forest food species in the National Reserve Forest catalog and the 14th Five-Year Key Research and Development Plan of China (Ma et al., 2023). *Corylus heterophylla* × *Corylus avellana* is the only cultivated Corylus species in China, which is a cross between Chinese wild *Corylus platyphylla* and European *Corylus avellana* (Liang et al., 2000). C*. heterophylla* × *C. avellana* has been developing as a new type of economic forest tree species in China (Cui, 2012). Previous studies have demonstrated that hazelnuts are rich in nutrients, primarily including proteins, fatty acids (monounsaturated fatty acids, MUFAs), vitamins, amino acids, and more. On average, hazelnut kernels are composed of 60% fat, 15% crude protein, 4% ash, and 4% water. Additionally, it has been reported that hazelnuts are the second-largest source of MUFAs (82%–83%) after walnuts (Liu, 2022; Markuszewski et al., 2022; Zhao et al., 2023). In particular, as a promising freshly edible but often neglected tree species, the research of metabolites and related quality traits in fresh fruit of *C. heterophylla* × *C. avellana* is still blank, despite it play a vital role in the industrial development of hazelnuts (Callahan et al., 1992).

Great progress has been made in study on fruit nutrition metabolism of economic forest tree species and their regulatory mechanisms since the past three decades due to the advances in gene sequencing and bioinformatics technics (Dominguez-Puigjaner et al., 1997; Hadfield et al., 1998; Font i Forcada et al., 2015; Liu et al., 2020; Hu et al., 2023; Zhang C. et al., 2022). Liu et al (2020) studied the polyunsaturated fatty acids in walnuts and showed that *JrFAD3-1* play an important role in the production of polyunsaturated fatty acids. Zhang Z. et al. (2022) detected the flavonoid in the kernels of two pecan cultivars varieties and discovered that the *MYB4* transcription factor (CIL1093S0100) may act as an inhibitor of flavonoid biosynthesis during fruit development. Chen et al. (2023) studied the citric acid in citrus and demonstrated a negative correlation between *CS* expression and citric acid, whereas *ACL* expression has shown a positive correlation with citric acid. Zhang Z. et al. (2022) studied the sugar synthesis of ‘Hongshuijing’ Pitaya fruit and showed that *HpVAI1* played a key role in sugar metabolism during the ripening of the fruit. Many studies have also been conducted on fruit nutrients in some other economic plants. Yin et al. (2010) studied the organic acids and γ-aminobutyric acid of developing tomato and showed that at the beginning of ripening, conversion of GABA to malic acid by succinic semialdehyde is necessary for its entry into the shunt pathway via pyruvate. It is subsequently cycled back into the tricarboxylic acid cycle, where it is stored as citric acid and serves as an energy source for respiration during fruit ripening. Fei et al. (2022) researched amino acids in green and red pepper fruits and identified 10 genes (including *PK*, *PFK*, *ENO*, *ASL*, *DAPD*, *THR*, *CYSK*, *METH*, *CM*, and *TRYA*) that played a significant role in amino acid synthesis.

The study of fruit metabolites not only revealed the nutrients in fruits of economic plants, but also helps us to understand the regulation of fruit flavor of these plants. The composition and content changes of their fruit nutrients are also the key factors that determine the formation of fruit flavor. Studies of loquats have shown that the relative proportions and concentrations of sugars, organic acids, and amino acids present in the fruit determined the taste of loquat, while its aroma is derived from volatile compounds like phenols and alcohols (Zou et al., 2020). Tieman et al. (2006) studied the flavor during tomato ripening and showed that the breakdown of certain organic acids and amino acids leads to the formation of volatile compounds, which contribute to the characteristic aroma of ripe tomatoes. Guo et al.’s (2022) study showed that the changes in flavor of litchi depend on the composition and content of sugars and acids, which were affected by the changes in carbohydrate metabolic pathways.

These results indicated that primary metabolites such as sugar, organic acids, amino acids and related compounds can directly or indirectly affect fruit flavor. And these flavor-related primary metabolites and their regulatory processes have been extensively studied in fruits, but there is limited understanding of flavor-related metabolite accumulation and gene regulation in some economically significant nuts, especially *C. heterophylla* × *C. avellana*. Metabolomics has been shown to be effective in identifying primary metabolites such as sugars, organic acids, amino acids, and other related compounds (Sangpong et al., 2021). And a combined analysis using primary metabolomics and transcriptomic techniques is essential for studying the molecular regulatory mechanisms that may influence the flavor characteristics of fresh *C. heterophylla* × *C. avellana*.

Therefore, in this study, primary metabolomics and transcriptomics were applied to analyze the main factors and potential key regulatory processes that may affect the flavor of 3 varieties fresh *C. heterophylla* × *C. avellana*. This study aims to provide a basis for further understanding and discovery of key genes affecting fresh nut flavor and is of great significance for breeding new varieties with ideal sensory characteristics of *C. heterophylla* × *C. avellana*.

## 2 Materials and methods

### 2.1 Plants materials and treatment

In order to understand the regulatory mechanism of flavor formation in the fresh food type hazelnut, metabolism and transcriptome analysis were performed on the fresh fruits of 3 varieties of *C. heterophylla* × *C. avellana* namely ‘Dawei(DW)’, ‘yuzhui (YZ)’ and ‘pingou21 (B21)’, which were cultivated in Taigu District, Shanxi Province, China(112°40’ 27.32” E, 37°24’ 42.73” N). The 3 varieties of *C. heterophylla* × *C. avellana* were all six years old but with different sensory characteristics. The oiliness of DW and YZ is heavier than that of B21, but the sweetness of B21 is heavier than that of DW and YZ. For each variety, we randomly collected 60 ripe fresh nuts from 5 consistently growing trees on July 25th, which were rapidly frozen in liquid nitrogen after removing the bracts and subsequent storage at a temperature of -80°C for physiological assays, metabolite analysis and RNA sequencing. For every experiment, we utilized three biological replicates.

### 2.2 Quality indicators evaluation

The single kernel weight was measured using a balance with an accuracy of one in ten thousand. The mean value of kernel three-diameter (kernel of a nut in three dimensions: length, width, and thickness) was determined using a vernier caliper. Soluble sugar and starch contents were assessed using the anthrone sulfuric acid method. The total soluble solids content of the nuts was measured with a handheld refractometer (ATAGO PAL-1, Japan). Titratable acid content was measured using NaOH titration, while crude fat content was measured via petroleum ether extraction. Data sorting was performed using Excel 2016, and Origin 8.5 was used for data visualization. SPSS19.0 was employed for statistical analysis. All data is presented as means ± standard errors. The threshold for statistical significance was set at a *P*-value of less than 0.05.

### 2.3 Metabolite extraction and profiling

The kernels were taken out from quick frozen *C. heterophylla* × *C. avellana* nuts and grounded into a powder form using a grinder of MM 400, Retsch at a frequency of 30 Hz for 1.5 minutes after freeze-dried in a vacuum. A total of 50 mg of sample powder was dissolved in 1200 μL of an internal standard extract of 70% methanol in water that had been pre-cooled to -20°C. After centrifugation at 12000 rpm for 3 minutes, the extracts were absorbed and filtered before undergoing UPLC-MS/MS analysis. The data acquisition instrument system primarily included Ultra Performance Liquid Chromatography (UPLC; ExionLC AD, https://sciex.com.cn/) and Tandem Mass Spectrometry (MS/MS; Applied Biosystems 6500 QTRAP, https://sciex.com.cn/) (Wang et al., 2017). The analytical conditions were as follows: Agilent SB-C18 column (1.8 µm, 2.1 mm × 100 mm), with the mobile phase comprised of solvent A (ultrapurified water with 0.1% formic acid) and solvent B (acetonitrile containing 0.1% formic acid). Sample measurements were conducted using a gradient procedure with 95%A and B starting conditions. Within a 9-minute timeframe, a linear gradient of 5% solvent A and 95% solvent B was implemented, and the composition of 5%A and 95%B was maintained for 1 minute. Subsequently, the composition was adjusted to 95% solvent A and 5% solvent B for 1.10 minutes, and this composition was maintained for 2.9 minutes. The column temperature was set at 40°C, and the injection volume was 2 μL. The effluent was connected to an ESI-triple quadrupole-linear ion TRAP (Q TRAP)-MS (AB SCIEX, USA). Triple quadrupole-linear ion trap mass spectrometer (QTRAP; Linear ion TRAP (LIT) and triple quadrupole (QQQ) scans were performed using an API 4500 Q TRAP LC/MS/MS system). Analyst 1.6.3 software and multiple reaction monitoring (MRM) were used for metabolite data analysis and quantification, respectively. Finally, the identified metabolites underwent partial least squares discriminant analysis (PLS-DA). The screening criteria for significant differences were log2|FC| ≥ 1 or log2|FC| ≤-1 and VIP ≥ 1.

### 2.4 RNA-seq and annotation

For this study, a total of nine transcriptome sequencing libraries were constructed for three sample varieties of *C. heterophylla* × *C. avellana*, with each sample variety represented by three biological replicates. Total RNA was obtained using the TRIzol method (Xia et al., 2021). The concentration, purity, and integrity of the RNA were evaluated using different instruments: the Qubit 2.0 fluorometer, NanoPhotometer spectrophotometer, and Agilent Bioanalyzer 2100 system (Agilent Technologies, Palo Alto, CA, USA), respectively.

### 2.5 Transcriptome data analysis

The clean reads were assembled using Trinity to obtain reference sequences for subsequent analysis. The Unigene sequence was subjected to comparison with the KEGG, NR, Swiss-Prot, GO, COG/KOG, and Trembl databases using the DIAMOND (Buchfink et al., 2015) BLASTX software. The Unigene was subjected to amino acid sequence prediction, and annotation information for the Unigene was obtained by comparing it with the Pfam database using HMMER software. Clean reads were aligned to reference using STAR with default parameters. Fragments per kilobase of exon model per million mapped reads (FPKM) values were generated using RSEM. PCA analysis was performed by R package ‘factoextra’. Differential expression analyses were performed using the R package ‘DESeq2’. Genetic screening conditions were set at |log2Fold Change| ≥ 1 and *P* < 0.05 to determine differences. Subsequently, the genes were annotated in the KEGG database, and the number of differentially expressed genes in each KEGG pathway was tallied to identify the significantly enriched pathways among them. Finally, enrichment analysis was conducted to examine the distribution of differentially expressed genes in gene ontology and elucidate the functional expression of these genes in the experiment.

## 3 Results and discussion

### 3.1 Morphological and flavor related indicators in 3 varieties of fresh *C. heterophylla* × *C. avellana* nuts

**Figure 1:**
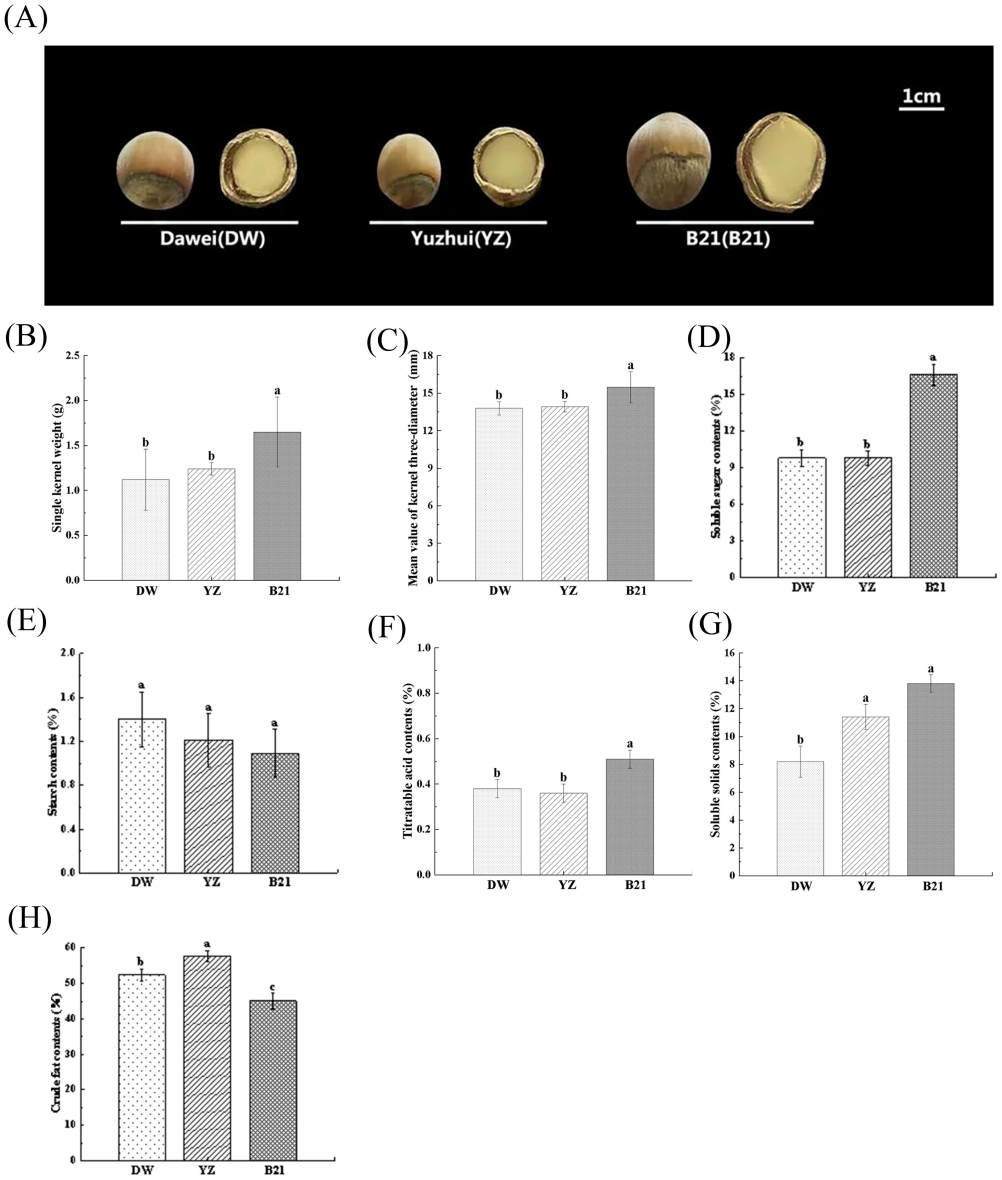
The appearance images **(A)**, single kernel weights **(B)**, mean value of kernel three- diameters **(C)**, soluble sugar contents **(D)**, starch contents **(E)**, soluble solids contents **(F)**, titratable acid contents **(G)**, crude fat contents **(H)** of three varieties of fresh *C*. *heterophylla × C. avellana* nuts (DW: ‘Dawei’, YZ: ‘Yuzhui’, and B21: ‘Pingou21’). Data show means ± SD. Different letters indicate significant difference (Tukey’s HSD test at *P*-value < 0.05).

Except for starch content, quality indicators, including nut shape and size, single kernel weight, mean value of kernel three-diameter, soluble sugar content, soluble solids content, titratable acid content and crude fat content showed significant differences among different varieties of *C. heterophylla* × *C. avellana* (**Figures 1A–H**). B21 was large in size, and it exhibited a different conical shape from the other two nearly circular varieties (**Figure 1A**). Actual measurement results showed that B21 had significantly higher single kernel weight and mean value of kernel three-diameter compared to the other varieties (**Figures 1B, C**), with values of 1.65g and 15.47mm, respectively. Moreover, the soluble sugar content and titratable acid content in B21 was significantly higher than that in DW and YZ (**Figures 1D, G**). Additionally, the total soluble solids (TSS) content in B21 and YZ was 13.80% and 11.41%, respectively, which was significantly higher than that in DW (**Figure 1F**). However, the crude fat content in B21, which was measured at 45.05%, indicated a significantly lower value than that of YZ and DW (*P* < 0.05), which were determined to be 57.72% and 52.36% (**Figure 1H**).

Although the differences of starch content in three varieties were not significant, we still detected a contrary order compared to the soluble sugar content and TSS among DW, YZ and B21 (**Figure 1E**). We also documented that the content of TSS showed a positive correlation with total sugar content, but a negative correlation with starch content relative to fruit (Ketsa and Daengkanit, 1998), and this also confirmed our results that an increase in TSS content might lead to higher levels of soluble sugars and lower levels of starch. Titrable acidity refers to the total acid content in food (Sadler and Murphy, 2010), and in our study, significant differences of the acidity existed in B21 vs YZ, and B21 vs DW. It is important to note that there were significant differences in the content of crude fat among the three varieties. The presence of crude fat plays a crucial role in determining the taste of hazelnuts. The unique flavor of hazelnuts can be attributed to the substantial influence of fat content, highlighting its indispensable role in shaping the overall sensory experience. These inclusions lead to unique quality and differences in flavor among the three hazelnut varieties, and the changes of these inclusions are caused by differences of metabolites at the biochemical level. This phenomenon also seems to be observed in other fruit species, including litchi (Hai-zhi et al., 2022), durian (Keawkim and Jom, 2022), and tomato (Wang et al., 2017), with different fruit having different expression patterns. In this study, we suggested that the expression patterns of sugar, organic acid and lipid metabolites of three different hazelnut varieties may be different, which is the key factors to trigger their unique flavor.

### 3.2 Screening of differential metabolites and function annotation and enrichment of fresh *C. heterophylla* × *C. avellana nuts*

**Figure 2:**
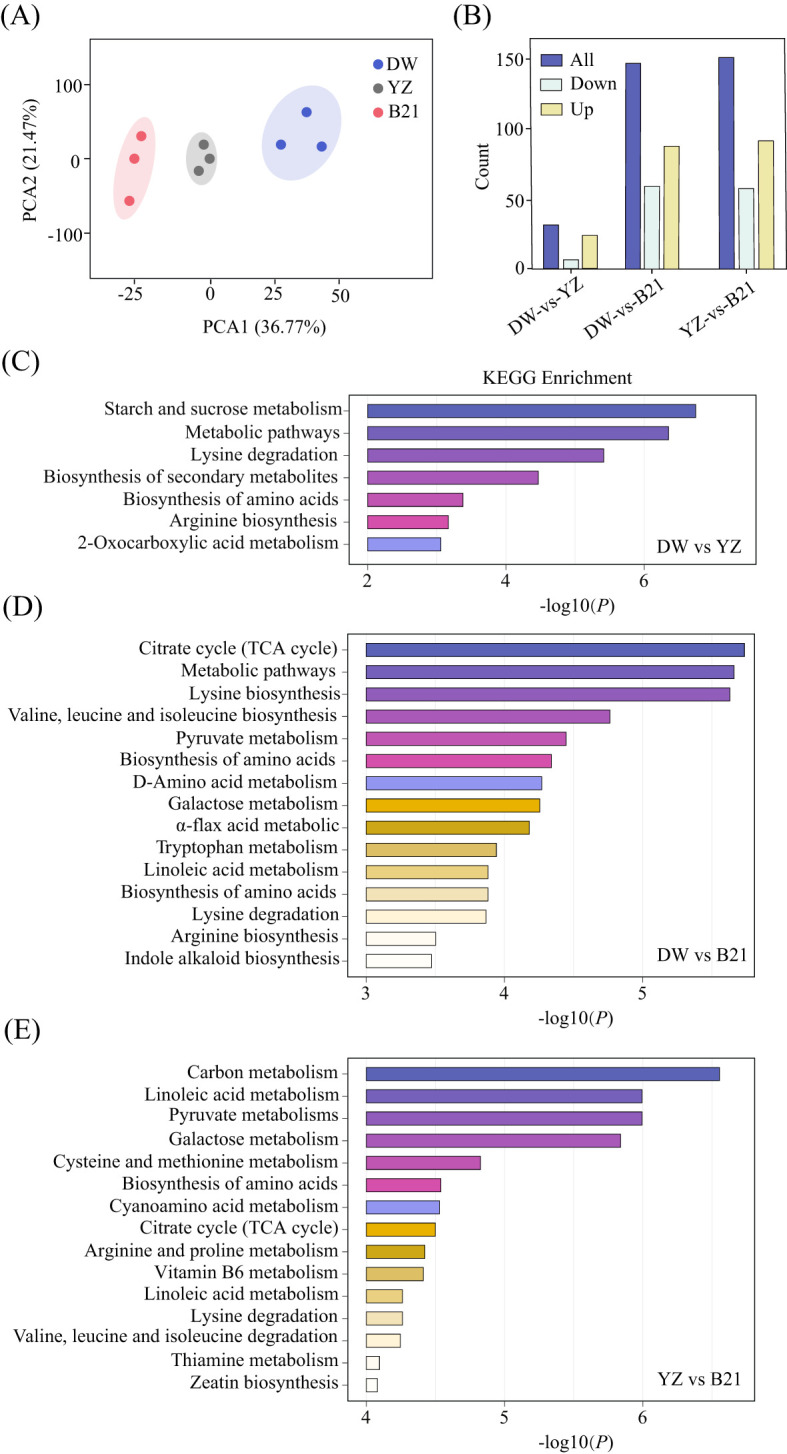
The PCA analysis of fresh *C.heterophylla × C.avellana* **(A)**. Bar graph of differential metabolites in fresh *C.heterophylla × C.avellana* **(B)**, KEGG enrichment analysis of DW-VS-YZ **(C)**, KEGG enrichment analysis of DW-VS-B21 **(D)**, KEGG enrichment analysis of YZ-VS-B21 **(E)** of three varieties of fresh *C*. *heterophylla × C. avellana nuts* (DW Dawei, YZ Yuzhui, and B21 Pingou21).

We utilized metabolomic data to identify relevant metabolites and pathways associated with the flavor of *C.heterophylla* × *C.avellana*. A comparative study was carried out to screen differential metabolites of DW-VS-YZ, DW-VS-B21 and YZ-VS-B21. The results showed that 661 primary metabolites were identified in the fresh nuts of *C. heterophylla* × *C. avellana* using UPLC-MS/MS technology, which were divided into six groups: amino acids and their derivatives, organic acids, lipids, nucleotides and their derivatives and the other categories including sugars and vitamins. (**Supplementary Table S1**). PCA analysis showed that PC1 accounted for 36.77% of the total variation and PC2 accounted for 21.47% (**Figure 2A**). There were 31 differential metabolites in DW-VS-YZ, of which 24 were up-regulated and 7 were down-regulated in DW compared with YZ (**Figure 2B**). In DW-VS-B21, 147 differential metabolites were identified, 87 were up-regulated and 60 were down-regulated in DW compared with B21 (**Figure 2B**). YZ-VS-B21 had 150 differential metabolites, with 92 up-regulated and 58 down-regulated in YZ compared with B21 (**Figure 2B**). Major contributors to nuts flavor and nutritional composition include organic acids, amino acids, sugars, and lipids (Fei et al., 2022). A total of 30 flavor related metabolites, which including 20 amino acids and derivatives, 1 organic acids, 3 lipids, and 6 sugars, were identified in DW and YZ. Of these metabolites, 24 were up-regulated (showing increased levels) and 6 were down-regulated (showing decreased levels) in the DW variety compared to the YZ variety (**Supplementary Table S2)**. A total of 138 flavor related metabolites, which including 87 amino acids and derivatives, 19 organic acids, 19 lipids, and 13 sugars, were identified in DW and B21. Of these metabolites, 79 were up-regulated (showing increased levels) and 59 were down-regulated (showing decreased levels) in the DW variety compared to the B21 variety(**Supplementary Table S3**). A total of 135 flavor related metabolites, which including 84 amino acids and derivatives, 21 organic acids, 17 lipids, and 13 sugars, were identified in YZ and B21. Of these metabolites, 78 were up-regulated (showing increased levels) and 57 were down-regulated (showing decreased levels) in the YZ variety compared to the B21 variety (**Supplementary Table S4**). The above results indicate significant metabolic differences between the varieties, and the trend of these differences is associated with quality indicators.

KEGG pathway enrichment analysis of DW and YZ showed that the identified substances were enriched in 31 biological pathways, of which 7 metabolic pathways were enriched. Of which, there are 4 metabolic pathways associated with flavor (**Figure 2C**). The metabolic pathways that included sugars (starch and sucrose metabolic pathway), amino acids and their derivatives (amino acid biosynthesis pathway). The differential metabolic pathways identified in the two hazelnut varieties, DW and YZ, are likely to play a key role in the formation of their distinct fresh flavors.

KEGG pathway enrichment analysis of DW and B21 showed that the identified substances were enriched in 147 biological pathways, of which 56 metabolic pathways were enriched. Of which, there are 28 metabolic pathways associated with flavor (**Figure 2D**). The metabolic pathways that included sugars (galactose metabolic pathway), organic acids (citric acid cycle (TCA cycle) metabolic pathway, pyruvate metabolic pathway), amino acids and their derivatives (amino acid biosynthesis metabolic pathway, cysteine and methionine metabolic pathway, valine, leucine and isoleucine biosynthesis metabolic pathway, etc.) and lipids (α-flax acid metabolic pathway, linoleic acid metabolic pathway). The differential metabolic pathways identified in the two hazelnut varieties, DW and B21, are likely to play a key role in the formation of their distinct fresh flavors.

KEGG pathway enrichment analysis of YZ and B21 showed that the identified substances were enriched in 150 biological pathways, of which 56 metabolic pathways were enriched. Of which, there are 30 metabolic pathways associated with flavor (**Figure 2E**). The metabolic pathways that included sugars (galactose metabolic pathway,starch and sucrose metabolic pathway), organic acids (glycolysis metabolic pathway, citric acid cycle (TCA cycle) metabolic pathway, pyruvate metabolism metabolic pathway), amino acids and their derivatives (amino acid biosynthesis metabolic pathway, tryptophan metabolic pathway, cysteine and methionine metabolic pathway, valine, leucine and isoleucine degradation metabolic pathway, etc.) and lipids (α-flax acid metabolic pathway, linoleic acid metabolic pathway). The metabolic pathways identified in the two hazelnut varieties, YZ and B21, are likely to play a key role in the formation of their distinct fresh flavors.

In this study, the different metabolites of the three sample groups (DW and YZ varieties, DW and B21 varieties, YZ and B21 varieties) were enriched into 4, 28, 30 metabolic pathways related to flavor, respectively. We screened out one particular sample group ‘YZ and B21’ to further study for a more comprehensive understanding of the flavor-related regulatory mechanisms, because YZ and B21 exhibited the highest number of differential metabolites compared with other groups.

### 3.3 Screening of differential genes and functional annotation and enrichment of fresh *C. heterophylla* × *C. avellana* nuts

**Figure 3:**
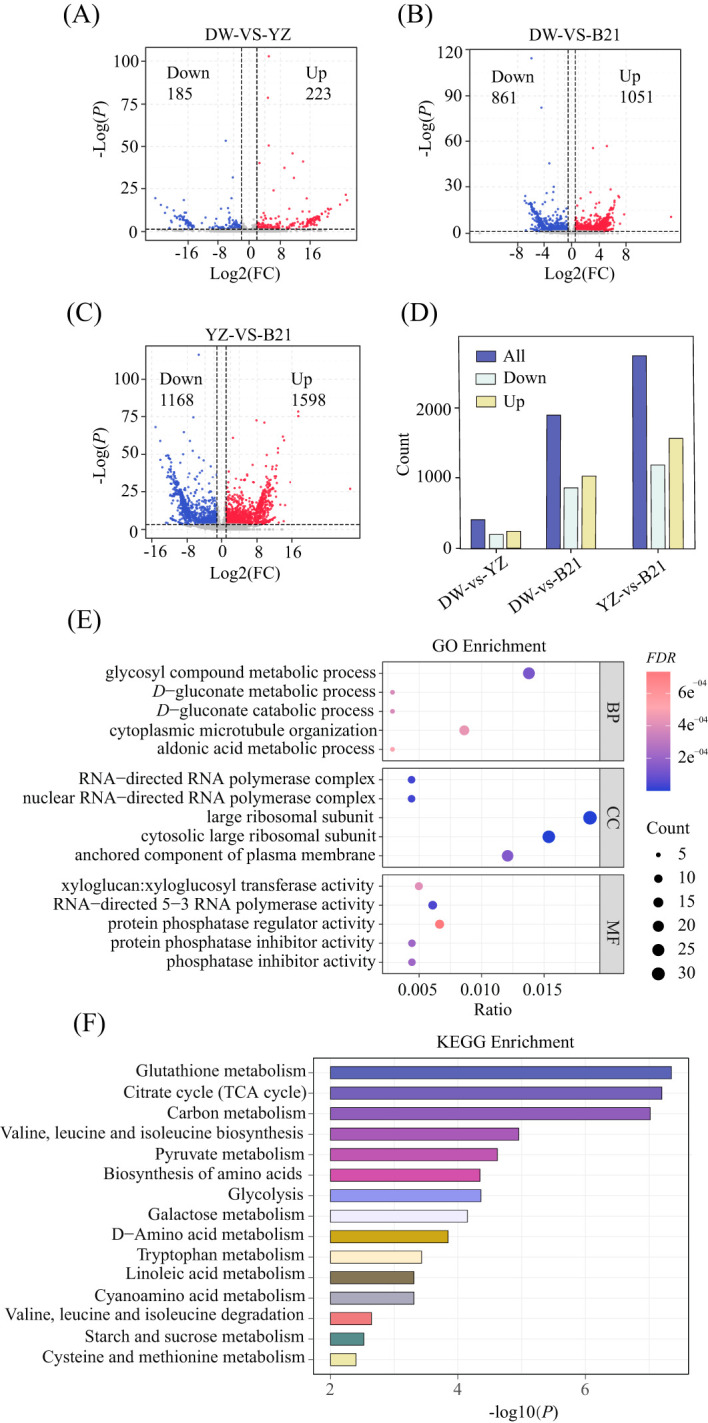
Volcano plot of DW-VS-YZ **(A)**, DW-VS-B21 **(B)** and DW-VS-B21 **(C)**. Identification of differentially expressed genes using thresholds *P* < 0.05 and fold change (FC) > 2. Bar graph of differential genes of three fresh *C.heterophylla × C.avellana* **(D)**, GO enrichment analysis of YZ-VS-B21 **(E)**. BP Biological process, CC cell components, MF molecular function. KEGG enrichment analysis of YZ-VS-B21 **(F)**.

To gain a more thorough understanding of the biochemical modifications within the identified flavor-related metabolites and pathways in fresh *C.heterophylla*×*C.avellana* nuts, we integrated transcriptome analysis to investigate the expression of candidate genes involved. In order to screen differentially expressed genes (DEGs), transcriptome data was compared between DW vs. YZ, DW vs. B21, and YZ vs. B21. According to the volcano diagram (**Figures 3A–C**) and bar graph (**Figure 3D**) shown in **Figure 3**, a total of 408 differential DEGs were screened between DW and YZ, with 185 genes being down-regulated and 223 genes up-regulated in DW compared with YZ, accounting for 45.3% and 54.7% of total DEGs respectively. In the comparison between DW and B21, a total of 1912 differential DEGs were screened, with 861 genes being down-regulated and 1051 genes being up-regulated, accounting for 45.0% and 55.0% of the total DEGs, respectively. Finally, when comparing YZ and B21, a total of 2766 differential DEGs were screened, with 1168 genes being down-regulated and 1598 genes being up-regulated, respectively accounting for 42.2% and 57.8% of the total DEGs, respectively. It was noteworthy that the YZ and B21 groups had the largest number of differential DEGs.

Based on the GO annotation of DEGs, it was determined that 372 individual genes were annotated as belonging to ‘biological process’, while 271 were assigned to ‘cellular component’ and 290 were classified as ‘molecular function’ (**Figure 3E**). To explore the biological pathways associated with the DEGs data, we assigned the DEGs to KEGG (**Figure 3F**), and the results showed that DEGs were involved in 57 biological pathways in YZ and B21. Notably, several significant differences were observed in the biological pathways, including those related to sugars (galactose metabolic pathway, starch and sucrose metabolic pathway), organic acids (glycolysis metabolic pathway, citric acid cycle (TCA cycle) metabolic pathway, pyruvate metabolism metabolic pathway), amino acids and their derivatives (amino acid biosynthesis metabolic pathway, tryptophan metabolic pathway, cysteine and methionine metabolic pathway, valine, leucine and isoleucine degradation metabolic pathway, etc.) and lipids (α-flax) acid metabolic pathway, linoleic acid metabolic pathway). These outcomes are in line with the metabolic pathway analysis, which indicated that changes in sugar metabolism, central carbon metabolism and lipid metabolism are closely linked to the flavor of the edible hazelnut varieties.

### 3.4 Screening of candidate genes for flavor metabolic pathway of fresh *C. heterophylla* × *C. avellana* nuts

**Figure 4:**
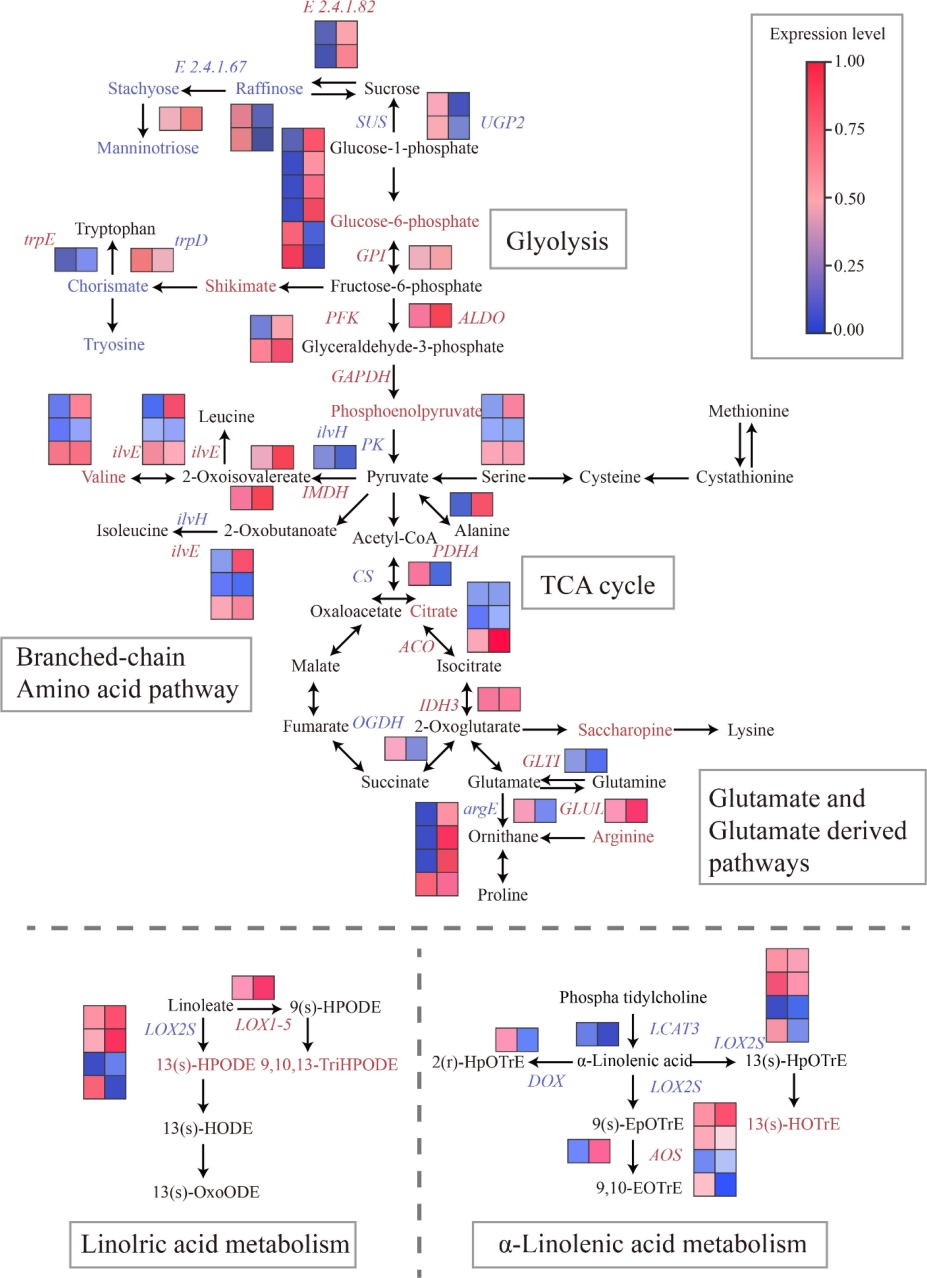
Biosynthetic pathway of flavor in fresh *C.heterophylla × C.avellana*. Significantly altered metabolites and genes of the tasting-related pathways in the *C.heterophylla × C.avellana* cultivar metabolic map. Metabolites with significantly increased, decreased, and unchanged content between YZ and B21 are outlined in red, green, and blue, respectively. Up-regulated, down-regulated and up-down-regulated genes are colored in red, green and blue, respectively. Solid arrows indicate one enzymatic reaction.

In our study, we discovered that the distinct flavor characteristics of two fresh-eaten hazelnut varieties (YZ and B21) are significantly influenced by central carbon pathways (glycolysis and the TCA cycle) and lipid metabolism pathways. These metabolic pathways play a crucial role in shaping the unique flavor of these hazelnut varieties. Sugars are metabolized through the central carbon pathway, which generates cellular energy as well as organic acids that contribute to fruit sourness and serve as precursors for amino acid biosynthesis (Crisosto et al., 2004). In this section, our focus was on investigating the regulation of the central carbon pathway and its connection to the flavor-related pathway. Therefore, we constructed a metabolic network to elucidate the relationship between changes in metabolite levels and candidate genes (**Figure 4**; **Supplementary Table S5**).

### 3.5 Flavor biosynthetic pathways: central carbon pathway

Fruit taste can be classified as either sweet or sour, which is closely associated with its composition, particularly the levels of sugars and organic acids (Wang et al., 2017; Minas et al., 2013). The flavor quality of mangoes is primarily influenced by sugars and organic acids involved in carbohydrate metabolism. The content and types of these different metabolites may largely determine the flavor of mangoes (Peng et al., 2022). In this study, the expression of manninotriose, stachyose, and raffinose was found to be down-regulated in YZ compared with B21. Additionally, *E2.4.1.67* was down-regulated, while *E2.4.1.82* and *INV* were up-regulated in YZ compared with B21. These results suggest that manninotriose, stachyose, and raffinose could potentially be the main contributors to the sugar profile affecting the flavor of fresh hazelnuts. Furthermore, glucose-6-phosphate expression was up-regulated in YZ compared with B21. Genes in the glycolytic pathway, such as *HK*, *PFK*, and *PK*, control the rate-limiting steps of glycolysis in fruits (Ball et al., 1991). Liu et al. (2021) studied the remodeling of GPI in polysaccharide and lipid components necessitates the interaction between GPI aminotransferase and protein. Changes in GAPDH upstream of glycolytic intermediates are the result of thermodynamic re-equilibration in response to perturbations of GAPDH and do not necessarily indicate glycolytic inhibition. Increases in FBP, DHAP, and GA3P correlated with changes in glycolytic rates only when GAPDH-SA was inhibited beyond the critical point (Zhu et al., 2021). Our results indicated that *GPI*, *PFK*, *ALDO*, and *GAPDH* were up-regulated with high expression values in YZ compared with B21. *PK* expression showed both upregulation and downregulation in YZ compared with B21, while the expression of pyruvate (the end product of the pathway) was unchanged (**Figure 4**; **Supplementary Table S5**). These findings suggest that *GPI*, *PFK*, *ALDO*, *GAPDH*, and *PK* genes play a role in the glycolysis of hazelnuts. Furthermore, an upregulation of the PDHA in YZ compared with B21 (**Figure 4**) was observed, indicating that pyruvate is converted to acetyl-coa and enters the TCA cycle through pyruvate dehydrogenase (PDH). The first step in the TCA cycle involves the production of citrate, a process regulated by CS.

Despite the downregulation of CS, there was an increase in the expression of citrate in YZ compared with B21 (**Figure 4**; **Supplementary Table S5**). Additionally, we noticed an upregulation of other genes associated with the TCA cycle, such as *ACO* and *IDH3*, while the expression of metabolites (isocitrate, succinate, fumarate, and malate) remained unchanged in YZ compared with B21 (**Figure 4**; **Supplementary Table S5**). These findings suggest the activation of the TCA cycle, which supports the biosynthesis of citrate in YZ. Citrate plays a vital role in glutamate metabolism and is converted to 2-Oxoglutarate. Notably, the gene *IDH3*, responsible for the biosynthesis of 2-Oxoglutarate, was up-regulated, while the gene *OGDH*, involved in its degradation, was down-regulated in YZ compared with B21 (**Figure 4**). This indicates that the expression of 2-Oxoglutarate remains constant as it enters the TCA cycle through OGDH and participates in glutamate metabolism via an alternative pathway. Similar observations have been reported in other fruits, such as tomatoes (Zhang and Fernie, 2018), suggesting a correlation between changes in citrate levels and the expression of 2-Oxoglutarate. The TCA cycle is found in animal, plant, and microbial cells. It not only provides essential cellular energy and carbon skeleton substances but also contributes to the catabolism of glucose, lipids, and proteins (Fei et al., 2022). Although some of the metabolites in this pathway might not contribute significantly to the overall taste of fresh *C. heterophylla* × *C. avellana*, they serve as important precursors for the biosynthesis of other taste-related compounds, which will be discussed in detail later on.

### 3.6 Flavor biosynthetic pathways: amino acid metabolism pathways

Amino acid composition and richness play a vital role in determining the nutritional quality and flavor of fruits (Choi et al., 2011). For example, the presence of free amino acids in tea leaves can indeed play a crucial role in determining their taste (Wang et al., 2011). Additionally, these amino acids are widely utilized as food additives to enhance the umami taste in various dishes (Obretenov et al., 2002). Salman et al. (2021) studied it is important to highlight that the type, content, and proportion of amino acids can vary greatly among different plant-based foods. Therefore, the study of amino acid composition in different fruits has important reference value for fruit flavor. Previous studies on loquat fruits have identified 17-18 amino acids (Zheng et al., 2013). In our study, we identified a total of 84 amino acids, four of which showed differential accumulation between YZ and B21. The expression of four amino acids (Tryptophan, Valine, Alanine, Arginine) was increased in YZ compared with B21 (**Figure 4**; **Supplementary Table S5**). Notably, previous studies have demonstrated that arginine can enhance protein deposition in meat products, thereby improving their quality (Hao et al., 2005). We observed a significant up-regulation of glutamate related biosynthetic genes *GLTI* expression in YZ compared with B21 (**Figure 4**), while the glutamate content remained unchanged. The result suggests that the rate of biosynthesis and consumption is relatively equal. Additionally, glutamic acid acts as a precursor for the biosynthesis of ornithine and arginine, which are necessary for polyamine production. Sangpong L, et al (Dou et al., 2023) studied these two amino acids can be converted into putrescine, a vital precursor for further polyamine synthesis. We observed that *argD* expression was down-regulated in YZ compared with B21, while ornithine expression remained unchanged and arginine expression was up-regulated (**Figure 4**). Based on these findings, we propose that the primary pathway for polyamine biosynthesis in *C.heterophylla* × *C.avellana* flesh is through the arginine pathway, as indicated by the upward-regulation and down-regulation of the key gene *argE* expression in YZ and B21 (**Figure 4**). Moreover, the expression levels controlling the conversion from ornithine to putrescine showed no significant alterations. Hao et al. (Wang et al., 2017) studied the arginine pathway is the main pathway for polyamine biosynthesis in other fruits, such as apples and tomatoes.

Branched-chain amino acids serve as precursors for the production of volatile esters (Guo et al., 2021). Fresh *C. heterophylla* × *C. avellana* varieties are known for their delightful nutty aroma, which may be attributed to the volatiles associated with branched-chain amino acid metabolism. Notably, our study revealed an up-regulation of the *trpE* gene expression (**Figure 4**), resulting in the increased expression of tryptophan in the YZ variety. The results of our study suggest that variations in amino acid composition and richness have an impact on fruit flavor. However, it should be noted that the effects of PFK expression differ for each amino acid. Our findings provide support for the hypothesis that fresh Hazelnut varieties exhibit up-regulated biosynthesis of branched-chain amino acids in YZ compared with B21. These amino acids are likely utilized in the production of volatile esters, which contribute to the distinctive aroma of fresh hazelnuts. Similarly, in tea, specific amino acids such as L-theanine and γ-aminobutyric acid (GABA) contribute to its distinctive flavor (Wang et al., 2017). In summary, the genes *trpE*, *PFK*, *ALDO*, *GAPDH*, *IMDH*, *ilvE*, *PDHA*, *ilvA*, *ACO*, *IDH3*, *GLT1*, and *GLUL* showed higher expression levels in YZ compared with B21. Conversely, the expressions of *trpD*, *ilvH*, *CS*, and *argD* was downregulated in YZ compared with B21. Notably, *PK* and *argE* expression demonstrated both upregulation and downregulation in YZ compared with B21. It is important to emphasize that amino acid biosynthesis is a precise and intricate process involving numerous reaction steps (Yu and Yang, 2020).

### 3.7 Flavor biosynthetic pathways: linolric acid and α-linolenic acid metabolism pathway

Aroma is among of the most important criteria that indicate the quality of food (Muradova et al., 2023). The fragrance in hazelnuts may be derived from lipids. Unsaturated fatty acids, such as linoleic acid and oleic acid, have been reported as precursors of aroma compounds, producing volatile substances like trans-2-hexenal and methyl jasmonate, which contribute to the umami and aroma of various foods (Pastorelli et al., 2006). Some of the odors in nuts are associated with hexane-derived molecules, such as caproaldehyde, cis-3-caproaldehyde, and hexol et al., whose precursors are linolenic acid and linoleic acid fatty acids (Li et al., 2024). As an essential unsaturated fatty acid and the primary ω-6 polyunsaturated fatty acid in the western diet, free fatty acid linoleic acid plays a crucial role. Mining differentially expressed genes involved in lipid biosynthesis is vital for investigating the molecular mechanism underlying oil accumulation in *C. heterophylla* × *C. avellana*. Consequently, synthetic pathways and heat maps were constructed to visualize lipid biosynthesis pathways and illustrate the dynamic changes in gene expression levels in the two hazel varieties. Seventeen lipid-related metabolites were identified in this study, and three of them exhibited heterogeneity between the YZ and B21 varieties of fresh *C. heterophylla* × *C. avellana* (**Figure 4**; **Supplementary Table S5**). In the linoleic acid metabolism pathway, *LOX1-5* gene expression was up-regulated, *LOX2S* gene expression was up-regulated and down-regulated, and 13(S)-HPODE and 9,10, 13-TriHOME was up-regulated in YZ compared with B21. In the α-linolenic acid metabolism pathway, the expression of *AOS* gene was up-regulated, the expression of *LOX2S* gene was up-regulated and down-regulated, the expression of *LCAT3* and *DOX* gene was down-regulated, and 13(S)-HOTrE were up-regulated in YZ compared with B21. *LOX* genes play a critical role in the enzymatic oxidation of polyunsaturated fatty acids (Oenel et al., 2017). Studies on Macadamia ternifolia nuts have revealed that *LOX 6* utilizes linoleic acid/linolenic acid as a substrate to produce unsaturated fatty acids. Additionally, *GPAT*, *DGK*, *PI*, *NPC*, *PK*, *KAR*, and *LOX* enzyme coding genes are considered the most likely key genes in lipid synthesis (Shi et al., 2021). *DOX*, *LOX2S* and *LOX1-5* may be important genes in the biosynthesis of α-linolenic acid and the linoleic acid. These findings shed light on the intricate regulation of lipid biosynthesis and provide valuable insights into the molecular mechanisms underlying oil accumulation in hazel varieties during the early stages of fruit ripening.

### 3.8 Screening of key genes to the flavor related biosynthesis

**Figure 5:**
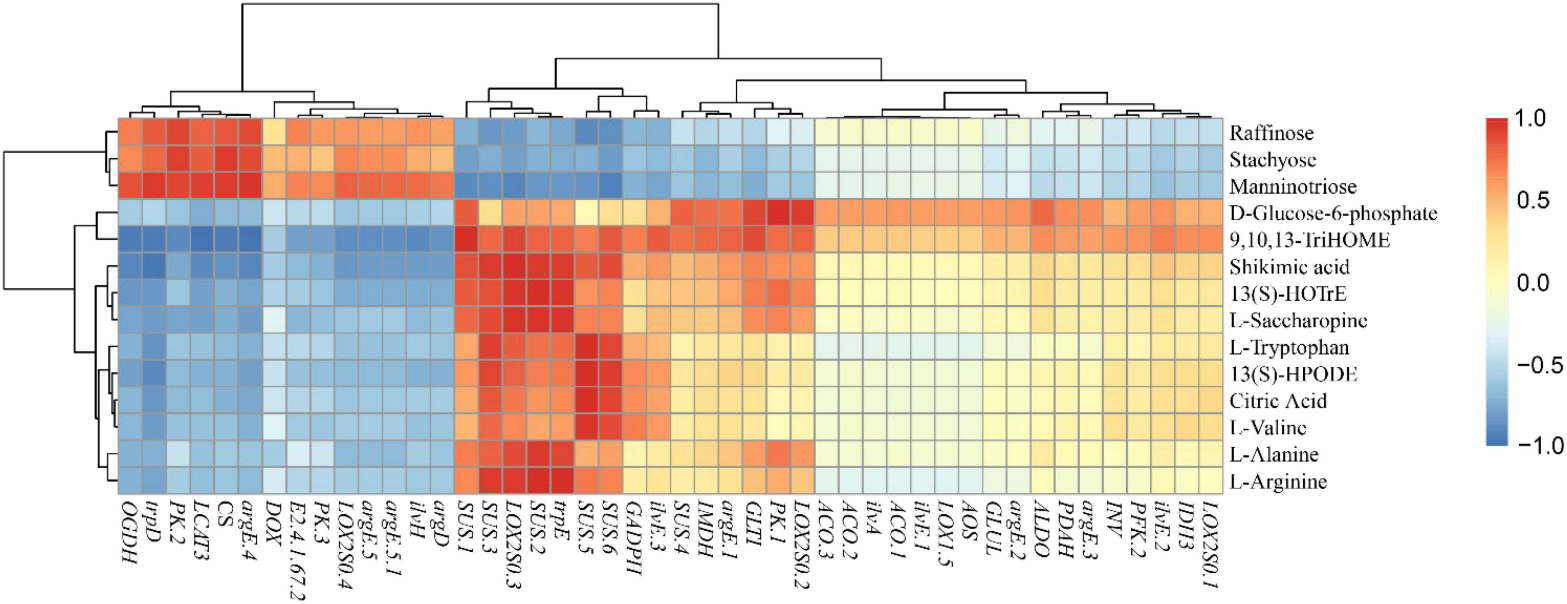
The correlation analysis of the related flavor metabolites and synthesis-related genes of fresh *C.heterophylla × C.avellana*.

**Figure 6:**
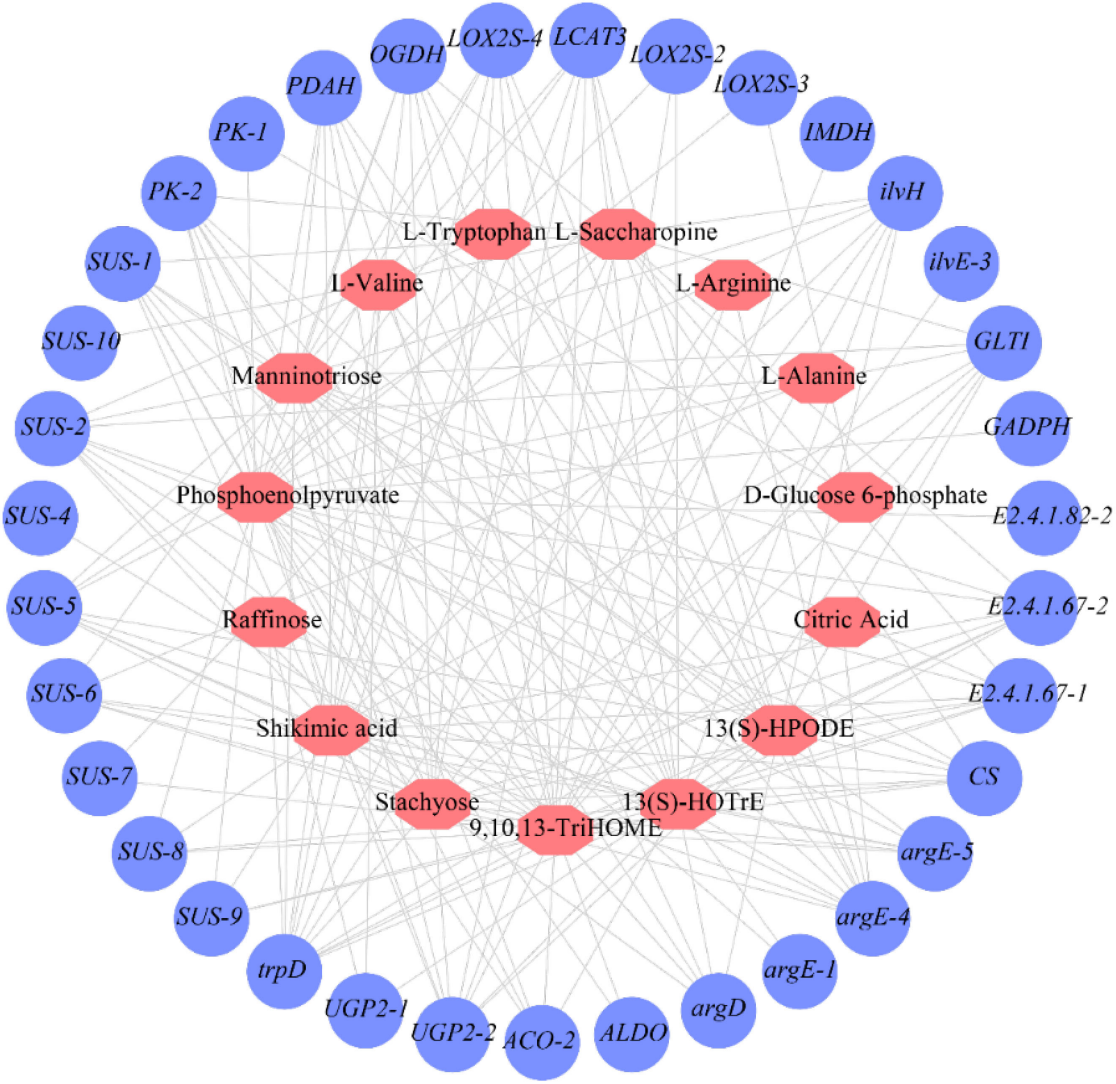
The connection network of related flavor metabolites and genes of fresh *C.heterophylla × C.avellana*.

We conducted a correlation analysis between the expression of flavor-related metabolites and the expression of synthesis related genes in fresh edible hazelnuts to identify key genes involved in the synthesis of flavor related compounds (**Figure 5**; **Supplementary Table S6**) and (**Figure 6**; **Supplementary Table S7**).

The correlation analysis between the expression levels of 56 genes and the expression levels of 15 flavor-related metabolomics revealed several significant correlations. Specifically, *trpD* and *argE-4* were found to be significantly positively correlated with raffinose. Manninotriose showed a significant positive correlation with *UGP2-1*, *UGP2-2*, *PK-2*, and *OGDH*. D-Glucose6-phosphate exhibited a highly significant positive correlation with *PK-1* and *LOX2S-2*. On the other hand, *PFK-1* and *PFK-2* displayed negative correlations with raffinose, manninotriose, and stachyose, while being positively correlated with D-Glucose6-phosphate. *CS* showed a highly significant positive correlation with stachyose and manninotriose. Among the *SUS* genes, *SUS-3*, *SUS-5*, *SUS-6*, and *SUS-7* demonstrated significant negative correlations with raffinose. Furthermore, a significant negative correlation was observed between *SUS-1*, *SUS-2*, and *SUS-3* and manninotriose (**Figure 5**; **Supplementary Table S6**). Based on the findings of the study, it can be inferred that *PFK*, *PK* and *SUS* coding genes may be the rate-limiting steps controlling fruit glycolysis. Rate-limiting steps controlling fruit glycolysis may involve *HK*, *PFK*, and *PK* genes in the glycolytic pathway (Dou et al., 2023).

L-tryptophan, L-valine, L-saccharine and L-alanine were positively correlated with *PK-1* and negatively correlated with *PK-2* and *PK-3*, indicating that they were actively involved in amino acid biosynthesis. These findings suggest that *PK-1*, *PK-2* and *PK-3* might be the primary genes responsible for amino acid biosynthesis (**Figure 5**; **Supplementary Table S6**). On the other hand, *PFK-1* and *PFK-2* showed a negative correlation with L-arginine but a positive correlation with D-Glucose6-phosphate, shikimic acid, 9,10,13-TriHOME, and 13(S)-HOTrE. Phosphofructokinase (PFK), as an upstream gene of the amino acid synthesis pathway, directly influences the synthesis of various amino acids, although the specific effects on different amino acids may vary (Liu et al., 2021). Regarding LOX2SO-3, 13(S)-HPODE, 9,10,13-TriHOME, and 13(S)-HOTrE were found to be significant positive correlated. Notably, there was a significant positive correlation between 13(S)-HOTrE and *LOX2S-3* (**Figure 5**; **Supplementary Table S4**). These results indicate that *LOX2S-3* likely plays a significant role in the synthesis of 13(S)-HPODE, 9,10,13-TriHOME, and 13(S)-HOTrE.

A total of 129 significant (*P*<0.05) and 61 highly significant (*P*<0.01) correlations were detected between 35 genes and 15 metabolites (**Figure 6**; **Supplementary Table S7**). The genes in the cluster showed close relationships with flavor metabolites. Fourteen genes (*SUS-1*, *SUS-5*, *PDAH*, *GLTI*, *LOX2S-3*, *E2.4.1.67-1*, *E2.4.1.67-2*, *PK-2*, *CS*, *OGDH*, *ilvH*, *argD*, *argE-5*, *LOX2S-4*) were highly significantly correlated with 9,10,13-TriHOME and shikimic acid. Three genes (*SUS-2*, *UGP2-2*, *trpD*) were highly significantly correlated with stachyose, manninotriose, and shikimic acid. One gene (*argE-4*) was highly significantly co-associated with stachyose, manninotriose, phosphoenolpyruvate, L-tryptophan, and L-arginine. One gene (*trpD*) was highly significantly associated with stachyose, manninotriose, shikimic acid, and phosphoenolpyruvate. One gene (*UGP2-2*) was highly significantly associated with stachyose, manninotriose, shikimic acid, and 13(S)-HOTrE (**Figure 6**; **Supplementary Table S7**). The fact that 9,10,13-TriHOME and shikimic acid share the largest number of highly significant genes suggests that they may have evolved similar mechanisms of accumulation. The genes shared by stachyose and manninotriose were *SUS-2*, *UGP2-2*, *trpD*, *argE-4*, and *OGDH*, while *argD* was a highly significant gene regulating manninotriose. Phosphoenolpyruvate is significantly positively correlated with *argE-1* and *LOX2S-2*; It was highly significantly negatively correlated with *SUS-8* and *SUS-9*. D-Glucose6-phosphate was positively correlated with *PK-1*. There was a highly significant negative correlation with *LCAT-3*. *SUS-4* and *ALDO* were highly significantly positively correlated with 9,10, 13-Trihome. *SUS-7* was highly significantly positively correlated with L-saccharopine. This indicates that stachyose and manninotriose may have evolved a similar accumulation mechanism. We found that these 27 genes were strongly associated with the 11 relevant flavor metabolites (**Figure 6**; **Supplementary Table S7**).

Joint analysis of metabolome and transcriptome and correlation analysis showed that: *E2.4.1.67-1*, *E2.4.1.67-2*, *SUS-1*, *SUS-2*, *SUS-4*, *SUS-5*, *SUS -7*, *SUS-8*, *SUS-9*, *UGP2-2*, *CS*, *OGDH*, *trpD*, *ALDO*, *PK-1*, *PK-2*, *ilvH*, *argE-1*, *argE-4*, *argE-5*, *argD*, *PDAH*, *GLTI*, *LOX2S-2*, *LOX2S-3*, *LOX2S-4* and *LCAT3* were identified as potential key genes in the flavor biosynthesis of fresh *C. heterophylla* × *C. avellana* (YZ and B21 varieties). Of which, *E2.4.1.67-1*, *E2.4.1.67-2*, the *SUS* coding genes (*SUS-1*, *SUS-2*, *SUS-4*, *SUS-5*, *SUS-7*, *SUS-8*, and *SUS-9*), and *UGP2-2* were identified as responsible for regulating the levels of stachyose, manninotriose, and raffinose in hazelnuts. *CS* and *OGDH* were deemed as the genes involved in the citric acid cycle, which was a central metabolic pathway that generated energy through the oxidation of carbohydrates, fats, and proteins in hazelnuts. Genes such as *trpD*, *ALDO*, *PK-1*, *PK-2*, *ilvH*, *argE-1*, *argE-4*, *argE-5*, *argD*, *PDAH*, and *GLTI* were regarded as involved in the biosynthesis of various amino acids like tryptophan, valine, alanine, and arginine. The genes *LOX2S-2*, *LOX2S-3*, *LOX2S-4*, and *LCAT3*, were viewed as involved in the regulation of lipid biosynthesis. Specifically, they play an important role in the production of compounds like 13(S)-HPODE, 9,10,13-trihome, and 13(S)-HOTrE in *C. heterophylla × C. avellana*. However, the flavor composition of foods remains complex. In future work, we will further assess the flavor characteristics of each variety and correlate the differential metabolites to identify more distinctive flavor compounds. Additionally, further studies are needed to explore the specific effects of candidate genes on hazelnuts flavor. For genes with identified functions, identification of favorable variants within the population for molecular marker-assisted breeding or genomic selection breeding is the key to breeding high-quality hazelnut varieties. Meanwhile, the combination of gene editing, synthetic biology and other high technologies will further accelerate the breeding of superior varieties.

## 4 Conclusions

In this study, the different metabolites of the three sample groups (DW vs. YZ varieties, DW vs. B21 varieties, YZ vs. B21 varieties) were enriched into 4, 28, 30 metabolic pathways related to flavor, respectively. We screened out one particular sample group ‘YZ and B21’ to further study for a more comprehensive understanding of the flavor-related regulatory mechanisms, because YZ and B21 exhibited the highest number of differential genes, and they associated with more different metabolites compared with ‘DW and B21’ and ‘DW and YZ’. The results showed that the higher crude fat content in YZ was 57.72%, the higher soluble sugar content and titrable acid content in B21 were 16.65% and 0.51%. Metabolome analysis showed that the expressions of mannose, stachyose and raffinose were down-regulated, while citrate, tryptophan, valine, alanine and arginine were up-regulated in YZ compared with B21. Meanwhile, metabolites associated with lipid biosynthesis (13(S)-HPODE and 9, 10, 13-TriHOME, and 13(S)-HOTrE) were up-regulated in YZ compared with B21. The change of the above metabolites may be the main factor causing the taste difference between YZ and B21 varieties. Joint analysis of metabolome and transcriptome and correlation analysis showed that: *E2.4.1.67-1*, *E2.4.1.67-2*, *SUS-1*, *SUS-2*, *SUS-4*, *SUS-5*, *SUS -7*, *SUS-8*, *SUS-9*, *UGP2-2*, *CS*, *OGDH*, *trpD*, *ALDO*, *PK-1*, *PK-2*, *ilvH*, *argE-1*, *argE-4*, *argE-5*, *argD*, *PDAH*, *GLTI*, *LOX2S-2*, *LOX2S-3*, *LOX2S-4* and *LCAT3* were identified as potential key genes in the flavor biosynthesis of fresh *C. heterophylla × C. avellana* (YZ and B21 varieties). Of which, *E2.4.1.67-1*, *E2.4.1.67-2*, the *SUS* coding genes (*SUS-1*, *SUS-2*, *SUS-4*, *SUS-5*, *SUS-7*, *SUS-8*, and *SUS-9*), and UGP2-2 were identified as responsible for regulating the levels of stachyose, manninotriose, and raffinose in hazelnuts. These sugars could potentially be the main contributors to the sugar profile affecting the flavor of fresh hazelnuts. *CS* and *OGDH* were deemed as the genes involved in the citric acid cycle, which was a central metabolic pathway that generated energy through the oxidation of carbohydrates, fats, and proteins in hazelnuts. Genes such as *trpD*, *ALDO*, *PK-1*, *PK-2*, *ilvH*, *argE-1*, *argE-4*, *argE-5*, *argD*, *PDAH*, and *GLTI* were regarded as involved in the biosynthesis of various amino acids like tryptophan, valine, alanine, and arginine. These amino acids determined the taste of *C. heterophylla × C. avellana* and were important precursors of other flavor-related compounds. The genes *LOX2S-2*, *LOX2S-3*, *LOX2S-4*, and *LCAT3*, were viewed as involved in the regulation of lipid biosynthesis. Specifically, they play an important role in the production of compounds like 13(S)-HPODE, 9,10,13-trihome, and 13(S)-HOTrE in *C. heterophylla × C. avellana*. These findings highlight the significance of genes and metabolites and internal regulatory mechanisms in shaping the flavor of fresh *C. heterophylla × C. avellana* cultivated in temperate continents. This study provides the theoretical basis for breeding excellent food functional hazelnut varieties.

## Data Availability

The de novo assembly of hazelnut transcriptome data and transcript abundance of hazelnut genes across different varieties are available at Figshare [https://figshare.com/s/ba37be825b2dab889d73].
