# Supplementary material for: Primary metabolomics and transcriptomic techniques were used to explore the regulatory mechanisms that may influence the flavor characteristics of fresh Corylus heterophylla × Corylus avellana
Source: Front Plant Sci. 2025 Jan 30;15:1475242. doi: 10.3389/fpls.2024.1475242 (PMC11821611; doi:10.3389/fpls.2024.1475242)
Supplement: Supplementary file 1 [file Table1.docx]

| **Supplementary Table S1. Metabolites from UPLC-MS/MS** | | | | | | | | |
| --- | --- | --- | --- | --- | --- | --- | --- | --- |
| **Class I** | **Class II** | **Metabolite** | **DW** | | **YZ** | | **B21** | |
|  |  |  | **Average** | **SD** | **Average** | **SD** | **Average** | **SD** |
| **mino acids and derivatives** | **mino acids and derivatives** | Lys-Gly | 3.33E+06 | 4.64E+05 | 3.28E+06 | 6.07E+05 | 4.03E+06 | 1.34E+06 |
|  |  | Abu-Ile-OH | 6.24E+05 | 3.23E+05 | 1.03E+06 | 3.94E+05 | 3.76E+05 | 3.16E+05 |
|  |  | Cycloleucine | 3.13E+06 | 6.45E+05 | 3.91E+06 | 1.01E+06 | 8.49E+06 | 3.14E+06 |
|  |  | γ-Glutamylphenylalanine | 1.91E+04 | 3.73E+03 | 1.86E+04 | 8.33E+03 | 6.89E+04 | 4.83E+04 |
|  |  | Ser-Asn-Ser | 5.41E+03 | 4.23E+03 | 4.02E+05 | 6.41E+05 | 3.97E+03 | 3.23E+03 |
|  |  | TyrMe-Met-OH | 1.67E+06 | 4.25E+05 | 1.33E+06 | 1.68E+05 | 5.72E+05 | 6.67E+05 |
|  |  | Cis-L-3-hydroxyproline* | 2.05E+05 | 1.79E+04 | 2.14E+05 | 2.03E+04 | 2.14E+05 | 9.83E+04 |
|  |  | N-Palmitoylglycine | 5.16E+03 | 7.24E+02 | 7.80E+03 | 5.69E+02 | 6.27E+03 | 5.93E+02 |
|  |  | N-Acetyl-L-glutamic acid | 1.12E+06 | 4.99E+05 | 1.27E+06 | 2.21E+05 | 3.34E+06 | 6.67E+05 |
|  |  | Asn-Gly-Ser-Pro-Ser | 1.80E+05 | 2.88E+04 | 2.48E+05 | 1.60E+05 | 1.21E+05 | 6.01E+04 |
|  |  | Tyrosyl-tryptophan | 2.73E+05 | 9.90E+04 | 1.61E+05 | 1.39E+04 | 5.26E+05 | 3.99E+05 |
|  |  | His-Tyr-Thr | 4.96E+04 | 1.67E+03 | 4.86E+04 | 1.39E+04 | 1.02E+05 | 4.54E+04 |
|  |  | Thr-Arg-Tyr | 9.19E+04 | 9.24E+03 | 9.45E+04 | 2.56E+04 | 7.05E+04 | 5.31E+04 |
|  |  | Met-Tyr-Tyr | 2.18E+05 | 3.27E+04 | 2.63E+05 | 2.94E+04 | 5.02E+04 | 3.38E+04 |
|  |  | L-Phenylalanine | 7.91E+06 | 8.49E+05 | 8.66E+06 | 5.69E+05 | 1.75E+07 | 1.45E+07 |
|  |  | L-Leucine* | 1.97E+06 | 8.27E+05 | 1.78E+06 | 1.11E+06 | 7.88E+06 | 7.71E+06 |
|  |  | Ser-His | 1.12E+05 | 7.57E+03 | 8.99E+04 | 9.20E+03 | 1.30E+05 | 2.30E+04 |
|  |  | Lys-Phe-Leu-Glu | 4.84E+05 | 2.61E+04 | 3.66E+05 | 8.30E+04 | 6.25E+05 | 2.05E+05 |
|  |  | Gly-Phe-Asn-Thr-Phe | 1.75E+05 | 1.40E+05 | 1.67E+05 | 8.87E+04 | 8.92E+04 | 3.75E+04 |
|  |  | L-Lysine | 3.32E+06 | 5.40E+05 | 5.37E+06 | 2.72E+06 | 8.64E+06 | 6.84E+06 |
|  |  | 1-Amino-1-cyclobutane-carboxylic-acid | 6.93E+05 | 1.13E+05 | 1.11E+06 | 2.22E+05 | 2.92E+06 | 1.75E+06 |
|  |  | Leu-Thr | 4.60E+05 | 1.33E+05 | 3.68E+05 | 4.53E+04 | 4.90E+05 | 2.26E+05 |
|  |  | His-Thr-Ser-Asp | 2.92E+05 | 3.73E+04 | 1.85E+05 | 8.41E+03 | 1.59E+05 | 9.66E+04 |
|  |  | N-Acetyl-L-Tryptophan | 4.99E+04 | 3.59E+03 | 5.39E+04 | 1.09E+04 | 1.08E+06 | 8.13E+05 |
| **Class I** | **Class II** | **Metabolite** | **DW** | | **YZ** | | **B21** | |
|  |  |  | **Average** | **SD** | **Average** | **SD** | **Average** | **SD** |
| **mino acids and derivatives** | **mino acids and derivatives** | S-Ribosyl-L-homocysteine | 1.64E+06 | 4.78E+05 | 1.62E+06 | 1.76E+05 | 2.62E+06 | 6.98E+05 |
|  |  | Lys-Asp-Trp-Ser-Phe | 1.37E+05 | 1.75E+04 | 9.21E+04 | 1.22E+04 | 1.52E+05 | 6.48E+04 |
|  |  | His-Tyr-Asp | 5.86E+05 | 1.90E+05 | 5.38E+05 | 1.31E+05 | 3.87E+05 | 1.66E+05 |
|  |  | Tyr-Asn-Asp-Lys | 7.19E+04 | 3.09E+04 | 6.85E+04 | 6.56E+03 | 3.44E+04 | 1.03E+04 |
|  |  | L-Asparagine | 1.52E+05 | 3.73E+04 | 2.20E+05 | 5.87E+04 | 1.21E+05 | 5.38E+04 |
|  |  | N-acetyl-beta-alanine | 4.18E+04 | 4.66E+03 | 3.85E+04 | 3.76E+03 | 1.58E+04 | 5.93E+03 |
|  |  | Lys-Ala | 6.07E+04 | 1.04E+04 | 6.28E+04 | 4.71E+03 | 7.33E+04 | 2.61E+04 |
|  |  | L-Aspartic acid | 2.41E+05 | 7.28E+04 | 2.01E+05 | 1.19E+04 | 3.10E+05 | 1.09E+05 |
|  |  | His-Met-Glu | 3.19E+06 | 4.86E+05 | 3.68E+06 | 6.40E+05 | 1.00E+06 | 7.07E+05 |
|  |  | N-Glycyl-L-leucine* | 4.23E+04 | 1.61E+04 | 3.03E+04 | 6.32E+03 | 2.44E+04 | 1.30E+04 |
|  |  | Ile-Abu-OH | 4.05E+04 | 6.63E+04 | 4.38E+05 | 4.37E+05 | 7.91E+03 | 1.32E+04 |
|  |  | Lys-Met-His | 1.73E+07 | 3.02E+06 | 1.67E+07 | 7.22E+05 | 1.58E+07 | 3.16E+05 |
|  |  | Oxiglutatione | 3.07E+06 | 8.51E+05 | 2.25E+06 | 3.02E+05 | 1.75E+06 | 2.80E+05 |
|  |  | Pro-Met-Asp | 8.32E+04 | 2.06E+04 | 8.28E+04 | 7.21E+03 | 5.14E+04 | 5.93E+03 |
|  |  | Trans-4-Hydroxy-L-proline* | 2.18E+05 | 1.74E+04 | 2.16E+05 | 4.42E+04 | 2.28E+05 | 1.18E+05 |
|  |  | N-Acetylproline | 7.60E+04 | 1.93E+04 | 3.69E+04 | 7.87E+03 | 8.39E+04 | 4.15E+04 |
|  |  | Arg-Thr-Gln | 2.56E+04 | 7.76E+03 | 1.66E+04 | 6.54E+03 | 2.80E+04 | 1.95E+04 |
|  |  | L-Threonine* | 1.37E+05 | 2.90E+04 | 1.68E+05 | 6.52E+03 | 1.44E+05 | 2.06E+04 |
|  |  | Asp-Ile-Ser-Glu | 7.25E+05 | 4.33E+04 | 1.41E+06 | 7.44E+05 | 5.08E+05 | 2.85E+05 |
|  |  | Tyr-Ala-Thr-Asp | 4.20E+05 | 6.93E+04 | 4.08E+05 | 3.86E+04 | 3.28E+05 | 1.98E+05 |
|  |  | Abu-Val-OH | 1.66E+04 | 1.00E+04 | 8.68E+05 | 1.23E+06 | 1.59E+04 | 1.22E+04 |
|  |  | N-Monomethyl-L-arginine* | 3.76E+04 | 1.39E+04 | 9.72E+04 | 5.44E+04 | 6.84E+05 | 5.24E+05 |
|  |  | Tyr-Ser-Val-Glu | 1.20E+04 | 8.39E+03 | 2.55E+04 | 2.76E+04 | 6.75E+05 | 1.15E+06 |
|  |  | N,N-Dimethylglycine | 4.15E+06 | 1.89E+05 | 4.08E+06 | 8.87E+05 | 2.22E+06 | 6.10E+05 |
|  |  | Trp-Trp | 2.47E+05 | 1.33E+05 | 2.05E+05 | 3.66E+04 | 8.83E+04 | 4.59E+04 |
|  |  | N(6),N(6)-Dimethyl-L-lysine | 3.75E+06 | 7.52E+05 | 8.26E+06 | 2.94E+06 | 8.35E+07 | 8.45E+07 |
| **Class I** | **Class II** | **Metabolite** | **DW** | | **YZ** | | **B21** | |
|  |  |  | **Average** | **SD** | **Average** | **SD** | **Average** | **SD** |
| **mino acids and derivatives** | **mino acids and derivatives** | cyclo-(Gly-Phe) | 6.17E+05 | 7.50E+04 | 6.57E+05 | 6.26E+04 | 1.58E+06 | 9.34E+05 |
|  |  | Ala-Pro-Trp | 9.13E+04 | 8.69E+04 | 6.19E+04 | 5.01E+04 | 1.05E+05 | 4.87E+04 |
|  |  | 6-Hydroxydopaquinone | 1.21E+04 | 1.34E+04 | 1.31E+04 | 1.01E+04 | 2.36E+04 | 1.58E+04 |
|  |  | L-Leucyl-L-alanine | 1.52E+05 | 1.49E+05 | 1.09E+05 | 7.14E+04 | 1.31E+04 | 4.99E+03 |
|  |  | Phe-Glu-Val-Glu | 1.33E+05 | 1.49E+05 | 1.40E+05 | 4.18E+04 | 9.80E+04 | 6.39E+04 |
|  |  | Homoproline | 6.08E+04 | 1.21E+04 | 6.27E+04 | 1.09E+04 | 4.05E+05 | 3.67E+05 |
|  |  | 1-(2-Amino-4-methylpentanoyl)pyrrolidine-2-carboxylic acid | 1.56E+06 | 2.41E+05 | 1.38E+06 | 3.05E+05 | 1.09E+06 | 3.33E+05 |
|  |  | (2S)-2-amino-4-methyl-4-pentenoic acid | 3.24E+06 | 5.02E+05 | 3.96E+06 | 9.39E+05 | 9.47E+06 | 3.67E+06 |
|  |  | S-Adenosylmethionine | 2.84E+05 | 1.25E+05 | 2.50E+05 | 1.04E+04 | 5.63E+05 | 1.73E+05 |
|  |  | His-Tyr-Gln-Asp | 1.45E+04 | 3.66E+03 | 1.08E+04 | 1.20E+03 | 3.25E+04 | 2.02E+04 |
|  |  | γ-glutamylmethionine | 5.79E+04 | 1.08E+04 | 4.21E+04 | 2.03E+04 | 3.30E+05 | 1.96E+05 |
|  |  | 5-Oxoproline | 3.07E+04 | 1.20E+04 | 2.81E+04 | 4.00E+03 | 6.19E+04 | 1.15E+04 |
|  |  | Thr-Trp-Met | 3.56E+05 | 1.19E+04 | 3.66E+05 | 2.73E+04 | 3.13E+05 | 1.10E+04 |
|  |  | S-Methyl-L-cysteine | 4.48E+05 | 5.60E+04 | 5.89E+05 | 1.03E+05 | 8.74E+05 | 4.49E+05 |
|  |  | Met-TyrMe-OH | 5.59E+05 | 4.96E+04 | 5.93E+05 | 1.01E+05 | 5.19E+04 | 1.09E+04 |
|  |  | Thr-Phe | 5.88E+05 | 7.27E+05 | 9.80E+05 | 5.44E+05 | 7.70E+05 | 7.49E+05 |
|  |  | Tyr-Gln-Thr-Lys | 3.86E+05 | 4.43E+05 | 1.18E+05 | 6.34E+04 | 4.01E+04 | 2.08E+04 |
|  |  | N-α-Acetyl-L-ornithine | 3.12E+05 | 9.41E+04 | 1.26E+06 | 9.49E+05 | 2.30E+07 | 2.43E+07 |
|  |  | N-Acetyl-L-Aspartic Acid | 8.59E+04 | 7.01E+03 | 9.69E+04 | 3.06E+03 | 8.96E+04 | 4.02E+04 |
|  |  | Lys-Thr | 3.95E+04 | 4.08E+03 | 4.86E+04 | 2.51E+03 | 2.82E+04 | 8.27E+03 |
|  |  | Arginine methyl ester* | 3.70E+04 | 1.77E+04 | 1.24E+05 | 5.60E+04 | 8.60E+05 | 6.67E+05 |
|  |  | Asn-Tyr-Glu-Asp | 2.09E+06 | 2.24E+06 | 1.68E+06 | 3.92E+05 | 2.01E+06 | 1.14E+06 |
|  |  | Pro-Tyr-Asp | 1.04E+06 | 8.38E+05 | 1.14E+06 | 4.02E+05 | 9.22E+05 | 3.78E+05 |
|  |  | N-Acetyl-L-Glutamine | 2.46E+05 | 4.28E+04 | 2.32E+05 | 1.51E+04 | 3.11E+05 | 1.38E+05 |
|  |  | Jasmonoyl-L-Isoleucine | 1.80E+03 | 5.25E+01 | 3.09E+03 | 6.48E+02 | 2.84E+04 | 1.11E+03 |
|  |  | L-Norleucine | 1.11E+06 | 2.76E+05 | 1.09E+06 | 3.94E+04 | 3.54E+06 | 2.66E+06 |
| **Class I** | **Class II** | **Metabolite** | **DW** | | **YZ** | | **B21** | |
|  |  |  | **Average** | **SD** | **Average** | **SD** | **Average** | **SD** |
| **mino acids and derivatives** | **mino acids and derivatives** | Thr-Glu-Arg | 6.32E+05 | 1.55E+05 | 6.42E+05 | 1.52E+05 | 4.76E+05 | 1.28E+05 |
|  |  | His-Asp-Tyr-Lys | 3.27E+05 | 1.42E+05 | 2.69E+05 | 6.71E+04 | 4.47E+05 | 1.33E+05 |
|  |  | Gln-Gly | 2.27E+06 | 3.00E+05 | 2.32E+06 | 4.40E+05 | 2.65E+06 | 6.61E+05 |
|  |  | Trp-tyr | 1.34E+06 | 4.18E+05 | 1.60E+06 | 1.38E+05 | 1.17E+06 | 2.97E+05 |
|  |  | N-Alpha-Acetyl-L-Asparagine | 4.27E+03 | 2.63E+03 | 1.53E+04 | 1.30E+04 | 4.02E+05 | 4.46E+05 |
|  |  | L-Glutamine-O-glycoside | 4.67E+04 | 7.26E+03 | 4.41E+04 | 4.03E+03 | 5.87E+04 | 8.28E+03 |
|  |  | Ala-Tyr-Glu-Asp | 3.57E+05 | 1.54E+05 | 1.96E+05 | 2.61E+04 | 1.14E+05 | 7.55E+04 |
|  |  | Arg-Ser-Tyr | 8.62E+06 | 1.95E+05 | 7.80E+06 | 2.57E+05 | 7.66E+06 | 2.81E+05 |
|  |  | Glu-Lys-Leu-Thr-His | 6.49E+04 | 9.89E+03 | 4.55E+04 | 4.99E+03 | 5.38E+04 | 4.45E+04 |
|  |  | Met-Val-His-Leu-Thr | 9.34E+05 | 2.17E+05 | 6.94E+05 | 1.53E+05 | 8.11E+05 | 3.36E+05 |
|  |  | Val-Met | 2.66E+06 | 9.08E+05 | 1.94E+06 | 5.39E+05 | 3.33E+06 | 2.37E+06 |
|  |  | Asn-Met-Tyr | 1.51E+05 | 2.24E+04 | 1.05E+05 | 1.81E+03 | 9.83E+04 | 4.56E+04 |
|  |  | L-Isoleucine* | 1.85E+06 | 9.32E+05 | 2.33E+06 | 1.10E+06 | 7.53E+06 | 7.17E+06 |
|  |  | Glutathione reduced form | 4.69E+05 | 1.33E+05 | 5.34E+05 | 1.12E+05 | 1.26E+06 | 5.78E+05 |
|  |  | L-Homoserine* | 1.60E+05 | 3.24E+04 | 2.24E+05 | 2.82E+04 | 2.02E+05 | 4.66E+04 |
|  |  | N-Acetyl-L-phenylalanine | 1.57E+05 | 3.08E+04 | 1.12E+05 | 1.59E+04 | 2.24E+05 | 1.48E+05 |
|  |  | 2-Amino-4-hydroxy-3-methylpentanoic acid | 1.36E+07 | 4.00E+06 | 1.39E+07 | 4.23E+06 | 2.97E+07 | 1.17E+07 |
|  |  | 3-Hydroxy-3-methylpentane-1,5-dioic acid | 6.79E+05 | 5.36E+04 | 8.15E+05 | 5.06E+04 | 8.59E+05 | 4.32E+05 |
|  |  | L-Valyl-L-Leucine | 2.13E+04 | 8.34E+03 | 2.02E+04 | 2.17E+03 | 1.31E+04 | 6.83E+03 |
|  |  | Ala-Asp-Phe-Asp | 1.36E+06 | 2.41E+05 | 1.24E+06 | 1.38E+05 | 1.16E+06 | 2.13E+05 |
|  |  | TyrMe-Ser-OH | 3.60E+04 | 2.51E+04 | 5.18E+05 | 6.14E+05 | 1.32E+04 | 7.84E+03 |
|  |  | N-Acetyl-L-threonine | 2.72E+05 | 5.57E+04 | 2.13E+05 | 2.69E+04 | 2.86E+05 | 5.06E+04 |
|  |  | Met-Glu-His | 2.29E+06 | 3.27E+05 | 2.87E+06 | 3.17E+05 | 7.71E+05 | 5.29E+05 |
|  |  | N5-(1-Iminoethyl)-L-ornithine | 1.17E+05 | 2.27E+04 | 1.22E+05 | 1.31E+04 | 1.14E+05 | 2.51E+04 |
|  |  | Abu-Met-OH | 1.11E+06 | 4.38E+05 | 6.21E+05 | 8.30E+04 | 1.54E+06 | 9.37E+05 |
|  |  | Cyclo(Pro-Glu) | 7.40E+04 | 3.43E+04 | 1.17E+05 | 4.37E+04 | 6.56E+04 | 4.38E+04 |
| **Class I** | **Class II** | **Metabolite** | **DW** | | **YZ** | | **B21** | |
|  |  |  | **Average** | **SD** | **Average** | **SD** | **Average** | **SD** |
| **mino acids and derivatives** | **mino acids and derivatives** | Leu-His-Phe-Lys | 4.42E+05 | 1.01E+05 | 3.58E+05 | 6.39E+04 | 3.18E+05 | 2.45E+05 |
|  |  | Val-Gly-Asp-Ile-Val | 1.91E+06 | 2.18E+05 | 2.19E+06 | 3.91E+05 | 3.05E+06 | 1.12E+05 |
|  |  | Tyr-Tyr-Glu | 3.36E+05 | 2.83E+05 | 3.25E+05 | 2.21E+05 | 1.65E+05 | 1.51E+05 |
|  |  | Tyr-Gly | 2.61E+04 | 3.31E+03 | 2.53E+04 | 7.79E+03 | 2.08E+04 | 3.99E+03 |
|  |  | L-Histidine | 5.00E+05 | 1.03E+05 | 7.50E+05 | 1.79E+05 | 1.42E+06 | 6.11E+05 |
|  |  | His-Ser-Lys-Lys | 2.88E+05 | 1.52E+05 | 2.92E+05 | 2.09E+05 | 1.75E+05 | 1.90E+05 |
|  |  | N-Acetyl-D-phenylalanine | 2.25E+05 | 4.60E+04 | 1.47E+05 | 2.05E+04 | 2.92E+05 | 1.14E+05 |
|  |  | Asn-Gln-Gln | 6.75E+05 | 2.61E+05 | 4.70E+05 | 1.15E+05 | 4.16E+05 | 1.65E+05 |
|  |  | Lys-Tyr | 8.20E+04 | 1.08E+04 | 8.08E+04 | 1.50E+04 | 1.17E+05 | 1.02E+04 |
|  |  | N-Propionylglycine | 5.14E+04 | 1.08E+04 | 4.91E+04 | 9.20E+02 | 5.53E+04 | 1.20E+04 |
|  |  | Pro-Tyr-Asn | 3.82E+03 | 3.40E+03 | 1.19E+04 | 7.75E+03 | 3.74E+03 | 4.00E+03 |
|  |  | Met-Trp-His | 5.43E+04 | 6.43E+04 | 5.25E+04 | 1.66E+04 | 3.71E+04 | 3.12E+04 |
|  |  | L-Isoleucyl-L-Aspartate | 2.94E+04 | 1.24E+04 | 2.94E+04 | 2.70E+03 | 3.70E+04 | 2.20E+04 |
|  |  | Lys-Asp-Leu-Glu | 4.46E+04 | 1.33E+04 | 5.16E+04 | 6.91E+03 | 9.89E+04 | 8.97E+04 |
|  |  | Asn-Thr-Gln-Glu | 1.39E+04 | 1.36E+03 | 2.64E+04 | 9.71E+03 | 1.67E+04 | 1.73E+04 |
|  |  | NG,NG-Dimethyl-L-arginine* | 3.24E+04 | 1.29E+04 | 5.71E+04 | 1.25E+04 | 1.03E+05 | 4.76E+04 |
|  |  | D-Allo-Isoleucine* | 1.17E+06 | 1.73E+05 | 1.23E+06 | 8.54E+04 | 3.79E+06 | 2.91E+06 |
|  |  | Thr-Glu-Lys-Asp-Glu | 9.49E+03 | 3.27E+03 | 8.52E+03 | 4.24E+03 | 1.21E+04 | 8.73E+03 |
|  |  | Thr-Ser-Phe-Asp | 4.35E+05 | 5.28E+04 | 4.47E+05 | 3.57E+04 | 3.45E+05 | 2.04E+05 |
|  |  | Gln-Tyr | 2.28E+06 | 1.52E+06 | 5.94E+06 | 1.32E+06 | 7.56E+06 | 4.70E+06 |
|  |  | Gln-Glu-Ser-Gln-Asp | 2.73E+04 | 2.76E+04 | 8.43E+04 | 6.88E+04 | 1.97E+04 | 2.26E+04 |
|  |  | Phe-Phe-Tyr | 2.81E+04 | 6.92E+03 | 1.50E+04 | 4.04E+03 | 8.59E+03 | 2.55E+03 |
|  |  | Cys-Trp-His | 3.78E+05 | 1.41E+05 | 5.44E+05 | 1.95E+05 | 5.22E+05 | 2.00E+05 |
|  |  | L-Valine | 5.71E+05 | 1.06E+05 | 6.85E+05 | 1.35E+05 | 1.53E+06 | 7.91E+05 |
|  |  | Asp-Gln-Ser | 1.57E+05 | 8.71E+04 | 2.83E+05 | 1.67E+05 | 2.70E+06 | 2.14E+06 |
|  |  | Thr-Met | 5.42E+05 | 2.13E+05 | 5.97E+05 | 1.14E+05 | 1.21E+05 | 9.51E+04 |
| **Class I** | **Class II** | **Metabolite** | **DW** | | **YZ** | | **B21** | |
|  |  |  | **Average** | **SD** | **Average** | **SD** | **Average** | **SD** |
| **mino acids and derivatives** | **mino acids and derivatives** | Ala-Pro-Thr | 7.03E+04 | 4.57E+04 | 1.73E+05 | 2.53E+04 | 1.86E+05 | 1.18E+05 |
|  |  | L-Cysteinyl-L-glycine | 1.79E+04 | 8.73E+03 | 1.79E+04 | 6.19E+03 | 2.36E+04 | 5.83E+03 |
|  |  | Tyrosyl-Valine | 6.18E+05 | 6.01E+04 | 5.28E+05 | 3.84E+04 | 2.07E+06 | 1.15E+06 |
|  |  | L-Glutamic acid | 2.09E+07 | 6.01E+06 | 2.35E+07 | 7.52E+06 | 4.69E+07 | 2.06E+07 |
|  |  | L-Glycyl-L-isoleucine* | 3.26E+04 | 1.05E+04 | 2.57E+04 | 4.44E+03 | 2.10E+04 | 9.22E+03 |
|  |  | Lys-Lys | 6.54E+04 | 1.26E+04 | 2.42E+05 | 1.38E+05 | 1.10E+05 | 1.16E+04 |
|  |  | DL-O-tyrosine | 1.45E+04 | 1.50E+03 | 1.79E+04 | 4.57E+03 | 2.44E+04 | 7.23E+03 |
|  |  | L-α-Glutamyl-L-Glutamic Acid | 7.84E+04 | 2.65E+04 | 1.31E+05 | 1.72E+04 | 8.69E+04 | 2.83E+04 |
|  |  | Methyl 3-aminopropanoate | 2.18E+06 | 2.38E+05 | 2.13E+06 | 3.50E+05 | 1.22E+06 | 3.69E+05 |
|  |  | L-Cyclopentylglycine | 1.44E+05 | 4.37E+03 | 1.15E+05 | 1.10E+04 | 2.04E+05 | 5.49E+04 |
|  |  | Ile-Thr-Tyr-Asp | 2.45E+05 | 9.36E+04 | 2.11E+05 | 6.86E+04 | 2.56E+05 | 1.09E+05 |
|  |  | Lys-Asp | 9.36E+04 | 1.20E+04 | 1.17E+05 | 2.35E+03 | 1.16E+05 | 1.47E+04 |
|  |  | Pyroglutamic acid | 3.43E+05 | 6.10E+04 | 3.51E+05 | 3.54E+04 | 3.64E+05 | 6.92E+04 |
|  |  | Cys-Gly-Asp-Val-Met | 6.71E+03 | 2.36E+03 | 8.25E+03 | 3.78E+03 | 9.00E+00 | 0.00E+00 |
|  |  | Ser-Met | 2.08E+06 | 5.86E+04 | 1.73E+06 | 4.32E+04 | 8.00E+05 | 8.62E+04 |
|  |  | L-Tryptophan | 1.90E+06 | 2.27E+05 | 2.14E+06 | 4.27E+05 | 6.51E+06 | 4.07E+06 |
|  |  | L-Arginine | 3.48E+06 | 6.64E+05 | 6.85E+06 | 3.58E+06 | 7.22E+07 | 6.87E+07 |
|  |  | L-Glutamine | 2.63E+06 | 1.82E+05 | 4.40E+06 | 2.45E+06 | 6.87E+06 | 5.62E+06 |
|  |  | Tyr-Nap-OH | 4.82E+06 | 1.10E+06 | 2.79E+06 | 8.58E+05 | 1.45E+06 | 1.12E+06 |
|  |  | Ile-Ala | 3.16E+04 | 2.27E+04 | 3.57E+04 | 1.62E+04 | 2.18E+04 | 9.54E+03 |
|  |  | His-Gln-Val | 5.57E+04 | 6.38E+03 | 4.79E+04 | 3.24E+03 | 4.41E+04 | 3.23E+03 |
|  |  | Asn-Ile-Arg-Asp | 4.77E+05 | 2.42E+05 | 4.82E+05 | 1.05E+05 | 2.03E+05 | 3.52E+04 |
|  |  | Phe-Tyr-Val-Lys | 7.19E+04 | 1.91E+04 | 5.52E+04 | 8.92E+03 | 5.00E+04 | 3.58E+04 |
|  |  | Val-His-Ile | 7.49E+04 | 6.78E+04 | 5.33E+04 | 4.15E+04 | 9.05E+04 | 4.10E+04 |
|  |  | L-Methionine | 1.33E+05 | 3.01E+04 | 1.18E+05 | 1.69E+04 | 2.31E+05 | 1.41E+05 |
|  |  | Asn-Leu-Arg-Arg | 4.13E+04 | 5.41E+03 | 3.81E+04 | 5.25E+03 | 3.06E+04 | 7.47E+03 |
| **Class I** | **Class II** | **Metabolite** | **DW** | | **YZ** | | **B21** | |
|  |  |  | **Average** | **SD** | **Average** | **SD** | **Average** | **SD** |
| **mino acids and derivatives** | **mino acids and derivatives** | Phe-TyrMe-OH | 6.21E+03 | 1.91E+03 | 4.08E+03 | 1.06E+03 | 9.58E+03 | 6.21E+03 |
|  |  | Tyr-Ile-Val-Glu | 7.60E+04 | 4.66E+04 | 5.09E+04 | 7.21E+03 | 8.95E+04 | 3.53E+04 |
|  |  | Abu-Leu-OH | 7.67E+04 | 4.81E+04 | 1.47E+05 | 1.04E+05 | 4.50E+04 | 2.18E+04 |
|  |  | 1-Methylhistidine* | 1.57E+05 | 1.53E+04 | 1.54E+05 | 2.23E+04 | 1.75E+05 | 2.29E+04 |
|  |  | Tyr-Gln-Asn-Glu | 1.36E+04 | 1.08E+04 | 7.14E+03 | 2.31E+03 | 1.24E+04 | 6.21E+03 |
|  |  | N-Ethylglycine* | 4.24E+06 | 4.94E+05 | 4.09E+06 | 8.08E+05 | 2.20E+06 | 4.73E+05 |
|  |  | Met-Asp-Glu | 7.40E+04 | 3.91E+04 | 6.86E+04 | 9.24E+03 | 3.14E+04 | 8.96E+03 |
|  |  | Met-Glu | 1.14E+05 | 1.61E+04 | 9.45E+04 | 4.30E+04 | 8.60E+05 | 5.13E+05 |
|  |  | Tryptophan glutamic acid | 2.30E+04 | 5.48E+03 | 2.05E+04 | 4.06E+03 | 1.67E+04 | 7.25E+03 |
|  |  | (3-(carboxyamino)-2-methylpropanoyl)phenylalanine | 1.57E+04 | 3.50E+03 | 1.29E+04 | 6.15E+03 | 4.83E+04 | 3.00E+04 |
|  |  | Gln-Gln-Ser | 1.27E+05 | 3.72E+04 | 9.62E+04 | 1.67E+04 | 6.81E+04 | 1.48E+04 |
|  |  | Glu-Ser-Val-Pro-Glu | 3.51E+04 | 1.02E+04 | 2.89E+04 | 1.25E+04 | 2.25E+04 | 3.25E+03 |
|  |  | Pro-Arg-Asp | 1.59E+04 | 2.04E+03 | 1.61E+04 | 9.61E+03 | 1.09E+04 | 1.52E+03 |
|  |  | L-Methionine Sulfoxide | 1.17E+05 | 2.95E+04 | 1.21E+05 | 1.74E+04 | 1.34E+05 | 5.30E+04 |
|  |  | Phe-Asn-Ile-Glu | 1.46E+05 | 3.44E+04 | 9.57E+04 | 1.23E+04 | 4.96E+04 | 4.19E+04 |
|  |  | Ser-His-Glu-Ala-Glu | 3.94E+04 | 8.12E+03 | 2.80E+04 | 2.38E+03 | 1.29E+04 | 2.13E+03 |
|  |  | 5-L-Glutamyl-L-amino acid | 1.40E+05 | 2.07E+04 | 1.30E+05 | 2.57E+04 | 8.24E+04 | 1.44E+04 |
|  |  | Leu-Asp | 9.33E+04 | 1.18E+04 | 9.32E+04 | 6.54E+03 | 8.49E+04 | 2.30E+04 |
|  |  | Trp-Pro-Asp | 6.59E+03 | 7.64E+03 | 2.55E+04 | 1.11E+04 | 1.10E+04 | 1.56E+04 |
|  |  | 3-Methyl-L-Histidine* | 4.37E+05 | 3.25E+04 | 4.27E+05 | 2.23E+04 | 4.91E+05 | 7.34E+03 |
|  |  | L-Tyrosine | 2.76E+06 | 2.58E+05 | 3.43E+06 | 9.58E+05 | 4.68E+06 | 2.77E+06 |
|  |  | Asn-His-Phe-Asp | 1.28E+04 | 6.06E+03 | 2.39E+04 | 2.92E+04 | 6.15E+03 | 3.67E+03 |
|  |  | L-Glutamic acid-O-glycoside | 1.81E+05 | 1.69E+04 | 1.25E+05 | 2.76E+04 | 2.04E+05 | 6.76E+04 |
|  |  | Asp-Gln-Ala-Asp | 4.93E+06 | 2.46E+06 | 4.60E+06 | 1.46E+06 | 2.65E+06 | 3.01E+06 |
|  |  | Trp-Val-Arg | 1.72E+05 | 1.06E+04 | 1.68E+05 | 4.85E+03 | 1.78E+05 | 9.30E+03 |
|  |  | Leu-His | 1.26E+05 | 3.71E+04 | 1.12E+05 | 1.10E+04 | 1.22E+05 | 3.92E+04 |
| **Class I** | **Class II** | **Metabolite** | **DW** | | **YZ** | | **B21** | |
|  |  |  | **Average** | **SD** | **Average** | **SD** | **Average** | **SD** |
| **mino acids and derivatives** | **mino acids and derivatives** | His-His-Val-Ala-Tyr | 2.42E+06 | 3.08E+05 | 2.25E+06 | 1.38E+04 | 1.85E+06 | 3.65E+05 |
|  |  | Thr-Asn-Gly | 4.00E+04 | 3.41E+04 | 8.68E+04 | 2.26E+04 | 2.10E+06 | 2.22E+06 |
|  |  | S-(5'-Adenosy)-L-homocysteine | 9.69E+04 | 1.60E+04 | 7.68E+04 | 1.16E+04 | 1.01E+05 | 3.03E+03 |
|  |  | L-Citrulline | 1.70E+06 | 3.11E+05 | 3.49E+06 | 1.65E+06 | 5.90E+06 | 4.83E+06 |
|  |  | 3,4-Dehydro-DL-proline | 3.36E+04 | 6.75E+03 | 3.92E+04 | 1.58E+03 | 2.53E+04 | 1.94E+03 |
|  |  | Asp-Tyr-Ile-Glu | 5.17E+03 | 2.80E+02 | 4.65E+03 | 3.80E+02 | 3.30E+03 | 1.14E+03 |
|  |  | L-Isoserine | 3.02E+05 | 1.05E+05 | 3.06E+05 | 7.31E+04 | 2.03E+05 | 1.09E+05 |
|  |  | Ala-Arg-Ala | 1.82E+04 | 3.30E+03 | 1.47E+04 | 3.04E+03 | 1.53E+04 | 8.51E+03 |
|  |  | Gly-Gly-Asn-Glu-Glu | 2.97E+04 | 1.10E+04 | 5.93E+04 | 1.97E+04 | 2.55E+04 | 1.71E+04 |
|  |  | His-Pro-Trp | 2.47E+04 | 8.64E+03 | 2.69E+04 | 1.37E+04 | 1.21E+04 | 5.86E+03 |
|  |  | 4-Hydroxy-L-Isoleucine | 4.87E+04 | 2.24E+03 | 5.67E+04 | 1.48E+04 | 1.27E+05 | 4.99E+04 |
|  |  | Oxaceprol | 9.84E+04 | 8.33E+03 | 9.57E+04 | 3.32E+03 | 6.33E+04 | 1.88E+04 |
|  |  | L-Alanine | 4.58E+03 | 1.93E+03 | 4.56E+03 | 2.12E+03 | 1.10E+04 | 6.79E+03 |
|  |  | Trp-Asp-Phe | 1.08E+05 | 1.34E+04 | 8.43E+04 | 1.29E+04 | 7.87E+04 | 1.62E+04 |
|  |  | Ser-Lys | 2.09E+04 | 2.79E+03 | 1.64E+04 | 4.88E+03 | 2.28E+04 | 4.73E+03 |
|  |  | His-Glu-Phe-Gly-Asp | 1.73E+04 | 7.49E+03 | 1.85E+04 | 1.14E+04 | 1.23E+04 | 1.22E+03 |
|  |  | Cys-His-Thr-Gly-Leu | 7.86E+03 | 1.01E+04 | 3.92E+03 | 1.50E+03 | 1.67E+03 | 1.23E+03 |
|  |  | Glu-Gln-Lys-Arg | 2.06E+05 | 1.55E+04 | 2.08E+05 | 1.09E+04 | 1.82E+05 | 4.54E+04 |
|  |  | Lys-Thr-Phe-Glu | 6.20E+04 | 6.51E+03 | 7.09E+04 | 1.02E+04 | 8.92E+04 | 6.56E+03 |
|  |  | L-Seryl-L-Isoleucine | 2.09E+04 | 5.92E+03 | 1.49E+04 | 2.14E+03 | 1.10E+04 | 7.41E+03 |
|  |  | N,N'-Dimethylarginine;SDMA* | 2.92E+04 | 8.39E+03 | 5.16E+04 | 1.25E+04 | 1.15E+05 | 6.10E+04 |
|  |  | Asp-Phe-Ser-Arg | 9.19E+03 | 9.07E+03 | 8.22E+03 | 1.13E+04 | 3.74E+04 | 1.12E+04 |
|  |  | 3-Hydroxy-L-phenylalanine | 3.77E+06 | 5.18E+05 | 4.54E+06 | 1.02E+06 | 6.13E+06 | 3.35E+06 |
|  |  | Cyclo(Phe-Glu) | 1.33E+04 | 1.59E+03 | 1.19E+04 | 2.74E+03 | 1.24E+04 | 6.27E+02 |
|  |  | 5-Hydroxy-L-tryptophan | 1.32E+04 | 1.68E+03 | 1.53E+04 | 6.11E+02 | 1.55E+04 | 2.29E+03 |
|  |  | Asn-Asp-Tyr-Asp | 2.38E+04 | 1.09E+04 | 2.67E+04 | 6.85E+03 | 2.74E+04 | 2.76E+02 |
| **Class I** | **Class II** | **Metabolite** | **DW** | | **YZ** | | **B21** | |
|  |  |  | **Average** | **SD** | **Average** | **SD** | **Average** | **SD** |
| **mino acids and derivatives** | **mino acids and derivatives** | Met-Phe-Thr-Glu-Asp | 1.06E+05 | 2.46E+04 | 9.13E+04 | 1.67E+04 | 8.28E+04 | 6.33E+04 |
|  |  | Met-Phe-Trp | 2.41E+04 | 2.55E+03 | 2.00E+04 | 1.16E+03 | 1.29E+04 | 4.72E+03 |
|  |  | Tyr-His-Asp | 1.83E+04 | 2.39E+03 | 1.52E+04 | 4.01E+03 | 4.15E+03 | 1.69E+03 |
|  |  | N6-Acetyl-L-lysine | 1.78E+05 | 2.78E+04 | 1.91E+05 | 3.73E+04 | 2.77E+05 | 1.52E+05 |
|  |  | γ-Glutamyltyrosine | 1.48E+04 | 3.85E+03 | 1.53E+04 | 6.11E+03 | 3.84E+04 | 1.60E+04 |
|  |  | Phe-Lys-Thr-Glu | 2.67E+05 | 3.15E+04 | 2.55E+05 | 2.96E+04 | 2.49E+05 | 6.86E+04 |
|  |  | Tyr-Pro-Phe | 1.05E+04 | 5.09E+03 | 6.96E+03 | 2.54E+03 | 1.97E+03 | 6.39E+02 |
|  |  | L-threo-3-Methylaspartate | 2.24E+06 | 9.02E+05 | 2.17E+06 | 8.17E+05 | 6.52E+06 | 2.15E+06 |
|  |  | Glu-Pro-Gly-Tyr-Ser | 5.31E+04 | 9.21E+03 | 3.99E+04 | 4.66E+03 | 3.68E+04 | 2.21E+04 |
|  |  | Pro-Asp | 3.34E+04 | 7.15E+03 | 3.36E+04 | 5.89E+03 | 3.58E+04 | 3.25E+03 |
|  |  | Gln-His-Thr-Leu-Lys | 1.19E+05 | 3.23E+04 | 7.42E+04 | 8.21E+03 | 1.33E+05 | 4.69E+04 |
|  |  | L-Aspartyl-L-Phenylalanine | 5.80E+03 | 1.17E+03 | 1.08E+04 | 6.67E+03 | 9.87E+03 | 5.14E+03 |
|  |  | Tyr-His-Gly | 1.31E+05 | 2.73E+04 | 1.19E+05 | 3.88E+04 | 7.37E+04 | 2.87E+04 |
|  |  | Lys-Asn | 1.01E+05 | 8.37E+03 | 7.03E+04 | 3.58E+04 | 8.69E+04 | 7.10E+03 |
|  |  | Asp-Gly-Asp-Lys-Asn | 5.20E+04 | 2.42E+04 | 3.36E+04 | 8.44E+03 | 1.88E+04 | 1.83E+04 |
|  |  | S-Sulfo-L-Cysteine | 2.34E+04 | 4.20E+03 | 2.81E+04 | 4.52E+03 | 6.45E+04 | 2.51E+04 |
|  |  | Asp-His-Phe-Asp | 1.65E+04 | 6.16E+03 | 2.09E+04 | 7.12E+03 | 1.03E+04 | 3.63E+03 |
|  |  | L-Leucyl-L-Leucine | 2.44E+04 | 5.23E+03 | 2.42E+04 | 2.05E+03 | 1.64E+04 | 6.89E+03 |
|  |  | L-Saccharopine | 1.73E+04 | 3.02E+03 | 2.10E+04 | 1.30E+04 | 6.78E+04 | 3.24E+04 |
|  |  | Tyr-Phe-Asp-Arg | 1.02E+04 | 1.11E+04 | 1.57E+04 | 4.25E+03 | 9.47E+03 | 5.36E+03 |
|  |  | Trp-Gln-His | 1.28E+04 | 7.24E+02 | 6.24E+03 | 1.12E+03 | 6.43E+03 | 9.82E+02 |
|  |  | His-Gln-Asn-Glu | 6.64E+04 | 2.35E+04 | 6.19E+04 | 3.92E+03 | 3.59E+04 | 4.73E+03 |
|  |  | Glu-Val-Phe-Asp-Glu | 1.12E+05 | 4.15E+04 | 9.46E+04 | 6.07E+04 | 1.48E+05 | 1.15E+05 |
|  |  | Tyr-Phe-Thr-Lys | 1.66E+05 | 2.05E+04 | 1.31E+05 | 1.91E+04 | 1.23E+05 | 6.78E+04 |
|  |  | Met-Cys-Asp-Phe-Thr | 1.12E+04 | 6.47E+02 | 8.73E+03 | 1.72E+03 | 2.36E+03 | 6.08E+02 |
|  |  | L-Homocitrulline | 3.64E+04 | 2.47E+04 | 4.21E+04 | 1.59E+04 | 4.05E+04 | 1.04E+04 |
| **Class I** | **Class II** | **Metabolite** | **DW** | | **YZ** | | **B21** | |
|  |  |  | **Average** | **SD** | **Average** | **SD** | **Average** | **SD** |
| **mino acids and derivatives** | **mino acids and derivatives** | Glu-Asp-Thr-Glu | 2.06E+05 | 4.46E+04 | 1.16E+05 | 2.55E+04 | 1.51E+05 | 4.90E+04 |
|  |  | N-Methyl-Trans-4-Hydroxy-L-Proline | 2.69E+05 | 6.05E+03 | 2.51E+05 | 2.06E+04 | 2.19E+05 | 8.59E+04 |
|  |  | L-Glutaminyl-L-valyl-L-valyl-L-cysteine | 4.19E+04 | 3.86E+03 | 3.12E+04 | 3.15E+03 | 1.97E+04 | 9.92E+03 |
|  |  | Ile-Arg-Tyr-Arg | 6.34E+03 | 1.17E+03 | 6.59E+03 | 9.62E+02 | 5.48E+03 | 2.65E+03 |
|  |  | L-Canavanine sulfate | 8.80E+04 | 1.31E+04 | 8.02E+04 | 1.13E+04 | 4.52E+04 | 1.29E+04 |
|  |  | Gln-Ala-Ala-Asp-Val | 9.06E+04 | 6.85E+04 | 4.33E+04 | 1.64E+04 | 5.49E+04 | 4.16E+04 |
|  |  | γ-Glutamyl-L-valine | 1.15E+04 | 1.79E+03 | 9.39E+03 | 8.61E+02 | 2.78E+04 | 1.10E+04 |
|  |  | 2-((L-tyrosyl)oxy)-5-amino-5-oxopentanoic acid | 1.29E+04 | 3.12E+03 | 1.93E+04 | 2.27E+03 | 3.67E+04 | 1.87E+04 |
|  |  | Trimethyllysine | 6.15E+04 | 4.52E+04 | 1.25E+05 | 2.62E+04 | 4.18E+05 | 2.72E+05 |
|  |  | L-γ-Glutamyl-L-leucine | 1.91E+04 | 2.17E+03 | 1.61E+04 | 2.65E+03 | 9.64E+03 | 6.99E+03 |
|  |  | Asp-Nap-OH | 3.22E+04 | 9.69E+03 | 3.12E+04 | 1.29E+04 | 9.02E+03 | 4.43E+03 |
|  |  | S-(5'-Adenosyl)-L-methionine | 3.17E+05 | 1.15E+04 | 2.70E+05 | 4.74E+04 | 5.61E+05 | 1.87E+05 |
|  |  | Ser-Asp-Ser-Gly-Val | 6.68E+04 | 1.56E+04 | 5.70E+04 | 3.23E+03 | 1.45E+04 | 6.61E+03 |
|  |  | Pro-Asn | 6.49E+04 | 1.04E+04 | 6.27E+04 | 5.62E+03 | 5.43E+04 | 1.57E+04 |
|  |  | Asp-Glu | 1.39E+04 | 1.48E+03 | 2.24E+04 | 1.01E+04 | 2.18E+04 | 6.69E+03 |
|  |  | L-Aspartic acid-O-diglucoside | 1.19E+05 | 3.87E+04 | 1.28E+05 | 1.72E+04 | 1.43E+05 | 3.68E+04 |
|  |  | H-Tyr-tyr-tyr-OH | 1.59E+04 | 7.83E+03 | 1.26E+04 | 2.56E+03 | 2.03E+03 | 1.01E+03 |
|  |  | Phe-Gln-Gln | 2.01E+04 | 7.30E+03 | 1.98E+04 | 6.60E+03 | 4.39E+03 | 4.68E+02 |
|  |  | Pro-Asp-Ala-Lys-Ser | 1.77E+05 | 7.83E+04 | 1.79E+05 | 5.33E+04 | 5.44E+04 | 1.06E+04 |
|  |  | L-Ornithine | 3.42E+04 | 1.41E+03 | 5.14E+04 | 2.14E+04 | 7.21E+04 | 3.94E+04 |
|  |  | Arg-Tyr-Leu-Lys | 1.54E+04 | 4.20E+03 | 2.28E+04 | 7.61E+03 | 1.14E+04 | 4.37E+03 |
|  |  | L-Serine | 2.04E+04 | 3.20E+03 | 2.53E+04 | 5.94E+03 | 1.40E+04 | 6.84E+03 |
|  |  | Phe-Asn-Lys | 5.20E+03 | 2.48E+03 | 8.14E+03 | 3.74E+03 | 9.27E+03 | 1.06E+04 |
|  |  | O-Acetyl-L-homoserine | 3.59E+04 | 9.76E+03 | 3.60E+04 | 9.14E+03 | 9.46E+04 | 2.74E+04 |
|  |  | Tyr-Tyr-Gln | 2.81E+04 | 1.61E+04 | 1.95E+04 | 6.46E+03 | 1.25E+04 | 2.90E+03 |
|  |  | Glu-Arg-Phe-Asp | 3.94E+03 | 1.05E+03 | 2.73E+03 | 1.33E+03 | 1.34E+04 | 1.52E+04 |
| **Class I** | **Class II** | **Metabolite** | **DW** | | **YZ** | | **B21** | |
|  |  |  | **Average** | **SD** | **Average** | **SD** | **Average** | **SD** |
| **mino acids and derivatives** | **mino acids and derivatives** | 3-nitro-L-tyrosine | 7.46E+03 | 3.78E+03 | 4.67E+03 | 1.71E+03 | 4.53E+03 | 9.15E+02 |
|  |  | His-Tyr-Glu-Asp | 1.40E+05 | 3.18E+04 | 9.05E+04 | 1.23E+04 | 4.58E+04 | 9.94E+03 |
|  |  | L-2-Aminoadipate | 8.21E+04 | 8.83E+03 | 7.45E+04 | 2.20E+04 | 1.98E+05 | 1.00E+05 |
|  |  | Asp-Asp-Glu | 6.04E+03 | 1.38E+03 | 6.83E+03 | 4.39E+03 | 7.00E+03 | 3.44E+03 |
|  |  | Thr-Thr | 3.20E+04 | 7.63E+03 | 4.98E+04 | 8.95E+03 | 2.72E+04 | 1.13E+04 |
|  |  | L-Lysine-Butanoic Acid | 3.50E+04 | 5.87E+03 | 1.91E+04 | 3.06E+03 | 1.43E+04 | 1.17E+04 |
|  |  | 4-Hydroxy-L-glutamic acid | 1.67E+05 | 3.06E+04 | 1.70E+05 | 2.85E+04 | 3.79E+05 | 1.05E+05 |
|  |  | TyrMe-His-OH | 3.70E+03 | 1.29E+03 | 3.68E+03 | 9.14E+02 | 9.00E+00 | 0.00E+00 |
|  |  | Tyr-Ser-His-Glu | 6.59E+05 | 1.34E+05 | 6.28E+05 | 1.24E+05 | 4.65E+05 | 2.01E+05 |
|  |  | Leu-Arg-Asp-Leu-Tyr | 8.67E+03 | 4.53E+03 | 4.42E+03 | 3.35E+03 | 3.00E+03 | 2.03E+03 |
|  |  | N-Methyl-α-aminoisobutyric acid | 4.07E+04 | 1.05E+03 | 2.89E+04 | 1.52E+04 | 1.88E+04 | 1.85E+04 |
|  |  | Lys-Ser-Asp-Glu-Met | 2.46E+04 | 4.93E+03 | 3.18E+04 | 1.45E+04 | 2.01E+04 | 1.13E+04 |
|  |  | Tyr-Lys | 3.81E+05 | 8.66E+04 | 3.20E+05 | 9.23E+04 | 3.14E+05 | 1.18E+04 |
|  |  | Allysine(6-Oxo DL-Norleucine) | 2.16E+04 | 4.36E+03 | 2.49E+04 | 1.33E+04 | 2.58E+04 | 4.84E+03 |
|  |  | Nicotinuric acid | 3.37E+03 | 1.19E+03 | 3.15E+03 | 2.64E+02 | 4.62E+03 | 5.11E+02 |
| **Organic acids** | **Organic acids** | 9-Oxononanoic acid | 2.17E+04 | 2.61E+03 | 2.48E+04 | 8.87E+01 | 2.28E+04 | 2.80E+03 |
|  |  | Piperonylic acid | 1.07E+06 | 2.79E+04 | 1.10E+06 | 5.78E+04 | 1.12E+06 | 7.39E+04 |
|  |  | 4-Hydroxy-2-Oxopentanoic Acid | 1.01E+05 | 2.22E+04 | 7.74E+04 | 5.11E+03 | 6.89E+04 | 3.08E+04 |
|  |  | 2-Hydroxyhexadecanoic acid | 3.86E+05 | 1.07E+04 | 3.47E+05 | 4.27E+04 | 3.76E+05 | 7.22E+04 |
|  |  | DL-3-Phenyllactic acid | 1.17E+05 | 2.42E+04 | 7.32E+04 | 2.67E+04 | 9.33E+04 | 4.96E+04 |
|  |  | Trans-Citridic acid | 5.85E+04 | 2.28E+04 | 9.19E+04 | 1.15E+04 | 6.05E+04 | 7.71E+03 |
|  |  | α-Ketoglutaric acid | 9.00E+05 | 2.14E+05 | 1.24E+06 | 3.74E+05 | 9.82E+05 | 2.49E+05 |
|  |  | Chelidonic acid | 3.11E+06 | 8.75E+05 | 2.15E+06 | 1.13E+05 | 5.82E+05 | 1.87E+05 |
|  |  | 4-Acetamidobutyric acid | 5.41E+05 | 2.03E+05 | 4.41E+05 | 8.44E+03 | 2.76E+05 | 4.50E+04 |
|  |  | 1-Hydroxy-2-Naphthoic Acid | 2.60E+04 | 1.06E+03 | 2.61E+04 | 2.72E+02 | 2.51E+04 | 1.28E+03 |
|  |  | Suberic Acid | 2.81E+05 | 1.88E+04 | 2.90E+05 | 2.84E+04 | 2.82E+05 | 2.87E+04 |
| **Class I** | **Class II** | **Metabolite** | **DW** | | **YZ** | | **B21** | |
|  |  |  | **Average** | **SD** | **Average** | **SD** | **Average** | **SD** |
| **Organic acids** | **Organic acids** | Aminomalonic acid | 7.16E+05 | 2.43E+04 | 8.00E+05 | 1.36E+05 | 7.78E+05 | 8.73E+04 |
|  |  | Azelaic acid | 4.97E+06 | 7.22E+04 | 4.99E+06 | 7.29E+05 | 5.02E+06 | 4.77E+05 |
|  |  | Sebacic acid | 5.22E+03 | 2.28E+02 | 5.10E+03 | 7.20E+02 | 5.06E+03 | 2.39E+02 |
|  |  | 3-(Beta-D-Glucopyranosyloxy)-5-Hydroxyhexanoic Acid Methyl Ester | 2.25E+04 | 9.23E+03 | 3.81E+04 | 1.73E+04 | 1.25E+04 | 6.12E+03 |
|  |  | 6-Aminocaproic acid | 3.15E+05 | 8.05E+04 | 3.23E+05 | 3.34E+04 | 9.47E+05 | 7.99E+05 |
|  |  | Mandelic acid | 9.73E+04 | 6.81E+03 | 8.35E+04 | 1.69E+04 | 1.62E+05 | 6.29E+04 |
|  |  | Isocitric Acid | 2.96E+06 | 7.65E+05 | 2.75E+06 | 1.10E+06 | 4.95E+06 | 1.61E+06 |
|  |  | 2-Methylglutaric acid | 4.25E+05 | 3.97E+04 | 4.29E+05 | 5.52E+04 | 2.94E+05 | 5.99E+04 |
|  |  | Pimelic acid* | 5.81E+04 | 5.15E+03 | 6.14E+04 | 9.24E+03 | 5.02E+04 | 1.78E+03 |
|  |  | Abscisic acid | 5.16E+05 | 7.22E+04 | 5.37E+05 | 7.21E+04 | 7.92E+05 | 2.26E+05 |
|  |  | Ethylmalonic acid | 2.56E+04 | 3.60E+03 | 2.55E+04 | 4.85E+03 | 1.72E+04 | 6.82E+03 |
|  |  | 2-Picolinic acid | 6.38E+05 | 5.29E+04 | 4.61E+05 | 1.69E+04 | 5.34E+05 | 4.24E+04 |
|  |  | 4,8-Dihydroxyquinoline-2-carboxylic acid | 7.46E+04 | 4.39E+04 | 4.12E+04 | 6.35E+02 | 8.82E+04 | 1.67E+04 |
|  |  | Succinic acid | 1.78E+07 | 6.92E+05 | 1.95E+07 | 3.96E+06 | 1.89E+07 | 3.37E+06 |
|  |  | Hydroxypyruvic acid | 2.82E+05 | 5.85E+04 | 3.28E+05 | 1.34E+05 | 5.31E+05 | 2.59E+05 |
|  |  | Shikimic acid | 2.21E+05 | 5.55E+04 | 2.51E+05 | 7.25E+03 | 8.15E+05 | 2.57E+05 |
|  |  | Citric Acid | 1.77E+06 | 5.67E+05 | 1.86E+06 | 8.50E+05 | 5.74E+06 | 3.38E+06 |
|  |  | 2,4-Dichlorophenoxyacetic Acid | 1.06E+05 | 9.77E+03 | 9.68E+04 | 2.14E+04 | 1.59E+05 | 5.99E+04 |
|  |  | 4,5,6-Trihydroxy-2-oxohexanoic acid | 2.23E+05 | 6.58E+04 | 1.62E+05 | 5.00E+03 | 8.28E+04 | 9.48E+03 |
|  |  | Methanesulfonic acid | 9.04E+05 | 7.80E+04 | 9.08E+05 | 5.01E+04 | 6.97E+05 | 9.33E+04 |
|  |  | Dimethylmalonic acid* | 4.70E+05 | 6.57E+05 | 6.48E+05 | 4.96E+05 | 4.55E+05 | 7.05E+05 |
|  |  | 2-Methylsuccinic acid* | 4.70E+05 | 6.57E+05 | 6.48E+05 | 4.96E+05 | 4.55E+05 | 7.05E+05 |
|  |  | Dehydro-L-(+)-ascorbic acid dimer | 5.47E+05 | 1.04E+05 | 5.35E+05 | 1.49E+05 | 7.41E+05 | 3.41E+05 |
|  |  | D-Mandelic acid | 3.75E+04 | 8.57E+03 | 3.09E+04 | 1.46E+03 | 6.11E+04 | 2.31E+04 |
|  |  | β-Hydroxyisovaleric acid | 2.13E+05 | 2.83E+04 | 2.23E+05 | 1.36E+04 | 2.89E+05 | 3.21E+04 |
|  |  | Phosphoenolpyruvate | 1.71E+06 | 3.27E+05 | 1.38E+06 | 3.32E+05 | 3.23E+06 | 7.57E+05 |
| **Class I** | **Class II** | **Metabolite** | **DW** | | **YZ** | | **B21** | |
|  |  |  | **Average** | **SD** | **Average** | **SD** | **Average** | **SD** |
| **Organic acids** | **Organic acids** | 5-Acetamidopentanoic Acid | 1.91E+05 | 4.00E+04 | 1.87E+05 | 1.64E+04 | 5.16E+05 | 4.55E+05 |
|  |  | Malonic acid | 2.00E+06 | 3.87E+05 | 2.29E+06 | 2.70E+05 | 3.24E+06 | 1.20E+06 |
|  |  | 2-Propylglutaric acid | 6.65E+05 | 1.12E+05 | 5.36E+05 | 7.21E+04 | 1.09E+06 | 3.39E+05 |
|  |  | Oxalic acid | 5.25E+04 | 6.04E+03 | 6.99E+04 | 5.15E+03 | 1.11E+05 | 5.59E+04 |
|  |  | 2-Oxoadipic acid | 1.10E+05 | 2.24E+04 | 9.17E+04 | 1.17E+04 | 8.74E+04 | 1.13E+04 |
|  |  | Glucosyl 2-Hydroxy-4-Methylpentanoic Acid | 1.24E+06 | 2.30E+05 | 1.06E+06 | 7.27E+04 | 1.10E+06 | 3.39E+05 |
|  |  | Spermidic acid | 9.31E+05 | 2.12E+05 | 1.61E+06 | 9.03E+04 | 7.87E+05 | 2.47E+05 |
|  |  | 4-Guanidinobutyric acid | 5.02E+05 | 5.27E+04 | 6.92E+05 | 2.07E+05 | 3.44E+06 | 2.11E+06 |
|  |  | Homovanillic acid sulfate | 1.21E+05 | 1.74E+04 | 1.11E+05 | 1.79E+04 | 1.27E+05 | 4.57E+04 |
|  |  | 2-Propylsuccinic acid* | 4.92E+04 | 1.59E+04 | 6.25E+04 | 1.15E+04 | 5.09E+04 | 3.07E+03 |
|  |  | Citric acid-1-O-diglucoside | 2.13E+04 | 2.00E+03 | 2.11E+04 | 7.14E+03 | 6.60E+04 | 3.11E+04 |
|  |  | 2-Hydroxy-2-methyl-3-oxobutanoic acid | 1.07E+05 | 1.73E+04 | 8.72E+04 | 5.16E+03 | 7.56E+04 | 3.93E+04 |
|  |  | L-Malic acid | 7.18E+05 | 1.00E+05 | 7.60E+05 | 3.01E+05 | 1.13E+06 | 8.02E+05 |
|  |  | Tuberonic acid glucoside | 1.37E+05 | 2.69E+04 | 1.50E+05 | 3.51E+04 | 5.24E+04 | 2.61E+04 |
|  |  | 2,2-Dimethylsuccinic acid | 5.10E+04 | 6.43E+03 | 6.67E+04 | 2.81E+04 | 1.18E+05 | 8.50E+04 |
|  |  | 3-Hydroxybutyric acid | 1.50E+06 | 2.28E+05 | 1.53E+06 | 3.55E+05 | 2.24E+06 | 7.56E+05 |
|  |  | 2-Propylmalic Acid | 3.42E+04 | 1.51E+04 | 2.37E+04 | 5.75E+03 | 1.00E+05 | 4.76E+04 |
|  |  | 2,6-Diaminooimelic acid | 6.41E+04 | 1.01E+04 | 8.36E+04 | 2.41E+04 | 5.26E+04 | 2.84E+04 |
|  |  | 2-Hydroxyglutaric Acid* | 1.22E+05 | 3.47E+04 | 1.43E+05 | 4.18E+04 | 4.37E+05 | 2.31E+05 |
|  |  | Adenylocuccinic Acid | 2.86E+05 | 1.40E+05 | 2.77E+05 | 3.02E+02 | 5.10E+05 | 7.88E+04 |
|  |  | Muconic acid | 2.42E+06 | 3.03E+05 | 3.13E+06 | 1.90E+05 | 2.10E+06 | 1.19E+06 |
|  |  | L-Tartaric acid | 7.66E+05 | 4.41E+04 | 6.64E+05 | 1.27E+05 | 1.37E+06 | 3.33E+05 |
|  |  | 2-Aminoisobutyric acid* | 4.37E+06 | 2.14E+05 | 4.16E+06 | 5.43E+05 | 2.20E+06 | 5.74E+05 |
|  |  | Mandelic acid-β-glucoside | 1.92E+05 | 1.50E+05 | 1.16E+05 | 1.70E+04 | 7.27E+04 | 1.87E+04 |
|  |  | L-Citramalic acid | 1.38E+05 | 4.32E+04 | 1.51E+05 | 6.71E+04 | 3.89E+05 | 2.22E+05 |
|  |  | L-Pipecolic Acid | 2.05E+06 | 3.30E+05 | 2.48E+06 | 4.70E+05 | 6.65E+06 | 2.05E+06 |
| **Class I** | **Class II** | **Metabolite** | **DW** | | **YZ** | | **B21** | |
|  |  |  | **Average** | **SD** | **Average** | **SD** | **Average** | **SD** |
| **Organic acids** | **Organic acids** | 2-Aminoheptanedioic acid | 5.61E+05 | 1.20E+05 | 7.32E+05 | 2.16E+04 | 4.54E+05 | 1.44E+05 |
|  |  | 2-Hydroxy-4-methylpentanoic acid | 6.42E+04 | 1.94E+04 | 4.33E+04 | 1.42E+04 | 8.74E+04 | 3.65E+04 |
|  |  | Fumaric acid | 2.07E+06 | 1.41E+05 | 2.25E+06 | 5.10E+05 | 3.50E+06 | 2.33E+06 |
|  |  | Quinic Acid | 1.41E+07 | 3.86E+06 | 1.49E+07 | 6.51E+06 | 2.01E+07 | 5.28E+06 |
|  |  | 2-Hydroxyisocaproic acid | 1.05E+05 | 1.07E+04 | 1.00E+05 | 1.01E+04 | 1.42E+05 | 3.08E+04 |
|  |  | cis-Citral | 6.86E+04 | 2.27E+03 | 6.43E+04 | 1.19E+04 | 7.76E+04 | 1.46E+04 |
|  |  | Creatine | 1.96E+05 | 4.51E+04 | 1.69E+05 | 2.61E+04 | 2.52E+05 | 1.13E+05 |
|  |  | 3-Oxooctadecanoic acid | 2.90E+04 | 4.36E+03 | 6.88E+04 | 1.12E+04 | 5.17E+04 | 2.06E+04 |
|  |  | 4-O-beta-D-glucosyl-4-coumaric acid | 6.85E+04 | 4.74E+04 | 6.02E+04 | 1.55E+04 | 1.06E+05 | 6.51E+04 |
|  |  | 3-Hydroxy-3-Methyl-2-Oxopentanoic Acid | 4.28E+04 | 4.47E+03 | 4.34E+04 | 5.15E+03 | 2.94E+04 | 5.30E+03 |
|  |  | Citric acid glucoside | 4.14E+04 | 6.51E+03 | 4.55E+04 | 2.04E+04 | 7.38E+04 | 1.73E+04 |
|  |  | 2-Amino-5-oxohexanoic acid | 2.00E+06 | 1.25E+06 | 1.67E+06 | 2.89E+05 | 1.36E+06 | 4.48E+05 |
|  |  | Citraconic acid | 6.33E+05 | 8.35E+04 | 5.06E+05 | 2.00E+05 | 1.43E+06 | 8.38E+05 |
|  |  | Tuberonic Acid | 3.90E+03 | 5.79E+02 | 3.67E+03 | 7.32E+02 | 3.08E+03 | 6.76E+02 |
|  |  | DL-Glyceric Acid | 2.61E+05 | 5.51E+04 | 2.77E+05 | 2.20E+04 | 3.27E+05 | 5.20E+04 |
|  |  | 2-Isopropylmalic Acid | 3.19E+04 | 1.03E+04 | 2.16E+04 | 5.74E+03 | 1.04E+05 | 5.70E+04 |
|  |  | Mevalonic acid | 4.98E+04 | 1.37E+04 | 4.02E+04 | 1.05E+04 | 6.98E+04 | 1.80E+04 |
|  |  | Creatinine | 1.84E+05 | 1.99E+04 | 1.63E+05 | 3.48E+04 | 1.36E+05 | 2.26E+04 |
|  |  | Dormin | 3.01E+05 | 5.38E+04 | 2.26E+05 | 4.77E+04 | 1.73E+05 | 3.26E+04 |
|  |  | 3-Ureidopropionic Acid | 6.22E+04 | 2.34E+04 | 5.73E+04 | 8.66E+03 | 1.11E+05 | 4.68E+04 |
|  |  | γ-Aminobutyric acid | 1.61E+05 | 1.29E+04 | 2.16E+05 | 6.29E+04 | 2.34E+05 | 7.01E+04 |
|  |  | 1-Aminocyclopropane-1-carboxylic acid | 8.02E+04 | 2.40E+04 | 9.35E+04 | 3.39E+04 | 1.55E+05 | 5.82E+04 |
|  |  | DL-3,4-Dihydroxymandelic acid | 7.11E+04 | 1.33E+04 | 1.04E+05 | 8.65E+03 | 6.26E+04 | 1.69E+04 |
|  |  | 3-Hydroxyglutaric acid* | 2.11E+05 | 2.33E+04 | 2.15E+05 | 5.44E+04 | 2.81E+05 | 8.67E+04 |
|  |  | Phytic acid | 6.38E+04 | 2.10E+03 | 6.60E+04 | 6.00E+03 | 6.40E+04 | 2.87E+03 |
|  |  | 2-Aminoethanesulfonic acid | 1.66E+05 | 8.60E+04 | 2.03E+05 | 1.20E+05 | 2.02E+05 | 2.75E+04 |
| **Class I** | **Class II** | **Metabolite** | **DW** | | **YZ** | | **B21** | |
|  |  |  | **Average** | **SD** | **Average** | **SD** | **Average** | **SD** |
| **Organic acids** | **Organic acids** | Malic acid-1-O-diglucoside | 2.44E+04 | 3.67E+03 | 3.14E+04 | 5.55E+03 | 5.30E+04 | 3.34E+04 |
|  |  | Argininosuccinic acid | 9.47E+04 | 3.53E+04 | 1.27E+05 | 2.49E+04 | 4.40E+05 | 1.90E+05 |
|  |  | d-Myo-inositol-1,4,5-triphosphate | 4.64E+04 | 1.70E+04 | 3.13E+04 | 1.70E+04 | 8.22E+04 | 7.50E+04 |
|  |  | 2-Methyl-3-oxosuccinic acid | 4.06E+04 | 1.03E+04 | 6.61E+04 | 2.59E+04 | 6.98E+04 | 2.49E+04 |
|  |  | Isocitric acid-1-O-diglucoside | 4.20E+04 | 2.34E+04 | 3.00E+04 | 7.56E+03 | 9.25E+04 | 5.13E+04 |
| **Lipids** | **Lipids** | 13S-Hydroperoxy-6Z,9Z,11E-octadecatrienoic acid | 6.80E+04 | 9.44E+03 | 6.89E+04 | 4.63E+03 | 5.72E+04 | 1.18E+04 |
|  |  | 2-Aminooctadecane-1,4-diol | 1.23E+05 | 4.91E+03 | 1.13E+05 | 7.33E+03 | 1.03E+05 | 8.49E+03 |
|  |  | 12-Hydroxyoctadecanoic acid | 5.55E+04 | 5.03E+03 | 6.46E+04 | 7.18E+03 | 5.67E+04 | 1.78E+03 |
|  |  | 1-Eicosanol | 3.25E+05 | 2.29E+04 | 3.48E+05 | 1.34E+05 | 4.11E+05 | 1.43E+05 |
|  |  | Tridecanedioic acid | 1.25E+06 | 1.58E+04 | 1.24E+06 | 3.82E+04 | 1.26E+06 | 2.32E+04 |
|  |  | 2-AminoicoSane-1,5,19-triol | 4.93E+04 | 2.77E+03 | 4.62E+04 | 1.54E+03 | 4.46E+04 | 4.61E+03 |
|  |  | 12,13-DHOME; (9Z)-12,13-Dihydroxyoctadec-9-enoic acid | 1.07E+05 | 5.15E+03 | 1.08E+05 | 2.40E+03 | 1.05E+05 | 5.03E+03 |
|  |  | 2-Amino-7-methyltridecan-1-ol | 3.38E+04 | 6.32E+03 | 3.31E+04 | 1.41E+03 | 2.85E+04 | 2.83E+03 |
|  |  | 2-(2,3-dihydroxypropoxy)-3-(((2-(dimethylamino)ethoxy)(hydroxy)phosphoryl)oxy)propyl (11Z,14Z)-octadeca-11,14-dienoate | 2.47E+06 | 7.05E+05 | 1.76E+06 | 1.17E+05 | 9.95E+05 | 8.31E+05 |
|  |  | 9-Decenoic acid | 1.24E+05 | 7.81E+04 | 9.47E+04 | 6.04E+04 | 1.30E+05 | 7.39E+04 |
|  |  | 9,12,13-Trihydroxy-10,15-octadecadienoic acid | 1.66E+05 | 1.01E+04 | 1.76E+05 | 3.77E+04 | 2.51E+05 | 3.65E+04 |
|  |  | LysoPE 16:0(2n isomer) | 9.67E+05 | 7.12E+04 | 7.80E+05 | 2.13E+05 | 1.30E+06 | 4.67E+05 |
|  |  | 9,10,11-Trihydroxy-12-octadecenoic acid | 1.57E+04 | 3.36E+03 | 1.84E+04 | 3.05E+03 | 5.37E+04 | 5.55E+03 |
|  |  | 12-Hydroxydodecanoic acid | 2.13E+04 | 1.16E+03 | 2.08E+04 | 2.17E+03 | 2.14E+04 | 5.90E+02 |
|  |  | 1,14-Tetradecanedioic Acid | 2.22E+05 | 1.05E+04 | 2.15E+05 | 3.62E+03 | 2.11E+05 | 1.20E+03 |
|  |  | LysoPC 18:3(2n isomer) | 1.62E+05 | 1.91E+04 | 1.23E+05 | 4.23E+04 | 2.96E+05 | 5.59E+04 |
|  |  | 1-Oleoyl-Sn-Glycerol | 3.67E+05 | 1.53E+05 | 4.89E+05 | 1.38E+05 | 1.70E+05 | 9.73E+04 |
|  |  | LysoPE 16:0 | 9.01E+05 | 1.02E+05 | 7.13E+05 | 1.90E+05 | 1.20E+06 | 5.08E+05 |
|  |  | LysoPC 16:1(2n isomer) | 1.18E+05 | 1.79E+04 | 7.12E+04 | 1.74E+04 | 1.32E+05 | 1.28E+05 |
|  |  | Palmitaldehyde | 1.43E+07 | 5.19E+05 | 1.41E+07 | 3.53E+05 | 1.80E+07 | 1.64E+06 |
| **Class I** | **Class II** | **Metabolite** | **DW** | | **YZ** | | **B21** | |
|  |  |  | **Average** | **SD** | **Average** | **SD** | **Average** | **SD** |
| **Lipids** | **Lipids** | 1-(2,3-dihydroxypropoxy)-3-(((2-(dimethylamino)ethoxy)(hydroxy)phosphoryl)oxy)propan-2-yl palmitate | 1.04E+06 | 1.86E+05 | 7.94E+05 | 1.46E+05 | 1.09E+06 | 6.49E+05 |
|  |  | 13(S)-HODE;13(S)-Hydroxyoctadeca-9Z,11E-dienoic acid* | 6.51E+04 | 8.74E+03 | 7.54E+04 | 1.41E+04 | 1.37E+05 | 4.72E+04 |
|  |  | Hydroxy ricinoleic acid | 1.65E+04 | 6.93E+03 | 1.26E+04 | 4.93E+02 | 2.79E+04 | 6.57E+03 |
|  |  | 1-O-Octadecyl-2-O-methyl-sn-glycero-3-phosphocholine | 4.92E+05 | 1.40E+04 | 4.79E+05 | 3.37E+04 | 3.62E+05 | 1.16E+05 |
|  |  | LysoPE 18:1 | 1.67E+06 | 2.37E+05 | 2.11E+06 | 3.39E+05 | 2.58E+06 | 2.86E+05 |
|  |  | 2-[(1R,2R)-3-oxo-2-[(Z)-5-[3,4,5-trihydroxy-6-(hydroxymethyl)oxan-2-yl]oxypent-2-enyl]cyclopentyl]acetic acid | 6.58E+05 | 1.35E+05 | 4.15E+05 | 4.52E+04 | 7.06E+05 | 4.20E+05 |
|  |  | 10-Hydroxydecanoic acid | 1.82E+04 | 5.85E+02 | 1.97E+04 | 1.47E+03 | 2.05E+04 | 2.42E+03 |
|  |  | 2-aminodocoSane-1,6,19,20,21-pentaol | 2.86E+05 | 2.59E+04 | 2.75E+05 | 2.11E+04 | 2.36E+05 | 2.12E+04 |
|  |  | Ricinoleic acid | 3.40E+05 | 2.89E+04 | 3.62E+05 | 1.26E+05 | 4.41E+05 | 1.57E+05 |
|  |  | 2-(2,3-dihydroxypropoxy)-3-(((2-(dimethylamino)ethoxy)(hydroxy)phosphoryl)oxy)propyl palmitate | 1.88E+05 | 1.93E+04 | 1.51E+05 | 3.59E+04 | 2.75E+05 | 1.52E+05 |
|  |  | LysoPC 18:1(2n isomer) | 1.51E+07 | 2.09E+06 | 1.53E+07 | 3.63E+05 | 1.07E+07 | 3.66E+06 |
|  |  | Stearic Acid | 3.97E+07 | 2.21E+06 | 5.05E+07 | 4.49E+06 | 5.42E+07 | 2.21E+06 |
|  |  | 2-AminoicoSane-1,5,7,19-tetraol | 1.54E+06 | 3.00E+04 | 1.50E+06 | 9.60E+04 | 1.35E+06 | 1.15E+05 |
|  |  | 1-(2,3-dihydroxypropoxy)-3-(((2-(dimethylamino)ethoxy)(hydroxy)phosphoryl)oxy)propan-2-yl (E)-hexadec-9-enoate | 5.37E+04 | 7.77E+03 | 3.71E+04 | 9.54E+03 | 2.33E+04 | 6.21E+03 |
|  |  | 5,6-DiHETrE[(±)5,6-dihydroxy-8Z,11Z,14Z-eicosatrienoic acid] | 2.70E+04 | 8.67E+03 | 3.97E+04 | 6.90E+03 | 1.54E+04 | 7.55E+03 |
|  |  | LysoPC 18:0 | 2.15E+05 | 2.97E+04 | 2.25E+05 | 6.00E+04 | 5.13E+05 | 1.75E+05 |
|  |  | Undecanedioic acid | 2.88E+05 | 1.52E+04 | 2.98E+05 | 1.53E+04 | 3.09E+05 | 4.92E+03 |
|  |  | 2R-hydroxy-9Z,12Z,15Z-octadecatrienoic acid | 6.54E+03 | 1.87E+03 | 1.09E+04 | 4.29E+03 | 1.53E+04 | 8.79E+03 |
|  |  | 3-Hydroxyoctadecanoic Acid | 2.84E+06 | 7.11E+04 | 2.86E+06 | 7.12E+04 | 2.81E+06 | 1.60E+05 |
|  |  | 3-Hydroxy-palmitic acid methyl ester | 2.58E+04 | 3.82E+03 | 3.43E+04 | 7.86E+03 | 2.65E+04 | 7.54E+02 |
|  |  | Undecylic Acid | 1.46E+06 | 3.53E+04 | 1.43E+06 | 3.76E+04 | 1.44E+06 | 2.55E+04 |
|  |  | 13S-Hydroperoxy-9Z,11E-octadecadienoic acid | 8.52E+03 | 2.36E+03 | 8.52E+03 | 1.48E+03 | 2.85E+04 | 1.50E+04 |
|  |  | Palmitic acid | 5.21E+04 | 1.76E+03 | 5.72E+04 | 5.48E+02 | 4.98E+04 | 1.25E+03 |
|  |  | 9,10,13-Trihydroxy-11-Octadecenoic Acid | 2.27E+05 | 6.25E+04 | 2.61E+05 | 6.13E+04 | 1.11E+06 | 1.31E+05 |
|  |  | 9S-Hydroxy-10E,12Z-octadecadienoic acid* | 6.19E+04 | 1.30E+04 | 6.40E+04 | 1.63E+04 | 1.21E+05 | 4.60E+04 |
| **Class I** | **Class II** | **Metabolite** | **DW** | | **YZ** | | **B21** | |
|  |  |  | **Average** | **SD** | **Average** | **SD** | **Average** | **SD** |
| **Lipids** | **Lipids** | Choline Alfoscerate | 9.47E+04 | 2.20E+04 | 9.79E+04 | 8.19E+03 | 4.18E+05 | 4.78E+05 |
|  |  | 9-Hydroperoxy-10E,12,15Z-octadecatrienoic acid | 3.74E+04 | 5.27E+03 | 4.28E+04 | 4.24E+03 | 3.44E+04 | 6.69E+03 |
|  |  | Cis-10-Pentadecenoic Acid(C15:1) | 3.99E+05 | 9.57E+03 | 4.00E+05 | 3.53E+04 | 4.13E+05 | 3.87E+04 |
|  |  | 1-Octadecanol | 6.33E+05 | 2.48E+04 | 6.30E+05 | 1.13E+04 | 6.16E+05 | 5.99E+03 |
|  |  | Hexadecanedioic acid | 2.07E+05 | 2.48E+03 | 2.18E+05 | 2.99E+03 | 2.16E+05 | 1.17E+04 |
|  |  | 9-Hydroxy-13-oxo-10-octadecenoic Acid | 3.35E+04 | 1.80E+04 | 3.46E+04 | 1.45E+04 | 3.79E+04 | 1.55E+04 |
|  |  | 1-(2,3-dihydroxypropoxy)-3-(((2-(dimethylamino)ethoxy)(hydroxy)phosphoryl)oxy)propan-2-yl (8E,11Z,14Z)-octadeca-8,11,14-trienoate | 6.10E+04 | 2.17E+04 | 4.74E+04 | 2.02E+04 | 9.25E+04 | 2.13E+04 |
|  |  | LysoPC 17:0 | 2.92E+04 | 6.90E+03 | 2.30E+04 | 1.87E+03 | 3.41E+04 | 1.56E+04 |
|  |  | Petroselinic acid* | 6.83E+06 | 9.29E+05 | 2.27E+07 | 1.26E+07 | 1.08E+07 | 1.15E+06 |
|  |  | alpha-Hydroxylinoleic acid* | 5.65E+04 | 5.23E+03 | 6.49E+04 | 1.54E+04 | 1.24E+05 | 4.60E+04 |
|  |  | LysoPC 16:1 | 1.08E+05 | 3.00E+04 | 8.37E+04 | 1.99E+04 | 5.33E+04 | 2.38E+04 |
|  |  | Gingerglycolipid A | 8.85E+04 | 2.04E+04 | 7.97E+04 | 2.98E+04 | 7.10E+04 | 2.87E+04 |
|  |  | LysoPC 18:0(2n isomer) | 2.24E+05 | 2.47E+04 | 2.44E+05 | 5.77E+04 | 5.41E+05 | 1.85E+05 |
|  |  | 11-Octadecanoic acid(Vaccenic acid)* | 5.19E+06 | 8.38E+05 | 1.97E+07 | 1.11E+07 | 9.38E+06 | 1.70E+06 |
|  |  | Pinolenic acid | 4.42E+06 | 1.70E+05 | 5.39E+06 | 2.85E+05 | 5.07E+06 | 7.87E+04 |
|  |  | 13(s)-hydroperoxy-(9z,11e,15z)-octadecatrienoic acid | 3.08E+06 | 2.21E+05 | 3.44E+06 | 1.39E+05 | 3.35E+06 | 3.06E+05 |
|  |  | LysoPC 18:2 | 1.27E+07 | 2.77E+06 | 9.12E+06 | 1.88E+06 | 7.61E+06 | 6.65E+06 |
|  |  | Dodecanoic acid (Lauric acid) | 1.82E+05 | 2.36E+04 | 1.78E+05 | 9.95E+03 | 2.02E+05 | 1.59E+04 |
|  |  | LysoPC 18:2(2n isomer) | 1.15E+07 | 2.75E+06 | 7.17E+06 | 1.21E+06 | 3.76E+06 | 3.36E+06 |
|  |  | 1-(9Z,12Z-octadecadienoyl)-sn-glycero-3-phosphocholine | 1.15E+07 | 2.75E+06 | 7.17E+06 | 1.21E+06 | 3.76E+06 | 3.36E+06 |
|  |  | 7S,8S-DiHODE; (9Z,12Z)-(7S,8S)-Dihydroxyoctadeca-9,12-dienoic acid* | 1.51E+04 | 2.07E+03 | 1.69E+04 | 2.99E+03 | 1.44E+04 | 1.90E+03 |
|  |  | 2-Aminotetradecane-1,4-diol | 6.70E+04 | 3.53E+03 | 5.84E+04 | 6.38E+03 | 4.43E+04 | 1.35E+03 |
|  |  | LysoPC 18:1 | 2.09E+07 | 2.32E+06 | 1.91E+07 | 2.56E+06 | 2.10E+07 | 6.25E+06 |
|  |  | Sn-glycerol-1-phosphate | 4.35E+05 | 7.29E+04 | 3.88E+05 | 6.39E+03 | 2.79E+05 | 1.61E+05 |
|  |  | 2-Palmitoyl-Sn-Glycerol 3-O-Diglucoside | 5.60E+04 | 1.12E+04 | 6.16E+04 | 1.13E+04 | 3.81E+04 | 1.69E+04 |
| **Class I** | **Class II** | **Metabolite** | **DW** | | **YZ** | | **B21** | |
|  |  |  | **Average** | **SD** | **Average** | **SD** | **Average** | **SD** |
| **Lipids** | **Lipids** | 8,11-eicosadiynoic acid | 7.57E+04 | 3.00E+03 | 7.95E+04 | 2.69E+03 | 7.95E+04 | 2.38E+03 |
|  |  | Gingerglycolipid B | 7.69E+05 | 2.22E+05 | 6.90E+05 | 2.70E+05 | 5.54E+05 | 4.72E+05 |
|  |  | 2R-Hydroxyoctadecanoic Acid* | 3.42E+04 | 6.00E+03 | 3.74E+04 | 6.90E+03 | 4.05E+04 | 9.36E+03 |
|  |  | 1-Pentadecanoyl-sn-glycero-3-phosphocholine | 1.05E+04 | 1.76E+03 | 9.85E+03 | 2.33E+03 | 1.28E+04 | 1.17E+04 |
|  |  | LysoPC 17:1 | 5.06E+04 | 1.54E+04 | 4.35E+04 | 3.71E+03 | 4.82E+04 | 1.85E+04 |
|  |  | LysoPC 15:1 | 1.00E+04 | 1.15E+03 | 8.47E+03 | 2.88E+03 | 8.02E+03 | 7.48E+03 |
|  |  | LysoPE 15:0(2n isomer) | 6.19E+03 | 1.25E+03 | 4.21E+03 | 1.53E+03 | 4.51E+03 | 3.95E+03 |
|  |  | 1-(2,3-dihydroxypropoxy)-3-(((2-(dimethylamino)ethoxy)(hydroxy)phosphoryl)oxy)propan-2-yl (Z)-14-Octadecenoic Acid | 2.01E+06 | 9.37E+04 | 2.17E+06 | 1.40E+05 | 1.83E+06 | 5.79E+05 |
|  |  | LysoPE 18:1(2n isomer) | 1.43E+06 | 1.60E+05 | 1.76E+06 | 4.45E+05 | 1.47E+06 | 3.83E+05 |
|  |  | LysoPE 18:0(2n isomer) | 1.35E+04 | 2.34E+03 | 1.68E+04 | 6.44E+03 | 2.14E+04 | 2.15E+03 |
|  |  | LysoPE 15:0 | 5.22E+03 | 1.37E+03 | 3.99E+03 | 9.67E+02 | 5.66E+03 | 5.44E+03 |
|  |  | LysoPC 16:0(2n isomer) | 4.95E+06 | 8.38E+05 | 3.78E+06 | 1.11E+06 | 4.64E+06 | 3.24E+06 |
|  |  | 1-Palmitoyl-Sn-Glycerol 3-O-Diglucoside | 8.10E+03 | 2.89E+03 | 9.19E+03 | 1.06E+03 | 6.52E+03 | 3.34E+03 |
|  |  | 1-(2,3-dihydroxypropoxy)-3-(((2-(dimethylamino)ethoxy)(hydroxy)phosphoryl)oxy)propan-2-yl (11Z,14Z)-octadeca-11,14-dienoate | 2.28E+06 | 7.22E+05 | 1.64E+06 | 2.84E+05 | 9.04E+05 | 7.66E+05 |
|  |  | LysoPC 16:0 | 4.88E+06 | 1.05E+06 | 3.28E+06 | 8.06E+05 | 4.38E+06 | 2.80E+06 |
|  |  | 13-Hydroperoxy-9Z,11E-octadecadienoic acid* | 1.25E+04 | 4.12E+02 | 1.23E+04 | 1.55E+03 | 1.29E+04 | 1.22E+03 |
|  |  | LysoPE 18:2 | 2.74E+06 | 1.36E+05 | 2.23E+06 | 5.54E+05 | 2.89E+06 | 1.41E+06 |
|  |  | α-Linolenic Acid* | 9.15E+05 | 1.58E+05 | 9.70E+05 | 1.24E+05 | 1.12E+06 | 3.68E+05 |
|  |  | 9-Hydroxy-12-oxo-10(E),15(Z)-octadecadienoic acid | 9.58E+03 | 2.54E+03 | 1.30E+04 | 2.56E+03 | 1.11E+04 | 1.64E+03 |
|  |  | LysoPE 18:3(2n isomer) | 3.68E+04 | 7.00E+03 | 2.78E+04 | 8.47E+03 | 5.33E+04 | 1.38E+04 |
|  |  | LysoPC 14:0 | 4.46E+04 | 9.53E+03 | 2.96E+04 | 8.59E+03 | 5.16E+04 | 4.72E+04 |
|  |  | LysoPC 18:3 | 1.52E+05 | 2.22E+04 | 1.15E+05 | 3.35E+04 | 3.06E+05 | 7.32E+04 |
|  |  | Elaidic Acid* | 1.90E+07 | 2.43E+06 | 6.12E+07 | 2.95E+07 | 2.93E+07 | 5.92E+06 |
|  |  | Dodecanedioic aicd | 9.86E+03 | 7.15E+02 | 9.42E+03 | 3.92E+02 | 9.02E+03 | 8.28E+02 |
| **Class I** | **Class II** | **Metabolite** | **DW** | | **YZ** | | **B21** | |
|  |  |  | **Average** | **SD** | **Average** | **SD** | **Average** | **SD** |
| **Lipids** | **Lipids** | Punicic acid (9Z,11E,13Z-octadecatrienoic acid) | 2.22E+04 | 3.05E+03 | 2.10E+04 | 1.25E+04 | 3.20E+04 | 9.40E+03 |
|  |  | LysoPE 16:1 | 1.38E+04 | 3.19E+03 | 1.34E+04 | 2.99E+03 | 1.97E+04 | 1.59E+04 |
|  |  | 13S-Hydroxy-9Z,11E,15Z-octadecatrienoic acid | 6.80E+03 | 1.39E+03 | 6.97E+03 | 1.69E+03 | 2.47E+04 | 1.28E+04 |
|  |  | LysoPC 17:0(2n isomer) | 5.68E+03 | 1.13E+03 | 4.28E+03 | 1.05E+03 | 7.14E+03 | 4.94E+02 |
|  |  | 1-α-Linolenoyl-glycerol* | 2.12E+04 | 1.94E+03 | 2.21E+04 | 6.37E+03 | 2.58E+04 | 1.03E+04 |
|  |  | 2-(2,3-dihydroxypropoxy)-3-(((2-(dimethylamino)ethoxy)(hydroxy)phosphoryl)oxy)propyl (8E,11Z,14Z)-octadeca-8,11,14-trienoate | 5.36E+04 | 1.53E+04 | 4.35E+04 | 1.34E+04 | 8.40E+04 | 7.93E+03 |
|  |  | LysoPC(18:3(9Z,12Z,15Z)) | 5.36E+04 | 1.53E+04 | 4.35E+04 | 1.34E+04 | 8.40E+04 | 7.93E+03 |
|  |  | LysoPE 14:0 | 6.64E+03 | 8.01E+02 | 5.07E+03 | 1.73E+03 | 8.75E+03 | 6.15E+03 |
|  |  | 2-α-Linolenoyl-glycerol* | 2.30E+04 | 2.80E+03 | 1.64E+04 | 5.15E+03 | 2.88E+04 | 1.11E+04 |
|  |  | 9(10)-EpOME;(9R,10S)-(12Z)-9,10-Epoxyoctadecenoic acid | 1.08E+04 | 3.03E+03 | 1.38E+04 | 1.43E+03 | 1.37E+04 | 6.82E+03 |
|  |  | Gingerglycolipid C | 2.96E+05 | 1.31E+05 | 4.24E+05 | 2.53E+05 | 1.47E+05 | 8.88E+04 |
|  |  | Cis-4,7,10,13,16,19-Docosahexaenoic Acid | 2.08E+04 | 7.29E+02 | 1.95E+04 | 1.31E+03 | 1.92E+04 | 2.30E+03 |
|  |  | 2-Aminotetradecane-1,11,13-triol | 1.70E+04 | 1.68E+03 | 1.55E+04 | 6.25E+02 | 1.08E+04 | 1.16E+03 |
|  |  | 1-Monomyristin | 5.62E+03 | 4.72E+02 | 5.67E+03 | 1.54E+03 | 4.68E+03 | 5.24E+02 |
|  |  | 17-Hydroxylinolenic acid | 2.29E+04 | 3.68E+03 | 3.67E+04 | 9.28E+03 | 3.82E+04 | 1.29E+04 |
|  |  | Pentadecanoic Acid* | 2.86E+05 | 1.33E+04 | 2.54E+05 | 2.54E+04 | 3.27E+05 | 6.43E+04 |
|  |  | 2-Aminohexadecane-1,4-diol | 2.47E+06 | 8.49E+04 | 2.11E+06 | 8.76E+04 | 1.64E+06 | 1.25E+05 |
|  |  | LysoPC 15:0 | 2.05E+04 | 3.68E+03 | 1.60E+04 | 4.75E+03 | 1.89E+04 | 1.76E+04 |
|  |  | LysoPC 15:0(2n isomer) | 2.05E+04 | 3.68E+03 | 1.60E+04 | 4.75E+03 | 1.89E+04 | 1.76E+04 |
|  |  | 2-Aminododecane-1,4-diol | 3.94E+04 | 1.78E+03 | 3.70E+04 | 5.26E+03 | 2.64E+04 | 4.86E+03 |
|  |  | Linoleic acid | 5.25E+04 | 1.30E+04 | 8.82E+04 | 2.11E+04 | 7.43E+04 | 3.19E+04 |
|  |  | (9Z,11E)-Octadecadienoic acid | 5.25E+04 | 1.30E+04 | 8.82E+04 | 2.11E+04 | 7.43E+04 | 3.19E+04 |
|  |  | γ-Linolenic Acid* | 1.19E+06 | 1.64E+05 | 1.24E+06 | 9.89E+04 | 1.44E+06 | 3.62E+05 |
|  |  | 2-Aminohexadecane-1,15-diol | 2.37E+06 | 1.23E+05 | 2.11E+06 | 1.09E+05 | 1.64E+06 | 1.30E+05 |
|  |  | Hexadecylsphingosine | 2.81E+06 | 1.63E+05 | 2.38E+06 | 1.51E+05 | 1.86E+06 | 1.80E+05 |
| **Class I** | **Class II** | **Metabolite** | **DW** | | **YZ** | | **B21** | |
|  |  |  | **Average** | **SD** | **Average** | **SD** | **Average** | **SD** |
| **Lipids** | **Lipids** | 1-(9Z-octadecenoyl)-sn-glycero-3-phosphocholine | 4.01E+06 | 5.44E+05 | 4.95E+06 | 6.75E+05 | 4.20E+06 | 1.35E+06 |
|  |  | 12,13-Epoxy-9-Octadecenoic Acid | 9.55E+03 | 2.60E+03 | 1.67E+04 | 2.46E+03 | 1.17E+04 | 5.19E+03 |
|  |  | 8,15-Dihydroxy-5,9,11,13-eicosatetraenoic acid | 1.35E+05 | 5.22E+03 | 1.29E+05 | 3.39E+03 | 1.03E+05 | 9.46E+03 |
|  |  | LysoPE 18:3 | 3.87E+04 | 2.89E+03 | 2.49E+04 | 7.68E+03 | 5.57E+04 | 1.02E+04 |
|  |  | 13-methylmyristic acid* | 2.40E+05 | 6.35E+04 | 2.06E+05 | 4.53E+04 | 2.56E+05 | 5.34E+04 |
|  |  | Octadeca-11E,13E,15Z-trienoic acid | 4.18E+07 | 3.24E+06 | 3.84E+07 | 2.57E+06 | 3.48E+07 | 3.41E+06 |
|  |  | DL-2-hydroxystearic acid* | 3.71E+04 | 5.46E+03 | 4.01E+04 | 5.08E+03 | 4.37E+04 | 1.28E+04 |
|  |  | 9(R)-Hete | 2.27E+05 | 2.40E+04 | 2.15E+05 | 5.52E+04 | 2.59E+05 | 1.07E+05 |
|  |  | LysoPE 18:2(2n isomer) | 2.44E+06 | 2.99E+05 | 1.74E+06 | 3.11E+05 | 1.44E+06 | 7.13E+05 |
|  |  | LysoPE 16:1(2n isomer) | 1.76E+04 | 3.55E+03 | 1.32E+04 | 6.94E+03 | 1.95E+04 | 1.11E+04 |
|  |  | 19,20-DiHDPA | 1.00E+06 | 3.42E+05 | 1.92E+06 | 5.23E+05 | 1.66E+06 | 6.49E+05 |
|  |  | 1-Linoleoylglycerol-2,3-di-O-glucoside* | 5.62E+03 | 1.73E+03 | 5.48E+03 | 2.11E+03 | 2.62E+03 | 2.32E+03 |
|  |  | LysoPE 17:1(2n isomer) | 7.50E+03 | 2.55E+03 | 8.00E+03 | 1.65E+03 | 9.42E+03 | 2.06E+03 |
|  |  | LysoPG 16:0 | 1.29E+05 | 2.21E+04 | 1.17E+05 | 2.14E+04 | 1.64E+05 | 2.20E+04 |
|  |  | Eicosenoic acid | 6.24E+06 | 5.96E+05 | 8.53E+06 | 1.04E+06 | 7.09E+06 | 9.97E+05 |
|  |  | 2-Linoleoylglycerol-1,3-di-O-glucoside* | 6.72E+03 | 2.10E+03 | 5.06E+03 | 4.15E+02 | 3.52E+03 | 3.20E+03 |
| **Nucleotides and derivatives** | **Nucleotides and derivatives** | 2-Aminopurine | 1.49E+06 | 3.37E+05 | 1.50E+06 | 1.82E+05 | 2.27E+06 | 5.33E+05 |
|  |  | Inosine 5'-monophosphate | 3.23E+05 | 2.82E+04 | 2.25E+05 | 6.15E+04 | 5.55E+05 | 1.81E+05 |
|  |  | Xanthosine | 1.14E+06 | 2.37E+05 | 1.02E+06 | 1.41E+05 | 2.45E+06 | 2.81E+06 |
|  |  | Guanosine 3',5'-cyclic monophosphate | 4.66E+04 | 1.28E+04 | 4.17E+04 | 1.78E+03 | 3.78E+04 | 1.36E+04 |
|  |  | 2'-Deoxycytidine-5'-monophosphate | 1.20E+04 | 2.68E+03 | 1.56E+04 | 4.03E+03 | 1.31E+04 | 1.17E+03 |
|  |  | Guanosine | 1.75E+06 | 2.70E+05 | 1.42E+06 | 1.39E+05 | 3.70E+06 | 2.46E+06 |
|  |  | Adenosine 5'-diphosphate | 3.00E+06 | 3.40E+05 | 3.00E+06 | 3.70E+05 | 2.66E+06 | 9.59E+05 |
|  |  | 4-methyl-1,5,2,3-dioxadiazinan-2-amine | 9.32E+05 | 8.74E+04 | 9.65E+05 | 8.23E+04 | 1.85E+06 | 1.42E+06 |
|  |  | 6-Chloropurine | 4.93E+05 | 3.63E+04 | 4.97E+05 | 4.58E+04 | 3.88E+05 | 7.94E+04 |
|  |  | 2'-Deoxyadenosine | 4.57E+04 | 2.63E+03 | 3.19E+04 | 8.32E+03 | 4.67E+04 | 1.30E+04 |
| **Class I** | **Class II** | **Metabolite** | **DW** | | **YZ** | | **B21** | |
|  |  |  | **Average** | **SD** | **Average** | **SD** | **Average** | **SD** |
| **Nucleotides and derivatives** | **Nucleotides and derivatives** | Succinyladenosine | 9.05E+05 | 1.66E+05 | 1.26E+06 | 2.91E+05 | 1.07E+06 | 5.17E+05 |
|  |  | Uridine | 2.23E+06 | 4.32E+05 | 2.47E+06 | 2.92E+05 | 3.58E+06 | 1.39E+06 |
|  |  | Uridine 5'-diphospho-N-acetylglucosamine | 3.23E+05 | 1.13E+05 | 3.89E+05 | 1.06E+05 | 6.90E+05 | 5.49E+04 |
|  |  | Vidarabine | 3.98E+07 | 1.03E+07 | 3.53E+07 | 2.68E+06 | 4.97E+07 | 1.85E+07 |
|  |  | (2r,3s,4r,5s)-2-(6-aminopurin-9-yl)-5-(hydroxymethyl)oxolane-3,4-diol* | 4.11E+07 | 7.80E+06 | 3.60E+07 | 3.87E+06 | 5.04E+07 | 1.98E+07 |
|  |  | 9-(Arabinosyl)hypoxanthine | 2.73E+05 | 1.74E+04 | 3.06E+05 | 8.34E+04 | 4.29E+05 | 1.23E+05 |
|  |  | Guanosine 5'-monophosphate | 3.72E+05 | 1.02E+05 | 2.49E+05 | 8.74E+04 | 4.31E+05 | 1.70E+05 |
|  |  | Adenosine 5'-monophosphate | 6.65E+06 | 1.44E+06 | 3.88E+06 | 1.07E+06 | 9.83E+06 | 4.35E+06 |
|  |  | Crotonoside; 2-Hydroxyadenosine | 2.70E+06 | 4.11E+05 | 2.22E+06 | 1.50E+05 | 5.98E+06 | 4.03E+06 |
|  |  | Isopentenyladenine-7-N-glucoside | 8.63E+05 | 6.33E+04 | 8.72E+05 | 1.24E+05 | 7.78E+05 | 2.85E+05 |
|  |  | Cyclic 3',5'-Adenylic acid | 6.07E+04 | 2.04E+04 | 6.18E+04 | 4.26E+03 | 8.92E+04 | 2.32E+04 |
|  |  | Barbituric acid | 1.98E+05 | 9.72E+04 | 1.85E+05 | 9.05E+04 | 2.41E+05 | 8.17E+04 |
|  |  | β-Pseudouridine | 2.66E+05 | 3.92E+04 | 2.81E+05 | 2.00E+04 | 3.72E+05 | 1.60E+05 |
|  |  | β-Nicotinamide mononucleotide | 1.10E+06 | 1.51E+05 | 9.53E+05 | 1.80E+05 | 7.03E+05 | 3.67E+05 |
|  |  | 6-Benzylaminopurine | 9.00E+00 | 0.00E+00 | 9.00E+00 | 0.00E+00 | 9.00E+00 | 0.00E+00 |
|  |  | Flavin Single Nucleotide(FMN) | 9.65E+04 | 1.47E+04 | 7.91E+04 | 1.12E+04 | 8.78E+04 | 2.37E+04 |
|  |  | Ribosyladenosine | 5.29E+04 | 1.10E+04 | 4.47E+04 | 8.61E+03 | 5.49E+04 | 2.22E+04 |
|  |  | Uridine-5'-Diphosphate-D-Xylose | 2.40E+06 | 7.39E+05 | 1.90E+06 | 5.00E+05 | 3.76E+06 | 1.36E+06 |
|  |  | Adenosine* | 4.38E+07 | 4.96E+06 | 3.88E+07 | 4.26E+06 | 5.45E+07 | 2.18E+07 |
|  |  | Uridine-5'-diphosphoglucuronic acid | 4.00E+05 | 1.03E+05 | 2.90E+05 | 9.39E+03 | 6.87E+05 | 3.58E+05 |
|  |  | Nicotinic acid adenine dinucleotide | 1.09E+06 | 1.87E+05 | 1.03E+06 | 1.94E+05 | 1.73E+06 | 3.75E+05 |
|  |  | Uridine 5'-diphosphate | 3.98E+05 | 7.42E+04 | 3.47E+05 | 7.33E+04 | 7.15E+05 | 2.53E+05 |
|  |  | NADP (Nicotinamide adenine dinucleotide phosphate) | 2.26E+05 | 1.03E+05 | 3.21E+05 | 5.15E+04 | 3.73E+05 | 6.60E+04 |
|  |  | Uridine-5'-diphosphogalactose disodium salt | 1.01E+07 | 1.12E+06 | 8.65E+06 | 1.05E+06 | 1.40E+07 | 1.06E+07 |
|  |  | Allopurinol | 2.45E+04 | 3.54E+03 | 2.26E+04 | 5.13E+03 | 4.34E+04 | 6.96E+03 |
|  |  | N6-(2-Hydroxyethyl)adenosine* | 1.24E+04 | 3.53E+03 | 1.32E+04 | 7.24E+02 | 7.01E+04 | 7.58E+04 |
| **Class I** | **Class II** | **Metabolite** | **DW** | | **YZ** | | **B21** | |
|  |  |  | **Average** | **SD** | **Average** | **SD** | **Average** | **SD** |
| **Nucleotides and derivatives** | **Nucleotides and derivatives** | 2-(Dimethylamino)guanosine* | 1.24E+04 | 3.53E+03 | 1.32E+04 | 7.24E+02 | 7.01E+04 | 7.58E+04 |
|  |  | 2-Deoxyribose-1-phosphate | 1.69E+06 | 5.70E+05 | 1.62E+06 | 2.80E+05 | 9.56E+05 | 1.58E+05 |
|  |  | 6-O-methylguanine | 1.52E+04 | 7.42E+02 | 1.96E+04 | 1.91E+03 | 2.63E+04 | 1.70E+04 |
|  |  | 5'-Deoxy-5'-(methylthio)adenosine | 1.17E+06 | 1.51E+05 | 1.17E+06 | 4.48E+04 | 1.30E+06 | 1.03E+05 |
|  |  | Uridine 5'-monophosphate | 9.31E+05 | 8.57E+04 | 6.49E+05 | 2.02E+05 | 2.55E+06 | 7.14E+05 |
|  |  | 2'-Deoxyinosine-5'-monophosphate | 4.91E+06 | 8.57E+05 | 4.10E+06 | 1.32E+05 | 3.62E+06 | 1.52E+06 |
|  |  | Cytosine | 1.05E+05 | 2.21E+04 | 1.27E+05 | 1.32E+03 | 1.12E+05 | 2.45E+04 |
|  |  | Xanthine | 4.25E+04 | 6.00E+03 | 4.57E+04 | 5.48E+03 | 6.61E+04 | 1.75E+04 |
|  |  | N-(1-Deoxy-1-fructosyl)Tryptophan | 1.69E+05 | 2.09E+04 | 1.60E+05 | 3.48E+04 | 6.58E+04 | 2.27E+04 |
|  |  | Uracil | 1.48E+05 | 4.20E+04 | 1.71E+05 | 7.05E+04 | 4.30E+05 | 2.67E+05 |
|  |  | Cytarabine | 3.80E+04 | 8.54E+03 | 4.06E+04 | 5.32E+03 | 5.16E+04 | 1.04E+04 |
|  |  | Isocytosine | 5.62E+04 | 1.67E+04 | 8.60E+04 | 1.30E+04 | 1.37E+05 | 5.25E+04 |
|  |  | Cytidine | 1.27E+05 | 1.93E+04 | 1.36E+05 | 1.80E+04 | 2.08E+05 | 1.14E+04 |
|  |  | Uridine 5'-diphospho-D-glucose | 9.21E+06 | 2.66E+06 | 1.04E+07 | 2.18E+06 | 1.63E+07 | 2.05E+06 |
|  |  | 2'-Deoxyadenosine-5'-monophosphate | 3.37E+04 | 1.13E+04 | 3.06E+04 | 1.03E+04 | 2.64E+04 | 7.05E+03 |
|  |  | 2'-O-Methyladenosine | 1.98E+04 | 8.88E+03 | 1.51E+04 | 2.92E+03 | 1.26E+04 | 1.15E+03 |
|  |  | 2-Deoxyribose-5'-phosphate | 7.51E+05 | 2.56E+05 | 5.49E+05 | 6.44E+04 | 3.77E+05 | 6.63E+04 |
|  |  | 3'-Adenylic Acid | 4.88E+04 | 4.93E+03 | 2.98E+04 | 7.13E+03 | 1.11E+05 | 3.38E+04 |
|  |  | Citicoline | 3.15E+04 | 8.75E+03 | 3.55E+04 | 7.39E+03 | 3.65E+04 | 6.61E+03 |
|  |  | 8-Azaguanine | 2.31E+04 | 3.29E+03 | 2.02E+04 | 2.68E+03 | 2.41E+04 | 9.16E+03 |
|  |  | 5-Aminoimidazole ribonucleotide | 1.15E+04 | 6.09E+03 | 5.30E+03 | 5.96E+02 | 1.31E+05 | 1.84E+05 |
|  |  | Cytidine 5'-monophosphate(Cytidylic acid) | 8.82E+04 | 2.84E+04 | 7.16E+04 | 4.16E+04 | 1.45E+05 | 5.00E+04 |
| **Others** | **Saccharides** | Inositol* | 2.07E+06 | 3.69E+05 | 2.12E+06 | 1.62E+05 | 1.69E+06 | 5.90E+05 |
|  |  | Melibiose | 2.90E+07 | 1.03E+07 | 2.45E+07 | 3.30E+06 | 2.43E+07 | 4.23E+06 |
|  |  | Lactobiose | 7.45E+05 | 2.47E+05 | 7.81E+05 | 1.46E+05 | 6.15E+05 | 3.16E+04 |
|  |  | D-Galactose* | 1.85E+06 | 3.78E+05 | 1.84E+06 | 1.34E+05 | 1.49E+06 | 4.28E+05 |
| **Class I** | **Class II** | **Metabolite** | **DW** | | **YZ** | | **B21** | |
|  |  |  | **Average** | **SD** | **Average** | **SD** | **Average** | **SD** |
| **Others** | **Saccharides** | Glucopyranose 6-Hydroxydecanoate | 9.44E+04 | 5.44E+04 | 1.25E+05 | 8.74E+04 | 1.77E+05 | 1.97E+05 |
|  |  | Rutinose | 9.61E+04 | 3.23E+04 | 1.25E+05 | 2.37E+04 | 1.46E+05 | 3.79E+04 |
|  |  | Allitol | 1.85E+05 | 3.38E+04 | 2.20E+05 | 5.56E+04 | 1.81E+05 | 6.92E+04 |
|  |  | D-Arabinose* | 3.95E+05 | 5.90E+04 | 3.25E+05 | 2.39E+04 | 3.88E+05 | 4.63E+04 |
|  |  | DL-Xylose* | 3.95E+05 | 5.90E+04 | 3.25E+05 | 2.39E+04 | 3.88E+05 | 4.63E+04 |
|  |  | Maltitol | 2.21E+06 | 2.77E+05 | 2.28E+06 | 1.03E+05 | 2.15E+06 | 2.81E+05 |
|  |  | D-Sorbitol | 1.22E+05 | 2.62E+04 | 1.23E+05 | 7.75E+03 | 1.03E+05 | 1.87E+04 |
|  |  | D-Fructose* | 2.04E+06 | 3.61E+05 | 2.23E+06 | 7.43E+04 | 1.65E+06 | 4.10E+05 |
|  |  | Rhamnose* | 1.53E+05 | 2.30E+04 | 1.57E+05 | 2.15E+04 | 2.00E+05 | 1.55E+04 |
|  |  | D-Pinitol | 9.93E+05 | 9.10E+04 | 1.21E+06 | 3.48E+04 | 9.67E+05 | 3.57E+04 |
|  |  | D-Threonic Acid | 4.32E+05 | 1.06E+05 | 4.99E+05 | 4.09E+04 | 8.75E+05 | 5.19E+04 |
|  |  | 2-Dehydro-3-deoxy-L-arabinonate* | 2.36E+05 | 3.74E+04 | 2.38E+05 | 4.29E+04 | 3.09E+05 | 2.58E+05 |
|  |  | L-Gulono-1,4-Lactone* | 1.46E+05 | 2.05E+04 | 1.43E+05 | 2.67E+04 | 1.86E+05 | 1.19E+05 |
|  |  | D-Melezitose | 1.63E+06 | 7.90E+05 | 1.84E+06 | 1.45E+05 | 4.29E+05 | 2.76E+05 |
|  |  | D-Fructose-1,6-biphosphate | 2.64E+05 | 1.65E+05 | 3.44E+05 | 1.11E+05 | 1.97E+05 | 8.40E+04 |
|  |  | Gluconic acid | 1.89E+07 | 9.44E+05 | 1.53E+07 | 4.09E+06 | 1.59E+07 | 2.82E+06 |
|  |  | D-Galacturonic acid* | 4.53E+05 | 4.84E+04 | 5.19E+05 | 5.36E+04 | 5.76E+05 | 6.79E+04 |
|  |  | D-Arabinono-1,4-lactone* | 1.78E+05 | 2.60E+04 | 1.85E+05 | 4.81E+04 | 3.11E+05 | 1.01E+05 |
|  |  | N-Acetyl-D-galactosamine | 3.09E+04 | 1.81E+03 | 2.79E+04 | 3.73E+03 | 2.17E+04 | 1.12E+04 |
|  |  | 1,6-anhydro-β-D-glucose | 1.20E+05 | 2.63E+04 | 1.11E+05 | 6.40E+03 | 9.15E+04 | 1.29E+04 |
|  |  | L-Fucitol | 4.98E+05 | 6.59E+04 | 5.01E+05 | 5.82E+04 | 6.36E+05 | 2.04E+05 |
|  |  | D-Glucono-1,5-lactone* | 1.89E+05 | 1.49E+04 | 2.58E+05 | 8.80E+04 | 1.45E+05 | 1.30E+05 |
|  |  | 1,5-Anhydro-D-glucitol | 3.02E+05 | 4.22E+04 | 2.91E+05 | 4.71E+04 | 3.44E+05 | 7.90E+04 |
|  |  | D-Glucose* | 1.89E+06 | 2.08E+05 | 2.25E+06 | 1.38E+05 | 1.70E+06 | 5.13E+05 |
|  |  | D-Glucose 1,6-bisphosphate | 4.28E+05 | 2.38E+05 | 1.08E+06 | 1.59E+05 | 3.65E+05 | 2.73E+05 |
|  |  | D-Cellobiose | 1.52E+06 | 4.78E+05 | 1.38E+06 | 3.44E+05 | 1.31E+06 | 3.20E+05 |
| **Class I** | **Class II** | **Metabolite** | **DW** | | **YZ** | | **B21** | |
|  |  |  | **Average** | **SD** | **Average** | **SD** | **Average** | **SD** |
| **Others** | **Saccharides** | Sedoheptulose | 1.17E+07 | 1.81E+06 | 1.16E+07 | 2.50E+06 | 1.26E+07 | 2.95E+06 |
|  |  | D-Galactaric acid | 2.44E+07 | 4.29E+06 | 2.20E+07 | 4.02E+06 | 2.33E+07 | 5.13E+06 |
|  |  | D-Saccharic acid | 3.07E+07 | 2.94E+06 | 2.96E+07 | 3.82E+06 | 3.07E+07 | 7.50E+06 |
|  |  | 1-O-Acetyl-Glucopyranose 6-Hydroxydecanoate | 1.88E+04 | 6.75E+03 | 7.82E+03 | 4.18E+03 | 1.75E+04 | 8.92E+03 |
|  |  | D-Mannitol* | 5.12E+04 | 8.20E+03 | 4.60E+04 | 1.24E+04 | 4.73E+04 | 1.36E+04 |
|  |  | Galactinol | 2.68E+06 | 4.54E+05 | 2.23E+06 | 1.07E+05 | 1.97E+06 | 1.19E+05 |
|  |  | D-Trehalose* | 6.05E+05 | 1.37E+05 | 5.61E+05 | 1.08E+05 | 5.31E+05 | 8.79E+04 |
|  |  | Planteose | 1.29E+04 | 1.85E+03 | 1.15E+04 | 6.12E+03 | 5.95E+03 | 2.14E+03 |
|  |  | D-Glucosamine 1-phosphate | 2.14E+05 | 8.90E+04 | 1.76E+05 | 2.93E+04 | 1.99E+05 | 4.08E+04 |
|  |  | D-Glucoronic acid* | 4.02E+05 | 3.33E+04 | 4.79E+05 | 1.89E+04 | 4.93E+05 | 8.76E+04 |
|  |  | D-Arabitol* | 5.63E+04 | 1.23E+04 | 4.94E+04 | 1.93E+04 | 4.97E+04 | 6.83E+03 |
|  |  | Raffinose* | 1.28E+07 | 4.72E+06 | 1.93E+07 | 4.72E+06 | 8.46E+06 | 4.96E+06 |
|  |  | Gal(1-3)[Fuc(1-2)]Gal(1-4)GlcSp | 4.02E+04 | 5.85E+03 | 2.80E+04 | 2.60E+03 | 1.55E+04 | 1.29E+04 |
|  |  | Dihydroxyacetone phosphate | 5.00E+05 | 1.15E+05 | 5.43E+05 | 2.97E+04 | 7.26E+05 | 3.12E+05 |
|  |  | Ribulose-5-phosphate | 1.22E+06 | 1.32E+05 | 1.78E+06 | 4.12E+05 | 1.36E+06 | 5.02E+05 |
|  |  | DMelezitose O-rhamnoside | 1.51E+05 | 3.04E+04 | 3.50E+05 | 1.27E+05 | 1.35E+05 | 9.99E+04 |
|  |  | D-Erythrose-4-phosphate | 2.64E+06 | 5.43E+05 | 2.73E+06 | 8.62E+05 | 5.87E+06 | 3.68E+06 |
|  |  | 3-Dehydro-L-Threonic Acid | 4.06E+06 | 6.42E+05 | 5.03E+06 | 2.24E+06 | 6.78E+06 | 4.96E+06 |
|  |  | Dulcitol* | 2.21E+05 | 4.88E+04 | 2.05E+05 | 3.77E+04 | 1.92E+05 | 2.56E+04 |
|  |  | N-Acetyl-D-glucosamine-1-phosphate | 6.39E+04 | 9.13E+03 | 6.49E+04 | 9.41E+03 | 6.61E+04 | 1.40E+04 |
|  |  | D-Sedoheptuiose 7-phosphate | 9.22E+06 | 2.81E+06 | 1.00E+07 | 2.41E+06 | 2.67E+07 | 1.78E+07 |
|  |  | 1-(sn-Glycero-3-phospho)-1D-myo-inositol | 8.33E+06 | 4.70E+06 | 9.28E+06 | 9.51E+05 | 5.03E+06 | 1.16E+06 |
|  |  | D-Glucose 6-phosphate* | 1.27E+06 | 6.19E+05 | 1.18E+06 | 3.80E+05 | 3.35E+06 | 2.10E+06 |
|  |  | 4-O-galactopyranosylxylose | 1.79E+05 | 9.41E+03 | 1.15E+05 | 3.21E+03 | 1.05E+05 | 3.53E+04 |
|  |  | Xylitol* | 3.08E+04 | 8.64E+03 | 2.82E+04 | 5.12E+03 | 4.11E+04 | 3.08E+03 |
|  |  | Ribitol* | 5.12E+04 | 6.84E+03 | 4.01E+04 | 8.72E+03 | 4.72E+04 | 9.25E+03 |
| **Class I** | **Class II** | **Metabolite** | **DW** | | **YZ** | | **B21** | |
|  |  |  | **Average** | **SD** | **Average** | **SD** | **Average** | **SD** |
| **Others** | **Saccharides** | D-Mannose* | 1.45E+06 | 4.00E+05 | 1.59E+06 | 1.76E+05 | 1.21E+06 | 5.34E+05 |
|  |  | D-Ribose | 1.68E+04 | 3.16E+03 | 1.85E+04 | 4.51E+03 | 3.88E+04 | 1.71E+04 |
|  |  | Neu5Gc2-6Gal1-4GlcNAcSp | 2.40E+03 | 1.39E+03 | 9.00E+00 | 0.00E+00 | 3.44E+03 | 3.98E+02 |
|  |  | D-Xylonic acid | 3.65E+06 | 1.02E+06 | 4.32E+06 | 3.19E+05 | 5.47E+06 | 2.10E+06 |
|  |  | Neu5Ac2-3Gal1-3GlcNAcSp | 1.19E+04 | 2.39E+03 | 1.01E+04 | 4.62E+03 | 1.13E+04 | 1.15E+03 |
|  |  | Isomaltulose* | 7.47E+05 | 1.44E+05 | 7.13E+05 | 4.58E+04 | 6.23E+05 | 1.90E+05 |
|  |  | D-Fucose* | 2.29E+04 | 2.78E+03 | 2.67E+04 | 6.30E+03 | 2.85E+04 | 1.15E+04 |
|  |  | D-Glucurono-6,3-lactone | 5.20E+04 | 1.62E+04 | 4.47E+04 | 3.35E+03 | 3.81E+04 | 2.16E+04 |
|  |  | Neu5Ac2-6LacDiNAcSp | 2.66E+03 | 1.29E+03 | 9.00E+00 | 0.00E+00 | 9.00E+00 | 0.00E+00 |
|  |  | D-Fructose 6-Phosphate* | 9.46E+05 | 8.20E+05 | 1.90E+06 | 9.09E+05 | 1.55E+06 | 2.07E+06 |
|  |  | Trehalose 6-phosphate | 4.75E+06 | 7.81E+05 | 2.91E+06 | 4.33E+05 | 3.51E+06 | 2.21E+06 |
|  |  | D-Maltose* | 8.21E+05 | 1.78E+05 | 7.18E+05 | 1.38E+05 | 7.46E+05 | 8.63E+04 |
|  |  | D-Sucrose* | 5.67E+05 | 9.09E+04 | 5.58E+05 | 2.84E+04 | 4.62E+05 | 2.17E+04 |
|  |  | 4-(3-Methylbutanoyl)Sucrose | 4.11E+03 | 4.04E+02 | 3.73E+03 | 1.18E+03 | 4.77E+03 | 2.31E+03 |
|  |  | Verbascose | 1.67E+04 | 5.55E+03 | 1.69E+04 | 4.38E+03 | 3.18E+03 | 1.92E+03 |
|  |  | D-Maltotetraose | 7.42E+05 | 1.61E+05 | 1.36E+06 | 6.19E+05 | 1.47E+05 | 1.01E+05 |
|  |  | Solatriose | 1.16E+05 | 6.09E+04 | 1.61E+05 | 8.20E+04 | 1.67E+05 | 1.03E+05 |
|  |  | Glucarate O-Phosphoric acid | 1.27E+06 | 8.36E+04 | 1.81E+06 | 3.58E+05 | 1.48E+06 | 5.71E+05 |
|  |  | Manninotriose | 4.60E+04 | 2.62E+03 | 5.14E+04 | 8.71E+03 | 1.27E+04 | 9.27E+03 |
|  |  | Sorbitol-6-phosphate | 4.48E+05 | 1.85E+05 | 6.66E+05 | 3.14E+05 | 1.08E+06 | 5.98E+05 |
|  |  | D-Panose* | 1.81E+06 | 6.93E+05 | 4.21E+06 | 1.75E+06 | 1.22E+06 | 6.01E+05 |
|  |  | Glucose-1-phosphate* | 1.22E+06 | 3.65E+05 | 1.64E+06 | 5.20E+05 | 2.91E+06 | 1.92E+06 |
|  |  | Stachyose | 5.35E+06 | 2.69E+06 | 8.53E+06 | 4.23E+06 | 1.24E+06 | 1.37E+06 |
|  |  | Nystose | 7.27E+06 | 2.54E+06 | 9.26E+06 | 3.12E+06 | 1.15E+06 | 1.03E+06 |
|  |  | D-Glucosamine | 1.13E+05 | 3.05E+03 | 1.75E+05 | 7.57E+04 | 1.74E+05 | 6.23E+04 |
|  |  | Maltotriose | 1.41E+06 | 4.33E+05 | 1.71E+06 | 2.83E+05 | 8.85E+05 | 1.46E+05 |
| **Class I** | **Class II** | **Metabolite** | **DW** | | **YZ** | | **B21** | |
|  |  |  | **Average** | **SD** | **Average** | **SD** | **Average** | **SD** |
| **Others** | **Vitamin** | Isoascorbic acid 2-O-glucoside | 8.98E+04 | 1.16E+04 | 8.94E+04 | 2.87E+04 | 6.94E+04 | 7.24E+03 |
|  |  | Pyridoxal-5'-phosphate | 6.42E+04 | 2.38E+03 | 5.28E+04 | 7.29E+03 | 1.14E+05 | 5.73E+04 |
|  |  | Nicotinamide | 1.88E+05 | 4.88E+04 | 1.94E+05 | 1.93E+04 | 2.04E+05 | 1.38E+04 |
|  |  | Nicotinic acid (Vitamin B3) | 1.08E+06 | 9.25E+04 | 8.05E+05 | 8.90E+04 | 8.44E+05 | 7.69E+04 |
|  |  | D-Pantothenic Acid | 6.40E+05 | 9.44E+04 | 4.87E+05 | 8.44E+04 | 4.22E+05 | 1.93E+05 |
|  |  | Erythorbic Acid; Isoascorbic Acid | 5.21E+04 | 6.36E+03 | 5.00E+04 | 4.84E+03 | 9.88E+04 | 2.80E+04 |
|  |  | Riboflavin (Vitamin B2) | 3.20E+04 | 3.76E+03 | 3.37E+04 | 6.31E+03 | 2.26E+04 | 3.00E+03 |
|  |  | Menatetrenone (Vitamin K2) | 2.39E+05 | 2.37E+04 | 2.20E+05 | 1.63E+04 | 2.09E+05 | 2.96E+04 |
|  |  | 4-Pyridoxic acid | 2.52E+04 | 5.62E+03 | 2.52E+04 | 5.90E+03 | 2.13E+04 | 1.05E+04 |
|  |  | Biotin | 5.37E+05 | 8.75E+04 | 3.52E+05 | 5.32E+04 | 4.38E+05 | 1.90E+05 |
|  |  | Isonicotinic acid | 3.23E+05 | 5.73E+04 | 3.14E+05 | 1.58E+04 | 2.57E+05 | 8.29E+04 |
|  |  | Pyridoxine-5'-O-glucoside | 2.65E+05 | 6.20E+04 | 2.41E+05 | 3.98E+04 | 2.60E+05 | 1.17E+05 |
|  |  | Phylloquinone (Vitamin K1) | 6.58E+03 | 4.04E+02 | 6.27E+03 | 7.26E+02 | 5.94E+03 | 1.82E+02 |
|  |  | Pyridoxine | 1.24E+05 | 3.03E+04 | 9.43E+04 | 1.45E+04 | 1.29E+05 | 4.24E+04 |
|  |  | Orotic acid (Vitamin B13) | 2.02E+05 | 3.83E+04 | 1.78E+05 | 5.07E+04 | 2.40E+05 | 7.76E+04 |
|  |  | Nicotinate D-ribonucleoside | 7.55E+05 | 9.97E+04 | 7.34E+05 | 8.50E+04 | 5.92E+05 | 2.97E+05 |
|  |  | Dehydroascorbic acid | 3.18E+04 | 6.78E+03 | 2.03E+04 | 2.28E+03 | 3.67E+04 | 8.94E+03 |
|  |  | Pyridoxal | 4.60E+05 | 3.12E+05 | 3.70E+05 | 2.81E+04 | 2.68E+05 | 5.95E+04 |
|  |  | 2-O-α-D-Glucopyranosyl-L-ascorbic acid | 2.88E+05 | 4.02E+04 | 3.83E+05 | 7.75E+04 | 5.21E+05 | 2.76E+05 |

| **Supplementary Table S2. Metabolites were identified in DW and YZ** | | | | | | | | | | | | | | | |
| --- | --- | --- | --- | --- | --- | --- | --- | --- | --- | --- | --- | --- | --- | --- | --- |
| **Index** | **Formula** | **Compounds** | **Class I** | **Class II** | **DW-1-1** | **DW-1-2** | **DW-1-3** | **YZ-1-1** | **YZ-1-2** | **YZ-1-3** | **VIP** | **P-value** | **Fold_Change** | **Log2FC** | **Type** |
| MW0156666 | C10H18N4O7 | Ser-Asn-Ser | Amino acids and derivatives | Amino acids and derivatives | 5.26E+02 | 7.83E+03 | 7.87E+03 | 6.30E+04 | 1.60E+03 | 1.14E+06 | 1.06E+00 | 3.96E-01 | 7.43E+01 | 6.21E+00 | up |
| MW0108646 | C7H11NO3 | N-Acetylproline | Amino acids and derivatives | Amino acids and derivatives | 5.46E+04 | 8.11E+04 | 9.22E+04 | 4.55E+04 | 3.01E+04 | 3.50E+04 | 1.74E+00 | 5.67E-02 | 4.85E-01 | -1.04E+00 | down |
| MW0144217 | C14H18N2O6 | Abu-Val-OH | Amino acids and derivatives | Amino acids and derivatives | 2.82E+04 | 1.05E+04 | 1.12E+04 | 3.24E+05 | 6.95E+03 | 2.27E+06 | 1.14E+00 | 3.53E-01 | 5.22E+01 | 5.71E+00 | up |
| Zmjp000182 | C7H16N4O2 | N-Monomethyl-L-arginine* | Amino acids and derivatives | Amino acids and derivatives | 2.56E+04 | 5.27E+04 | 3.44E+04 | 1.59E+05 | 7.51E+04 | 5.74E+04 | 1.51E+00 | 1.92E-01 | 2.59E+00 | 1.37E+00 | up |
| MWS04412 | C8H18N2O2 | N(6),N(6)-Dimethyl-L-lysine | Amino acids and derivatives | Amino acids and derivatives | 2.88E+06 | 4.13E+06 | 4.23E+06 | 1.17E+07 | 6.57E+06 | 6.56E+06 | 1.68E+00 | 1.09E-01 | 2.21E+00 | 1.14E+00 | up |
| Zmyn000155 | C7H14N2O3 | N-α-Acetyl-L-ornithine | Amino acids and derivatives | Amino acids and derivatives | 2.10E+05 | 3.96E+05 | 3.30E+05 | 2.34E+06 | 8.86E+05 | 5.60E+05 | 1.57E+00 | 2.24E-01 | 4.05E+00 | 2.02E+00 | up |
| Zmdp000292 | C7H16N4O2 | Arginine methyl ester* | Amino acids and derivatives | Amino acids and derivatives | 2.82E+04 | 5.73E+04 | 2.54E+04 | 1.88E+05 | 9.01E+04 | 9.24E+04 | 1.71E+00 | 1.05E-01 | 3.34E+00 | 1.74E+00 | up |
| MWS4471 | C6H10N2O4 | N-Alpha-Acetyl-L-Asparagine | Amino acids and derivatives | Amino acids and derivatives | 1.57E+03 | 6.81E+03 | 4.45E+03 | 2.99E+04 | 5.02E+03 | 1.10E+04 | 1.28E+00 | 2.76E-01 | 3.58E+00 | 1.84E+00 | up |
| MW0158711 | C19H20N2O8 | TyrMe-Ser-OH | Amino acids and derivatives | Amino acids and derivatives | 1.08E+04 | 6.10E+04 | 3.62E+04 | 2.66E+05 | 7.00E+04 | 1.22E+06 | 1.50E+00 | 3.07E-01 | 1.44E+01 | 3.85E+00 | up |
| Lmyn012331 | C18H34O2 | Petroselinic acid* | Lipids | Free fatty acids | 5.78E+06 | 7.19E+06 | 7.54E+06 | 1.62E+07 | 3.72E+07 | 1.47E+07 | 1.73E+00 | 1.59E-01 | 3.32E+00 | 1.73E+00 | up |
| mws2623 | C18H34O2 | 11-Octadecanoic acid(Vaccenic acid)* | Lipids | Free fatty acids | 4.25E+06 | 5.86E+06 | 5.47E+06 | 1.41E+07 | 3.25E+07 | 1.24E+07 | 1.76E+00 | 1.53E-01 | 3.79E+00 | 1.92E+00 | up |
| MW0156024 | C18H24N4O6 | Pro-Tyr-Asn | Amino acids and derivatives | Amino acids and derivatives | 2.44E+03 | 1.32E+03 | 7.69E+03 | 3.04E+03 | 1.54E+04 | 1.73E+04 | 1.18E+00 | 2.04E-01 | 3.12E+00 | 1.64E+00 | up |
| MW0107005 | C14H19N3O5 | Gln-Tyr | Amino acids and derivatives | Amino acids and derivatives | 3.93E+06 | 1.97E+06 | 9.33E+05 | 5.63E+06 | 4.79E+06 | 7.39E+06 | 1.57E+00 | 3.57E-02 | 2.60E+00 | 1.38E+00 | up |
| MW0149687 | C22H35N7O13 | Gln-Glu-Ser-Gln-Asp | Amino acids and derivatives | Amino acids and derivatives | 5.91E+04 | 1.29E+04 | 9.80E+03 | 3.99E+04 | 1.64E+05 | 4.94E+04 | 1.33E+00 | 2.87E-01 | 3.09E+00 | 1.63E+00 | up |
| Zmyn000083 | C6H14O12P2 | D-Glucose 1,6-bisphosphate | Others | Saccharides | 1.56E+05 | 5.30E+05 | 5.97E+05 | 1.22E+06 | 9.07E+05 | 1.10E+06 | 1.50E+00 | 2.22E-02 | 2.51E+00 | 1.33E+00 | up |
| MW0144720 | C12H21N3O5 | Ala-Pro-Thr | Amino acids and derivatives | Amino acids and derivatives | 1.17E+05 | 6.86E+04 | 2.55E+04 | 1.59E+05 | 1.57E+05 | 2.02E+05 | 1.53E+00 | 4.01E-02 | 2.46E+00 | 1.30E+00 | up |
| Wafn003633 | C18H32O9 | 1-O-Acetyl-Glucopyranose 6-Hydroxydecanoate | Others | Saccharides | 2.57E+04 | 1.86E+04 | 1.22E+04 | 1.24E+04 | 4.20E+03 | 6.87E+03 | 1.52E+00 | 8.66E-02 | 4.15E-01 | -1.27E+00 | down |
| MW0108101 | C12H26N4O3 | Lys-Lys | Amino acids and derivatives | Amino acids and derivatives | 5.09E+04 | 7.15E+04 | 7.37E+04 | 1.83E+05 | 3.99E+05 | 1.43E+05 | 1.73E+00 | 1.56E-01 | 3.70E+00 | 1.89E+00 | up |
| mws0396 | C18H34O2 | Elaidic Acid* | Lipids | Free fatty acids | 1.63E+07 | 1.97E+07 | 2.10E+07 | 4.98E+07 | 9.47E+07 | 3.92E+07 | 1.76E+00 | 1.30E-01 | 3.22E+00 | 1.69E+00 | up |
| MW0014259 | C18H34O3 | 3-Oxooctadecanoic acid | Organic acids | Organic acids | 3.16E+04 | 2.40E+04 | 3.13E+04 | 5.92E+04 | 8.11E+04 | 6.60E+04 | 1.89E+00 | 1.55E-02 | 2.37E+00 | 1.25E+00 | up |
| **Index** | **Formula** | **Compounds** | **Class I** | **Class II** | **DW-1-1** | **DW-1-2** | **DW-1-3** | **YZ-1-1** | **YZ-1-2** | **YZ-1-3** | **VIP** | **P-value** | **Fold_Change** | **Log2FC** | **Type** |
| MW0158225 | C20H24N4O6 | Trp-Pro-Asp | Amino acids and derivatives | Amino acids and derivatives | 1.19E+03 | 3.25E+03 | 1.53E+04 | 1.35E+04 | 3.54E+04 | 2.76E+04 | 1.45E+00 | 8.04E-02 | 3.87E+00 | 1.95E+00 | up |
| MW0157568 | C10H18N4O6 | Thr-Asn-Gly | Amino acids and derivatives | Amino acids and derivatives | 1.54E+04 | 7.89E+04 | 2.58E+04 | 7.21E+04 | 7.56E+04 | 1.13E+05 | 1.39E+00 | 1.29E-01 | 2.17E+00 | 1.12E+00 | up |
| pme0008 | C6H13N3O3 | L-Citrulline | Amino acids and derivatives | Amino acids and derivatives | 1.43E+06 | 1.63E+06 | 2.04E+06 | 3.01E+06 | 5.33E+06 | 2.13E+06 | 1.48E+00 | 1.99E-01 | 2.05E+00 | 1.04E+00 | up |
| pmb2653 | C24H42O20 | DMelezitose O-rhamnoside | Others | Saccharides | 1.78E+05 | 1.58E+05 | 1.18E+05 | 3.25E+05 | 4.87E+05 | 2.37E+05 | 1.71E+00 | 1.06E-01 | 2.31E+00 | 1.21E+00 | up |
| MW0154413 | C27H45N5O20 | Neu5Gc2-6Gal1-4GlcNAcSp | Others | Saccharides | 3.96E+03 | 1.30E+03 | 1.94E+03 | 9.00E+00 | 9.00E+00 | 9.00E+00 | 1.96E+00 | 9.67E-02 | 3.75E-03 | -8.06E+00 | down |
| MW0154405 | C29H48N6O19 | Neu5Ac2-6LacDiNAcSp | Others | Saccharides | 3.97E+03 | 1.40E+03 | 2.61E+03 | 9.00E+00 | 9.00E+00 | 9.00E+00 | 1.96E+00 | 7.06E-02 | 3.38E-03 | -8.21E+00 | down |
| MW0158132 | C22H27N7O5 | Trp-Gln-His | Amino acids and derivatives | Amino acids and derivatives | 1.36E+04 | 1.22E+04 | 1.26E+04 | 7.43E+03 | 6.08E+03 | 5.21E+03 | 1.89E+00 | 1.99E-03 | 4.86E-01 | -1.04E+00 | down |
| MW0149607 | C20H34N6O9 | Gln-Ala-Ala-Asp-Val | Amino acids and derivatives | Amino acids and derivatives | 4.19E+04 | 6.09E+04 | 1.69E+05 | 2.92E+04 | 6.13E+04 | 3.96E+04 | 1.09E+00 | 3.55E-01 | 4.79E-01 | -1.06E+00 | down |
| Zmzp000145 | C9H20N2O2 | Trimethyllysine | Amino acids and derivatives | Amino acids and derivatives | 3.25E+04 | 1.14E+05 | 3.84E+04 | 1.51E+05 | 9.89E+04 | 1.26E+05 | 1.43E+00 | 1.19E-01 | 2.04E+00 | 1.03E+00 | up |
| mws1589 | C18H32O16 | D-Panose* | Others | Saccharides | 2.59E+06 | 1.25E+06 | 1.59E+06 | 2.84E+06 | 6.18E+06 | 3.61E+06 | 1.59E+00 | 1.27E-01 | 2.32E+00 | 1.22E+00 | up |

| **Supplementary Table S3. Metabolites were identified in DW and B21** | | | | | | | | | | | | | | | |
| --- | --- | --- | --- | --- | --- | --- | --- | --- | --- | --- | --- | --- | --- | --- | --- |
| **Index** | **Formula** | **Compounds** | **Class I** | **Class II** | **DW-1-1** | **DW-1-2** | **DW-1-3** | **B21-1-1** | **B21-1-2** | **B21-1-3** | **VIP** | **P-value** | **Fold_Change** | **Log2FC** | **Type** |
| MW0153524 | C25H37N5O9S2 | Met-Cys-Asp-Phe-Thr | Amino acids and derivatives | Amino acids and derivatives | 1.04E+04 | 1.16E+04 | 1.15E+04 | 3.06E+03 | 1.96E+03 | 2.07E+03 | 1.60E+00 | 6.85E-05 | 2.11E-01 | -2.24E+00 | down |
| MW0157013 | C8H16N2O4S | Ser-Met | Amino acids and derivatives | Amino acids and derivatives | 2.03E+06 | 2.15E+06 | 2.06E+06 | 8.84E+05 | 8.05E+05 | 7.11E+05 | 1.61E+00 | 7.53E-05 | 3.84E-01 | -1.38E+00 | down |
| pme2074 | C18H29NO4 | Jasmonoyl-L-Isoleucine | Amino acids and derivatives | Amino acids and derivatives | 1.80E+03 | 1.75E+03 | 1.86E+03 | 2.73E+04 | 2.95E+04 | 2.85E+04 | 1.62E+00 | 5.61E-04 | 1.57E+01 | 3.98E+00 | up |
| Hmqn003054 | C18H34O5 | 9,10,11-Trihydroxy-12-octadecenoic acid | Lipids | Free fatty acids | 1.26E+04 | 1.51E+04 | 1.93E+04 | 5.10E+04 | 6.01E+04 | 5.00E+04 | 1.58E+00 | 1.36E-03 | 3.43E+00 | 1.78E+00 | up |
| MW0158478 | C19H23N5O7 | Tyr-His-Asp | Amino acids and derivatives | Amino acids and derivatives | 1.75E+04 | 1.64E+04 | 2.10E+04 | 5.70E+03 | 2.36E+03 | 4.39E+03 | 1.55E+00 | 1.75E-03 | 2.26E-01 | -2.14E+00 | down |
| MW0153883 | C20H22N2O7S | Met-TyrMe-OH | Amino acids and derivatives | Amino acids and derivatives | 5.76E+05 | 5.98E+05 | 5.04E+05 | 6.44E+04 | 4.75E+04 | 4.39E+04 | 1.61E+00 | 2.18E-03 | 9.28E-02 | -3.43E+00 | down |
| pmn001694 | C18H34O5 | 9,10,13-Trihydroxy-11-Octadecenoic Acid | Lipids | Free fatty acids | 1.75E+05 | 2.09E+05 | 2.96E+05 | 1.03E+06 | 1.26E+06 | 1.05E+06 | 1.59E+00 | 2.21E-03 | 4.92E+00 | 2.30E+00 | up |
| MW0153708 | C23H29N3O6S1 | Met-Tyr-Tyr | Amino acids and derivatives | Amino acids and derivatives | 2.16E+05 | 2.51E+05 | 1.86E+05 | 4.46E+04 | 1.96E+04 | 8.65E+04 | 1.42E+00 | 3.52E-03 | 2.31E-01 | -2.12E+00 | down |
| MWS2413 | C5H9NO3 | N-acetyl-beta-alanine | Amino acids and derivatives | Amino acids and derivatives | 4.68E+04 | 3.75E+04 | 4.12E+04 | 1.37E+04 | 2.25E+04 | 1.13E+04 | 1.49E+00 | 4.70E-03 | 3.78E-01 | -1.40E+00 | down |
| Waln010449 | C26H52NO9P | 1-(2,3-dihydroxypropoxy)-3-(((2-(dimethylamino)ethoxy)(hydroxy)phosphoryl)oxy)propan-2-yl (E)-hexadec-9-enoate | Lipids | LPE | 5.70E+04 | 4.48E+04 | 5.92E+04 | 3.01E+04 | 2.16E+04 | 1.81E+04 | 1.53E+00 | 6.97E-03 | 4.33E-01 | -1.21E+00 | down |
| mws0889 | C4H8O5 | D-Threonic Acid | Others | Saccharides | 3.26E+05 | 5.37E+05 | 4.33E+05 | 9.26E+05 | 8.77E+05 | 8.22E+05 | 1.51E+00 | 8.05E-03 | 2.02E+00 | 1.02E+00 | up |
| mws1593 | C24H42O21 | D-Maltotetraose | Others | Saccharides | 7.87E+05 | 5.63E+05 | 8.76E+05 | 1.31E+05 | 5.45E+04 | 2.55E+05 | 1.45E+00 | 9.20E-03 | 1.98E-01 | -2.34E+00 | down |
| pme0075 | C7H11NO5 | N-Acetyl-L-glutamic acid | Amino acids and derivatives | Amino acids and derivatives | 6.13E+05 | 1.15E+06 | 1.61E+06 | 3.91E+06 | 3.51E+06 | 2.61E+06 | 1.43E+00 | 1.19E-02 | 2.97E+00 | 1.57E+00 | up |
| Hmqp000191 | C18H32O16 | Planteose | Others | Saccharides | 1.10E+04 | 1.30E+04 | 1.47E+04 | 4.28E+03 | 5.21E+03 | 8.37E+03 | 1.41E+00 | 1.36E-02 | 4.61E-01 | -1.12E+00 | down |
| MW0151026 | C16H25N5O6S1 | His-Met-Glu | Amino acids and derivatives | Amino acids and derivatives | 3.13E+06 | 3.71E+06 | 2.75E+06 | 1.78E+06 | 4.04E+05 | 8.16E+05 | 1.38E+00 | 1.49E-02 | 3.13E-01 | -1.67E+00 | down |
| MW0156686 | C17H29N5O10 | Ser-Asp-Ser-Gly-Val | Amino acids and derivatives | Amino acids and derivatives | 6.36E+04 | 5.31E+04 | 8.37E+04 | 2.19E+04 | 9.18E+03 | 1.24E+04 | 1.55E+00 | 1.66E-02 | 2.17E-01 | -2.21E+00 | down |
| Sazp003601 | C18H28O9 | Tuberonic acid glucoside | Organic acids | Organic acids | 1.26E+05 | 1.68E+05 | 1.18E+05 | 8.03E+04 | 4.85E+04 | 2.85E+04 | 1.39E+00 | 1.73E-02 | 3.82E-01 | -1.39E+00 | down |
| MWSmce486 | C18H32O16 | Manninotriose | Others | Saccharides | 4.73E+04 | 4.30E+04 | 4.78E+04 | 7.33E+03 | 7.39E+03 | 2.34E+04 | 1.42E+00 | 1.86E-02 | 2.76E-01 | -1.86E+00 | down |
| **Index** | **Formula** | **Compounds** | **Class I** | **Class II** | **DW-1-1** | **DW-1-2** | **DW-1-3** | **B21-1-1** | **B21-1-2** | **B21-1-3** | **VIP** | **P-value** | **Fold_Change** | **Log2FC** | **Type** |
| MW0153543 | C16H25N5O6S1 | Met-Glu-His | Amino acids and derivatives | Amino acids and derivatives | 2.26E+06 | 2.63E+06 | 1.98E+06 | 1.35E+06 | 3.19E+05 | 6.42E+05 | 1.36E+00 | 1.96E-02 | 3.37E-01 | -1.57E+00 | down |
| MW0158719 | C28H24N2O7 | Tyr-Nap-OH | Amino acids and derivatives | Amino acids and derivatives | 4.83E+06 | 3.72E+06 | 5.91E+06 | 2.73E+06 | 6.61E+05 | 9.56E+05 | 1.40E+00 | 2.03E-02 | 3.01E-01 | -1.73E+00 | down |
| MW0156774 | C22H33N7O11 | Ser-His-Glu-Ala-Glu | Amino acids and derivatives | Amino acids and derivatives | 3.51E+04 | 4.88E+04 | 3.44E+04 | 1.50E+04 | 1.08E+04 | 1.30E+04 | 1.56E+00 | 2.39E-02 | 3.28E-01 | -1.61E+00 | down |
| MW0151128 | C24H30N6O10 | His-Tyr-Glu-Asp | Amino acids and derivatives | Amino acids and derivatives | 1.03E+05 | 1.58E+05 | 1.58E+05 | 5.38E+04 | 3.46E+04 | 4.89E+04 | 1.53E+00 | 2.75E-02 | 3.28E-01 | -1.61E+00 | down |
| MW0155308 | C27H29N3O5 | Phe-Phe-Tyr | Amino acids and derivatives | Amino acids and derivatives | 2.64E+04 | 2.22E+04 | 3.57E+04 | 1.15E+04 | 6.73E+03 | 7.54E+03 | 1.55E+00 | 2.79E-02 | 3.05E-01 | -1.71E+00 | down |
| MW0146227 | C22H33N7O8 | Asp-Phe-Ser-Arg | Amino acids and derivatives | Amino acids and derivatives | 1.90E+04 | 7.58E+03 | 1.03E+03 | 3.85E+04 | 4.80E+04 | 2.57E+04 | 1.20E+00 | 2.93E-02 | 4.07E+00 | 2.03E+00 | up |
| MWS0813 | C5H7NO3 | 5-Oxoproline | Amino acids and derivatives | Amino acids and derivatives | 1.80E+04 | 3.24E+04 | 4.18E+04 | 5.67E+04 | 7.50E+04 | 5.38E+04 | 1.33E+00 | 3.14E-02 | 2.01E+00 | 1.01E+00 | up |
| MWSmce210 | C7H4O6 | Chelidonic acid | Organic acids | Organic acids | 3.81E+06 | 2.13E+06 | 3.39E+06 | 3.68E+05 | 6.70E+05 | 7.10E+05 | 1.54E+00 | 3.29E-02 | 1.87E-01 | -2.42E+00 | down |
| MW0146364 | C22H18N2O8 | Asp-Nap-OH | Amino acids and derivatives | Amino acids and derivatives | 3.24E+04 | 2.24E+04 | 4.18E+04 | 1.39E+04 | 5.32E+03 | 7.80E+03 | 1.48E+00 | 3.67E-02 | 2.80E-01 | -1.84E+00 | down |
| mws4163 | C24H42O21 | Nystose | Others | Saccharides | 9.22E+06 | 4.40E+06 | 8.18E+06 | 9.08E+05 | 2.53E+05 | 2.28E+06 | 1.39E+00 | 3.81E-02 | 1.58E-01 | -2.67E+00 | down |
| MW0158702 | C22H22N4O7 | TyrMe-His-OH | Amino acids and derivatives | Amino acids and derivatives | 4.78E+03 | 2.27E+03 | 4.06E+03 | 9.00E+00 | 9.00E+00 | 9.00E+00 | 1.62E+00 | 3.84E-02 | 2.43E-03 | -8.68E+00 | down |
| MW0155108 | C24H35N5O8 | Phe-Asn-Ile-Glu | Amino acids and derivatives | Amino acids and derivatives | 1.57E+05 | 1.07E+05 | 1.73E+05 | 9.76E+04 | 3.00E+04 | 2.10E+04 | 1.34E+00 | 3.88E-02 | 3.40E-01 | -1.56E+00 | down |
| MW0148009 | C19H33N5O8S2 | Cys-Gly-Asp-Val-Met | Amino acids and derivatives | Amino acids and derivatives | 8.27E+03 | 4.00E+03 | 7.85E+03 | 9.00E+00 | 9.00E+00 | 9.00E+00 | 1.62E+00 | 3.89E-02 | 1.34E-03 | -9.54E+00 | down |
| pmp001251 | C26H50NO7P | LysoPC 18:2(2n isomer) | Lipids | LPC | 1.36E+07 | 8.36E+06 | 1.24E+07 | 7.64E+06 | 1.88E+06 | 1.77E+06 | 1.34E+00 | 3.92E-02 | 3.28E-01 | -1.61E+00 | down |
| MEDP1341 | C26H50NO7P | 1-(9Z,12Z-octadecadienoyl)-sn-glycero-3-phosphocholine | Lipids | LPC | 1.36E+07 | 8.36E+06 | 1.24E+07 | 7.64E+06 | 1.88E+06 | 1.77E+06 | 1.34E+00 | 3.92E-02 | 3.28E-01 | -1.61E+00 | down |
| Lmdn000248 | C30H52O26 | Verbascose | Others | Saccharides | 1.03E+04 | 1.97E+04 | 2.00E+04 | 2.18E+03 | 1.98E+03 | 5.39E+03 | 1.46E+00 | 4.03E-02 | 1.91E-01 | -2.39E+00 | down |
| pmb1283 | C18H33N5O6S | L-Glutaminyl-L-valyl-L-valyl-L-cysteine | Amino acids and derivatives | Amino acids and derivatives | 4.52E+04 | 3.76E+04 | 4.28E+04 | 3.06E+04 | 1.12E+04 | 1.73E+04 | 1.36E+00 | 4.66E-02 | 4.71E-01 | -1.08E+00 | down |
| mws0154 | C7H10O5 | Shikimic acid | Organic acids | Organic acids | 2.83E+05 | 1.78E+05 | 2.00E+05 | 8.32E+05 | 1.06E+06 | 5.50E+05 | 1.51E+00 | 5.13E-02 | 3.69E+00 | 1.88E+00 | up |
| MWS201054 | C6H11NO4 | O-Acetyl-L-homoserine | Amino acids and derivatives | Amino acids and derivatives | 4.64E+04 | 3.39E+04 | 2.72E+04 | 7.46E+04 | 1.26E+05 | 8.33E+04 | 1.48E+00 | 5.25E-02 | 2.64E+00 | 1.40E+00 | up |
| pmb0854 | C26H48NO7P | LysoPC 18:3 | Lipids | LPC | 1.33E+05 | 1.46E+05 | 1.76E+05 | 2.49E+05 | 3.88E+05 | 2.81E+05 | 1.49E+00 | 5.69E-02 | 2.02E+00 | 1.01E+00 | up |
| MWS0811 | C6H11NO2 | L-Pipecolic Acid | Organic acids | Organic acids | 1.67E+06 | 2.25E+06 | 2.23E+06 | 5.00E+06 | 8.95E+06 | 6.01E+06 | 1.55E+00 | 5.70E-02 | 3.25E+00 | 1.70E+00 | up |
| **Index** | **Formula** | **Compounds** | **Class I** | **Class II** | **DW-1-1** | **DW-1-2** | **DW-1-3** | **B21-1-1** | **B21-1-2** | **B21-1-3** | **VIP** | **P-value** | **Fold_Change** | **Log2FC** | **Type** |
| MW0109886 | C9H18N2O4S | Thr-Met | Amino acids and derivatives | Amino acids and derivatives | 7.86E+05 | 4.41E+05 | 3.98E+05 | 1.36E+05 | 1.93E+04 | 2.08E+05 | 1.27E+00 | 5.79E-02 | 2.23E-01 | -2.16E+00 | down |
| Zmzn000113 | C5H9NO4 | L-threo-3-Methylaspartate | Amino acids and derivatives | Amino acids and derivatives | 1.22E+06 | 2.54E+06 | 2.95E+06 | 5.57E+06 | 8.98E+06 | 5.01E+06 | 1.41E+00 | 5.82E-02 | 2.91E+00 | 1.54E+00 | up |
| MW0149376 | C26H45N3O20 | Gal(1-3)[Fuc(1-2)]Gal(1-4)GlcSp | Others | Saccharides | 4.54E+04 | 3.39E+04 | 4.13E+04 | 2.96E+04 | 1.29E+04 | 4.09E+03 | 1.22E+00 | 6.29E-02 | 3.86E-01 | -1.37E+00 | down |
| pme2758 | C5H9NO5 | 4-Hydroxy-L-glutamic acid | Amino acids and derivatives | Amino acids and derivatives | 1.32E+05 | 1.82E+05 | 1.88E+05 | 3.08E+05 | 4.99E+05 | 3.29E+05 | 1.49E+00 | 6.30E-02 | 2.26E+00 | 1.18E+00 | up |
| Lmgn000242 | C6H10O6 | 4,5,6-Trihydroxy-2-oxohexanoic acid | Organic acids | Organic acids | 2.76E+05 | 1.49E+05 | 2.43E+05 | 7.56E+04 | 9.35E+04 | 7.92E+04 | 1.51E+00 | 6.38E-02 | 3.72E-01 | -1.43E+00 | down |
| MW0155159 | C19H27N5O6 | Phe-Gln-Gln | Amino acids and derivatives | Amino acids and derivatives | 1.32E+04 | 2.78E+04 | 1.93E+04 | 4.88E+03 | 3.95E+03 | 4.33E+03 | 1.54E+00 | 6.45E-02 | 2.18E-01 | -2.19E+00 | down |
| MW0154405 | C29H48N6O19 | Neu5Ac2-6LacDiNAcSp | Others | Saccharides | 3.97E+03 | 1.40E+03 | 2.61E+03 | 9.00E+00 | 9.00E+00 | 9.00E+00 | 1.62E+00 | 7.06E-02 | 3.38E-03 | -8.21E+00 | down |
| pmp001270 | C24H48NO7P | LysoPC 16:1 | Lipids | LPC | 1.26E+05 | 7.34E+04 | 1.25E+05 | 7.89E+04 | 4.90E+04 | 3.20E+04 | 1.29E+00 | 7.19E-02 | 4.93E-01 | -1.02E+00 | down |
| pmb0962 | C10H22N2O4 | L-Lysine-Butanoic Acid | Amino acids and derivatives | Amino acids and derivatives | 3.75E+04 | 2.82E+04 | 3.91E+04 | 1.02E+04 | 5.26E+03 | 2.76E+04 | 1.22E+00 | 7.42E-02 | 4.10E-01 | -1.29E+00 | down |
| Waln011009 | C28H54NO9P | 2-(2,3-dihydroxypropoxy)-3-(((2-(dimethylamino)ethoxy)(hydroxy)phosphoryl)oxy)propyl (11Z,14Z)-octadeca-11,14-dienoate | Lipids | LPE | 2.83E+06 | 1.66E+06 | 2.91E+06 | 1.95E+06 | 5.56E+05 | 4.76E+05 | 1.27E+00 | 8.10E-02 | 4.03E-01 | -1.31E+00 | down |
| pmb2657 | C10H18N4O6 | Argininosuccinic acid | Organic acids | Organic acids | 5.87E+04 | 1.29E+05 | 9.60E+04 | 3.55E+05 | 6.58E+05 | 3.08E+05 | 1.51E+00 | 8.32E-02 | 4.65E+00 | 2.22E+00 | up |
| Waln010743 | C28H54NO9P | 1-(2,3-dihydroxypropoxy)-3-(((2-(dimethylamino)ethoxy)(hydroxy)phosphoryl)oxy)propan-2-yl (11Z,14Z)-octadeca-11,14-dienoate | Lipids | LPE | 2.67E+06 | 1.44E+06 | 2.72E+06 | 1.79E+06 | 5.16E+05 | 4.11E+05 | 1.26E+00 | 8.69E-02 | 3.97E-01 | -1.33E+00 | down |
| MW0110229 | C27H29N3O7 | H-Tyr-tyr-tyr-OH | Amino acids and derivatives | Amino acids and derivatives | 2.36E+04 | 7.99E+03 | 1.59E+04 | 1.88E+03 | 3.10E+03 | 1.10E+03 | 1.51E+00 | 9.00E-02 | 1.28E-01 | -2.97E+00 | down |
| ML10181668 | C6H11NO2 | Cycloleucine | Amino acids and derivatives | Amino acids and derivatives | 2.39E+06 | 3.50E+06 | 3.51E+06 | 5.51E+06 | 1.18E+07 | 8.20E+06 | 1.46E+00 | 9.21E-02 | 2.71E+00 | 1.44E+00 | up |
| MW0144774 | C21H28N4O10 | Ala-Tyr-Glu-Asp | Amino acids and derivatives | Amino acids and derivatives | 4.75E+05 | 4.13E+05 | 1.83E+05 | 6.60E+04 | 7.49E+04 | 2.01E+05 | 1.26E+00 | 9.36E-02 | 3.19E-01 | -1.65E+00 | down |
| mws0126 | C26H54NO7P | LysoPC 18:0 | Lipids | LPC | 1.94E+05 | 2.02E+05 | 2.49E+05 | 6.90E+05 | 5.08E+05 | 3.40E+05 | 1.41E+00 | 9.43E-02 | 2.38E+00 | 1.25E+00 | up |
| pmd0136 | C26H54NO7P | LysoPC 18:0(2n isomer) | Lipids | LPC | 2.21E+05 | 2.01E+05 | 2.50E+05 | 7.31E+05 | 5.29E+05 | 3.62E+05 | 1.42E+00 | 9.49E-02 | 2.41E+00 | 1.27E+00 | up |
| **Index** | **Formula** | **Compounds** | **Class I** | **Class II** | **DW-1-1** | **DW-1-2** | **DW-1-3** | **B21-1-1** | **B21-1-2** | **B21-1-3** | **VIP** | **P-value** | **Fold_Change** | **Log2FC** | **Type** |
| Zmmp000635 | C6H11NO2 | (2S)-2-amino-4-methyl-4-pentenoic acid | Amino acids and derivatives | Amino acids and derivatives | 2.66E+06 | 3.55E+06 | 3.51E+06 | 6.20E+06 | 1.34E+07 | 8.78E+06 | 1.49E+00 | 9.61E-02 | 2.93E+00 | 1.55E+00 | up |
| MW0158726 | C23H27N3O5 | Tyr-Pro-Phe | Amino acids and derivatives | Amino acids and derivatives | 8.88E+03 | 6.46E+03 | 1.62E+04 | 2.45E+03 | 1.24E+03 | 2.21E+03 | 1.52E+00 | 9.82E-02 | 1.87E-01 | -2.42E+00 | down |
| Lmqn000213 | C24H42O21 | Stachyose | Others | Saccharides | 3.49E+06 | 4.11E+06 | 8.43E+06 | 5.69E+05 | 3.29E+05 | 2.82E+06 | 1.28E+00 | 1.01E-01 | 2.32E-01 | -2.11E+00 | down |
| MWS0631 | C3H7NO5S2 | S-Sulfo-L-Cysteine | Amino acids and derivatives | Amino acids and derivatives | 2.17E+04 | 2.02E+04 | 2.81E+04 | 6.24E+04 | 4.04E+04 | 9.05E+04 | 1.42E+00 | 1.01E-01 | 2.76E+00 | 1.46E+00 | up |
| pme0500 | C18H32O16 | D-Melezitose | Others | Saccharides | 1.09E+06 | 1.27E+06 | 2.54E+06 | 2.19E+05 | 3.26E+05 | 7.41E+05 | 1.35E+00 | 1.06E-01 | 2.62E-01 | -1.93E+00 | down |
| MW0155805 | C21H36N6O9 | Pro-Asp-Ala-Lys-Ser | Amino acids and derivatives | Amino acids and derivatives | 2.67E+05 | 1.44E+05 | 1.21E+05 | 6.63E+04 | 4.62E+04 | 5.08E+04 | 1.49E+00 | 1.11E-01 | 3.07E-01 | -1.70E+00 | down |
| MWSmce190 | C6H13NO3 | 4-Hydroxy-L-Isoleucine | Amino acids and derivatives | Amino acids and derivatives | 4.66E+04 | 5.10E+04 | 4.84E+04 | 8.49E+04 | 1.82E+05 | 1.15E+05 | 1.48E+00 | 1.12E-01 | 2.62E+00 | 1.39E+00 | up |
| pme1712 | C11H20N2O6 | L-Saccharopine | Amino acids and derivatives | Amino acids and derivatives | 1.42E+04 | 2.03E+04 | 1.75E+04 | 5.93E+04 | 1.04E+05 | 4.05E+04 | 1.49E+00 | 1.13E-01 | 3.91E+00 | 1.97E+00 | up |
| MW0151069 | C22H26N6O4 | His-Pro-Trp | Amino acids and derivatives | Amino acids and derivatives | 2.59E+04 | 1.56E+04 | 3.27E+04 | 1.59E+04 | 5.35E+03 | 1.51E+04 | 1.15E+00 | 1.13E-01 | 4.89E-01 | -1.03E+00 | down |
| Lmbn005369 | C18H32O3 | 13(S)-HODE;13(S)-Hydroxyoctadeca-9Z,11E-dienoic acid* | Lipids | Free fatty acids | 7.44E+04 | 6.37E+04 | 5.71E+04 | 1.64E+05 | 1.65E+05 | 8.26E+04 | 1.32E+00 | 1.14E-01 | 2.11E+00 | 1.07E+00 | up |
| NK10251888 | C8H18N4O2 | NG,NG-Dimethyl-L-arginine* | Amino acids and derivatives | Amino acids and derivatives | 1.89E+04 | 4.45E+04 | 3.37E+04 | 1.43E+05 | 1.15E+05 | 5.03E+04 | 1.31E+00 | 1.16E-01 | 3.18E+00 | 1.67E+00 | up |
| mws0254 | C6H9N3O2 | L-Histidine | Amino acids and derivatives | Amino acids and derivatives | 4.36E+05 | 6.19E+05 | 4.45E+05 | 1.80E+06 | 1.74E+06 | 7.15E+05 | 1.35E+00 | 1.17E-01 | 2.84E+00 | 1.51E+00 | up |
| Zmdp001857 | C14H18N2O6 | γ-Glutamyltyrosine | Amino acids and derivatives | Amino acids and derivatives | 1.17E+04 | 1.91E+04 | 1.37E+04 | 4.71E+04 | 4.81E+04 | 1.99E+04 | 1.31E+00 | 1.18E-01 | 2.59E+00 | 1.37E+00 | up |
| Zmdp001647 | C10H18N2O5 | γ-Glutamyl-L-valine | Amino acids and derivatives | Amino acids and derivatives | 1.10E+04 | 1.34E+04 | 9.92E+03 | 3.46E+04 | 3.37E+04 | 1.51E+04 | 1.32E+00 | 1.21E-01 | 2.43E+00 | 1.28E+00 | up |
| Wafn011571 | C18H32O3 | alpha-Hydroxylinoleic acid* | Lipids | Free fatty acids | 6.03E+04 | 5.06E+04 | 5.88E+04 | 1.58E+05 | 1.42E+05 | 7.14E+04 | 1.30E+00 | 1.25E-01 | 2.19E+00 | 1.13E+00 | up |
| MW0153757 | C10H18N2O5S | Met-Glu | Amino acids and derivatives | Amino acids and derivatives | 9.89E+04 | 1.13E+05 | 1.31E+05 | 8.79E+05 | 1.36E+06 | 3.37E+05 | 1.47E+00 | 1.28E-01 | 7.52E+00 | 2.91E+00 | up |
| Zmgn001448 | C7H12O5 | 2-Propylmalic Acid | Organic acids | Organic acids | 5.09E+04 | 3.04E+04 | 2.14E+04 | 1.53E+05 | 8.68E+04 | 6.04E+04 | 1.30E+00 | 1.29E-01 | 2.92E+00 | 1.55E+00 | up |
| Zmmp000606 | C6H13NO3 | 2-Amino-4-hydroxy-3-methylpentanoic acid | Amino acids and derivatives | Amino acids and derivatives | 9.25E+06 | 1.71E+07 | 1.44E+07 | 1.84E+07 | 4.18E+07 | 2.87E+07 | 1.32E+00 | 1.29E-01 | 2.18E+00 | 1.13E+00 | up |
| Wayn000597 | C18H28O17 | Citric acid-1-O-diglucoside | Organic acids | Organic acids | 2.08E+04 | 2.35E+04 | 1.96E+04 | 9.89E+04 | 6.20E+04 | 3.71E+04 | 1.40E+00 | 1.30E-01 | 3.10E+00 | 1.63E+00 | up |
| MWS5164 | C8H18N4O2 | N,N'-Dimethylarginine;SDMA* | Amino acids and derivatives | Amino acids and derivatives | 2.36E+04 | 3.89E+04 | 2.52E+04 | 1.51E+05 | 1.49E+05 | 4.44E+04 | 1.33E+00 | 1.33E-01 | 3.93E+00 | 1.98E+00 | up |
|  |  |  |  |  |  |  |  |  |  |  |  |  |  |  |  |
| **Index** | **Formula** | **Compounds** | **Class I** | **Class II** | **DW-1-1** | **DW-1-2** | **DW-1-3** | **B21-1-1** | **B21-1-2** | **B21-1-3** | **VIP** | **P-value** | **Fold_Change** | **Log2FC** | **Type** |
| pme1086 | C10H17N3O6S | Glutathione reduced form | Amino acids and derivatives | Amino acids and derivatives | 3.36E+05 | 6.02E+05 | 4.69E+05 | 1.89E+06 | 1.14E+06 | 7.57E+05 | 1.34E+00 | 1.34E-01 | 2.69E+00 | 1.43E+00 | up |
| Zmpn003368 | C18H30O3 | 13S-Hydroxy-9Z,11E,15Z-octadecatrienoic acid | Lipids | Free fatty acids | 7.22E+03 | 5.24E+03 | 7.93E+03 | 1.88E+04 | 3.94E+04 | 1.60E+04 | 1.45E+00 | 1.35E-01 | 3.63E+00 | 1.86E+00 | up |
| MW0146111 | C20H33N7O11 | Asp-Gly-Asp-Lys-Asn | Amino acids and derivatives | Amino acids and derivatives | 6.63E+04 | 2.41E+04 | 6.55E+04 | 3.99E+04 | 7.99E+03 | 8.43E+03 | 1.19E+00 | 1.36E-01 | 3.61E-01 | -1.47E+00 | down |
| mws0567 | C5H11N3O2 | 4-Guanidinobutyric acid | Organic acids | Organic acids | 5.54E+05 | 5.03E+05 | 4.49E+05 | 3.93E+06 | 5.27E+06 | 1.14E+06 | 1.41E+00 | 1.37E-01 | 6.86E+00 | 2.78E+00 | up |
| Zmdp001663 | C10H18N2O5S | γ-glutamylmethionine | Amino acids and derivatives | Amino acids and derivatives | 5.55E+04 | 4.85E+04 | 6.96E+04 | 3.23E+05 | 5.29E+05 | 1.37E+05 | 1.44E+00 | 1.38E-01 | 5.70E+00 | 2.51E+00 | up |
| Zmyn000247 | C5H8O5 | 2-Hydroxyglutaric Acid* | Organic acids | Organic acids | 9.58E+04 | 1.62E+05 | 1.10E+05 | 6.88E+05 | 3.89E+05 | 2.32E+05 | 1.39E+00 | 1.40E-01 | 3.57E+00 | 1.83E+00 | up |
| pmb2804 | C18H32O4 | 13S-Hydroperoxy-9Z,11E-octadecadienoic acid | Lipids | Free fatty acids | 9.85E+03 | 5.79E+03 | 9.90E+03 | 4.34E+04 | 2.86E+04 | 1.35E+04 | 1.30E+00 | 1.44E-01 | 3.35E+00 | 1.74E+00 | up |
| pmb0296 | C21H40O4 | 1-Oleoyl-Sn-Glycerol | Lipids | Glycerol ester | 2.66E+05 | 2.91E+05 | 5.43E+05 | 2.16E+05 | 5.81E+04 | 2.36E+05 | 1.10E+00 | 1.46E-01 | 4.63E-01 | -1.11E+00 | down |
| Zmzp000145 | C9H20N2O2 | Trimethyllysine | Amino acids and derivatives | Amino acids and derivatives | 3.25E+04 | 1.14E+05 | 3.84E+04 | 5.17E+05 | 6.27E+05 | 1.10E+05 | 1.32E+00 | 1.48E-01 | 6.80E+00 | 2.76E+00 | up |
| MW0152218 | C31H50N8O9 | Leu-Arg-Asp-Leu-Tyr | Amino acids and derivatives | Amino acids and derivatives | 1.39E+04 | 5.96E+03 | 6.16E+03 | 4.61E+03 | 7.16E+02 | 3.67E+03 | 1.16E+00 | 1.49E-01 | 3.46E-01 | -1.53E+00 | down |
| Zmgn000173 | C5H10O5 | D-Ribose | Others | Saccharides | 1.38E+04 | 2.01E+04 | 1.66E+04 | 5.33E+04 | 4.31E+04 | 2.00E+04 | 1.24E+00 | 1.52E-01 | 2.31E+00 | 1.20E+00 | up |
| pme0014 | C5H9NO4 | L-Glutamic acid | Amino acids and derivatives | Amino acids and derivatives | 1.40E+07 | 2.47E+07 | 2.41E+07 | 2.78E+07 | 6.88E+07 | 4.43E+07 | 1.29E+00 | 1.53E-01 | 2.24E+00 | 1.16E+00 | up |
| Lmhp009384 | C33H58O14 | 1-Linoleoylglycerol-2,3-di-O-glucoside* | Lipids | Glycerol ester | 4.64E+03 | 4.60E+03 | 7.62E+03 | 5.22E+03 | 7.86E+02 | 1.86E+03 | 1.12E+00 | 1.53E-01 | 4.67E-01 | -1.10E+00 | down |
| Zmmp002167 | C14H18N2O6 | 2-((L-tyrosyl)oxy)-5-amino-5-oxopentanoic acid | Amino acids and derivatives | Amino acids and derivatives | 1.46E+04 | 1.49E+04 | 9.33E+03 | 5.23E+04 | 4.19E+04 | 1.60E+04 | 1.23E+00 | 1.55E-01 | 2.84E+00 | 1.51E+00 | up |
| pmb3101 | C7H12O5 | 2-Isopropylmalic Acid | Organic acids | Organic acids | 4.22E+04 | 3.20E+04 | 2.15E+04 | 1.66E+05 | 8.90E+04 | 5.52E+04 | 1.32E+00 | 1.58E-01 | 3.25E+00 | 1.70E+00 | up |
| MWS00330g | C5H9NO2 | 1-Amino-1-cyclobutane-carboxylic-acid | Amino acids and derivatives | Amino acids and derivatives | 5.63E+05 | 7.67E+05 | 7.49E+05 | 3.03E+06 | 4.62E+06 | 1.12E+06 | 1.35E+00 | 1.58E-01 | 4.22E+00 | 2.08E+00 | up |
| MW0110297 | C14H20N2O4 | Tyrosyl-Valine | Amino acids and derivatives | Amino acids and derivatives | 6.36E+05 | 5.51E+05 | 6.68E+05 | 3.16E+06 | 2.17E+06 | 8.71E+05 | 1.28E+00 | 1.60E-01 | 3.35E+00 | 1.74E+00 | up |
| pmb2591 | C13H14N2O3 | N-Acetyl-L-Tryptophan | Amino acids and derivatives | Amino acids and derivatives | 4.92E+04 | 4.66E+04 | 5.37E+04 | 1.19E+06 | 1.83E+06 | 2.12E+05 | 1.45E+00 | 1.61E-01 | 2.16E+01 | 4.43E+00 | up |
| MW0158390 | C23H34N6O9 | Tyr-Asn-Asp-Lys | Amino acids and derivatives | Amino acids and derivatives | 1.07E+05 | 6.05E+04 | 4.83E+04 | 4.33E+04 | 2.32E+04 | 3.68E+04 | 1.26E+00 | 1.62E-01 | 4.79E-01 | -1.06E+00 | down |
| Zmdp000292 | C7H16N4O2 | Arginine methyl ester* | Amino acids and derivatives | Amino acids and derivatives | 2.82E+04 | 5.73E+04 | 2.54E+04 | 1.03E+06 | 1.43E+06 | 1.25E+05 | 1.40E+00 | 1.66E-01 | 2.33E+01 | 4.54E+00 | up |
| MW0110084 | C22H22N4O3 | Trp-Trp | Amino acids and derivatives | Amino acids and derivatives | 4.00E+05 | 1.60E+05 | 1.81E+05 | 1.36E+05 | 4.50E+04 | 8.36E+04 | 1.29E+00 | 1.66E-01 | 3.58E-01 | -1.48E+00 | down |
| **Index** | **Formula** | **Compounds** | **Class I** | **Class II** | **DW-1-1** | **DW-1-2** | **DW-1-3** | **B21-1-1** | **B21-1-2** | **B21-1-3** | **VIP** | **P-value** | **Fold_Change** | **Log2FC** | **Type** |
| Zmjp000182 | C7H16N4O2 | N-Monomethyl-L-arginine* | Amino acids and derivatives | Amino acids and derivatives | 2.56E+04 | 5.27E+04 | 3.44E+04 | 8.51E+05 | 1.11E+06 | 9.68E+04 | 1.36E+00 | 1.66E-01 | 1.82E+01 | 4.19E+00 | up |
| mws0256 | C5H11NO2 | L-Valine | Amino acids and derivatives | Amino acids and derivatives | 4.90E+05 | 6.91E+05 | 5.31E+05 | 2.41E+06 | 1.31E+06 | 8.72E+05 | 1.32E+00 | 1.68E-01 | 2.68E+00 | 1.42E+00 | up |
| MW0105852 | C12H20N4O8 | Asp-Gln-Ser | Amino acids and derivatives | Amino acids and derivatives | 8.19E+04 | 2.53E+05 | 1.38E+05 | 3.07E+06 | 4.63E+06 | 4.03E+05 | 1.36E+00 | 1.75E-01 | 1.72E+01 | 4.10E+00 | up |
| mws0281 | C6H8O7 | Citric Acid | Organic acids | Organic acids | 1.51E+06 | 2.42E+06 | 1.38E+06 | 9.31E+06 | 5.31E+06 | 2.58E+06 | 1.28E+00 | 1.76E-01 | 3.24E+00 | 1.70E+00 | up |
| MWS5209 | C5H11NO2 | N-Methyl-α-aminoisobutyric acid | Amino acids and derivatives | Amino acids and derivatives | 4.12E+04 | 3.95E+04 | 4.15E+04 | 9.67E+03 | 6.68E+03 | 4.01E+04 | 1.13E+00 | 1.76E-01 | 4.62E-01 | -1.11E+00 | down |
| MW0151136 | C19H25N5O6 | His-Tyr-Thr | Amino acids and derivatives | Amino acids and derivatives | 4.88E+04 | 4.85E+04 | 5.15E+04 | 1.44E+05 | 1.09E+05 | 5.40E+04 | 1.17E+00 | 1.81E-01 | 2.06E+00 | 1.04E+00 | up |
| MWS00411g | C6H11NO4 | L-2-Aminoadipate | Amino acids and derivatives | Amino acids and derivatives | 7.80E+04 | 9.22E+04 | 7.60E+04 | 1.23E+05 | 3.12E+05 | 1.59E+05 | 1.36E+00 | 1.82E-01 | 2.41E+00 | 1.27E+00 | up |
| MW0107005 | C14H19N3O5 | Gln-Tyr | Amino acids and derivatives | Amino acids and derivatives | 3.93E+06 | 1.97E+06 | 9.33E+05 | 1.07E+07 | 9.80E+06 | 2.15E+06 | 1.04E+00 | 1.83E-01 | 3.32E+00 | 1.73E+00 | up |
| pmb2826 | C5H8O5 | L-Citramalic acid | Organic acids | Organic acids | 1.14E+05 | 1.88E+05 | 1.12E+05 | 6.35E+05 | 3.27E+05 | 2.04E+05 | 1.27E+00 | 1.85E-01 | 2.82E+00 | 1.50E+00 | up |
| Lmyn006221 | C33H60O14 | Gingerglycolipid C | Lipids | Glycerol ester | 2.43E+05 | 2.01E+05 | 4.45E+05 | 2.04E+05 | 4.51E+04 | 1.93E+05 | 1.02E+00 | 1.87E-01 | 4.97E-01 | -1.01E+00 | down |
| MW0145782 | C20H36N8O8 | Asn-Ile-Arg-Asp | Amino acids and derivatives | Amino acids and derivatives | 7.46E+05 | 4.08E+05 | 2.76E+05 | 2.35E+05 | 1.66E+05 | 2.09E+05 | 1.29E+00 | 1.88E-01 | 4.27E-01 | -1.23E+00 | down |
| mws0282 | C11H12N2O2 | L-Tryptophan | Amino acids and derivatives | Amino acids and derivatives | 1.93E+06 | 2.11E+06 | 1.66E+06 | 1.01E+07 | 7.30E+06 | 2.11E+06 | 1.17E+00 | 1.88E-01 | 3.43E+00 | 1.78E+00 | up |
| MW0145775 | C23H29N7O8 | Asn-His-Phe-Asp | Amino acids and derivatives | Amino acids and derivatives | 1.47E+04 | 6.04E+03 | 1.77E+04 | 4.27E+03 | 3.80E+03 | 1.04E+04 | 1.04E+00 | 1.94E-01 | 4.80E-01 | -1.06E+00 | down |
| MW0153514 | C14H23N3O8S1 | Met-Asp-Glu | Amino acids and derivatives | Amino acids and derivatives | 1.08E+05 | 8.26E+04 | 3.13E+04 | 3.14E+04 | 4.04E+04 | 2.25E+04 | 1.09E+00 | 1.95E-01 | 4.25E-01 | -1.24E+00 | down |
| Zmmp002946 | C14H18N2O5 | (3-(carboxyamino)-2-methylpropanoyl)phenylalanine | Amino acids and derivatives | Amino acids and derivatives | 1.16E+04 | 1.77E+04 | 1.77E+04 | 7.73E+04 | 5.02E+04 | 1.74E+04 | 1.15E+00 | 1.99E-01 | 3.08E+00 | 1.62E+00 | up |
| Zmpn000095 | C6H15O9P | Sorbitol-6-phosphate | Others | Saccharides | 3.96E+05 | 2.93E+05 | 6.53E+05 | 4.21E+05 | 1.25E+06 | 1.58E+06 | 1.07E+00 | 2.00E-01 | 2.42E+00 | 1.28E+00 | up |
| pmf0096 | C2H2O4 | Oxalic acid | Organic acids | Organic acids | 5.70E+04 | 4.56E+04 | 5.47E+04 | 1.73E+05 | 6.49E+04 | 9.56E+04 | 1.17E+00 | 2.09E-01 | 2.12E+00 | 1.08E+00 | up |
| Zazp002547 | C11H12N2O2 | cyclo-(Gly-Phe) | Amino acids and derivatives | Amino acids and derivatives | 5.53E+05 | 6.99E+05 | 6.00E+05 | 2.33E+06 | 1.88E+06 | 5.36E+05 | 1.02E+00 | 2.15E-01 | 2.56E+00 | 1.36E+00 | up |
| Zmdn001564 | C14H18N2O5 | γ-Glutamylphenylalanine | Amino acids and derivatives | Amino acids and derivatives | 1.61E+04 | 2.33E+04 | 1.79E+04 | 1.20E+05 | 6.29E+04 | 2.39E+04 | 1.19E+00 | 2.16E-01 | 3.60E+00 | 1.85E+00 | up |
| Zmyn004732 | C18H30O3 | 2R-hydroxy-9Z,12Z,15Z-octadecatrienoic acid | Lipids | Free fatty acids | 5.72E+03 | 5.21E+03 | 8.67E+03 | 2.51E+04 | 1.27E+04 | 8.19E+03 | 1.13E+00 | 2.21E-01 | 2.35E+00 | 1.23E+00 | up |
| MWSmce119 | C6H14N4O2 | L-Arginine | Amino acids and derivatives | Amino acids and derivatives | 2.72E+06 | 3.95E+06 | 3.76E+06 | 6.93E+07 | 1.42E+08 | 4.97E+06 | 1.22E+00 | 2.25E-01 | 2.08E+01 | 4.38E+00 | up |
| **Index** | **Formula** | **Compounds** | **Class I** | **Class II** | **DW-1-1** | **DW-1-2** | **DW-1-3** | **B21-1-1** | **B21-1-2** | **B21-1-3** | **VIP** | **P-value** | **Fold_Change** | **Log2FC** | **Type** |
| pme3163 | C7H15O10P | D-Sedoheptuiose 7-phosphate | Others | Saccharides | 6.00E+06 | 1.05E+07 | 1.12E+07 | 8.22E+06 | 2.81E+07 | 4.38E+07 | 1.08E+00 | 2.29E-01 | 2.90E+00 | 1.53E+00 | up |
| MW0158648 | C23H28N4O7 | Tyr-Tyr-Gln | Amino acids and derivatives | Amino acids and derivatives | 3.15E+04 | 1.05E+04 | 4.23E+04 | 1.30E+04 | 1.51E+04 | 9.34E+03 | 1.03E+00 | 2.33E-01 | 4.44E-01 | -1.17E+00 | down |
| pme2527 | C5H12N2O2 | L-Ornithine | Amino acids and derivatives | Amino acids and derivatives | 3.55E+04 | 3.44E+04 | 3.27E+04 | 4.39E+04 | 1.17E+05 | 5.54E+04 | 1.23E+00 | 2.37E-01 | 2.11E+00 | 1.08E+00 | up |
| pme1987 | C3H7NO2 | L-Alanine | Amino acids and derivatives | Amino acids and derivatives | 5.82E+03 | 5.57E+03 | 2.36E+03 | 7.59E+03 | 1.88E+04 | 6.62E+03 | 1.14E+00 | 2.38E-01 | 2.40E+00 | 1.27E+00 | up |
| mws0425 | C5H6O4 | Citraconic acid | Organic acids | Organic acids | 6.41E+05 | 7.13E+05 | 5.46E+05 | 2.38E+06 | 1.12E+06 | 7.95E+05 | 1.15E+00 | 2.40E-01 | 2.26E+00 | 1.18E+00 | up |
| MWS04412 | C8H18N2O2 | N(6),N(6)-Dimethyl-L-lysine | Amino acids and derivatives | Amino acids and derivatives | 2.88E+06 | 4.13E+06 | 4.23E+06 | 7.03E+07 | 1.74E+08 | 6.39E+06 | 1.25E+00 | 2.44E-01 | 2.23E+01 | 4.48E+00 | up |
| MWS04447 | C6H11NO2 | Homoproline | Amino acids and derivatives | Amino acids and derivatives | 5.95E+04 | 4.95E+04 | 7.35E+04 | 3.52E+05 | 7.95E+05 | 6.70E+04 | 1.14E+00 | 2.46E-01 | 6.65E+00 | 2.73E+00 | up |
| Zmyn000155 | C7H14N2O3 | N-α-Acetyl-L-ornithine | Amino acids and derivatives | Amino acids and derivatives | 2.10E+05 | 3.96E+05 | 3.30E+05 | 1.91E+07 | 4.90E+07 | 9.22E+05 | 1.32E+00 | 2.47E-01 | 7.38E+01 | 6.21E+00 | up |
| MW0107902 | C9H18N2O3 | L-Leucyl-L-alanine | Amino acids and derivatives | Amino acids and derivatives | 1.14E+04 | 1.36E+05 | 3.08E+05 | 1.53E+04 | 7.43E+03 | 1.67E+04 | 1.07E+00 | 2.48E-01 | 8.65E-02 | -3.53E+00 | down |
| MW0157568 | C10H18N4O6 | Thr-Asn-Gly | Amino acids and derivatives | Amino acids and derivatives | 1.54E+04 | 7.89E+04 | 2.58E+04 | 4.49E+06 | 1.73E+06 | 8.86E+04 | 1.28E+00 | 2.50E-01 | 5.25E+01 | 5.71E+00 | up |
| mws1587 | C6H13NO2 | L-Norleucine | Amino acids and derivatives | Amino acids and derivatives | 8.53E+05 | 1.40E+06 | 1.07E+06 | 6.55E+06 | 2.56E+06 | 1.50E+06 | 1.17E+00 | 2.54E-01 | 3.19E+00 | 1.67E+00 | up |
| MW0151126 | C24H31N7O9 | His-Tyr-Gln-Asp | Amino acids and derivatives | Amino acids and derivatives | 1.59E+04 | 1.72E+04 | 1.03E+04 | 3.05E+04 | 1.34E+04 | 5.36E+04 | 1.00E+00 | 2.60E-01 | 2.24E+00 | 1.17E+00 | up |
| MWS04555g | C6H13NO2 | D-Allo-Isoleucine* | Amino acids and derivatives | Amino acids and derivatives | 1.01E+06 | 1.35E+06 | 1.15E+06 | 6.94E+06 | 3.23E+06 | 1.19E+06 | 1.06E+00 | 2.60E-01 | 3.23E+00 | 1.69E+00 | up |
| MWS4471 | C6H10N2O4 | N-Alpha-Acetyl-L-Asparagine | Amino acids and derivatives | Amino acids and derivatives | 1.57E+03 | 6.81E+03 | 4.45E+03 | 2.95E+05 | 8.91E+05 | 1.88E+04 | 1.37E+00 | 2.63E-01 | 9.40E+01 | 6.55E+00 | up |
| pme0008 | C6H13N3O3 | L-Citrulline | Amino acids and derivatives | Amino acids and derivatives | 1.43E+06 | 1.63E+06 | 2.04E+06 | 3.10E+06 | 1.15E+07 | 3.12E+06 | 1.25E+00 | 2.71E-01 | 3.47E+00 | 1.79E+00 | up |
| Wayn000504 | C16H26O15 | Malic acid-1-O-diglucoside | Organic acids | Organic acids | 2.05E+04 | 2.47E+04 | 2.78E+04 | 9.15E+04 | 3.08E+04 | 3.68E+04 | 1.07E+00 | 2.75E-01 | 2.18E+00 | 1.12E+00 | up |
| Lmyn002403 | C14H18O8 | Mandelic acid-β-glucoside | Organic acids | Organic acids | 3.65E+05 | 1.12E+05 | 9.77E+04 | 9.04E+04 | 7.46E+04 | 5.31E+04 | 1.12E+00 | 3.03E-01 | 3.79E-01 | -1.40E+00 | down |
| Wmzn000227 | C6H10O4 | 2,2-Dimethylsuccinic acid | Organic acids | Organic acids | 4.53E+04 | 4.96E+04 | 5.80E+04 | 6.17E+04 | 2.16E+05 | 7.71E+04 | 1.10E+00 | 3.04E-01 | 2.32E+00 | 1.21E+00 | up |
| pme0274 | C6H13NO2 | 6-Aminocaproic acid | Organic acids | Organic acids | 2.48E+05 | 4.04E+05 | 2.93E+05 | 1.85E+06 | 6.73E+05 | 3.20E+05 | 1.00E+00 | 3.04E-01 | 3.01E+00 | 1.59E+00 | up |
| MW0158444 | C24H38N6O8 | Tyr-Gln-Thr-Lys | Amino acids and derivatives | Amino acids and derivatives | 8.90E+05 | 2.08E+05 | 6.04E+04 | 2.46E+04 | 3.20E+04 | 6.37E+04 | 1.19E+00 | 3.08E-01 | 1.04E-01 | -3.27E+00 | down |

| **Supplementary Table S4. Metabolites were identified in YZ and B21** | | | | | | | | | | | | | | | |
| --- | --- | --- | --- | --- | --- | --- | --- | --- | --- | --- | --- | --- | --- | --- | --- |
| **Index** | **Formula** | **Compounds** | **Class I** | **Class II** | **YZ-1-1** | **YZ-1-2** | **YZ-1-3** | **B21-1-1** | **B21-1-2** | **B21-1-3** | **VIP** | **P-value** | **Fold_Change** | **Log2FC** | **Type** |
| MW0144205 | C15H20N2O6 | Abu-Ile-OH | Amino acids and derivatives | Amino acids and derivatives | 7.08E+05 | 9.08E+05 | 1.47E+06 | 4.40E+05 | 3.26E+04 | 6.54E+05 | 1.07E+00 | 9.20E-02 | 3.65E-01 | -1.45E+00 | down |
| ML10181668 | C6H11NO2 | Cycloleucine | Amino acids and derivatives | Amino acids and derivatives | 5.01E+06 | 3.70E+06 | 3.04E+06 | 5.51E+06 | 1.18E+07 | 8.20E+06 | 1.37E+00 | 1.16E-01 | 2.17E+00 | 1.12E+00 | up |
| Zmdn001564 | C14H18N2O5 | γ-Glutamylphenylalanine | Amino acids and derivatives | Amino acids and derivatives | 2.82E+04 | 1.39E+04 | 1.36E+04 | 1.20E+05 | 6.29E+04 | 2.39E+04 | 1.17E+00 | 2.10E-01 | 3.71E+00 | 1.89E+00 | up |
| pme0075 | C7H11NO5 | N-Acetyl-L-glutamic acid | Amino acids and derivatives | Amino acids and derivatives | 1.22E+06 | 1.51E+06 | 1.08E+06 | 3.91E+06 | 3.51E+06 | 2.61E+06 | 1.51E+00 | 2.37E-02 | 2.64E+00 | 1.40E+00 | up |
| MW0110295 | C20H21N3O4 | Tyrosyl-tryptophan | Amino acids and derivatives | Amino acids and derivatives | 1.70E+05 | 1.45E+05 | 1.68E+05 | 9.64E+05 | 1.83E+05 | 4.30E+05 | 1.05E+00 | 2.54E-01 | 3.26E+00 | 1.71E+00 | up |
| MW0151136 | C19H25N5O6 | His-Tyr-Thr | Amino acids and derivatives | Amino acids and derivatives | 4.10E+04 | 6.47E+04 | 4.02E+04 | 1.44E+05 | 1.09E+05 | 5.40E+04 | 1.12E+00 | 1.68E-01 | 2.11E+00 | 1.07E+00 | up |
| MW0153708 | C23H29N3O6S1 | Met-Tyr-Tyr | Amino acids and derivatives | Amino acids and derivatives | 2.87E+05 | 2.72E+05 | 2.30E+05 | 4.46E+04 | 1.96E+04 | 8.65E+04 | 1.47E+00 | 1.29E-03 | 1.91E-01 | -2.39E+00 | down |
| MWS00330g | C5H9NO2 | 1-Amino-1-cyclobutane-carboxylic-acid | Amino acids and derivatives | Amino acids and derivatives | 1.33E+06 | 1.10E+06 | 8.90E+05 | 3.03E+06 | 4.62E+06 | 1.12E+06 | 1.12E+00 | 2.13E-01 | 2.64E+00 | 1.40E+00 | up |
| pmb2591 | C13H14N2O3 | N-Acetyl-L-Tryptophan | Amino acids and derivatives | Amino acids and derivatives | 4.43E+04 | 6.58E+04 | 5.16E+04 | 1.19E+06 | 1.83E+06 | 2.12E+05 | 1.44E+00 | 1.62E-01 | 1.99E+01 | 4.32E+00 | up |
| MWS2413 | C5H9NO3 | N-acetyl-beta-alanine | Amino acids and derivatives | Amino acids and derivatives | 3.49E+04 | 4.24E+04 | 3.83E+04 | 1.37E+04 | 2.25E+04 | 1.13E+04 | 1.42E+00 | 8.10E-03 | 4.11E-01 | -1.28E+00 | down |
| MW0151026 | C16H25N5O6S1 | His-Met-Glu | Amino acids and derivatives | Amino acids and derivatives | 3.89E+06 | 2.96E+06 | 4.19E+06 | 1.78E+06 | 4.04E+05 | 8.16E+05 | 1.43E+00 | 8.45E-03 | 2.72E-01 | -1.88E+00 | down |
| MW0151662 | C15H20N2O6 | Ile-Abu-OH | Amino acids and derivatives | Amino acids and derivatives | 4.36E+05 | 1.31E+03 | 8.76E+05 | 2.31E+04 | 6.08E+02 | 9.00E+00 | 1.04E+00 | 2.31E-01 | 1.81E-02 | -5.79E+00 | down |
| MW0108646 | C7H11NO3 | N-Acetylproline | Amino acids and derivatives | Amino acids and derivatives | 4.55E+04 | 3.01E+04 | 3.50E+04 | 5.17E+04 | 1.31E+05 | 6.92E+04 | 1.33E+00 | 1.85E-01 | 2.27E+00 | 1.19E+00 | up |
| MW0146157 | C18H30N4O10 | Asp-Ile-Ser-Glu | Amino acids and derivatives | Amino acids and derivatives | 6.55E+05 | 2.14E+06 | 1.43E+06 | 7.72E+05 | 5.46E+05 | 2.06E+05 | 1.16E+00 | 1.60E-01 | 3.61E-01 | -1.47E+00 | down |
| Zmjp000182 | C7H16N4O2 | N-Monomethyl-L-arginine* | Amino acids and derivatives | Amino acids and derivatives | 1.59E+05 | 7.51E+04 | 5.74E+04 | 8.51E+05 | 1.11E+06 | 9.68E+04 | 1.13E+00 | 1.91E-01 | 7.04E+00 | 2.82E+00 | up |
| MW0110084 | C22H22N4O3 | Trp-Trp | Amino acids and derivatives | Amino acids and derivatives | 2.41E+05 | 2.04E+05 | 1.68E+05 | 1.36E+05 | 4.50E+04 | 8.36E+04 | 1.36E+00 | 2.85E-02 | 4.31E-01 | -1.21E+00 | down |
| MWS04412 | C8H18N2O2 | N(6),N(6)-Dimethyl-L-lysine | Amino acids and derivatives | Amino acids and derivatives | 1.17E+07 | 6.57E+06 | 6.56E+06 | 7.03E+07 | 1.74E+08 | 6.39E+06 | 1.05E+00 | 2.63E-01 | 1.01E+01 | 3.34E+00 | up |
| MW0107902 | C9H18N2O3 | L-Leucyl-L-alanine | Amino acids and derivatives | Amino acids and derivatives | 1.65E+05 | 2.84E+04 | 1.33E+05 | 1.53E+04 | 7.43E+03 | 1.67E+04 | 1.36E+00 | 1.45E-01 | 1.21E-01 | -3.05E+00 | down |
| MWS04447 | C6H11NO2 | Homoproline | Amino acids and derivatives | Amino acids and derivatives | 6.36E+04 | 7.31E+04 | 5.14E+04 | 3.52E+05 | 7.95E+05 | 6.70E+04 | 1.15E+00 | 2.48E-01 | 6.45E+00 | 2.69E+00 | up |
| **Index** | **Formula** | **Compounds** | **Class I** | **Class II** | **YZ-1-1** | **YZ-1-2** | **YZ-1-3** | **B21-1-1** | **B21-1-2** | **B21-1-3** | **VIP** | **P-value** | **Fold_Change** | **Log2FC** | **Type** |
| Zmmp000635 | C6H11NO2 | (2S)-2-amino-4-methyl-4-pentenoic acid | Amino acids and derivatives | Amino acids and derivatives | 4.99E+06 | 3.73E+06 | 3.16E+06 | 6.20E+06 | 1.34E+07 | 8.78E+06 | 1.42E+00 | 1.14E-01 | 2.39E+00 | 1.26E+00 | up |
| mws1051 | C15H23N6O5S+ | S-Adenosylmethionine | Amino acids and derivatives | Amino acids and derivatives | 2.49E+05 | 2.40E+05 | 2.61E+05 | 7.61E+05 | 4.83E+05 | 4.45E+05 | 1.43E+00 | 8.74E-02 | 2.26E+00 | 1.17E+00 | up |
| MW0151126 | C24H31N7O9 | His-Tyr-Gln-Asp | Amino acids and derivatives | Amino acids and derivatives | 9.92E+03 | 1.21E+04 | 1.02E+04 | 3.05E+04 | 1.34E+04 | 5.36E+04 | 1.17E+00 | 2.03E-01 | 3.02E+00 | 1.59E+00 | up |
| Zmdp001663 | C10H18N2O5S | γ-glutamylmethionine | Amino acids and derivatives | Amino acids and derivatives | 6.56E+04 | 3.03E+04 | 3.04E+04 | 3.23E+05 | 5.29E+05 | 1.37E+05 | 1.47E+00 | 1.25E-01 | 7.83E+00 | 2.97E+00 | up |
| MWS0813 | C5H7NO3 | 5-Oxoproline | Amino acids and derivatives | Amino acids and derivatives | 2.68E+04 | 3.26E+04 | 2.49E+04 | 5.67E+04 | 7.50E+04 | 5.38E+04 | 1.54E+00 | 2.62E-02 | 2.20E+00 | 1.14E+00 | up |
| MW0153883 | C20H22N2O7S | Met-TyrMe-OH | Amino acids and derivatives | Amino acids and derivatives | 6.07E+05 | 6.86E+05 | 4.86E+05 | 6.44E+04 | 4.75E+04 | 4.39E+04 | 1.60E+00 | 1.07E-02 | 8.76E-02 | -3.51E+00 | down |
| MW0158444 | C24H38N6O8 | Tyr-Gln-Thr-Lys | Amino acids and derivatives | Amino acids and derivatives | 4.51E+04 | 1.57E+05 | 1.53E+05 | 2.46E+04 | 3.20E+04 | 6.37E+04 | 1.14E+00 | 1.57E-01 | 3.39E-01 | -1.56E+00 | down |
| Zmyn000155 | C7H14N2O3 | N-α-Acetyl-L-ornithine | Amino acids and derivatives | Amino acids and derivatives | 2.34E+06 | 8.86E+05 | 5.60E+05 | 1.91E+07 | 4.90E+07 | 9.22E+05 | 1.07E+00 | 2.61E-01 | 1.82E+01 | 4.19E+00 | up |
| Zmdp000292 | C7H16N4O2 | Arginine methyl ester* | Amino acids and derivatives | Amino acids and derivatives | 1.88E+05 | 9.01E+04 | 9.24E+04 | 1.03E+06 | 1.43E+06 | 1.25E+05 | 1.14E+00 | 1.95E-01 | 6.96E+00 | 2.80E+00 | up |
| pme2074 | C18H29NO4 | Jasmonoyl-L-Isoleucine | Amino acids and derivatives | Amino acids and derivatives | 2.78E+03 | 3.84E+03 | 2.66E+03 | 2.73E+04 | 2.95E+04 | 2.85E+04 | 1.59E+00 | 3.03E-05 | 9.19E+00 | 3.20E+00 | up |
| mws1587 | C6H13NO2 | L-Norleucine | Amino acids and derivatives | Amino acids and derivatives | 1.10E+06 | 1.04E+06 | 1.12E+06 | 6.55E+06 | 2.56E+06 | 1.50E+06 | 1.16E+00 | 2.52E-01 | 3.25E+00 | 1.70E+00 | up |
| MWS4471 | C6H10N2O4 | N-Alpha-Acetyl-L-Asparagine | Amino acids and derivatives | Amino acids and derivatives | 2.99E+04 | 5.02E+03 | 1.10E+04 | 2.95E+05 | 8.91E+05 | 1.88E+04 | 1.19E+00 | 2.72E-01 | 2.62E+01 | 4.71E+00 | up |
| pme1086 | C10H17N3O6S | Glutathione reduced form | Amino acids and derivatives | Amino acids and derivatives | 6.35E+05 | 5.53E+05 | 4.13E+05 | 1.89E+06 | 1.14E+06 | 7.57E+05 | 1.26E+00 | 1.56E-01 | 2.37E+00 | 1.24E+00 | up |
| Zmmp000606 | C6H13NO3 | 2-Amino-4-hydroxy-3-methylpentanoic acid | Amino acids and derivatives | Amino acids and derivatives | 1.88E+07 | 1.22E+07 | 1.09E+07 | 1.84E+07 | 4.18E+07 | 2.87E+07 | 1.32E+00 | 1.34E-01 | 2.13E+00 | 1.09E+00 | up |
| MW0158711 | C19H20N2O8 | TyrMe-Ser-OH | Amino acids and derivatives | Amino acids and derivatives | 2.66E+05 | 7.00E+04 | 1.22E+06 | 1.66E+04 | 1.87E+04 | 4.21E+03 | 1.37E+00 | 2.91E-01 | 2.55E-02 | -5.29E+00 | down |
| MW0153543 | C16H25N5O6S1 | Met-Glu-His | Amino acids and derivatives | Amino acids and derivatives | 2.56E+06 | 2.84E+06 | 3.20E+06 | 1.35E+06 | 3.19E+05 | 6.42E+05 | 1.45E+00 | 7.63E-03 | 2.69E-01 | -1.89E+00 | down |
| MW0144208 | C14H18N2O6S | Abu-Met-OH | Amino acids and derivatives | Amino acids and derivatives | 6.05E+05 | 7.11E+05 | 5.48E+05 | 2.59E+06 | 7.97E+05 | 1.22E+06 | 1.11E+00 | 2.32E-01 | 2.47E+00 | 1.31E+00 | up |
| MW0156024 | C18H24N4O6 | Pro-Tyr-Asn | Amino acids and derivatives | Amino acids and derivatives | 3.04E+03 | 1.54E+04 | 1.73E+04 | 8.31E+03 | 9.08E+02 | 2.00E+03 | 1.06E+00 | 2.03E-01 | 3.14E-01 | -1.67E+00 | down |
| MWS04555g | C6H13NO2 | D-Allo-Isoleucine* | Amino acids and derivatives | Amino acids and derivatives | 1.29E+06 | 1.13E+06 | 1.26E+06 | 6.94E+06 | 3.23E+06 | 1.19E+06 | 1.01E+00 | 2.68E-01 | 3.08E+00 | 1.62E+00 | up |
| MW0149687 | C22H35N7O13 | Gln-Glu-Ser-Gln-Asp | Amino acids and derivatives | Amino acids and derivatives | 3.99E+04 | 1.64E+05 | 4.94E+04 | 4.56E+04 | 3.56E+03 | 1.01E+04 | 1.23E+00 | 2.41E-01 | 2.34E-01 | -2.09E+00 | down |
| **Index** | **Formula** | **Compounds** | **Class I** | **Class II** | **YZ-1-1** | **YZ-1-2** | **YZ-1-3** | **B21-1-1** | **B21-1-2** | **B21-1-3** | **VIP** | **P-value** | **Fold_Change** | **Log2FC** | **Type** |
| mws0256 | C5H11NO2 | L-Valine | Amino acids and derivatives | Amino acids and derivatives | 8.29E+05 | 6.63E+05 | 5.62E+05 | 2.41E+06 | 1.31E+06 | 8.72E+05 | 1.17E+00 | 2.02E-01 | 2.24E+00 | 1.16E+00 | up |
| MW0105852 | C12H20N4O8 | Asp-Gln-Ser | Amino acids and derivatives | Amino acids and derivatives | 2.81E+05 | 1.16E+05 | 4.51E+05 | 3.07E+06 | 4.63E+06 | 4.03E+05 | 1.23E+00 | 1.88E-01 | 9.55E+00 | 3.26E+00 | up |
| MW0109886 | C9H18N2O4S | Thr-Met | Amino acids and derivatives | Amino acids and derivatives | 5.65E+05 | 7.23E+05 | 5.02E+05 | 1.36E+05 | 1.93E+04 | 2.08E+05 | 1.34E+00 | 5.68E-03 | 2.03E-01 | -2.30E+00 | down |
| MW0110297 | C14H20N2O4 | Tyrosyl-Valine | Amino acids and derivatives | Amino acids and derivatives | 5.61E+05 | 5.37E+05 | 4.86E+05 | 3.16E+06 | 2.17E+06 | 8.71E+05 | 1.34E+00 | 1.46E-01 | 3.92E+00 | 1.97E+00 | up |
| MW0108101 | C12H26N4O3 | Lys-Lys | Amino acids and derivatives | Amino acids and derivatives | 1.83E+05 | 3.99E+05 | 1.43E+05 | 1.23E+05 | 1.07E+05 | 1.00E+05 | 1.21E+00 | 2.39E-01 | 4.55E-01 | -1.14E+00 | down |
| MW0148009 | C19H33N5O8S2 | Cys-Gly-Asp-Val-Met | Amino acids and derivatives | Amino acids and derivatives | 5.59E+03 | 1.26E+04 | 6.58E+03 | 9.00E+00 | 9.00E+00 | 9.00E+00 | 1.60E+00 | 6.36E-02 | 1.09E-03 | -9.84E+00 | down |
| MW0157013 | C8H16N2O4S | Ser-Met | Amino acids and derivatives | Amino acids and derivatives | 1.69E+06 | 1.74E+06 | 1.77E+06 | 8.84E+05 | 8.05E+05 | 7.11E+05 | 1.59E+00 | 5.13E-04 | 4.61E-01 | -1.12E+00 | down |
| mws0282 | C11H12N2O2 | L-Tryptophan | Amino acids and derivatives | Amino acids and derivatives | 2.19E+06 | 1.69E+06 | 2.54E+06 | 1.01E+07 | 7.30E+06 | 2.11E+06 | 1.09E+00 | 2.03E-01 | 3.04E+00 | 1.60E+00 | up |
| MWSmce119 | C6H14N4O2 | L-Arginine | Amino acids and derivatives | Amino acids and derivatives | 1.07E+07 | 6.22E+06 | 3.63E+06 | 6.93E+07 | 1.42E+08 | 4.97E+06 | 1.04E+00 | 2.41E-01 | 1.05E+01 | 3.40E+00 | up |
| MW0145782 | C20H36N8O8 | Asn-Ile-Arg-Asp | Amino acids and derivatives | Amino acids and derivatives | 6.04E+05 | 4.26E+05 | 4.17E+05 | 2.35E+05 | 1.66E+05 | 2.09E+05 | 1.52E+00 | 3.41E-02 | 4.22E-01 | -1.24E+00 | down |
| MW0155501 | C24H22N2O7 | Phe-TyrMe-OH | Amino acids and derivatives | Amino acids and derivatives | 2.94E+03 | 4.25E+03 | 5.05E+03 | 4.82E+03 | 1.66E+04 | 7.33E+03 | 1.17E+00 | 2.63E-01 | 2.35E+00 | 1.23E+00 | up |
| MW0144206 | C15H20N2O6 | Abu-Leu-OH | Amino acids and derivatives | Amino acids and derivatives | 4.66E+04 | 2.54E+05 | 1.40E+05 | 5.95E+04 | 2.00E+04 | 5.56E+04 | 1.12E+00 | 2.28E-01 | 3.07E-01 | -1.71E+00 | down |
| MW0153514 | C14H23N3O8S1 | Met-Asp-Glu | Amino acids and derivatives | Amino acids and derivatives | 7.86E+04 | 6.67E+04 | 6.04E+04 | 3.14E+04 | 4.04E+04 | 2.25E+04 | 1.43E+00 | 7.53E-03 | 4.58E-01 | -1.13E+00 | down |
| MW0153757 | C10H18N2O5S | Met-Glu | Amino acids and derivatives | Amino acids and derivatives | 1.44E+05 | 7.63E+04 | 6.36E+04 | 8.79E+05 | 1.36E+06 | 3.37E+05 | 1.47E+00 | 1.22E-01 | 9.10E+00 | 3.19E+00 | up |
| Zmmp002946 | C14H18N2O5 | (3-(carboxyamino)-2-methylpropanoyl)phenylalanine | Amino acids and derivatives | Amino acids and derivatives | 2.00E+04 | 9.09E+03 | 9.61E+03 | 7.73E+04 | 5.02E+04 | 1.74E+04 | 1.21E+00 | 1.73E-01 | 3.74E+00 | 1.90E+00 | up |
| MW0156774 | C22H33N7O11 | Ser-His-Glu-Ala-Glu | Amino acids and derivatives | Amino acids and derivatives | 2.77E+04 | 3.05E+04 | 2.57E+04 | 1.50E+04 | 1.08E+04 | 1.30E+04 | 1.57E+00 | 1.30E-03 | 4.63E-01 | -1.11E+00 | down |
| MW0158225 | C20H24N4O6 | Trp-Pro-Asp | Amino acids and derivatives | Amino acids and derivatives | 1.35E+04 | 3.54E+04 | 2.76E+04 | 2.89E+04 | 1.01E+03 | 3.03E+03 | 1.10E+00 | 2.66E-01 | 4.31E-01 | -1.21E+00 | down |
| MW0157568 | C10H18N4O6 | Thr-Asn-Gly | Amino acids and derivatives | Amino acids and derivatives | 7.21E+04 | 7.56E+04 | 1.13E+05 | 4.49E+06 | 1.73E+06 | 8.86E+04 | 1.10E+00 | 2.57E-01 | 2.42E+01 | 4.60E+00 | up |
| MW0150365 | C18H28N6O11 | Gly-Gly-Asn-Glu-Glu | Amino acids and derivatives | Amino acids and derivatives | 8.02E+04 | 5.65E+04 | 4.12E+04 | 9.09E+03 | 2.42E+04 | 4.33E+04 | 1.08E+00 | 8.96E-02 | 4.30E-01 | -1.22E+00 | down |
| MW0151069 | C22H26N6O4 | His-Pro-Trp | Amino acids and derivatives | Amino acids and derivatives | 1.83E+04 | 4.27E+04 | 1.98E+04 | 1.59E+04 | 5.35E+03 | 1.51E+04 | 1.17E+00 | 1.93E-01 | 4.49E-01 | -1.15E+00 | down |
| **Index** | **Formula** | **Compounds** | **Class I** | **Class II** | **YZ-1-1** | **YZ-1-2** | **YZ-1-3** | **B21-1-1** | **B21-1-2** | **B21-1-3** | **VIP** | **P-value** | **Fold_Change** | **Log2FC** | **Type** |
| MWSmce190 | C6H13NO3 | 4-Hydroxy-L-Isoleucine | Amino acids and derivatives | Amino acids and derivatives | 7.06E+04 | 5.85E+04 | 4.11E+04 | 8.49E+04 | 1.82E+05 | 1.15E+05 | 1.37E+00 | 1.24E-01 | 2.25E+00 | 1.17E+00 | up |
| pme1987 | C3H7NO2 | L-Alanine | Amino acids and derivatives | Amino acids and derivatives | 2.17E+03 | 5.28E+03 | 6.22E+03 | 7.59E+03 | 1.88E+04 | 6.62E+03 | 1.13E+00 | 2.36E-01 | 2.42E+00 | 1.27E+00 | up |
| MW0148017 | C21H35N7O7S | Cys-His-Thr-Gly-Leu | Amino acids and derivatives | Amino acids and derivatives | 4.81E+03 | 4.76E+03 | 2.19E+03 | 1.49E+03 | 5.46E+02 | 2.98E+03 | 1.12E+00 | 1.17E-01 | 4.26E-01 | -1.23E+00 | down |
| MW0146227 | C22H33N7O8 | Asp-Phe-Ser-Arg | Amino acids and derivatives | Amino acids and derivatives | 2.91E+03 | 2.12E+04 | 6.05E+02 | 3.85E+04 | 4.80E+04 | 2.57E+04 | 1.19E+00 | 3.34E-02 | 4.55E+00 | 2.19E+00 | up |
| MW0158478 | C19H23N5O7 | Tyr-His-Asp | Amino acids and derivatives | Amino acids and derivatives | 1.24E+04 | 1.97E+04 | 1.33E+04 | 5.70E+03 | 2.36E+03 | 4.39E+03 | 1.51E+00 | 2.76E-02 | 2.74E-01 | -1.87E+00 | down |
| Zmdp001857 | C14H18N2O6 | γ-Glutamyltyrosine | Amino acids and derivatives | Amino acids and derivatives | 2.21E+04 | 1.37E+04 | 1.02E+04 | 4.71E+04 | 4.81E+04 | 1.99E+04 | 1.24E+00 | 1.16E-01 | 2.51E+00 | 1.33E+00 | up |
| MW0158726 | C23H27N3O5 | Tyr-Pro-Phe | Amino acids and derivatives | Amino acids and derivatives | 7.86E+03 | 8.93E+03 | 4.10E+03 | 2.45E+03 | 1.24E+03 | 2.21E+03 | 1.46E+00 | 6.86E-02 | 2.83E-01 | -1.82E+00 | down |
| Zmzn000113 | C5H9NO4 | L-threo-3-Methylaspartate | Amino acids and derivatives | Amino acids and derivatives | 2.99E+06 | 2.17E+06 | 1.35E+06 | 5.57E+06 | 8.98E+06 | 5.01E+06 | 1.45E+00 | 5.81E-02 | 3.00E+00 | 1.59E+00 | up |
| MWS0631 | C3H7NO5S2 | S-Sulfo-L-Cysteine | Amino acids and derivatives | Amino acids and derivatives | 2.56E+04 | 3.33E+04 | 2.54E+04 | 6.24E+04 | 4.04E+04 | 9.05E+04 | 1.31E+00 | 1.25E-01 | 2.29E+00 | 1.20E+00 | up |
| MW0146130 | C23H28N6O9 | Asp-His-Phe-Asp | Amino acids and derivatives | Amino acids and derivatives | 1.96E+04 | 2.85E+04 | 1.45E+04 | 6.56E+03 | 1.38E+04 | 1.06E+04 | 1.19E+00 | 1.07E-01 | 4.94E-01 | -1.02E+00 | down |
| pme1712 | C11H20N2O6 | L-Saccharopine | Amino acids and derivatives | Amino acids and derivatives | 3.60E+04 | 1.30E+04 | 1.41E+04 | 5.93E+04 | 1.04E+05 | 4.05E+04 | 1.34E+00 | 1.16E-01 | 3.22E+00 | 1.69E+00 | up |
| MW0153524 | C25H37N5O9S2 | Met-Cys-Asp-Phe-Thr | Amino acids and derivatives | Amino acids and derivatives | 7.72E+03 | 1.07E+04 | 7.75E+03 | 3.06E+03 | 1.96E+03 | 2.07E+03 | 1.57E+00 | 1.51E-02 | 2.71E-01 | -1.89E+00 | down |
| Zmdp001647 | C10H18N2O5 | γ-Glutamyl-L-valine | Amino acids and derivatives | Amino acids and derivatives | 9.14E+03 | 8.68E+03 | 1.03E+04 | 3.46E+04 | 3.37E+04 | 1.51E+04 | 1.40E+00 | 1.01E-01 | 2.96E+00 | 1.57E+00 | up |
| Zmzp000145 | C9H20N2O2 | Trimethyllysine | Amino acids and derivatives | Amino acids and derivatives | 1.51E+05 | 9.89E+04 | 1.26E+05 | 5.17E+05 | 6.27E+05 | 1.10E+05 | 1.06E+00 | 2.03E-01 | 3.34E+00 | 1.74E+00 | up |
| MW0146364 | C22H18N2O8 | Asp-Nap-OH | Amino acids and derivatives | Amino acids and derivatives | 4.19E+04 | 3.47E+04 | 1.68E+04 | 1.39E+04 | 5.32E+03 | 7.80E+03 | 1.40E+00 | 8.47E-02 | 2.89E-01 | -1.79E+00 | down |
| pme2735 | C15H22N6O5S | S-(5'-Adenosyl)-L-methionine | Amino acids and derivatives | Amino acids and derivatives | 2.67E+05 | 2.23E+05 | 3.18E+05 | 7.70E+05 | 4.12E+05 | 5.01E+05 | 1.33E+00 | 1.06E-01 | 2.08E+00 | 1.06E+00 | up |
| MW0156686 | C17H29N5O10 | Ser-Asp-Ser-Gly-Val | Amino acids and derivatives | Amino acids and derivatives | 5.58E+04 | 6.06E+04 | 5.45E+04 | 2.19E+04 | 9.18E+03 | 1.24E+04 | 1.55E+00 | 2.45E-03 | 2.54E-01 | -1.98E+00 | down |
| MW0110229 | C27H29N3O7 | H-Tyr-tyr-tyr-OH | Amino acids and derivatives | Amino acids and derivatives | 9.69E+03 | 1.43E+04 | 1.39E+04 | 1.88E+03 | 3.10E+03 | 1.10E+03 | 1.50E+00 | 1.05E-02 | 1.60E-01 | -2.64E+00 | down |
| MW0155159 | C19H27N5O6 | Phe-Gln-Gln | Amino acids and derivatives | Amino acids and derivatives | 1.34E+04 | 2.65E+04 | 1.96E+04 | 4.88E+03 | 3.95E+03 | 4.33E+03 | 1.56E+00 | 5.51E-02 | 2.21E-01 | -2.18E+00 | down |
| MW0155805 | C21H36N6O9 | Pro-Asp-Ala-Lys-Ser | Amino acids and derivatives | Amino acids and derivatives | 2.39E+05 | 1.36E+05 | 1.63E+05 | 6.63E+04 | 4.62E+04 | 5.08E+04 | 1.53E+00 | 5.10E-02 | 3.04E-01 | -1.72E+00 | down |
| **Index** | **Formula** | **Compounds** | **Class I** | **Class II** | **YZ-1-1** | **YZ-1-2** | **YZ-1-3** | **B21-1-1** | **B21-1-2** | **B21-1-3** | **VIP** | **P-value** | **Fold_Change** | **Log2FC** | **Type** |
| MW0145546 | C27H46N8O6 | Arg-Tyr-Leu-Lys | Amino acids and derivatives | Amino acids and derivatives | 1.58E+04 | 3.09E+04 | 2.19E+04 | 1.58E+04 | 7.10E+03 | 1.12E+04 | 1.29E+00 | 1.03E-01 | 4.99E-01 | -1.00E+00 | down |
| MWS201054 | C6H11NO4 | O-Acetyl-L-homoserine | Amino acids and derivatives | Amino acids and derivatives | 4.49E+04 | 3.65E+04 | 2.66E+04 | 7.46E+04 | 1.26E+05 | 8.33E+04 | 1.49E+00 | 5.40E-02 | 2.63E+00 | 1.39E+00 | up |
| MWS00411g | C6H11NO4 | L-2-Aminoadipate | Amino acids and derivatives | Amino acids and derivatives | 9.75E+04 | 7.24E+04 | 5.36E+04 | 1.23E+05 | 3.12E+05 | 1.59E+05 | 1.37E+00 | 1.61E-01 | 2.65E+00 | 1.41E+00 | up |
| pme2758 | C5H9NO5 | 4-Hydroxy-L-glutamic acid | Amino acids and derivatives | Amino acids and derivatives | 1.63E+05 | 2.01E+05 | 1.46E+05 | 3.08E+05 | 4.99E+05 | 3.29E+05 | 1.49E+00 | 6.56E-02 | 2.23E+00 | 1.16E+00 | up |
| MW0158702 | C22H22N4O7 | TyrMe-His-OH | Amino acids and derivatives | Amino acids and derivatives | 2.79E+03 | 3.65E+03 | 4.61E+03 | 9.00E+00 | 9.00E+00 | 9.00E+00 | 1.60E+00 | 2.00E-02 | 2.44E-03 | -8.68E+00 | down |
| MWSmce210 | C7H4O6 | Chelidonic acid | Organic acids | Organic acids | 2.11E+06 | 2.07E+06 | 2.28E+06 | 3.68E+05 | 6.70E+05 | 7.10E+05 | 1.49E+00 | 7.03E-04 | 2.70E-01 | -1.89E+00 | down |
| Wasn003197 | C13H24O9 | 3-(Beta-D-Glucopyranosyloxy)-5-Hydroxyhexanoic Acid Methyl Ester | Organic acids | Organic acids | 2.75E+04 | 2.87E+04 | 5.80E+04 | 1.70E+04 | 5.51E+03 | 1.50E+04 | 1.34E+00 | 1.11E-01 | 3.28E-01 | -1.61E+00 | down |
| Zmtn001464 | C10H7NO4 | 4,8-Dihydroxyquinoline-2-carboxylic acid | Organic acids | Organic acids | 4.05E+04 | 4.11E+04 | 4.18E+04 | 7.89E+04 | 1.07E+05 | 7.82E+04 | 1.57E+00 | 3.93E-02 | 2.14E+00 | 1.10E+00 | up |
| mws0154 | C7H10O5 | Shikimic acid | Organic acids | Organic acids | 2.45E+05 | 2.48E+05 | 2.59E+05 | 8.32E+05 | 1.06E+06 | 5.50E+05 | 1.53E+00 | 6.25E-02 | 3.25E+00 | 1.70E+00 | up |
| mws0281 | C6H8O7 | Citric Acid | Organic acids | Organic acids | 2.40E+06 | 8.77E+05 | 2.29E+06 | 9.31E+06 | 5.31E+06 | 2.58E+06 | 1.17E+00 | 1.80E-01 | 3.09E+00 | 1.63E+00 | up |
| mws2125 | C3H5O6P | Phosphoenolpyruvate | Organic acids | Organic acids | 1.21E+06 | 1.77E+06 | 1.18E+06 | 2.43E+06 | 3.94E+06 | 3.32E+06 | 1.48E+00 | 3.59E-02 | 2.33E+00 | 1.22E+00 | up |
| Lmgn002555 | C8H14O4 | 2-Propylglutaric acid | Organic acids | Organic acids | 6.10E+05 | 5.31E+05 | 4.66E+05 | 1.47E+06 | 9.39E+05 | 8.46E+05 | 1.36E+00 | 1.01E-01 | 2.03E+00 | 1.02E+00 | up |
| MW0104699 | C7H13NO4 | Spermidic acid | Organic acids | Organic acids | 1.51E+06 | 1.66E+06 | 1.66E+06 | 1.05E+06 | 5.59E+05 | 7.52E+05 | 1.49E+00 | 1.90E-02 | 4.89E-01 | -1.03E+00 | down |
| mws0567 | C5H11N3O2 | 4-Guanidinobutyric acid | Organic acids | Organic acids | 9.19E+05 | 6.44E+05 | 5.13E+05 | 3.93E+06 | 5.27E+06 | 1.14E+06 | 1.33E+00 | 1.51E-01 | 4.98E+00 | 2.31E+00 | up |
| Wayn000597 | C18H28O17 | Citric acid-1-O-diglucoside | Organic acids | Organic acids | 2.93E+04 | 1.76E+04 | 1.63E+04 | 9.89E+04 | 6.20E+04 | 3.71E+04 | 1.34E+00 | 1.23E-01 | 3.13E+00 | 1.65E+00 | up |
| Sazp003601 | C18H28O9 | Tuberonic acid glucoside | Organic acids | Organic acids | 1.85E+05 | 1.51E+05 | 1.15E+05 | 8.03E+04 | 4.85E+04 | 2.85E+04 | 1.41E+00 | 2.10E-02 | 3.49E-01 | -1.52E+00 | down |
| Zmgn001448 | C7H12O5 | 2-Propylmalic Acid | Organic acids | Organic acids | 2.38E+04 | 1.79E+04 | 2.94E+04 | 1.53E+05 | 8.68E+04 | 6.04E+04 | 1.44E+00 | 1.07E-01 | 4.22E+00 | 2.08E+00 | up |
| Zmyn000247 | C5H8O5 | 2-Hydroxyglutaric Acid* | Organic acids | Organic acids | 1.75E+05 | 9.55E+04 | 1.57E+05 | 6.88E+05 | 3.89E+05 | 2.32E+05 | 1.29E+00 | 1.55E-01 | 3.06E+00 | 1.61E+00 | up |
| mws0262 | C4H6O6 | L-Tartaric acid | Organic acids | Organic acids | 7.26E+05 | 5.18E+05 | 7.49E+05 | 1.71E+06 | 1.36E+06 | 1.05E+06 | 1.41E+00 | 5.21E-02 | 2.07E+00 | 1.05E+00 | up |
| pmb2826 | C5H8O5 | L-Citramalic acid | Organic acids | Organic acids | 1.94E+05 | 7.37E+04 | 1.86E+05 | 6.35E+05 | 3.27E+05 | 2.04E+05 | 1.12E+00 | 1.98E-01 | 2.57E+00 | 1.36E+00 | up |
| MWS0811 | C6H11NO2 | L-Pipecolic Acid | Organic acids | Organic acids | 2.76E+06 | 2.74E+06 | 1.93E+06 | 5.00E+06 | 8.95E+06 | 6.01E+06 | 1.51E+00 | 6.55E-02 | 2.69E+00 | 1.43E+00 | up |
| Lmrn002746 | C6H12O3 | 2-Hydroxy-4-methylpentanoic acid | Organic acids | Organic acids | 5.94E+04 | 3.86E+04 | 3.21E+04 | 1.30E+05 | 6.70E+04 | 6.57E+04 | 1.17E+00 | 1.61E-01 | 2.02E+00 | 1.01E+00 | up |
| mws0425 | C5H6O4 | Citraconic acid | Organic acids | Organic acids | 6.31E+05 | 2.75E+05 | 6.13E+05 | 2.38E+06 | 1.12E+06 | 7.95E+05 | 1.19E+00 | 1.91E-01 | 2.83E+00 | 1.50E+00 | up |
| pmb3101 | C7H12O5 | 2-Isopropylmalic Acid | Organic acids | Organic acids | 1.87E+04 | 1.80E+04 | 2.82E+04 | 1.66E+05 | 8.90E+04 | 5.52E+04 | 1.42E+00 | 1.29E-01 | 4.79E+00 | 2.26E+00 | up |
| pmb2657 | C10H18N4O6 | Argininosuccinic acid | Organic acids | Organic acids | 1.50E+05 | 1.01E+05 | 1.31E+05 | 3.55E+05 | 6.58E+05 | 3.08E+05 | 1.50E+00 | 1.02E-01 | 3.46E+00 | 1.79E+00 | up |
| Wayn000918 | C18H28O17 | Isocitric acid-1-O-diglucoside | Organic acids | Organic acids | 3.87E+04 | 2.68E+04 | 2.46E+04 | 1.10E+05 | 1.33E+05 | 3.48E+04 | 1.21E+00 | 1.67E-01 | 3.08E+00 | 1.62E+00 | up |
| Hmqn003054 | C18H34O5 | 9,10,11-Trihydroxy-12-octadecenoic acid | Lipids | Free fatty acids | 2.15E+04 | 1.54E+04 | 1.83E+04 | 5.10E+04 | 6.01E+04 | 5.00E+04 | 1.58E+00 | 2.04E-03 | 2.92E+00 | 1.54E+00 | up |
| pmb0865 | C26H48NO7P | LysoPC 18:3(2n isomer) | Lipids | LPC | 9.52E+04 | 1.72E+05 | 1.03E+05 | 2.67E+05 | 3.61E+05 | 2.61E+05 | 1.46E+00 | 1.51E-02 | 2.40E+00 | 1.27E+00 | up |
| pmb0296 | C21H40O4 | 1-Oleoyl-Sn-Glycerol | Lipids | Glycerol ester | 4.00E+05 | 6.48E+05 | 4.19E+05 | 2.16E+05 | 5.81E+04 | 2.36E+05 | 1.32E+00 | 3.57E-02 | 3.47E-01 | -1.53E+00 | down |
| **Index** | **Formula** | **Compounds** | **Class I** | **Class II** | **YZ-1-1** | **YZ-1-2** | **YZ-1-3** | **B21-1-1** | **B21-1-2** | **B21-1-3** | **VIP** | **P-value** | **Fold_Change** | **Log2FC** | **Type** |
| Lmbn007891 | C18H34O4 | Hydroxy ricinoleic acid | Lipids | Free fatty acids | 1.32E+04 | 1.25E+04 | 1.22E+04 | 3.51E+04 | 2.65E+04 | 2.22E+04 | 1.49E+00 | 5.58E-02 | 2.21E+00 | 1.14E+00 | up |
| MWS2673 | C20H34O4 | 5,6-DiHETrE[(±)5,6-dihydroxy-8Z,11Z,14Z-eicosatrienoic acid] | Lipids | Free fatty acids | 3.74E+04 | 4.75E+04 | 3.43E+04 | 2.11E+04 | 6.84E+03 | 1.82E+04 | 1.38E+00 | 1.48E-02 | 3.87E-01 | -1.37E+00 | down |
| mws0126 | C26H54NO7P | LysoPC 18:0 | Lipids | LPC | 2.72E+05 | 1.57E+05 | 2.45E+05 | 6.90E+05 | 5.08E+05 | 3.40E+05 | 1.33E+00 | 9.12E-02 | 2.28E+00 | 1.19E+00 | up |
| pmb2804 | C18H32O4 | 13S-Hydroperoxy-9Z,11E-octadecadienoic acid | Lipids | Free fatty acids | 9.90E+03 | 8.71E+03 | 6.96E+03 | 4.34E+04 | 2.86E+04 | 1.35E+04 | 1.32E+00 | 1.46E-01 | 3.34E+00 | 1.74E+00 | up |
| pmn001694 | C18H34O5 | 9,10,13-Trihydroxy-11-Octadecenoic Acid | Lipids | Free fatty acids | 3.30E+05 | 2.11E+05 | 2.43E+05 | 1.03E+06 | 1.26E+06 | 1.05E+06 | 1.58E+00 | 2.56E-03 | 4.26E+00 | 2.09E+00 | up |
| Lmyn012331 | C18H34O2 | Petroselinic acid* | Lipids | Free fatty acids | 1.62E+07 | 3.72E+07 | 1.47E+07 | 1.21E+07 | 1.05E+07 | 9.92E+06 | 1.21E+00 | 2.43E-01 | 4.77E-01 | -1.07E+00 | down |
| pmd0136 | C26H54NO7P | LysoPC 18:0(2n isomer) | Lipids | LPC | 2.94E+05 | 1.81E+05 | 2.59E+05 | 7.31E+05 | 5.29E+05 | 3.62E+05 | 1.33E+00 | 9.79E-02 | 2.21E+00 | 1.15E+00 | up |
| mws2623 | C18H34O2 | 11-Octadecanoic acid(Vaccenic acid)* | Lipids | Free fatty acids | 1.41E+07 | 3.25E+07 | 1.24E+07 | 1.12E+07 | 9.18E+06 | 7.78E+06 | 1.18E+00 | 2.49E-01 | 4.77E-01 | -1.07E+00 | down |
| pmb0854 | C26H48NO7P | LysoPC 18:3 | Lipids | LPC | 8.37E+04 | 1.50E+05 | 1.12E+05 | 2.49E+05 | 3.88E+05 | 2.81E+05 | 1.49E+00 | 2.97E-02 | 2.66E+00 | 1.41E+00 | up |
| mws0396 | C18H34O2 | Elaidic Acid* | Lipids | Free fatty acids | 4.98E+07 | 9.47E+07 | 3.92E+07 | 3.54E+07 | 2.91E+07 | 2.35E+07 | 1.25E+00 | 1.98E-01 | 4.79E-01 | -1.06E+00 | down |
| Zmpn003368 | C18H30O3 | 13S-Hydroxy-9Z,11E,15Z-octadecatrienoic acid | Lipids | Free fatty acids | 5.13E+03 | 8.45E+03 | 7.31E+03 | 1.88E+04 | 3.94E+04 | 1.60E+04 | 1.45E+00 | 1.36E-01 | 3.55E+00 | 1.83E+00 | up |
| Lmyn006221 | C33H60O14 | Gingerglycolipid C | Lipids | Glycerol ester | 2.49E+05 | 7.13E+05 | 3.09E+05 | 2.04E+05 | 4.51E+04 | 1.93E+05 | 1.19E+00 | 1.91E-01 | 3.48E-01 | -1.52E+00 | down |
| Lmhp008801 | C23H42NO7P | LysoPE 18:3 | Lipids | LPE | 1.88E+04 | 3.36E+04 | 2.24E+04 | 4.81E+04 | 6.73E+04 | 5.18E+04 | 1.45E+00 | 1.61E-02 | 2.24E+00 | 1.16E+00 | up |
| Lmhp009384 | C33H58O14 | 1-Linoleoylglycerol-2,3-di-O-glucoside* | Lipids | Glycerol ester | 5.46E+03 | 7.60E+03 | 3.38E+03 | 5.22E+03 | 7.86E+02 | 1.86E+03 | 1.10E+00 | 1.90E-01 | 4.79E-01 | -1.06E+00 | down |
| pme0500 | C18H32O16 | D-Melezitose | Others | Saccharides | 1.68E+06 | 1.89E+06 | 1.95E+06 | 2.19E+05 | 3.26E+05 | 7.41E+05 | 1.44E+00 | 4.18E-03 | 2.33E-01 | -2.10E+00 | down |
| Zmyn000083 | C6H14O12P2 | D-Glucose 1,6-bisphosphate | Others | Saccharides | 1.22E+06 | 9.07E+05 | 1.10E+06 | 5.58E+04 | 4.71E+05 | 5.69E+05 | 1.04E+00 | 2.63E-02 | 3.40E-01 | -1.56E+00 | down |
| pme2125 | C18H32O16 | Raffinose* | Others | Saccharides | 1.45E+07 | 2.39E+07 | 1.94E+07 | 3.52E+06 | 8.42E+06 | 1.34E+07 | 1.15E+00 | 5.26E-02 | 4.39E-01 | -1.19E+00 | down |
| pme3163 | C7H15O10P | D-Sedoheptuiose 7-phosphate | Others | Saccharides | 1.21E+07 | 7.36E+06 | 1.07E+07 | 8.22E+06 | 2.81E+07 | 4.38E+07 | 1.04E+00 | 2.45E-01 | 2.66E+00 | 1.41E+00 | up |
| mws0866 | C6H13O9P | D-Glucose 6-phosphate* | Others | Saccharides | 1.35E+06 | 7.49E+05 | 1.45E+06 | 9.24E+05 | 4.64E+06 | 4.49E+06 | 1.05E+00 | 2.13E-01 | 2.83E+00 | 1.50E+00 | up |
| Zmgn000173 | C5H10O5 | D-Ribose | Others | Saccharides | 2.32E+04 | 1.42E+04 | 1.81E+04 | 5.33E+04 | 4.31E+04 | 2.00E+04 | 1.14E+00 | 1.70E-01 | 2.09E+00 | 1.07E+00 | up |
| MW0154413 | C27H45N5O20 | Neu5Gc2-6Gal1-4GlcNAcSp | Others | Saccharides | 9.00E+00 | 9.00E+00 | 9.00E+00 | 3.12E+03 | 3.89E+03 | 3.32E+03 | 1.60E+00 | 4.46E-03 | 3.82E+02 | 8.58E+00 | up |
| Lmdn000248 | C30H52O26 | Verbascose | Others | Saccharides | 1.21E+04 | 1.77E+04 | 2.07E+04 | 2.18E+03 | 1.98E+03 | 5.39E+03 | 1.49E+00 | 1.96E-02 | 1.89E-01 | -2.40E+00 | down |
| mws1593 | C24H42O21 | D-Maltotetraose | Others | Saccharides | 9.41E+05 | 2.07E+06 | 1.08E+06 | 1.31E+05 | 5.45E+04 | 2.55E+05 | 1.50E+00 | 7.29E-02 | 1.08E-01 | -3.21E+00 | down |
| MWSmce486 | C18H32O16 | Manninotriose | Others | Saccharides | 4.54E+04 | 6.14E+04 | 4.75E+04 | 7.33E+03 | 7.39E+03 | 2.34E+04 | 1.43E+00 | 6.26E-03 | 2.47E-01 | -2.02E+00 | down |
| mws1589 | C18H32O16 | D-Panose* | Others | Saccharides | 2.84E+06 | 6.18E+06 | 3.61E+06 | 5.27E+05 | 1.50E+06 | 1.62E+06 | 1.30E+00 | 8.46E-02 | 2.89E-01 | -1.79E+00 | down |
| Lmqn000213 | C24H42O21 | Stachyose | Others | Saccharides | 6.40E+06 | 1.34E+07 | 5.80E+06 | 5.69E+05 | 3.29E+05 | 2.82E+06 | 1.38E+00 | 8.43E-02 | 1.45E-01 | -2.78E+00 | down |
| mws4163 | C24H42O21 | Nystose | Others | Saccharides | 7.05E+06 | 1.28E+07 | 7.91E+06 | 9.08E+05 | 2.53E+05 | 2.28E+06 | 1.44E+00 | 3.53E-02 | 1.24E-01 | -3.02E+00 | down |

| **Supplementary Table S5. Annotations analysis of Metabolites in pathway of figure 4** | | | | | | | | | | | | | |
| --- | --- | --- | --- | --- | --- | --- | --- | --- | --- | --- | --- | --- | --- |
| **Compounds** | **Class I** | **Class II** | **YZ-1** | **YZ-2** | **YZ-3** | **B21-1** | **B21-2** | **B21-3** | **VIP** | **P-value** | **Fold_Change** | **Log2FC** | **Type** |
| Raffinose | Others | Saccharides | 1.45E+07 | 2.39E+07 | 1.94E+07 | 3.52E+06 | 8.42E+06 | 1.34E+07 | 1.15E+00 | 5.26E-02 | 4.39E-01 | -1.19E+00 | down |
| Manninotriose | Others | Saccharides | 4.54E+04 | 6.14E+04 | 4.75E+04 | 7.33E+03 | 7.39E+03 | 2.34E+04 | 1.43E+00 | 6.26E-03 | 2.47E-01 | -2.02E+00 | down |
| Stachyose | Others | Saccharides | 6.40E+06 | 1.34E+07 | 5.80E+06 | 5.69E+05 | 3.29E+05 | 2.82E+06 | 1.38E+00 | 8.43E-02 | 1.45E-01 | -2.78E+00 | down |
| D-Glucose 6-phosphate | Others | Saccharides | 1.35E+06 | 7.49E+05 | 1.45E+06 | 9.24E+05 | 4.64E+06 | 4.49E+06 | 1.05E+00 | 2.13E-01 | 2.83E+00 | 1.50E+00 | up |
| Citric Acid | Organic acids | Organic acids | 2.40E+06 | 8.77E+05 | 2.29E+06 | 9.31E+06 | 5.31E+06 | 2.58E+06 | 1.17E+00 | 1.80E-01 | 3.09E+00 | 1.63E+00 | up |
| Shikimic acid | Organic acids | Organic acids | 2.45E+05 | 2.48E+05 | 2.59E+05 | 8.32E+05 | 1.06E+06 | 5.50E+05 | 1.53E+00 | 6.25E-02 | 3.25E+00 | 1.70E+00 | up |
| Phosphoenolpyruvate | Organic acids | Organic acids | 1.21E+06 | 1.77E+06 | 1.18E+06 | 2.43E+06 | 3.94E+06 | 3.32E+06 | 1.48E+00 | 3.59E-02 | 2.33E+00 | 1.22E+00 | up |
| 13S-Hydroperoxy-9Z,11E-octadecadienoic acid | Lipids | Free fatty acids | 9.90E+03 | 8.71E+03 | 6.96E+03 | 4.34E+04 | 2.86E+04 | 1.35E+04 | 1.32E+00 | 1.46E-01 | 3.34E+00 | 1.74E+00 | up |
| 9,10,13-Trihydroxy-11-Octadecenoic Acid | Lipids | Free fatty acids | 3.30E+05 | 2.11E+05 | 2.43E+05 | 1.03E+06 | 1.26E+06 | 1.05E+06 | 1.58E+00 | 2.56E-03 | 4.26E+00 | 2.09E+00 | up |
| 13S-Hydroxy-9Z,11E,15Z-octadecatrienoic acid | Lipids | Free fatty acids | 5.13E+03 | 8.45E+03 | 7.31E+03 | 1.88E+04 | 3.94E+04 | 1.60E+04 | 1.45E+00 | 1.36E-01 | 3.55E+00 | 1.83E+00 | up |
| L-Tryptophan | Amino acids and derivatives | Amino acids and derivatives | 2.19E+06 | 1.69E+06 | 2.54E+06 | 1.01E+07 | 7.30E+06 | 2.11E+06 | 1.09E+00 | 2.03E-01 | 3.04E+00 | 1.60E+00 | up |
| L-Valine | Amino acids and derivatives | Amino acids and derivatives | 8.29E+05 | 6.63E+05 | 5.62E+05 | 2.41E+06 | 1.31E+06 | 8.72E+05 | 1.17E+00 | 2.02E-01 | 2.24E+00 | 1.16E+00 | up |
| L-Saccharopine | Amino acids and derivatives | Amino acids and derivatives | 3.60E+04 | 1.30E+04 | 1.41E+04 | 5.93E+04 | 1.04E+05 | 4.05E+04 | 1.34E+00 | 1.16E-01 | 3.22E+00 | 1.69E+00 | up |
| L-Alanine | Amino acids and derivatives | Amino acids and derivatives | 2.17E+03 | 5.28E+03 | 6.22E+03 | 7.59E+03 | 1.88E+04 | 6.62E+03 | 1.13E+00 | 2.36E-01 | 2.42E+00 | 1.27E+00 | up |
| L-Arginine | Amino acids and derivatives | Amino acids and derivatives | 1.07E+07 | 6.22E+06 | 3.63E+06 | 6.93E+07 | 1.42E+08 | 4.97E+06 | 1.04E+00 | 2.41E-01 | 1.05E+01 | 3.40E+00 | up |

| **Supplementary Table S6. The correlation analysis of the related flavor metabolites and synthesis-related genes of 5 fresh *C.heterophylla* × *C.avellana* of figure 5** *. At the 0.05 level (two-tailed), the correlation was significant. | | | | | | | | | | | | | | | | | | | | | | | | | | | | | | | | | | | | | | | |
| --- | --- | --- | --- | --- | --- | --- | --- | --- | --- | --- | --- | --- | --- | --- | --- | --- | --- | --- | --- | --- | --- | --- | --- | --- | --- | --- | --- | --- | --- | --- | --- | --- | --- | --- | --- | --- | --- | --- | --- |
|  | Raffinose | | Stachyose | | Manninotriose | | D-Glucose 6-phosphate | | Citric Acid | | Shikimic acid | | 13(S)-HPODE | | 9,10,13-TriHOME | | 13(S)-HOTrE | | L-Tryptophan | | L-Valine | | L-Saccharopine | | L-Alanine | | L-Arginine | | E2.4.1.82-1 | | E2.4.1.82-2 | | | E2.4.1.67-1 | | E2.4.1.67-2 | | INV | |
| Raffinose | 1 | | .904* | | .941** | | -0.308 | | -.920** | | -.823* | | -.899* | | -.819* | | -0.615 | | -.874* | | -.878* | | -0.776 | | -0.453 | | -0.680 | | -0.289 | | -0.182 | | | 0.777 | | 0.696 | | -0.401 | |
| Stachyose | .904* | | 1 | | .941** | | -0.562 | | -0.756 | | -0.803 | | -0.706 | | -.848* | | -0.668 | | -0.712 | | -0.642 | | -0.767 | | -0.561 | | -0.644 | | -0.438 | | -0.319 | | | 0.640 | | 0.519 | | -0.456 | |
| Manninotriose | .941** | | .941** | | 1 | | -0.559 | | -.837* | | -.935** | | -.853* | | -.953** | | -0.796 | | -.828* | | -0.775 | | -.859* | | -0.663 | | -0.765 | | -0.437 | | -0.329 | | | 0.772 | | 0.697 | | -0.504 | |
| D-Glucose 6-phosphate | -0.308 | | -0.562 | | -0.559 | | 1 | | 0.016 | | 0.578 | | 0.088 | | 0.730 | | 0.703 | | 0.056 | | -0.065 | | 0.632 | | 0.663 | | 0.461 | | 0.626 | | 0.591 | | | -0.521 | | -0.473 | | 0.497 | |
| Citric Acid | -.920** | | -0.756 | | -.837* | | 0.016 | | 1 | | 0.762 | | .976** | | 0.670 | | 0.519 | | .973** | | .976** | | 0.628 | | 0.394 | | 0.642 | | 0.096 | | -0.012 | | | -0.566 | | -0.511 | | 0.260 | |
| Shikimic acid | -.823* | | -0.803 | | -.935** | | 0.578 | | 0.762 | | 1 | | .835* | | .933** | | .942** | | .833* | | 0.709 | | .939** | | .854* | | .919** | | 0.253 | | 0.156 | | | -0.704 | | -0.668 | | 0.313 | |
| 13(S)-HPODE | -.899* | | -0.706 | | -.853* | | 0.088 | | .976** | | .835* | | 1 | | 0.737 | | 0.623 | | .980** | | .978** | | 0.701 | | 0.490 | | 0.721 | | 0.110 | | 0.014 | | | -0.644 | | -0.618 | | 0.281 | |
| 9,10,13-TriHOME | -.819* | | -.848* | | -.953** | | 0.730 | | 0.670 | | .933** | | 0.737 | | 1 | | .854* | | 0.682 | | 0.632 | | .862* | | 0.719 | | 0.741 | | 0.572 | | 0.490 | | | -.824* | | -0.778 | | 0.611 | |
| 13(S)-HOTrE | -0.615 | | -0.668 | | -0.796 | | 0.703 | | 0.519 | | .942** | | 0.623 | | .854* | | 1 | | 0.645 | | 0.448 | | .941** | | .968** | | .941** | | 0.166 | | 0.086 | | | -0.582 | | -0.561 | | 0.163 | |
| L-Tryptophan | -.874* | | -0.712 | | -.828* | | 0.056 | | .973** | | .833* | | .980** | | 0.682 | | 0.645 | | 1 | | .943** | | 0.714 | | 0.552 | | 0.774 | | -0.025 | | -0.133 | | | -0.528 | | -0.489 | | 0.129 | |
| L-Valine | -.878* | | -0.642 | | -0.775 | | -0.065 | | .976** | | 0.709 | | .978** | | 0.632 | | 0.448 | | .943** | | 1 | | 0.562 | | 0.297 | | 0.571 | | 0.107 | | 0.020 | | | -0.615 | | -0.590 | | 0.303 | |
| L-Saccharopine | -0.776 | | -0.767 | | -.859* | | 0.632 | | 0.628 | | .939** | | 0.701 | | .862* | | .941** | | 0.714 | | 0.562 | | 1 | | .857* | | .949** | | 0.123 | | 0.036 | | | -0.732 | | -0.688 | | 0.145 | |
| L-Alanine | -0.453 | | -0.561 | | -0.663 | | 0.663 | | 0.394 | | .854* | | 0.490 | | 0.719 | | .968** | | 0.552 | | 0.297 | | .857* | | 1 | | .913* | | 0.034 | | -0.045 | | | -0.362 | | -0.343 | | -0.005 | |
| L-Arginine | -0.680 | | -0.644 | | -0.765 | | 0.461 | | 0.642 | | .919** | | 0.721 | | 0.741 | | .941** | | 0.774 | | 0.571 | | .949** | | .913* | | 1 | | -0.112 | | -0.202 | | | -0.534 | | -0.510 | | -0.074 | |
| E2.4.1.82-1 | -0.289 | | -0.438 | | -0.437 | | 0.626 | | 0.096 | | 0.253 | | 0.110 | | 0.572 | | 0.166 | | -0.025 | | 0.107 | | 0.123 | | 0.034 | | -0.112 | | 1 | | .991** | | | -0.501 | | -0.465 | | .970** | |
| E2.4.1.82-2 | -0.182 | | -0.319 | | -0.329 | | 0.591 | | -0.012 | | 0.156 | | 0.014 | | 0.490 | | 0.086 | | -0.133 | | 0.020 | | 0.036 | | -0.045 | | -0.202 | | .991** | | 1 | | | -0.465 | | -0.446 | | .957** | |
| E2.4.1.67-1 | 0.777 | | 0.640 | | 0.772 | | -0.521 | | -0.566 | | -0.704 | | -0.644 | | -.824* | | -0.582 | | -0.528 | | -0.615 | | -0.732 | | -0.362 | | -0.534 | | -0.501 | | -0.465 | | | 1 | | .986** | | -0.588 | |
| E2.4.1.67-2 | 0.696 | | 0.519 | | 0.697 | | -0.473 | | -0.511 | | -0.668 | | -0.618 | | -0.778 | | -0.561 | | -0.489 | | -0.590 | | -0.688 | | -0.343 | | -0.510 | | -0.465 | | -0.446 | | | .986** | | 1 | | -0.565 | |
| INV | -0.401 | | -0.456 | | -0.504 | | 0.497 | | 0.260 | | 0.313 | | 0.281 | | 0.611 | | 0.163 | | 0.129 | | 0.303 | | 0.145 | | -0.005 | | -0.074 | | .970** | | .957** | | | -0.588 | | -0.565 | | 1 | |
| SUS-1 | -0.705 | | -0.783 | | -.887* | | .818* | | 0.533 | | .875* | | 0.613 | | .982** | | .832* | | 0.547 | | 0.497 | | 0.797 | | 0.711 | | 0.661 | | 0.670 | | 0.601 | | | -0.779 | | -0.741 | | 0.678 | |
| SUS-2 | -0.688 | | -0.692 | | -.812* | | 0.564 | | 0.630 | | .954** | | 0.714 | | .813* | | .978** | | 0.756 | | 0.555 | | .959** | | .946** | | .989** | | 0.021 | | -0.070 | | | -0.564 | | -0.535 | | 0.046 | |
| SUS-3 | -.821* | | -0.723 | | -.861* | | 0.322 | | .845* | | .951** | | .901* | | 0.789 | | .869* | | .932** | | 0.796 | | .896* | | 0.801 | | .947** | | -0.022 | | -0.125 | | | -0.589 | | -0.560 | | 0.076 | |
| SUS-4 | -0.426 | | -0.578 | | -0.605 | | 0.809 | | 0.181 | | 0.479 | | 0.228 | | 0.756 | | 0.434 | | 0.107 | | 0.173 | | 0.402 | | 0.296 | | 0.164 | | .951** | | .926** | | | -0.664 | | -0.624 | | .910* | |
| SUS-5 | -.878* | | -0.694 | | -.833* | | 0.055 | | .978** | | .828* | | .996** | | 0.705 | | 0.622 | | .992** | | .970** | | 0.687 | | 0.508 | | 0.732 | | 0.053 | | -0.047 | | | -0.579 | | -0.551 | | 0.222 | |
| SUS-6 | -.866* | | -0.794 | | -.918** | | 0.310 | | .913* | | .883* | | .938** | | .853* | | 0.694 | | .909* | | .887* | | 0.692 | | 0.579 | | 0.685 | | 0.367 | | 0.264 | | | -0.619 | | -0.579 | | 0.491 | |
| SUS-7 | -.889* | | -0.719 | | -.857* | | 0.123 | | .963** | | .872* | | .990** | | 0.738 | | 0.693 | | .994** | | .944** | | 0.761 | | 0.584 | | 0.802 | | 0.027 | | -0.075 | | | -0.607 | | -0.577 | | 0.182 | |
| SUS-8 | 0.567 | | 0.417 | | 0.675 | | -0.494 | | -0.492 | | -0.728 | | -0.643 | | -0.811 | | -0.648 | | -0.514 | | -0.582 | | -0.624 | | -0.478 | | -0.528 | | -0.528 | | -0.512 | | | .838* | | .890* | | -0.634 | |
| SUS-9 | 0.504 | | 0.312 | | 0.588 | | -0.420 | | -0.435 | | -0.659 | | -0.597 | | -0.732 | | -0.587 | | -0.462 | | -0.547 | | -0.577 | | -0.413 | | -0.487 | | -0.454 | | -0.453 | | | .829* | | .897* | | -0.570 | |
| SUS-10 | 0.595 | | 0.375 | | 0.547 | | -0.361 | | -0.398 | | -0.521 | | -0.505 | | -0.626 | | -0.427 | | -0.372 | | -0.498 | | -0.595 | | -0.206 | | -0.412 | | -0.348 | | -0.351 | | | .948** | | .974** | | -0.450 | |
| UGP2-1 | 0.659 | | 0.784 | | .841* | | -0.577 | | -0.651 | | -0.806 | | -0.668 | | -.848* | | -0.709 | | -0.654 | | -0.573 | | -0.599 | | -0.655 | | -0.568 | | -0.579 | | -0.480 | | | 0.441 | | 0.382 | | -0.604 | |
| UGP2-2 | 0.770 | | 0.660 | | .867* | | -0.562 | | -0.682 | | -.924** | | -0.800 | | -.932** | | -.845* | | -0.727 | | -0.705 | | -.858* | | -0.695 | | -0.784 | | -0.395 | | -0.336 | | | .867* | | .874* | | -0.489 | |
| GPI | -0.425 | | -0.493 | | -0.530 | | 0.483 | | 0.303 | | 0.333 | | 0.312 | | 0.619 | | 0.173 | | 0.170 | | 0.333 | | 0.146 | | 0.015 | | -0.059 | | .965** | | .942** | | | -0.553 | | -0.522 | | .996** | |
| PFK-1 | -0.052 | | -0.227 | | -0.208 | | 0.593 | | -0.161 | | 0.038 | | -0.141 | | 0.384 | | 0.006 | | -0.280 | | -0.136 | | -0.053 | | -0.102 | | -0.295 | | .966^**^ | | .987^**^ | | | -0.368 | | -0.349 | | .901^*^ | |
|  | | | | | | | | | | | | | | | | | | | | | | | | | | | | | | | | | | | | | | | |
|  | **Raffinose** | | **Stachyose** | | **Manninotriose** | | **D-Glucose 6-phosphate** | | **Citric Acid** | | **Shikimic acid** | | **13(S)-HPODE** | | **9,10,13-TriHOME** | | **13(S)-HOTrE** | | **L-Tryptophan** | | **L-Valine** | | **L-Saccharopine** | | **L-Alanine** | | **L-Arginine** | | **E2.4.1.82-1** | | **E2.4.1.82-2** | | | **E2.4.1.67-1** | | **E2.4.1.67-2** | | **INV** | |
| **PFK-2** | -0.374 | | -0.486 | | -0.502 | | 0.589 | | 0.198 | | 0.312 | | 0.213 | | 0.620 | | 0.194 | | 0.073 | | 0.218 | | 0.167 | | 0.043 | | -0.064 | | .993^**^ | | .977^**^ | | | -0.561 | | -0.526 | | .990^**^ | |
| **ALDO** | -0.288 | | -0.452 | | -0.476 | | 0.778 | | 0.046 | | 0.352 | | 0.097 | | 0.654 | | 0.326 | | -0.033 | | 0.053 | | 0.273 | | 0.199 | | 0.033 | | .969^**^ | | .963^**^ | | | -0.582 | | -0.555 | | .916^*^ | |
| **GADPH** | -0.678 | | -0.599 | | -0.705 | | 0.282 | | 0.651 | | 0.534 | | 0.666 | | 0.714 | | 0.291 | | 0.536 | | 0.701 | | 0.315 | | 0.101 | | 0.173 | | 0.763 | | 0.715 | | | -0.677 | | -0.654 | | .887^*^ | |
| **PK-1** | -0.328 | | -0.538 | | -0.587 | | .988^**^ | | 0.064 | | 0.646 | | 0.164 | | 0.772 | | 0.772 | | 0.125 | | 0.001 | | 0.684 | | 0.727 | | 0.536 | | 0.594 | | 0.561 | | | -0.566 | | -0.538 | | 0.487 | |
| **PK-2** | .899^*^ | | .917^**^ | | .904^*^ | | -0.610 | | -0.666 | | -0.746 | | -0.654 | | -.872^*^ | | -0.602 | | -0.595 | | -0.615 | | -0.760 | | -0.423 | | -0.552 | | -0.566 | | -0.481 | | | .868^*^ | | 0.774 | | -0.600 | |
| **PK-3** | 0.606 | | 0.423 | | 0.666 | | -0.458 | | -0.496 | | -0.692 | | -0.638 | | -0.786 | | -0.595 | | -0.500 | | -0.596 | | -0.631 | | -0.399 | | -0.504 | | -0.500 | | -0.489 | | | .908^*^ | | .954^**^ | | -0.615 | |
| **PDAH** | -0.288 | | -0.421 | | -0.442 | | 0.649 | | 0.087 | | 0.277 | | 0.118 | | 0.594 | | 0.200 | | -0.024 | | 0.109 | | 0.155 | | 0.061 | | -0.082 | | .996^**^ | | .992^**^ | | | -0.543 | | -0.516 | | .970^**^ | |
| **CS** | .847^*^ | | .950^**^ | | .953^**^ | | -0.664 | | -0.707 | | -.833^*^ | | -0.698 | | -.932^**^ | | -0.703 | | -0.663 | | -0.624 | | -0.730 | | -0.582 | | -0.593 | | -0.642 | | -0.538 | | | 0.689 | | 0.596 | | -0.663 | |
| **ACO-1** | -0.072 | | -0.245 | | -0.229 | | 0.601 | | -0.141 | | 0.060 | | -0.119 | | 0.403 | | 0.025 | | -0.259 | | -0.116 | | -0.035 | | -0.085 | | -0.277 | | .971^**^ | | .990^**^ | | | -0.381 | | -0.361 | | .908^*^ | |
| **ACO-2** | -0.095 | | -0.253 | | -0.243 | | 0.581 | | -0.112 | | 0.067 | | -0.092 | | 0.411 | | 0.018 | | -0.235 | | -0.083 | | -0.034 | | -0.100 | | -0.277 | | .976^**^ | | .994^**^ | | | -0.400 | | -0.381 | | .923^**^ | |
| **ACO-3** | -0.087 | | -0.252 | | -0.241 | | 0.598 | | -0.125 | | 0.071 | | -0.102 | | 0.414 | | 0.030 | | -0.244 | | -0.098 | | -0.025 | | -0.085 | | -0.269 | | .974^**^ | | .993^**^ | | | -0.398 | | -0.379 | | .916^*^ | |
| **IDH3** | -0.452 | | -0.527 | | -0.561 | | 0.514 | | 0.320 | | 0.367 | | 0.330 | | 0.650 | | 0.210 | | 0.192 | | 0.344 | | 0.186 | | 0.051 | | -0.022 | | .966^**^ | | .940^**^ | | | -0.575 | | -0.539 | | .994^**^ | |
| **OGDH** | 0.709 | | 0.659 | | .860^*^ | | -0.571 | | -0.674 | | -.904^*^ | | -0.782 | | -.933^**^ | | -.814^*^ | | -0.710 | | -0.686 | | -0.750 | | -0.691 | | -0.700 | | -0.517 | | -0.452 | | | 0.740 | | 0.745 | | -0.602 | |
| **trpE** | -0.732 | | -0.717 | | -.829^*^ | | 0.544 | | 0.660 | | .953^**^ | | 0.736 | | .815^*^ | | .962^**^ | | 0.775 | | 0.587 | | .976^**^ | | .915^*^ | | .992^**^ | | 0.008 | | -0.085 | | | -0.607 | | -0.573 | | 0.041 | |
| **trpD** | .830^*^ | | 0.779 | | .938^**^ | | -0.505 | | -0.810 | | -.951^**^ | | -.881^*^ | | -.944^**^ | | -.826^*^ | | -.837^*^ | | -0.792 | | -0.803 | | -0.706 | | -0.767 | | -0.432 | | -0.342 | | | 0.726 | | 0.703 | | -0.528 | |
| **ilvA** | -0.087 | | -0.247 | | -0.235 | | 0.581 | | -0.121 | | 0.060 | | -0.101 | | 0.405 | | 0.014 | | -0.244 | | -0.092 | | -0.039 | | -0.103 | | -0.282 | | .974^**^ | | .993^**^ | | | -0.395 | | -0.376 | | .919^**^ | |
| **ilvH** | 0.627 | | 0.526 | | 0.761 | | -0.547 | | -0.565 | | -0.803 | | -0.699 | | -.879^*^ | | -0.715 | | -0.587 | | -0.624 | | -0.667 | | -0.559 | | -0.581 | | -0.572 | | -0.536 | | | 0.808 | | .840^*^ | | -0.668 | |
| **ilvE-1** | -0.072 | | -0.238 | | -0.223 | | 0.588 | | -0.140 | | 0.052 | | -0.119 | | 0.396 | | 0.012 | | -0.261 | | -0.112 | | -0.043 | | -0.101 | | -0.287 | | .970^**^ | | .991^**^ | | | -0.385 | | -0.366 | | .911^*^ | |
| **ilvE-2** | -0.479 | | -0.586 | | -0.615 | | 0.628 | | 0.311 | | 0.439 | | 0.330 | | 0.718 | | 0.313 | | 0.200 | | 0.318 | | 0.285 | | 0.161 | | 0.068 | | .974^**^ | | .942^**^ | | | -0.616 | | -0.574 | | .980^**^ | |
| **ilvE-3** | -0.697 | | -0.662 | | -0.762 | | 0.513 | | 0.564 | | 0.609 | | 0.604 | | .823^*^ | | 0.423 | | 0.465 | | 0.603 | | 0.461 | | 0.221 | | 0.265 | | .840^*^ | | 0.796 | | | -.814^*^ | | -0.787 | | .921^**^ | |
| **IMDH** | -0.494 | | -0.675 | | -0.679 | | 0.760 | | 0.310 | | 0.538 | | 0.327 | | 0.783 | | 0.460 | | 0.233 | | 0.271 | | 0.402 | | 0.345 | | 0.204 | | .937^**^ | | .887^*^ | | | -0.573 | | -0.514 | | .906^*^ | |
| **GLTI** | -0.505 | | -0.658 | | -0.727 | | .915^*^ | | 0.272 | | 0.697 | | 0.353 | | .883^*^ | | 0.705 | | 0.269 | | 0.239 | | 0.643 | | 0.597 | | 0.459 | | 0.802 | | 0.759 | | | -0.703 | | -0.669 | | 0.756 | |
| **GLUL** | -0.236 | | -0.379 | | -0.365 | | 0.603 | | 0.019 | | 0.168 | | 0.030 | | 0.507 | | 0.088 | | -0.113 | | 0.040 | | 0.068 | | -0.052 | | -0.188 | | .992^**^ | | .995^**^ | | | -0.498 | | -0.464 | | .955^**^ | |
| **argD** | 0.564 | | 0.476 | | 0.724 | | -0.510 | | -0.552 | | -0.797 | | -0.691 | | -.844^*^ | | -0.723 | | -0.594 | | -0.607 | | -0.627 | | -0.597 | | -0.585 | | -0.528 | | -0.491 | | | 0.703 | | 0.745 | | -0.623 | |
| **argE-1** | -0.437 | | -0.533 | | -0.650 | | 0.757 | | 0.285 | | 0.603 | | 0.374 | | .817^*^ | | 0.557 | | 0.255 | | 0.299 | | 0.457 | | 0.433 | | 0.293 | | .884^*^ | | .857^*^ | | | -0.651 | | -0.645 | | .883^*^ | |
| **argE-2** | -0.155 | | -0.313 | | -0.322 | | 0.637 | | -0.050 | | 0.166 | | -0.017 | | 0.496 | | 0.120 | | -0.159 | | -0.025 | | 0.056 | | -0.001 | | -0.181 | | .987^**^ | | .997^**^ | | | -0.452 | | -0.433 | | .939^**^ | |
| **argE-3** | -0.240 | | -0.373 | | -0.395 | | 0.654 | | 0.025 | | 0.236 | | 0.063 | | 0.561 | | 0.175 | | -0.084 | | 0.054 | | 0.132 | | 0.036 | | -0.113 | | .990^**^ | | .995^**^ | | | -0.537 | | -0.516 | | .956^**^ | |
| **argE-4** | .882^*^ | | .893^*^ | | .971^**^ | | -0.652 | | -0.736 | | -.890^*^ | | -0.773 | | -.982^**^ | | -0.756 | | -0.708 | | -0.701 | | -0.803 | | -0.598 | | -0.660 | | -0.618 | | -0.530 | | | .842^*^ | | 0.782 | | -0.675 | |
| **argE-5** | 0.586 | | 0.657 | | 0.791 | | -0.582 | | -0.591 | | -0.805 | | -0.659 | | -.856^*^ | | -0.726 | | -0.613 | | -0.559 | | -0.584 | | -0.659 | | -0.559 | | -0.606 | | -0.530 | | | 0.496 | | 0.479 | | -0.649 | |
| **argE-5** | 0.586 | | 0.657 | | 0.791 | | -0.582 | | -0.591 | | -0.805 | | -0.659 | | -.856^*^ | | -0.726 | | -0.613 | | -0.559 | | -0.584 | | -0.659 | | -0.559 | | -0.606 | | -0.530 | | | 0.496 | | 0.479 | | -0.649 | |
| **LOX2S0-1** | -0.451 | | -0.572 | | -0.577 | | 0.508 | | 0.347 | | 0.379 | | 0.336 | | 0.641 | | 0.223 | | 0.223 | | 0.341 | | 0.174 | | 0.094 | | -0.008 | | .946^**^ | | .904^*^ | | | -0.474 | | -0.423 | | .961^**^ | |
| **LOX2S0-2** | -0.349 | | -0.511 | | -0.601 | | .938^**^ | | 0.109 | | 0.622 | | 0.219 | | 0.804 | | 0.689 | | 0.133 | | 0.089 | | 0.594 | | 0.601 | | 0.421 | | 0.757 | | 0.736 | | | -0.642 | | -0.631 | | 0.689 | |
| **LOX2S0-3** | -0.794 | | -0.773 | | -.905^*^ | | 0.567 | | 0.735 | | .995^**^ | | .812^*^ | | .901^*^ | | .960^**^ | | .823^*^ | | 0.676 | | .957^**^ | | .887^*^ | | .954^**^ | | 0.170 | | 0.074 | | | -0.672 | | -0.640 | | 0.223 | |
| **LOX2S0-4** | 0.610 | | 0.696 | | 0.811 | | -0.592 | | -0.606 | | -0.810 | | -0.662 | | -.863^*^ | | -0.726 | | -0.622 | | -0.563 | | -0.593 | | -0.659 | | -0.562 | | -0.613 | | -0.531 | | | 0.493 | | 0.465 | | -0.650 | |
| **LOX1-5** | -0.061 | | -0.238 | | -0.217 | | 0.587 | | -0.146 | | 0.044 | | -0.129 | | 0.388 | | 0.007 | | -0.266 | | -0.123 | | -0.056 | | -0.099 | | -0.295 | | .970^**^ | | .989^**^ | | | -0.359 | | -0.337 | | .906^*^ | |
| **LCAT3** | 0.804 | | .822^*^ | | .928^**^ | | -0.722 | | -0.639 | | -.872^*^ | | -0.704 | | -.988^**^ | | -0.771 | | -0.623 | | -0.620 | | -0.789 | | -0.613 | | -0.636 | | -0.673 | | -0.601 | | | .859^*^ | | .816^*^ | | -0.717 | |
| **DOX** | 0.300 | | 0.480 | | 0.531 | | -0.409 | | -0.402 | | -0.557 | | -0.418 | | -0.557 | | -0.521 | | -0.437 | | -0.321 | | -0.295 | | -0.562 | | -0.360 | | -0.444 | | -0.366 | | | 0.035 | | 0.003 | | -0.438 | |
| **AOS** | -0.064 | | -0.229 | | -0.216 | | 0.590 | | -0.150 | | 0.046 | | -0.127 | | 0.392 | | 0.010 | | -0.270 | | -0.121 | | -0.045 | | -0.104 | | -0.290 | | .967^**^ | | .990^**^ | | | -0.386 | | -0.368 | | .907^*^ | |
|  | | **SUS-1** | | **SUS-2** | | **SUS-3** | | **SUS-4** | | **SUS-5** | | **SUS-6** | | **SUS-7** | | **SUS-8** | **SUS-9** | **SUS-10** | | **UGP2-1** | | **UGP2-2** | | **GPI** | | **PFK-1** | | **PFK-2** | | **ALDO** | | **GADPH** | **PK-1** | | **PK-2** | | **PK-3** | |  |
| **Raffinose** | | -0.705 | | -0.688 | | -.821^*^ | | -0.426 | | -.878^*^ | | -.866^*^ | | -.889^*^ | | 0.567 | 0.504 | 0.595 | | 0.659 | | 0.770 | | -0.425 | | -0.052 | | -0.374 | | -0.288 | | -0.678 | -0.328 | | .899^*^ | | 0.606 | |  |
| **Stachyose** | | -0.783 | | -0.692 | | -0.723 | | -0.578 | | -0.694 | | -0.794 | | -0.719 | | 0.417 | 0.312 | 0.375 | | 0.784 | | 0.660 | | -0.493 | | -0.227 | | -0.486 | | -0.452 | | -0.599 | -0.538 | | .917^**^ | | 0.423 | |  |
| **Manninotriose** | | -.887^*^ | | -.812^*^ | | -.861^*^ | | -0.605 | | -.833^*^ | | -.918^**^ | | -.857^*^ | | 0.675 | 0.588 | 0.547 | | .841^*^ | | .867^*^ | | -0.530 | | -0.208 | | -0.502 | | -0.476 | | -0.705 | -0.587 | | .904^*^ | | 0.666 | |  |
| **D-Glucose 6-phosphate** | | .818^*^ | | 0.564 | | 0.322 | | 0.809 | | 0.055 | | 0.310 | | 0.123 | | -0.494 | -0.420 | -0.361 | | -0.577 | | -0.562 | | 0.483 | | 0.593 | | 0.589 | | 0.778 | | 0.282 | .988^**^ | | -0.610 | | -0.458 | |  |
| **Citric Acid** | | 0.533 | | 0.630 | | .845^*^ | | 0.181 | | .978^**^ | | .913^*^ | | .963^**^ | | -0.492 | -0.435 | -0.398 | | -0.651 | | -0.682 | | 0.303 | | -0.161 | | 0.198 | | 0.046 | | 0.651 | 0.064 | | -0.666 | | -0.496 | |  |
| **Shikimic acid** | | .875^*^ | | .954^**^ | | .951^**^ | | 0.479 | | .828^*^ | | .883^*^ | | .872^*^ | | -0.728 | -0.659 | -0.521 | | -0.806 | | -.924^**^ | | 0.333 | | 0.038 | | 0.312 | | 0.352 | | 0.534 | 0.646 | | -0.746 | | -0.692 | |  |
| **13(S)-HPODE** | | 0.613 | | 0.714 | | .901^*^ | | 0.228 | | .996^**^ | | .938^**^ | | .990^**^ | | -0.643 | -0.597 | -0.505 | | -0.668 | | -0.800 | | 0.312 | | -0.141 | | 0.213 | | 0.097 | | 0.666 | 0.164 | | -0.654 | | -0.638 | |  |
| **9,10,13-TriHOME** | | .982^**^ | | .813^*^ | | 0.789 | | 0.756 | | 0.705 | | .853^*^ | | 0.738 | | -0.811 | -0.732 | -0.626 | | -.848^*^ | | -.932^**^ | | 0.619 | | 0.384 | | 0.620 | | 0.654 | | 0.714 | 0.772 | | -.872^*^ | | -0.786 | |  |
| **13(S)-HOTrE** | | .832^*^ | | .978^**^ | | .869^*^ | | 0.434 | | 0.622 | | 0.694 | | 0.693 | | -0.648 | -0.587 | -0.427 | | -0.709 | | -.845^*^ | | 0.173 | | 0.006 | | 0.194 | | 0.326 | | 0.291 | 0.772 | | -0.602 | | -0.595 | |  |
| **L-Tryptophan** | | 0.547 | | 0.756 | | .932^**^ | | 0.107 | | .992^**^ | | .909^*^ | | .994^**^ | | -0.514 | -0.462 | -0.372 | | -0.654 | | -0.727 | | 0.170 | | -0.280 | | 0.073 | | -0.033 | | 0.536 | 0.125 | | -0.595 | | -0.500 | |  |
| **L-Valine** | | 0.497 | | 0.555 | | 0.796 | | 0.173 | | .970^**^ | | .887^*^ | | .944^**^ | | -0.582 | -0.547 | -0.498 | | -0.573 | | -0.705 | | 0.333 | | -0.136 | | 0.218 | | 0.053 | | 0.701 | 0.001 | | -0.615 | | -0.596 | |  |
| **L-Saccharopine** | | 0.797 | | .959^**^ | | .896^*^ | | 0.402 | | 0.687 | | 0.692 | | 0.761 | | -0.624 | -0.577 | -0.595 | | -0.599 | | -.858^*^ | | 0.146 | | -0.053 | | 0.167 | | 0.273 | | 0.315 | 0.684 | | -0.760 | | -0.631 | |  |
| **L-Alanine** | | 0.711 | | .946^**^ | | 0.801 | | 0.296 | | 0.508 | | 0.579 | | 0.584 | | -0.478 | -0.413 | -0.206 | | -0.655 | | -0.695 | | 0.015 | | -0.102 | | 0.043 | | 0.199 | | 0.101 | 0.727 | | -0.423 | | -0.399 | |  |
| **L-Arginine** | | 0.661 | | .989^**^ | | .947^**^ | | 0.164 | | 0.732 | | 0.685 | | 0.802 | | -0.528 | -0.487 | -0.412 | | -0.568 | | -0.784 | | -0.059 | | -0.295 | | -0.064 | | 0.033 | | 0.173 | 0.536 | | -0.552 | | -0.504 | |  |
| **E2.4.1.82-1** | | 0.670 | | 0.021 | | -0.022 | | .951^**^ | | 0.053 | | 0.367 | | 0.027 | | -0.528 | -0.454 | -0.348 | | -0.579 | | -0.395 | | .965^**^ | | .966^**^ | | .993^**^ | | .969^**^ | | 0.763 | 0.594 | | -0.566 | | -0.500 | |  |
| **E2.4.1.82-2** | | 0.601 | | -0.070 | | -0.125 | | .926^**^ | | -0.047 | | 0.264 | | -0.075 | | -0.512 | -0.453 | -0.351 | | -0.480 | | -0.336 | | .942^**^ | | .987^**^ | | .977^**^ | | .963^**^ | | 0.715 | 0.561 | | -0.481 | | -0.489 | |  |
| **E2.4.1.67-1** | | -0.779 | | -0.564 | | -0.589 | | -0.664 | | -0.579 | | -0.619 | | -0.607 | | .838^*^ | .829^*^ | .948^**^ | | 0.441 | | .867^*^ | | -0.553 | | -0.368 | | -0.561 | | -0.582 | | -0.677 | -0.566 | | .868^*^ | | .908^*^ | |  |
| **E2.4.1.67-2** | | -0.741 | | -0.535 | | -0.560 | | -0.624 | | -0.551 | | -0.579 | | -0.577 | | .890^*^ | .897^*^ | .974^**^ | | 0.382 | | .874^*^ | | -0.522 | | -0.349 | | -0.526 | | -0.555 | | -0.654 | -0.538 | | 0.774 | | .954^**^ | |  |
| **INV** | | 0.678 | | 0.046 | | 0.076 | | .910^*^ | | 0.222 | | 0.491 | | 0.182 | | -0.634 | -0.570 | -0.450 | | -0.604 | | -0.489 | | .996^**^ | | .901^*^ | | .990^**^ | | .916^*^ | | .887^*^ | 0.487 | | -0.600 | | -0.615 | |  |
| **SUS-1** | | 1 | | 0.754 | | 0.685 | | .841^*^ | | 0.577 | | 0.780 | | 0.611 | | -.815^*^ | -0.732 | -0.582 | | -.853^*^ | | -.893^*^ | | 0.682 | | 0.513 | | 0.700 | | 0.760 | | 0.702 | .856^*^ | | -.819^*^ | | -0.774 | |  |
| **SUS-2** | | 0.754 | | 1 | | .940^**^ | | 0.292 | | 0.721 | | 0.728 | | 0.789 | | -0.586 | -0.530 | -0.414 | | -0.666 | | -.825^*^ | | 0.063 | | -0.163 | | 0.064 | | 0.166 | | 0.256 | 0.636 | | -0.599 | | -0.547 | |  |
| **SUS-3** | | 0.685 | | .940^**^ | | 1 | | 0.198 | | .910^*^ | | .861^*^ | | .949^**^ | | -0.601 | -0.550 | -0.439 | | -0.681 | | -.837^*^ | | 0.104 | | -0.253 | | 0.053 | | 0.057 | | 0.408 | 0.402 | | -0.621 | | -0.576 | |  |
| **SUS-4** | | .841^*^ | | 0.292 | | 0.198 | | 1 | | 0.170 | | 0.467 | | 0.176 | | -0.661 | -0.585 | -0.501 | | -0.660 | | -0.596 | | .898^*^ | | .891^*^ | | .947^**^ | | .988^**^ | | 0.729 | 0.795 | | -0.711 | | -0.639 | |  |
| **SUS-5** | | 0.577 | | 0.721 | | .910^*^ | | 0.170 | | 1 | | .933^**^ | | .993^**^ | | -0.591 | -0.543 | -0.433 | | -0.670 | | -0.765 | | 0.259 | | -0.200 | | 0.156 | | 0.037 | | 0.622 | 0.131 | | -0.608 | | -0.578 | |  |
| **SUS-6** | | 0.780 | | 0.728 | | .861^*^ | | 0.467 | | .933^**^ | | 1 | | .923^**^ | | -0.692 | -0.609 | -0.407 | | -.882^*^ | | -.828^*^ | | 0.533 | | 0.118 | | 0.450 | | 0.350 | | 0.784 | 0.371 | | -0.703 | | -0.643 | |  |
| **SUS-7** | | 0.611 | | 0.789 | | .949^**^ | | 0.176 | | .993^**^ | | .923^**^ | | 1 | | -0.601 | -0.553 | -0.461 | | -0.667 | | -0.797 | | 0.215 | | -0.224 | | 0.125 | | 0.037 | | 0.571 | 0.200 | | -0.636 | | -0.591 | |  |
| **SUS-8** | | -.815^*^ | | -0.586 | | -0.601 | | -0.661 | | -0.591 | | -0.692 | | -0.601 | | 1 | .989^**^ | 0.800 | | 0.584 | | .928^**^ | | -0.607 | | -0.403 | | -0.585 | | -0.610 | | -0.731 | -0.596 | | 0.593 | | .983^**^ | |  |
| **SUS-9** | | -0.732 | | -0.530 | | -0.550 | | -0.585 | | -0.543 | | -0.609 | | -0.553 | | .989^**^ | 1 | .837^*^ | | 0.462 | | .893^*^ | | -0.532 | | -0.350 | | -0.511 | | -0.543 | | -0.667 | -0.530 | | 0.524 | | .986^**^ | |  |
| **SUS-10** | | -0.582 | | -0.414 | | -0.439 | | -0.501 | | -0.433 | | -0.407 | | -0.461 | | 0.800 | .837^*^ | 1 | | 0.164 | | 0.761 | | -0.392 | | -0.270 | | -0.405 | | -0.445 | | -0.522 | -0.423 | | 0.681 | | .895^*^ | |  |
| **UGP2-1** | | -.853^*^ | | -0.666 | | -0.681 | | -0.660 | | -0.670 | | -.882^*^ | | -0.667 | | 0.584 | 0.462 | 0.164 | | 1 | | 0.700 | | -0.655 | | -0.380 | | -0.617 | | -0.577 | | -0.720 | -0.603 | | 0.634 | | 0.483 | |  |
| **UGP2-2** | | -.893^*^ | | -.825^*^ | | -.837^*^ | | -0.596 | | -0.765 | | -.828^*^ | | -0.797 | | .928^**^ | .893^*^ | 0.761 | | 0.700 | | 1 | | -0.480 | | -0.214 | | -0.460 | | -0.497 | | -0.666 | -0.653 | | 0.745 | | .915^*^ | |  |
| **GPI** | | 0.682 | | 0.063 | | 0.104 | | .898^*^ | | 0.259 | | 0.533 | | 0.215 | | -0.607 | -0.532 | -0.392 | | -0.655 | | -0.480 | | 1 | | .881^*^ | | .987^**^ | | .898^*^ | | .901^*^ | 0.471 | | -0.603 | | -0.578 | |  |
| **PFK-1** | | 0.513 | | -0.163 | | -0.253 | | .891^*^ | | -0.200 | | 0.118 | | -0.224 | | -0.403 | -0.350 | -0.270 | | -0.380 | | -0.214 | | .881^*^ | | 1 | | .935^**^ | | .945^**^ | | 0.598 | 0.550 | | -0.392 | | -0.382 | |  |
| **PFK-2** | | 0.700 | | 0.064 | | 0.053 | | .947^**^ | | 0.156 | | 0.450 | | 0.125 | | -0.585 | -0.511 | -0.405 | | -0.617 | | -0.460 | | .987^**^ | | .935^**^ | | 1 | | .954^**^ | | .832^*^ | 0.565 | | -0.615 | | -0.562 | |  |
| **ALDO** | | 0.760 | | 0.166 | | 0.057 | | .988^**^ | | 0.037 | | 0.350 | | 0.037 | | -0.610 | -0.543 | -0.445 | | -0.577 | | -0.497 | | .898^*^ | | .945^**^ | | .954^**^ | | 1 | | 0.682 | 0.759 | | -0.603 | | -0.584 | |  |

|  | **SUS-1** | **SUS-2** | **SUS-3** | **SUS-4** | **SUS-5** | **SUS-6** | **SUS-7** | **SUS-8** | **SUS-9** | **SUS-10** | **UGP2-1** | **UGP2-2** | **GPI** | **PFK-1** | **PFK-2** | **ALDO** | **GADPH** | **PK-1** | **PK-2** | **PK-3** |
| --- | --- | --- | --- | --- | --- | --- | --- | --- | --- | --- | --- | --- | --- | --- | --- | --- | --- | --- | --- | --- |
| **GADPH** | 0.702 | 0.256 | 0.408 | 0.729 | 0.622 | 0.784 | 0.571 | -0.731 | -0.667 | -0.522 | -0.720 | -0.666 | .901^*^ | 0.598 | .832^*^ | 0.682 | 1 | 0.310 | -0.681 | -0.714 |
| **PK-1** | .856^*^ | 0.636 | 0.402 | 0.795 | 0.131 | 0.371 | 0.200 | -0.596 | -0.530 | -0.423 | -0.603 | -0.653 | 0.471 | 0.550 | 0.565 | 0.759 | 0.310 | 1 | -0.595 | -0.552 |
| **PK-2** | -.819^*^ | -0.599 | -0.621 | -0.711 | -0.608 | -0.703 | -0.636 | 0.593 | 0.524 | 0.681 | 0.634 | 0.745 | -0.603 | -0.392 | -0.615 | -0.603 | -0.681 | -0.595 | 1 | 0.646 |
| **PK-3** | -0.774 | -0.547 | -0.576 | -0.639 | -0.578 | -0.643 | -0.591 | .983^**^ | .986^**^ | .895^*^ | 0.483 | .915^*^ | -0.578 | -0.382 | -0.562 | -0.584 | -0.714 | -0.552 | 0.646 | 1 |
| **PDAH** | 0.693 | 0.051 | -0.003 | .962^**^ | 0.058 | 0.367 | 0.037 | -0.581 | -0.514 | -0.406 | -0.568 | -0.439 | .958^**^ | .966^**^ | .991^**^ | .981^**^ | 0.762 | 0.626 | -0.570 | -0.556 |
| **CS** | -.909^*^ | -0.676 | -0.690 | -0.760 | -0.675 | -.855^*^ | -0.689 | 0.601 | 0.490 | 0.422 | .903^*^ | 0.758 | -0.695 | -0.440 | -0.685 | -0.656 | -0.756 | -0.660 | .900^*^ | 0.574 |
| **ACO-1** | 0.530 | -0.144 | -0.231 | .899^*^ | -0.179 | 0.141 | -0.202 | -0.419 | -0.363 | -0.279 | -0.400 | -0.234 | .890^*^ | 1.000^**^ | .942^**^ | .951^**^ | 0.613 | 0.559 | -0.407 | -0.396 |
| **ACO-2** | 0.533 | -0.146 | -0.220 | .900^*^ | -0.152 | 0.162 | -0.179 | -0.435 | -0.381 | -0.299 | -0.405 | -0.248 | .905^*^ | .998^**^ | .951^**^ | .950^**^ | 0.640 | 0.541 | -0.421 | -0.416 |
| **ACO-3** | 0.538 | -0.137 | -0.219 | .904^*^ | -0.163 | 0.154 | -0.187 | -0.435 | -0.380 | -0.297 | -0.404 | -0.249 | .897^*^ | .999^**^ | .947^**^ | .953^**^ | 0.627 | 0.558 | -0.419 | -0.414 |
| **IDH3** | 0.710 | 0.101 | 0.135 | .912^*^ | 0.277 | 0.552 | 0.237 | -0.618 | -0.540 | -0.407 | -0.675 | -0.505 | .999^**^ | .878^*^ | .988^**^ | .907^*^ | .902^*^ | 0.501 | -0.633 | -0.589 |
| **OGDH** | -.922^**^ | -0.771 | -0.791 | -0.666 | -0.756 | -.892^*^ | -0.769 | .909^*^ | .847^*^ | 0.583 | .852^*^ | .954^**^ | -0.612 | -0.329 | -0.576 | -0.581 | -0.760 | -0.657 | 0.677 | .850^*^ |
| **trpE** | 0.744 | .995^**^ | .948^**^ | 0.282 | 0.740 | 0.728 | 0.809 | -0.582 | -0.531 | -0.464 | -0.634 | -.831^*^ | 0.055 | -0.180 | 0.055 | 0.150 | 0.263 | 0.611 | -0.642 | -0.560 |
| **trpD** | -.898^*^ | -.823^*^ | -.880^*^ | -0.585 | -.865^*^ | -.963^**^ | -.877^*^ | .821^*^ | 0.746 | 0.536 | .884^*^ | .937^**^ | -0.552 | -0.208 | -0.502 | -0.475 | -0.752 | -0.578 | 0.744 | 0.771 |
| **ilvA** | 0.528 | -0.151 | -0.227 | .898^*^ | -0.161 | 0.153 | -0.188 | -0.430 | -0.376 | -0.295 | -0.398 | -0.241 | .901^*^ | .999^**^ | .948^**^ | .948^**^ | 0.633 | 0.541 | -0.415 | -0.410 |
| **ilvH** | -.883^*^ | -0.652 | -0.669 | -0.704 | -0.656 | -0.788 | -0.664 | .983^**^ | .948^**^ | 0.714 | 0.721 | .949^**^ | -0.657 | -0.421 | -0.629 | -0.642 | -0.779 | -0.641 | 0.640 | .945^**^ |
| **ilvE-1** | 0.522 | -0.155 | -0.238 | .896^*^ | -0.179 | 0.137 | -0.204 | -0.420 | -0.367 | -0.287 | -0.389 | -0.231 | .892^*^ | 1.000^**^ | .942^**^ | .948^**^ | 0.617 | 0.547 | -0.406 | -0.400 |
| **ilvE-2** | 0.783 | 0.195 | 0.190 | .958^**^ | 0.276 | 0.564 | 0.252 | -0.643 | -0.560 | -0.435 | -0.713 | -0.558 | .983^**^ | .884^*^ | .990^**^ | .945^**^ | .869^*^ | 0.614 | -0.688 | -0.613 |
| **ilvE-3** | .830^*^ | 0.357 | 0.427 | .869^*^ | 0.546 | 0.737 | 0.524 | -0.810 | -0.749 | -0.664 | -0.714 | -0.758 | .919^**^ | 0.695 | .893^*^ | .819^*^ | .956^**^ | 0.535 | -0.801 | -0.809 |
| **IMDH** | .854^*^ | 0.337 | 0.280 | .966^**^ | 0.285 | 0.593 | 0.280 | -0.601 | -0.498 | -0.349 | -0.804 | -0.581 | .918^**^ | .837^*^ | .942^**^ | .939^**^ | 0.784 | 0.740 | -0.713 | -0.551 |
| **GLTI** | .952^**^ | 0.576 | 0.441 | .941^**^ | 0.308 | 0.576 | 0.342 | -0.741 | -0.661 | -0.526 | -0.756 | -0.754 | 0.748 | 0.710 | 0.802 | .900^*^ | 0.636 | .929^**^ | -0.739 | -0.700 |
| **GLUL** | 0.608 | -0.060 | -0.111 | .934^**^ | -0.032 | 0.270 | -0.057 | -0.487 | -0.425 | -0.373 | -0.476 | -0.335 | .941^**^ | .982^**^ | .980^**^ | .963^**^ | 0.717 | 0.562 | -0.543 | -0.478 |
| **argD** | -.855^*^ | -0.659 | -0.676 | -0.649 | -0.660 | -0.799 | -0.663 | .956^**^ | .915^*^ | 0.602 | 0.758 | .919^**^ | -0.621 | -0.375 | -0.583 | -0.592 | -0.749 | -0.614 | 0.542 | .891^*^ |
| **argE-1** | .896^*^ | 0.420 | 0.351 | .947^**^ | 0.326 | 0.612 | 0.327 | -0.804 | -0.730 | -0.492 | -0.773 | -0.725 | .878^*^ | 0.794 | .896^*^ | .928^**^ | 0.785 | 0.788 | -0.628 | -0.743 |
| **argE-2** | 0.615 | -0.045 | -0.125 | .935^**^ | -0.077 | 0.244 | -0.098 | -0.508 | -0.448 | -0.336 | -0.484 | -0.337 | .923^**^ | .991^**^ | .967^**^ | .974^**^ | 0.677 | 0.608 | -0.469 | -0.480 |
| **argE-3** | 0.668 | 0.018 | -0.050 | .957^**^ | -0.001 | 0.306 | -0.019 | -0.571 | -0.511 | -0.418 | -0.513 | -0.414 | .938^**^ | .977^**^ | .979^**^ | .984^**^ | 0.723 | 0.631 | -0.543 | -0.551 |
| **argE-4** | -.950^**^ | -0.731 | -0.755 | -0.766 | -0.737 | -.879^*^ | -0.756 | 0.777 | 0.694 | 0.632 | .845^*^ | .894^*^ | -0.688 | -0.417 | -0.675 | -0.661 | -0.798 | -0.680 | .923^**^ | 0.767 |
| **argE-5** | -.878^*^ | -0.660 | -0.666 | -0.691 | -0.651 | -.868^*^ | -0.647 | 0.733 | 0.633 | 0.270 | .967^**^ | 0.779 | -0.685 | -0.426 | -0.646 | -0.624 | -0.753 | -0.639 | 0.567 | 0.626 |
| **argE-5** | -.878^*^ | -0.660 | -0.666 | -0.691 | -0.651 | -.868^*^ | -0.647 | 0.733 | 0.633 | 0.270 | .967^**^ | 0.779 | -0.685 | -0.426 | -0.646 | -0.624 | -0.753 | -0.639 | 0.567 | 0.626 |
| **LOX2S0-1** | 0.701 | 0.120 | 0.156 | .882^*^ | 0.296 | 0.586 | 0.253 | -0.534 | -0.438 | -0.266 | -0.749 | -0.456 | .982^**^ | .843^*^ | .965^**^ | .871^*^ | .882^*^ | 0.486 | -0.607 | -0.485 |
| **LOX2S0-2** | .898^*^ | 0.537 | 0.354 | .906^*^ | 0.173 | 0.444 | 0.216 | -0.728 | -0.665 | -0.507 | -0.655 | -0.705 | 0.669 | 0.707 | 0.741 | .885^*^ | 0.515 | .961^**^ | -0.617 | -0.681 |
| **LOX2S0-3** | .838^*^ | .978^**^ | .961^**^ | 0.413 | 0.809 | .842^*^ | .861^*^ | -0.692 | -0.629 | -0.503 | -0.759 | -.904^*^ | 0.241 | -0.038 | 0.226 | 0.284 | 0.449 | 0.639 | -0.707 | -0.657 |
| **LOX2S0-4** | -.883^*^ | -0.664 | -0.670 | -0.698 | -0.656 | -.875^*^ | -0.652 | 0.704 | 0.598 | 0.253 | .981^**^ | 0.767 | -0.689 | -0.428 | -0.652 | -0.627 | -0.753 | -0.641 | 0.595 | 0.599 |
| **LOX1-5** | 0.516 | -0.161 | -0.245 | .891^*^ | -0.187 | 0.135 | -0.212 | -0.399 | -0.342 | -0.254 | -0.399 | -0.213 | .890^*^ | .999^**^ | .940^**^ | .944^**^ | 0.610 | 0.543 | -0.393 | -0.375 |
| **LCAT3** | -.980^**^ | -0.717 | -0.706 | -.827^*^ | -0.662 | -.830^*^ | -0.684 | .840^*^ | 0.765 | 0.671 | .828^*^ | .916^*^ | -0.721 | -0.496 | -0.721 | -0.737 | -0.791 | -0.759 | .888^*^ | .824^*^ |
| **DOX** | -0.601 | -0.462 | -0.456 | -0.454 | -0.446 | -0.678 | -0.427 | 0.347 | 0.227 | -0.220 | .898^*^ | 0.412 | -0.502 | -0.300 | -0.456 | -0.414 | -0.507 | -0.436 | 0.238 | 0.191 |
| **AOS** | 0.518 | -0.158 | -0.244 | .894^*^ | -0.188 | 0.127 | -0.212 | -0.420 | -0.368 | -0.292 | -0.379 | -0.229 | .886^*^ | 1.000^**^ | .939^**^ | .947^**^ | 0.609 | 0.548 | -0.402 | -0.401 |

|  | **PDAH** | **CS** | **ACO-1** | **ACO-2** | **ACO-3** | **IDH3** | **OGDH** | **trpE** | **trpD** | **ilvA** | **ilvH** | **ilvE-1** | **ilvE-2** | **ilvE-3** | **IMDH** | **GLTI** | **GLUL** | **argD** | **argE-1** | **argE-2** |
| --- | --- | --- | --- | --- | --- | --- | --- | --- | --- | --- | --- | --- | --- | --- | --- | --- | --- | --- | --- | --- |
| **Raffinose** | -0.288 | .847^*^ | -0.072 | -0.095 | -0.087 | -0.452 | 0.709 | -0.732 | .830^*^ | -0.087 | 0.627 | -0.072 | -0.479 | -0.697 | -0.494 | -0.505 | -0.236 | 0.564 | -0.437 | -0.155 |
| **Stachyose** | -0.421 | .950^**^ | -0.245 | -0.253 | -0.252 | -0.527 | 0.659 | -0.717 | 0.779 | -0.247 | 0.526 | -0.238 | -0.586 | -0.662 | -0.675 | -0.658 | -0.379 | 0.476 | -0.533 | -0.313 |
| **Manninotriose** | -0.442 | .953^**^ | -0.229 | -0.243 | -0.241 | -0.561 | .860^*^ | -.829^*^ | .938^**^ | -0.235 | 0.761 | -0.223 | -0.615 | -0.762 | -0.679 | -0.727 | -0.365 | 0.724 | -0.650 | -0.322 |
| **D-Glucose 6-phosphate** | 0.649 | -0.664 | 0.601 | 0.581 | 0.598 | 0.514 | -0.571 | 0.544 | -0.505 | 0.581 | -0.547 | 0.588 | 0.628 | 0.513 | 0.760 | .915^*^ | 0.603 | -0.510 | 0.757 | 0.637 |
| **Citric Acid** | 0.087 | -0.707 | -0.141 | -0.112 | -0.125 | 0.320 | -0.674 | 0.660 | -0.810 | -0.121 | -0.565 | -0.140 | 0.311 | 0.564 | 0.310 | 0.272 | 0.019 | -0.552 | 0.285 | -0.050 |
| **Shikimic acid** | 0.277 | -.833^*^ | 0.060 | 0.067 | 0.071 | 0.367 | -.904^*^ | .953^**^ | -.951^**^ | 0.060 | -0.803 | 0.052 | 0.439 | 0.609 | 0.538 | 0.697 | 0.168 | -0.797 | 0.603 | 0.166 |
| **13(S)-HPODE** | 0.118 | -0.698 | -0.119 | -0.092 | -0.102 | 0.330 | -0.782 | 0.736 | -.881^*^ | -0.101 | -0.699 | -0.119 | 0.330 | 0.604 | 0.327 | 0.353 | 0.030 | -0.691 | 0.374 | -0.017 |
| **9,10,13-TriHOME** | 0.594 | -.932^**^ | 0.403 | 0.411 | 0.414 | 0.650 | -.933^**^ | .815^*^ | -.944^**^ | 0.405 | -.879^*^ | 0.396 | 0.718 | .823^*^ | 0.783 | .883^*^ | 0.507 | -.844^*^ | .817^*^ | 0.496 |
| **13(S)-HOTrE** | 0.200 | -0.703 | 0.025 | 0.018 | 0.030 | 0.210 | -.814^*^ | .962^**^ | -.826^*^ | 0.014 | -0.715 | 0.012 | 0.313 | 0.423 | 0.460 | 0.705 | 0.088 | -0.723 | 0.557 | 0.120 |
| **L-Tryptophan** | -0.024 | -0.663 | -0.259 | -0.235 | -0.244 | 0.192 | -0.710 | 0.775 | -.837^*^ | -0.244 | -0.587 | -0.261 | 0.200 | 0.465 | 0.233 | 0.269 | -0.113 | -0.594 | 0.255 | -0.159 |
| **L-Valine** | 0.109 | -0.624 | -0.116 | -0.083 | -0.098 | 0.344 | -0.686 | 0.587 | -0.792 | -0.092 | -0.624 | -0.112 | 0.318 | 0.603 | 0.271 | 0.239 | 0.040 | -0.607 | 0.299 | -0.025 |
| **L-Saccharopine** | 0.155 | -0.730 | -0.035 | -0.034 | -0.025 | 0.186 | -0.750 | .976^**^ | -0.803 | -0.039 | -0.667 | -0.043 | 0.285 | 0.461 | 0.402 | 0.643 | 0.068 | -0.627 | 0.457 | 0.056 |
| **L-Alanine** | 0.061 | -0.582 | -0.085 | -0.100 | -0.085 | 0.051 | -0.691 | .915^*^ | -0.706 | -0.103 | -0.559 | -0.101 | 0.161 | 0.221 | 0.345 | 0.597 | -0.052 | -0.597 | 0.433 | -0.001 |
| **L-Arginine** | -0.082 | -0.593 | -0.277 | -0.277 | -0.269 | -0.022 | -0.700 | .992^**^ | -0.767 | -0.282 | -0.581 | -0.287 | 0.068 | 0.265 | 0.204 | 0.459 | -0.188 | -0.585 | 0.293 | -0.181 |
| **E2.4.1.82-1** | .996^**^ | -0.642 | .971^**^ | .976^**^ | .974^**^ | .966^**^ | -0.517 | 0.008 | -0.432 | .974^**^ | -0.572 | .970^**^ | .974^**^ | .840^*^ | .937^**^ | 0.802 | .992^**^ | -0.528 | .884^*^ | .987^**^ |
| **E2.4.1.82-2** | .992^**^ | -0.538 | .990^**^ | .994^**^ | .993^**^ | .940^**^ | -0.452 | -0.085 | -0.342 | .993^**^ | -0.536 | .991^**^ | .942^**^ | 0.796 | .887^*^ | 0.759 | .995^**^ | -0.491 | .857^*^ | .997^**^ |
| **E2.4.1.67-1** | -0.543 | 0.689 | -0.381 | -0.400 | -0.398 | -0.575 | 0.740 | -0.607 | 0.726 | -0.395 | 0.808 | -0.385 | -0.616 | -.814^*^ | -0.573 | -0.703 | -0.498 | 0.703 | -0.651 | -0.452 |
| **E2.4.1.67-2** | -0.516 | 0.596 | -0.361 | -0.381 | -0.379 | -0.539 | 0.745 | -0.573 | 0.703 | -0.376 | .840^*^ | -0.366 | -0.574 | -0.787 | -0.514 | -0.669 | -0.464 | 0.745 | -0.645 | -0.433 |
| **INV** | .970^**^ | -0.663 | .908^*^ | .923^**^ | .916^*^ | .994^**^ | -0.602 | 0.041 | -0.528 | .919^**^ | -0.668 | .911^*^ | .980^**^ | .921^**^ | .906^*^ | 0.756 | .955^**^ | -0.623 | .883^*^ | .939^**^ |
| **SUS-1** | 0.693 | -.909^*^ | 0.530 | 0.533 | 0.538 | 0.710 | -.922^**^ | 0.744 | -.898^*^ | 0.528 | -.883^*^ | 0.522 | 0.783 | .830^*^ | .854^*^ | .952^**^ | 0.608 | -.855^*^ | .896^*^ | 0.615 |
| **SUS-2** | 0.051 | -0.676 | -0.144 | -0.146 | -0.137 | 0.101 | -0.771 | .995^**^ | -.823^*^ | -0.151 | -0.652 | -0.155 | 0.195 | 0.357 | 0.337 | 0.576 | -0.060 | -0.659 | 0.420 | -0.045 |
| **SUS-3** | -0.003 | -0.690 | -0.231 | -0.220 | -0.219 | 0.135 | -0.791 | .948^**^ | -.880^*^ | -0.227 | -0.669 | -0.238 | 0.190 | 0.427 | 0.280 | 0.441 | -0.111 | -0.676 | 0.351 | -0.125 |
| **SUS-4** | .962^**^ | -0.760 | .899^*^ | .900^*^ | .904^*^ | .912^*^ | -0.666 | 0.282 | -0.585 | .898^*^ | -0.704 | .896^*^ | .958^**^ | .869^*^ | .966^**^ | .941^**^ | .934^**^ | -0.649 | .947^**^ | .935^**^ |
| **SUS-5** | 0.058 | -0.675 | -0.179 | -0.152 | -0.163 | 0.277 | -0.756 | 0.740 | -.865^*^ | -0.161 | -0.656 | -0.179 | 0.276 | 0.546 | 0.285 | 0.308 | -0.032 | -0.660 | 0.326 | -0.077 |
| **SUS-6** | 0.367 | -.855^*^ | 0.141 | 0.162 | 0.154 | 0.552 | -.892^*^ | 0.728 | -.963^**^ | 0.153 | -0.788 | 0.137 | 0.564 | 0.737 | 0.593 | 0.576 | 0.270 | -0.799 | 0.612 | 0.244 |
| **SUS-7** | 0.037 | -0.689 | -0.202 | -0.179 | -0.187 | 0.237 | -0.769 | 0.809 | -.877^*^ | -0.188 | -0.664 | -0.204 | 0.252 | 0.524 | 0.280 | 0.342 | -0.057 | -0.663 | 0.327 | -0.098 |
| **SUS-8** | -0.581 | 0.601 | -0.419 | -0.435 | -0.435 | -0.618 | .909^*^ | -0.582 | .821^*^ | -0.430 | .983^**^ | -0.420 | -0.643 | -0.810 | -0.601 | -0.741 | -0.487 | .956^**^ | -0.804 | -0.508 |
| **SUS-9** | -0.514 | 0.490 | -0.363 | -0.381 | -0.380 | -0.540 | .847^*^ | -0.531 | 0.746 | -0.376 | .948^**^ | -0.367 | -0.560 | -0.749 | -0.498 | -0.661 | -0.425 | .915^*^ | -0.730 | -0.448 |
| **SUS-10** | -0.406 | 0.422 | -0.279 | -0.299 | -0.297 | -0.407 | 0.583 | -0.464 | 0.536 | -0.295 | 0.714 | -0.287 | -0.435 | -0.664 | -0.349 | -0.526 | -0.373 | 0.602 | -0.492 | -0.336 |
| **UGP2-1** | -0.568 | .903^*^ | -0.400 | -0.405 | -0.404 | -0.675 | .852^*^ | -0.634 | .884^*^ | -0.398 | 0.721 | -0.389 | -0.713 | -0.714 | -0.804 | -0.756 | -0.476 | 0.758 | -0.773 | -0.484 |
| **UGP2-2** | -0.439 | 0.758 | -0.234 | -0.248 | -0.249 | -0.505 | .954^**^ | -.831^*^ | .937^**^ | -0.241 | .949^**^ | -0.231 | -0.558 | -0.758 | -0.581 | -0.754 | -0.335 | .919^**^ | -0.725 | -0.337 |
| **GPI** | .958^**^ | -0.695 | .890^*^ | .905^*^ | .897^*^ | .999^**^ | -0.612 | 0.055 | -0.552 | .901^*^ | -0.657 | .892^*^ | .983^**^ | .919^**^ | .918^**^ | 0.748 | .941^**^ | -0.621 | .878^*^ | .923^**^ |
| **PFK-1** | .966^**^ | -0.440 | 1.000^**^ | .998^**^ | .999^**^ | .878^*^ | -0.329 | -0.180 | -0.208 | .999^**^ | -0.421 | 1.000^**^ | .884^*^ | 0.695 | .837^*^ | 0.710 | .982^**^ | -0.375 | 0.794 | .991^**^ |
| **PFK-2** | .991^**^ | -0.685 | .942^**^ | .951^**^ | .947^**^ | .988^**^ | -0.576 | 0.055 | -0.502 | .948^**^ | -0.629 | .942^**^ | .990^**^ | .893^*^ | .942^**^ | 0.802 | .980^**^ | -0.583 | .896^*^ | .967^**^ |
| **ALDO** | .981^**^ | -0.656 | .951^**^ | .950^**^ | .953^**^ | .907^*^ | -0.581 | 0.150 | -0.475 | .948^**^ | -0.642 | .948^**^ | .945^**^ | .819^*^ | .939^**^ | .900^*^ | .963^**^ | -0.592 | .928^**^ | .974^**^ |
| **GADPH** | 0.762 | -0.756 | 0.613 | 0.640 | 0.627 | .902^*^ | -0.760 | 0.263 | -0.752 | 0.633 | -0.779 | 0.617 | .869^*^ | .956^**^ | 0.784 | 0.636 | 0.717 | -0.749 | 0.785 | 0.677 |
| **PK-1** | 0.626 | -0.660 | 0.559 | 0.541 | 0.558 | 0.501 | -0.657 | 0.611 | -0.578 | 0.541 | -0.641 | 0.547 | 0.614 | 0.535 | 0.740 | .929^**^ | 0.562 | -0.614 | 0.788 | 0.608 |

|  | **PDAH** | **CS** | **ACO-1** | **ACO-2** | **ACO-3** | **IDH3** | **OGDH** | **trpE** | **trpD** | **ilvA** | **ilvH** | **ilvE-1** | **ilvE-2** | **ilvE-3** | **IMDH** | **GLTI** | **GLUL** | **argD** | **argE-1** | **argE-2** |
| --- | --- | --- | --- | --- | --- | --- | --- | --- | --- | --- | --- | --- | --- | --- | --- | --- | --- | --- | --- | --- |
| **PK-2** | -0.570 | .900^*^ | -0.407 | -0.421 | -0.419 | -0.633 | 0.677 | -0.642 | 0.744 | -0.415 | 0.640 | -0.406 | -0.688 | -0.801 | -0.713 | -0.739 | -0.543 | 0.542 | -0.628 | -0.469 |
| **PK-3** | -0.556 | 0.574 | -0.396 | -0.416 | -0.414 | -0.589 | .850^*^ | -0.560 | 0.771 | -0.410 | .945^**^ | -0.400 | -0.613 | -0.809 | -0.551 | -0.700 | -0.478 | .891^*^ | -0.743 | -0.480 |
| **PDAH** | 1 | -0.633 | .971^**^ | .976^**^ | .975^**^ | .961^**^ | -0.547 | 0.037 | -0.450 | .974^**^ | -0.615 | .971^**^ | .972^**^ | .851^*^ | .933^**^ | .825^*^ | .990^**^ | -0.568 | .905^*^ | .990^**^ |
| **CS** | -0.633 | 1 | -0.458 | -0.467 | -0.466 | -0.724 | .811^*^ | -0.682 | .872^*^ | -0.461 | 0.710 | -0.451 | -0.776 | -.820^*^ | -.848^*^ | -.821^*^ | -0.573 | 0.676 | -0.762 | -0.534 |
| **ACO-1** | .971^**^ | -0.458 | 1 | .999^**^ | 1.000^**^ | .887^*^ | -0.349 | -0.161 | -0.230 | .999^**^ | -0.438 | 1.000^**^ | .894^*^ | 0.709 | .849^*^ | 0.723 | .985^**^ | -0.393 | 0.806 | .994^**^ |
| **ACO-2** | .976^**^ | -0.467 | .999^**^ | 1 | 1.000^**^ | .901^*^ | -0.362 | -0.161 | -0.245 | 1.000^**^ | -0.454 | 1.000^**^ | .904^*^ | 0.730 | .851^*^ | 0.719 | .989^**^ | -0.406 | 0.810 | .994^**^ |
| **ACO-3** | .975^**^ | -0.466 | 1.000^**^ | 1.000^**^ | 1 | .894^*^ | -0.362 | -0.153 | -0.243 | 1.000^**^ | -0.453 | 1.000^**^ | .900^*^ | 0.723 | .852^*^ | 0.727 | .988^**^ | -0.406 | .812^*^ | .995^**^ |
| **IDH3** | .961^**^ | -0.724 | .887^*^ | .901^*^ | .894^*^ | 1 | -0.632 | 0.093 | -0.575 | .897^*^ | -0.670 | .888^*^ | .990^**^ | .928^**^ | .933^**^ | 0.772 | .941^**^ | -0.632 | .890^*^ | .922^**^ |
| **OGDH** | -0.547 | .811^*^ | -0.349 | -0.362 | -0.362 | -0.632 | 1 | -0.754 | .973^**^ | -0.355 | .967^**^ | -0.344 | -0.672 | -0.806 | -0.704 | -0.800 | -0.435 | .973^**^ | -.828^*^ | -0.453 |
| **trpE** | 0.037 | -0.682 | -0.161 | -0.161 | -0.153 | 0.093 | -0.754 | 1 | -.817^*^ | -0.166 | -0.641 | -0.171 | 0.186 | 0.368 | 0.319 | 0.558 | -0.066 | -0.634 | 0.392 | -0.064 |
| **trpD** | -0.450 | .872^*^ | -0.230 | -0.245 | -0.243 | -0.575 | .973^**^ | -.817^*^ | 1 | -0.237 | .898^*^ | -0.225 | -0.614 | -0.775 | -0.661 | -0.730 | -0.342 | .899^*^ | -0.731 | -0.337 |
| **ilvA** | .974^**^ | -0.461 | .999^**^ | 1.000^**^ | 1.000^**^ | .897^*^ | -0.355 | -0.166 | -0.237 | 1 | -0.447 | 1.000^**^ | .900^*^ | 0.724 | .847^*^ | 0.716 | .988^**^ | -0.400 | 0.806 | .994^**^ |
| **ilvH** | -0.615 | 0.710 | -0.438 | -0.454 | -0.453 | -0.670 | .967^**^ | -0.641 | .898^*^ | -0.447 | 1 | -0.437 | -0.700 | -.844^*^ | -0.686 | -0.797 | -0.513 | .987^**^ | -.855^*^ | -0.534 |
| **ilvE-1** | .971^**^ | -0.451 | 1.000^**^ | 1.000^**^ | 1.000^**^ | .888^*^ | -0.344 | -0.171 | -0.225 | 1.000^**^ | -0.437 | 1 | .893^*^ | 0.712 | .843^*^ | 0.715 | .986^**^ | -0.390 | 0.801 | .993^**^ |
| **ilvE-2** | .972^**^ | -0.776 | .894^*^ | .904^*^ | .900^*^ | .990^**^ | -0.672 | 0.186 | -0.614 | .900^*^ | -0.700 | .893^*^ | 1 | .930^**^ | .970^**^ | .850^*^ | .947^**^ | -0.658 | .928^**^ | .933^**^ |
| **ilvE-3** | .851^*^ | -.820^*^ | 0.709 | 0.730 | 0.723 | .928^**^ | -0.806 | 0.368 | -0.775 | 0.724 | -.844^*^ | 0.712 | .930^**^ | 1 | .871^*^ | 0.803 | 0.807 | -0.787 | .883^*^ | 0.774 |
| **IMDH** | .933^**^ | -.848^*^ | .849^*^ | .851^*^ | .852^*^ | .933^**^ | -0.704 | 0.319 | -0.661 | .847^*^ | -0.686 | .843^*^ | .970^**^ | .871^*^ | 1 | .917^*^ | .895^*^ | -0.657 | .939^**^ | .892^*^ |
| **GLTI** | .825^*^ | -.821^*^ | 0.723 | 0.719 | 0.727 | 0.772 | -0.800 | 0.558 | -0.730 | 0.716 | -0.797 | 0.715 | .850^*^ | 0.803 | .917^*^ | 1 | 0.762 | -0.761 | .945^**^ | 0.783 |
| **GLUL** | .990^**^ | -0.573 | .985^**^ | .989^**^ | .988^**^ | .941^**^ | -0.435 | -0.066 | -0.342 | .988^**^ | -0.513 | .986^**^ | .947^**^ | 0.807 | .895^*^ | 0.762 | 1 | -0.455 | .839^*^ | .990^**^ |
| **argD** | -0.568 | 0.676 | -0.393 | -0.406 | -0.406 | -0.632 | .973^**^ | -0.634 | .899^*^ | -0.400 | .987^**^ | -0.390 | -0.658 | -0.787 | -0.657 | -0.761 | -0.455 | 1 | -.840^*^ | -0.492 |
| **argE-1** | .905^*^ | -0.762 | 0.806 | 0.810 | .812^*^ | .890^*^ | -.828^*^ | 0.392 | -0.731 | 0.806 | -.855^*^ | 0.801 | .928^**^ | .883^*^ | .939^**^ | .945^**^ | .839^*^ | -.840^*^ | 1 | .867^*^ |
| **argE-2** | .990^**^ | -0.534 | .994^**^ | .994^**^ | .995^**^ | .922^**^ | -0.453 | -0.064 | -0.337 | .994^**^ | -0.534 | .993^**^ | .933^**^ | 0.774 | .892^*^ | 0.783 | .990^**^ | -0.492 | .867^*^ | 1 |
| **argE-3** | .997^**^ | -0.587 | .981^**^ | .984^**^ | .984^**^ | .940^**^ | -0.512 | 0.006 | -0.403 | .983^**^ | -0.594 | .981^**^ | .953^**^ | .826^*^ | .910^*^ | .816^*^ | .992^**^ | -0.542 | .891^*^ | .995^**^ |
| **argE-4** | -0.630 | .963^**^ | -0.436 | -0.450 | -0.448 | -0.716 | .900^*^ | -0.743 | .932^**^ | -0.443 | .847^*^ | -0.432 | -0.767 | -.880^*^ | -0.805 | -.844^*^ | -0.557 | 0.800 | -0.799 | -0.524 |
| **argE-5** | -0.612 | .834^*^ | -0.446 | -0.452 | -0.452 | -0.701 | .927^**^ | -0.620 | .908^*^ | -0.445 | .843^*^ | -0.437 | -0.733 | -0.755 | -0.798 | -0.793 | -0.506 | .885^*^ | -.851^*^ | -0.536 |
| **argE-5** | -0.612 | .834^*^ | -0.446 | -0.452 | -0.452 | -0.701 | .927^**^ | -0.620 | .908^*^ | -0.445 | .843^*^ | -0.437 | -0.733 | -0.755 | -0.798 | -0.793 | -0.506 | .885^*^ | -.851^*^ | -0.536 |
| **LOX2S0-1** | .929^**^ | -0.758 | .854^*^ | .865^*^ | .858^*^ | .984^**^ | -0.619 | 0.105 | -0.584 | .861^*^ | -0.616 | .852^*^ | .975^**^ | .885^*^ | .945^**^ | 0.753 | .906^*^ | -0.597 | .867^*^ | .889^*^ |
| **LOX2S0-2** | 0.791 | -0.694 | 0.717 | 0.707 | 0.719 | 0.691 | -0.742 | 0.511 | -0.637 | 0.706 | -0.764 | 0.708 | 0.777 | 0.707 | .849^*^ | .979^**^ | 0.726 | -0.735 | .920^**^ | 0.769 |
| **LOX2S0-3** | 0.196 | -0.785 | -0.017 | -0.012 | -0.007 | 0.276 | -.868^*^ | .978^**^ | -.919^**^ | -0.019 | -0.762 | -0.026 | 0.355 | 0.531 | 0.466 | 0.655 | 0.086 | -0.759 | 0.540 | 0.087 |
| **LOX2S0-4** | -0.615 | .860^*^ | -0.448 | -0.453 | -0.453 | -0.707 | .915^*^ | -0.626 | .907^*^ | -0.447 | .821^*^ | -0.438 | -0.741 | -0.757 | -.812^*^ | -0.797 | -0.512 | .860^*^ | -.844^*^ | -0.537 |
| **LOX1-5** | .968^**^ | -0.452 | 1.000^**^ | .999^**^ | .999^**^ | .886^*^ | -0.335 | -0.179 | -0.218 | .999^**^ | -0.421 | .999^**^ | .891^*^ | 0.700 | .845^*^ | 0.710 | .983^**^ | -0.379 | 0.797 | .992^**^ |
| **LCAT3** | -0.695 | .930^**^ | -0.515 | -0.525 | -0.526 | -0.747 | .925^**^ | -0.722 | .920^**^ | -0.519 | .898^*^ | -0.510 | -0.803 | -.897^*^ | -.839^*^ | -.907^*^ | -0.618 | .852^*^ | -.869^*^ | -0.601 |
| **DOX** | -0.422 | 0.636 | -0.316 | -0.312 | -0.313 | -0.512 | 0.650 | -0.397 | 0.654 | -0.308 | 0.501 | -0.303 | -0.530 | -0.439 | -0.637 | -0.544 | -0.331 | 0.599 | -0.620 | -0.379 |
| **AOS** | .968^**^ | -0.443 | 1.000^**^ | .999^**^ | 1.000^**^ | .883^*^ | -0.339 | -0.174 | -0.217 | .999^**^ | -0.435 | 1.000^**^ | .888^*^ | 0.707 | .837^*^ | 0.713 | .984^**^ | -0.387 | 0.798 | .992^**^ |

|  | **argE-3** | **argE-4** | **argE-5** | **argE-5** | **LOX2S0-1** | **LOX2S0-2** | **LOX2S0-3** | **LOX2S0-4** | **LOX1-5** | **LCAT3** | **DOX** | **AOS** |
| --- | --- | --- | --- | --- | --- | --- | --- | --- | --- | --- | --- | --- |
| **Raffinose** | -0.240 | .882^*^ | 0.586 | 0.586 | -0.451 | -0.349 | -0.794 | 0.610 | -0.061 | 0.804 | 0.300 | -0.064 |
| **Stachyose** | -0.373 | .893^*^ | 0.657 | 0.657 | -0.572 | -0.511 | -0.773 | 0.696 | -0.238 | .822^*^ | 0.480 | -0.229 |
| **Manninotriose** | -0.395 | .971^**^ | 0.791 | 0.791 | -0.577 | -0.601 | -.905^*^ | 0.811 | -0.217 | .928^**^ | 0.531 | -0.216 |
| **D-Glucose 6-phosphate** | 0.654 | -0.652 | -0.582 | -0.582 | 0.508 | .938^**^ | 0.567 | -0.592 | 0.587 | -0.722 | -0.409 | 0.590 |
| **Citric Acid** | 0.025 | -0.736 | -0.591 | -0.591 | 0.347 | 0.109 | 0.735 | -0.606 | -0.146 | -0.639 | -0.402 | -0.150 |
| **Shikimic acid** | 0.236 | -.890^*^ | -0.805 | -0.805 | 0.379 | 0.622 | .995^**^ | -0.810 | 0.044 | -.872^*^ | -0.557 | 0.046 |
| **13(S)-HPODE** | 0.063 | -0.773 | -0.659 | -0.659 | 0.336 | 0.219 | .812^*^ | -0.662 | -0.129 | -0.704 | -0.418 | -0.127 |
| **9,10,13-TriHOME** | 0.561 | -.982^**^ | -.856^*^ | -.856^*^ | 0.641 | 0.804 | .901^*^ | -.863^*^ | 0.388 | -.988^**^ | -0.557 | 0.392 |
| **13(S)-HOTrE** | 0.175 | -0.756 | -0.726 | -0.726 | 0.223 | 0.689 | .960^**^ | -0.726 | 0.007 | -0.771 | -0.521 | 0.010 |
| **L-Tryptophan** | -0.084 | -0.708 | -0.613 | -0.613 | 0.223 | 0.133 | .823^*^ | -0.622 | -0.266 | -0.623 | -0.437 | -0.270 |
| **L-Valine** | 0.054 | -0.701 | -0.559 | -0.559 | 0.341 | 0.089 | 0.676 | -0.563 | -0.123 | -0.620 | -0.321 | -0.121 |
| **L-Saccharopine** | 0.132 | -0.803 | -0.584 | -0.584 | 0.174 | 0.594 | .957^**^ | -0.593 | -0.056 | -0.789 | -0.295 | -0.045 |
| **L-Alanine** | 0.036 | -0.598 | -0.659 | -0.659 | 0.094 | 0.601 | .887^*^ | -0.659 | -0.099 | -0.613 | -0.562 | -0.104 |
| **L-Arginine** | -0.113 | -0.660 | -0.559 | -0.559 | -0.008 | 0.421 | .954^**^ | -0.562 | -0.295 | -0.636 | -0.360 | -0.290 |
| **E2.4.1.82-1** | .990^**^ | -0.618 | -0.606 | -0.606 | .946^**^ | 0.757 | 0.170 | -0.613 | .970^**^ | -0.673 | -0.444 | .967^**^ |
| **E2.4.1.82-2** | .995^**^ | -0.530 | -0.530 | -0.530 | .904^*^ | 0.736 | 0.074 | -0.531 | .989^**^ | -0.601 | -0.366 | .990^**^ |
| **E2.4.1.67-1** | -0.537 | .842^*^ | 0.496 | 0.496 | -0.474 | -0.642 | -0.672 | 0.493 | -0.359 | .859^*^ | 0.035 | -0.386 |
| **E2.4.1.67-2** | -0.516 | 0.782 | 0.479 | 0.479 | -0.423 | -0.631 | -0.640 | 0.465 | -0.337 | .816^*^ | 0.003 | -0.368 |
| **INV** | .956^**^ | -0.675 | -0.649 | -0.649 | .961^**^ | 0.689 | 0.223 | -0.650 | .906^*^ | -0.717 | -0.438 | .907^*^ |
| **SUS-1** | 0.668 | -.950^**^ | -.878^*^ | -.878^*^ | 0.701 | .898^*^ | .838^*^ | -.883^*^ | 0.516 | -.980^**^ | -0.601 | 0.518 |
| **SUS-2** | 0.018 | -0.731 | -0.660 | -0.660 | 0.120 | 0.537 | .978^**^ | -0.664 | -0.161 | -0.717 | -0.462 | -0.158 |
| **SUS-3** | -0.050 | -0.755 | -0.666 | -0.666 | 0.156 | 0.354 | .961^**^ | -0.670 | -0.245 | -0.706 | -0.456 | -0.244 |
| **SUS-4** | .957^**^ | -0.766 | -0.691 | -0.691 | .882^*^ | .906^*^ | 0.413 | -0.698 | .891^*^ | -.827^*^ | -0.454 | .894^*^ |
| **SUS-5** | -0.001 | -0.737 | -0.651 | -0.651 | 0.296 | 0.173 | 0.809 | -0.656 | -0.187 | -0.662 | -0.446 | -0.188 |
| **SUS-6** | 0.306 | -.879^*^ | -.868^*^ | -.868^*^ | 0.586 | 0.444 | .842^*^ | -.875^*^ | 0.135 | -.830^*^ | -0.678 | 0.127 |
| **SUS-7** | -0.019 | -0.756 | -0.647 | -0.647 | 0.253 | 0.216 | .861^*^ | -0.652 | -0.212 | -0.684 | -0.427 | -0.212 |
| **SUS-8** | -0.571 | 0.777 | 0.733 | 0.733 | -0.534 | -0.728 | -0.692 | 0.704 | -0.399 | .840^*^ | 0.347 | -0.420 |
| **SUS-9** | -0.511 | 0.694 | 0.633 | 0.633 | -0.438 | -0.665 | -0.629 | 0.598 | -0.342 | 0.765 | 0.227 | -0.368 |
| **SUS-10** | -0.418 | 0.632 | 0.270 | 0.270 | -0.266 | -0.507 | -0.503 | 0.253 | -0.254 | 0.671 | -0.220 | -0.292 |
| **UGP2-1** | -0.513 | .845^*^ | .967^**^ | .967^**^ | -0.749 | -0.655 | -0.759 | .981^**^ | -0.399 | .828^*^ | .898^*^ | -0.379 |
| **UGP2-2** | -0.414 | .894^*^ | 0.779 | 0.779 | -0.456 | -0.705 | -.904^*^ | 0.767 | -0.213 | .916^*^ | 0.412 | -0.229 |
| **GPI** | .938^**^ | -0.688 | -0.685 | -0.685 | .982^**^ | 0.669 | 0.241 | -0.689 | .890^*^ | -0.721 | -0.502 | .886^*^ |
| **PFK-1** | .977^**^ | -0.417 | -0.426 | -0.426 | .843^*^ | 0.707 | -0.038 | -0.428 | .999^**^ | -0.496 | -0.300 | 1.000^**^ |
| **PFK-2** | .979^**^ | -0.675 | -0.646 | -0.646 | .965^**^ | 0.741 | 0.226 | -0.652 | .940^**^ | -0.721 | -0.456 | .939^**^ |
| **ALDO** | .984^**^ | -0.661 | -0.624 | -0.624 | .871^*^ | .885^*^ | 0.284 | -0.627 | .944^**^ | -0.737 | -0.414 | .947^**^ |
| **GADPH** | 0.723 | -0.798 | -0.753 | -0.753 | .882^*^ | 0.515 | 0.449 | -0.753 | 0.610 | -0.791 | -0.507 | 0.609 |
|  | **argE-3** | **argE-4** | **argE-5** | **argE-5** | **LOX2S0-1** | **LOX2S0-2** | **LOX2S0-3** | **LOX2S0-4** | **LOX1-5** | **LCAT3** | **DOX** | **AOS** |
| **PK-1** | 0.631 | -0.680 | -0.639 | -0.639 | 0.486 | .961^**^ | 0.639 | -0.641 | 0.543 | -0.759 | -0.436 | 0.548 |
| **PK-2** | -0.543 | .923^**^ | 0.567 | 0.567 | -0.607 | -0.617 | -0.707 | 0.595 | -0.393 | .888^*^ | 0.238 | -0.402 |
| **PK-3** | -0.551 | 0.767 | 0.626 | 0.626 | -0.485 | -0.681 | -0.657 | 0.599 | -0.375 | .824^*^ | 0.191 | -0.401 |
| **PDAH** | .997^**^ | -0.630 | -0.612 | -0.612 | .929^**^ | 0.791 | 0.196 | -0.615 | .968^**^ | -0.695 | -0.422 | .968^**^ |
| **CS** | -0.587 | .963^**^ | .834^*^ | .834^*^ | -0.758 | -0.694 | -0.785 | .860^*^ | -0.452 | .930^**^ | 0.636 | -0.443 |
| **ACO-1** | .981^**^ | -0.436 | -0.446 | -0.446 | .854^*^ | 0.717 | -0.017 | -0.448 | 1.000^**^ | -0.515 | -0.316 | 1.000^**^ |
| **ACO-2** | .984^**^ | -0.450 | -0.452 | -0.452 | .865^*^ | 0.707 | -0.012 | -0.453 | .999^**^ | -0.525 | -0.312 | .999^**^ |
| **ACO-3** | .984^**^ | -0.448 | -0.452 | -0.452 | .858^*^ | 0.719 | -0.007 | -0.453 | .999^**^ | -0.526 | -0.313 | 1.000^**^ |
| **IDH3** | .940^**^ | -0.716 | -0.701 | -0.701 | .984^**^ | 0.691 | 0.276 | -0.707 | .886^*^ | -0.747 | -0.512 | .883^*^ |
| **OGDH** | -0.512 | .900^*^ | .927^**^ | .927^**^ | -0.619 | -0.742 | -.868^*^ | .915^*^ | -0.335 | .925^**^ | 0.650 | -0.339 |
| **trpE** | 0.006 | -0.743 | -0.620 | -0.620 | 0.105 | 0.511 | .978^**^ | -0.626 | -0.179 | -0.722 | -0.397 | -0.174 |
| **trpD** | -0.403 | .932^**^ | .908^*^ | .908^*^ | -0.584 | -0.637 | -.919^**^ | .907^*^ | -0.218 | .920^**^ | 0.654 | -0.217 |
| **ilvA** | .983^**^ | -0.443 | -0.445 | -0.445 | .861^*^ | 0.706 | -0.019 | -0.447 | .999^**^ | -0.519 | -0.308 | .999^**^ |
| **ilvH** | -0.594 | .847^*^ | .843^*^ | .843^*^ | -0.616 | -0.764 | -0.762 | .821^*^ | -0.421 | .898^*^ | 0.501 | -0.435 |
| **ilvE-1** | .981^**^ | -0.432 | -0.437 | -0.437 | .852^*^ | 0.708 | -0.026 | -0.438 | .999^**^ | -0.510 | -0.303 | 1.000^**^ |
| **ilvE-2** | .953^**^ | -0.767 | -0.733 | -0.733 | .975^**^ | 0.777 | 0.355 | -0.741 | .891^*^ | -0.803 | -0.530 | .888^*^ |
| **ilvE-3** | .826^*^ | -.880^*^ | -0.755 | -0.755 | .885^*^ | 0.707 | 0.531 | -0.757 | 0.700 | -.897^*^ | -0.439 | 0.707 |
| **IMDH** | .910^*^ | -0.805 | -0.798 | -0.798 | .945^**^ | .849^*^ | 0.466 | -.812^*^ | .845^*^ | -.839^*^ | -0.637 | .837^*^ |
| **GLTI** | .816^*^ | -.844^*^ | -0.793 | -0.793 | 0.753 | .979^**^ | 0.655 | -0.797 | 0.710 | -.907^*^ | -0.544 | 0.713 |
| **GLUL** | .992^**^ | -0.557 | -0.506 | -0.506 | .906^*^ | 0.726 | 0.086 | -0.512 | .983^**^ | -0.618 | -0.331 | .984^**^ |
| **argD** | -0.542 | 0.800 | .885^*^ | .885^*^ | -0.597 | -0.735 | -0.759 | .860^*^ | -0.379 | .852^*^ | 0.599 | -0.387 |
| **argE-1** | .891^*^ | -0.799 | -.851^*^ | -.851^*^ | .867^*^ | .920^**^ | 0.540 | -.844^*^ | 0.797 | -.869^*^ | -0.620 | 0.798 |
| **argE-2** | .995^**^ | -0.524 | -0.536 | -0.536 | .889^*^ | 0.769 | 0.087 | -0.537 | .992^**^ | -0.601 | -0.379 | .992^**^ |
| **argE-3** | 1 | -0.592 | -0.566 | -0.566 | .899^*^ | 0.793 | 0.158 | -0.566 | .977^**^ | -0.665 | -0.368 | .980^**^ |
| **argE-4** | -0.592 | 1 | .831^*^ | .831^*^ | -0.708 | -0.737 | -.845^*^ | .844^*^ | -0.423 | .987^**^ | 0.529 | -0.426 |
| **argE-5** | -0.566 | .831^*^ | 1 | 1.000^**^ | -0.745 | -0.725 | -0.758 | .998^**^ | -0.441 | .847^*^ | .879^*^ | -0.428 |
| **argE-5** | -0.566 | .831^*^ | 1.000^**^ | 1 | -0.745 | -0.725 | -0.758 | .998^**^ | -0.441 | .847^*^ | .879^*^ | -0.428 |
| **LOX2S0-1** | .899^*^ | -0.708 | -0.745 | -0.745 | 1 | 0.659 | 0.289 | -0.757 | .857^*^ | -0.725 | -0.626 | .845^*^ |
| **LOX2S0-2** | 0.793 | -0.737 | -0.725 | -0.725 | 0.659 | 1 | 0.590 | -0.720 | 0.702 | -.826^*^ | -0.484 | 0.709 |
| **LOX2S0-3** | 0.158 | -.845^*^ | -0.758 | -0.758 | 0.289 | 0.590 | 1 | -0.762 | -0.034 | -.828^*^ | -0.517 | -0.031 |
| **LOX2S0-4** | -0.566 | .844^*^ | .998^**^ | .998^**^ | -0.757 | -0.720 | -0.762 | 1 | -0.444 | .853^*^ | .886^*^ | -0.429 |
| **LOX1-5** | .977^**^ | -0.423 | -0.441 | -0.441 | .857^*^ | 0.702 | -0.034 | -0.444 | 1 | -0.500 | -0.324 | .999^**^ |
| **LCAT3** | -0.665 | .987^**^ | .847^*^ | .847^*^ | -0.725 | -.826^*^ | -.828^*^ | .853^*^ | -0.500 | 1 | 0.527 | -0.506 |
| **DOX** | -0.368 | 0.529 | .879^*^ | .879^*^ | -0.626 | -0.484 | -0.517 | .886^*^ | -0.324 | 0.527 | 1 | -0.291 |
| **AOS** | .980^**^ | -0.426 | -0.428 | -0.428 | .845^*^ | 0.709 | -0.031 | -0.429 | .999^**^ | -0.506 | -0.291 | 1 |

| **Supplementary Table 7. The connection network of related flavor metabolites and genes of fresh *C.heterophylla* × *C.avellana*  of figure 6** P-value<0.05 is a significant correlation; P-value<0.01 is a highly significant correlation. | | | |
| --- | --- | --- | --- |
| **Index.1** | **Index.2** | **Correlation** | **P-value** |
| Raffinose | UGP2-1 | 0.916277466 | 0.010220769 |
| Raffinose | trpD | 0.894407442 | 0.016136015 |
| Raffinose | argE-4 | 0.8808108 | 0.020462493 |
| Stachyose | E2.4.1.67-1 | 0.853831139 | 0.03048653 |
| Stachyose | E2.4.1.67-2 | 0.852122974 | 0.031184563 |
| Stachyose | SUS-1 | -0.852960631 | 0.030841326 |
| Stachyose | SUS-2 | -0.926436204 | 0.007918398 |
| Stachyose | SUS-5 | -0.879202103 | 0.021006849 |
| Stachyose | SUS-6 | -0.834457987 | 0.038837967 |
| Stachyose | UGP2-2 | 0.918064752 | 0.009795046 |
| Stachyose | PK-2 | 0.856947668 | 0.029232245 |
| Stachyose | PDAH | -0.832049894 | 0.039942153 |
| Stachyose | CS | 0.856438307 | 0.029435539 |
| Stachyose | OGDH | 0.908816807 | 0.012092496 |
| Stachyose | trpD | 0.947828824 | 0.004011747 |
| Stachyose | ilvH | 0.849011709 | 0.032475121 |
| Stachyose | GLTI | -0.846895124 | 0.033367181 |
| Stachyose | argD | 0.892307075 | 0.016772151 |
| Stachyose | argE-4 | 0.954419937 | 0.003068966 |
| Stachyose | argE-5 | 0.83816457 | 0.037166767 |
| Stachyose | LOX2S-3 | -0.869001017 | 0.024617081 |
| Stachyose | LOX2S-4 | 0.853142768 | 0.030766932 |
| Manninotriose | E2.4.1.67-1 | 0.890730327 | 0.01725746 |
| Manninotriose | E2.4.1.67-2 | 0.889797402 | 0.017547735 |
| Manninotriose | SUS-1 | -0.888020436 | 0.018107055 |
| Manninotriose | SUS-2 | -0.932353468 | 0.006709303 |
| Manninotriose | SUS-5 | -0.915494736 | 0.010409977 |
| Manninotriose | SUS-6 | -0.866298669 | 0.025619043 |
| Manninotriose | UGP2-2 | 0.934697132 | 0.006257456 |
| Manninotriose | PK-2 | 0.892106408 | 0.016833545 |
| Manninotriose | PDAH | -0.870583136 | 0.024039303 |
| Manninotriose | CS | 0.891248682 | 0.017097181 |
| Manninotriose | ACO-2 | -0.827823054 | 0.041915267 |
| **Index.1** | **Index.2** | **Correlation** | **P-value** |
| Manninotriose | OGDH | 0.940830066 | 0.005148042 |
| Manninotriose | trpD | 0.983365474 | 0.000412760 |
| Manninotriose | ilvH | 0.887457861 | 0.018285885 |
| Manninotriose | GLTI | -0.88099783 | 0.020399649 |
| Manninotriose | argD | 0.933856697 | 0.006417718 |
| Manninotriose | argE-4 | 0.96602708 | 0.001711634 |
| Manninotriose | argE-5 | 0.870573043 | 0.024042968 |
| Manninotriose | LOX2S-3 | -0.903162346 | 0.013612248 |
| Manninotriose | LOX2S-4 | 0.889112995 | 0.017762163 |
| D-Glucose 6-phosphate | PK-1 | 0.950431081 | 0.003624719 |
| D-Glucose 6-phosphate | LCAT3 | -0.941128869 | 0.005096697 |
| Citric Acid | trpD | -0.854477341 | 0.030224411 |
| Citric Acid | argE-4 | -0.860600813 | 0.027793788 |
| Shikimic acid | E2.4.1.67-1 | -0.945941874 | 0.004304435 |
| Shikimic acid | E2.4.1.67-2 | -0.945980005 | 0.004298420 |
| Shikimic acid | SUS-1 | 0.94752429 | 0.004058299 |
| Shikimic acid | SUS-2 | 0.971188712 | 0.001233177 |
| Shikimic acid | SUS-5 | 0.962365671 | 0.002097863 |
| Shikimic acid | SUS-6 | 0.899015741 | 0.014781821 |
| Shikimic acid | SUS-8 | -0.861916775 | 0.027284051 |
| Shikimic acid | SUS-9 | -0.862771337 | 0.026955434 |
| Shikimic acid | UGP2-2 | -0.969609772 | 0.001371315 |
| Shikimic acid | PK-2 | -0.946750845 | 0.004177715 |
| Shikimic acid | PDAH | 0.933006705 | 0.006581816 |
| Shikimic acid | CS | -0.947677358 | 0.004034868 |
| Shikimic acid | ACO-2 | 0.89222929 | 0.016795936 |
| Shikimic acid | OGDH | -0.984915222 | 0.000339610 |
| Shikimic acid | trpD | -0.96938178 | 0.001391861 |
| Shikimic acid | ilvH | -0.94694936 | 0.004146903 |
| Shikimic acid | GLTI | 0.943995612 | 0.004616909 |
| Shikimic acid | argD | -0.975440886 | 0.000897319 |
| Shikimic acid | argE-4 | -0.914144339 | 0.010740362 |
| Shikimic acid | argE-5 | -0.942494384 | 0.004865261 |
| Shikimic acid | LOX2S-3 | 0.957510442 | 0.002669689 |
| Shikimic acid | LOX2S-4 | -0.947793193 | 0.004017180 |
| Phosphoenolpyruvate | E2.4.1.82-2 | 0.828964006 | 0.041378282 |
| Phosphoenolpyruvate | E2.4.1.67-1 | -0.907473697 | 0.012445611 |
| Phosphoenolpyruvate | E2.4.1.67-2 | -0.910123216 | 0.011753750 |
| Phosphoenolpyruvate | SUS-1 | 0.913347129 | 0.010937754 |
| Phosphoenolpyruvate | SUS-2 | 0.861250147 | 0.027541710 |
| **Index.1** | **Index.2** | **Correlation** | **P-value** |
| Phosphoenolpyruvate | SUS-5 | 0.901068291 | 0.014197078 |
| Phosphoenolpyruvate | SUS-8 | -0.944797155 | 0.004486920 |
| Phosphoenolpyruvate | SUS-9 | -0.919312088 | 0.009503148 |
| Phosphoenolpyruvate | UGP2-2 | -0.901751691 | 0.014004913 |
| Phosphoenolpyruvate | ALDO | 0.887743754 | 0.018194901 |
| Phosphoenolpyruvate | GADPH | 0.816434424 | 0.047451739 |
| Phosphoenolpyruvate | PK-1 | 0.845282835 | 0.034054338 |
| Phosphoenolpyruvate | PK-2 | -0.903883506 | 0.013413590 |
| Phosphoenolpyruvate | PDAH | 0.908072372 | 0.012287607 |
| Phosphoenolpyruvate | CS | -0.905703829 | 0.012918422 |
| Phosphoenolpyruvate | ACO-2 | 0.881161437 | 0.020344751 |
| Phosphoenolpyruvate | OGDH | -0.921440304 | 0.009015018 |
| Phosphoenolpyruvate | ilvH | -0.915884274 | 0.010315604 |
| Phosphoenolpyruvate | GLTI | 0.917043448 | 0.010037239 |
| Phosphoenolpyruvate | argD | -0.915686708 | 0.010363417 |
| Phosphoenolpyruvate | argE-1 | 0.930084025 | 0.007161482 |
| Phosphoenolpyruvate | argE-5 | -0.926186158 | 0.007971638 |
| Phosphoenolpyruvate | LOX2S-2 | 0.96939647 | 0.001390533 |
| Phosphoenolpyruvate | LOX2S-3 | 0.911190676 | 0.011480420 |
| Phosphoenolpyruvate | LOX2S-4 | -0.910521244 | 0.011651468 |
| Phosphoenolpyruvate | LCAT3 | -0.873723555 | 0.022911825 |
| 13(S)-HPODE | E2.4.1.67-1 | -0.845412678 | 0.033998755 |
| 13(S)-HPODE | E2.4.1.67-2 | -0.845492595 | 0.033964566 |
| 13(S)-HPODE | SUS-1 | 0.836442846 | 0.037938760 |
| 13(S)-HPODE | SUS-2 | 0.850556231 | 0.031831364 |
| 13(S)-HPODE | SUS-5 | 0.871104377 | 0.023850381 |
| 13(S)-HPODE | UGP2-1 | -0.823389558 | 0.044032522 |
| 13(S)-HPODE | UGP2-2 | -0.904608089 | 0.013215410 |
| 13(S)-HPODE | PK-2 | -0.842787457 | 0.035130860 |
| 13(S)-HPODE | PDAH | 0.82130521 | 0.045044716 |
| 13(S)-HPODE | CS | -0.839122123 | 0.036740641 |
| 13(S)-HPODE | OGDH | -0.907457972 | 0.012449774 |
| 13(S)-HPODE | trpD | -0.973417566 | 0.001050547 |
| 13(S)-HPODE | ilvH | -0.841685667 | 0.035611192 |
| 13(S)-HPODE | GLTI | 0.826849456 | 0.042376044 |
| 13(S)-HPODE | argD | -0.915950144 | 0.010299688 |
| 13(S)-HPODE | argE-4 | -0.955478161 | 0.002929166 |
| **Index.1** | **Index.2** | **Correlation** | **P-value** |
| 13(S)-HPODE | LOX2S-3 | 0.853051906 | 0.030804034 |
| 13(S)-HPODE | LOX2S-4 | -0.837680693 | 0.037382978 |
| 9,10,13-TriHOME | E2.4.1.67-1 | -0.985097526 | 0.000331471 |
| 9,10,13-TriHOME | E2.4.1.67-2 | -0.983817346 | 0.000390698 |
| 9,10,13-TriHOME | SUS-1 | 0.98104246 | 0.000535676 |
| 9,10,13-TriHOME | SUS-2 | 0.871812732 | 0.023594778 |
| 9,10,13-TriHOME | SUS-4 | 0.939048048 | 0.005459488 |
| 9,10,13-TriHOME | SUS-5 | 0.979796386 | 0.000608156 |
| 9,10,13-TriHOME | SUS-6 | 0.821081328 | 0.045154074 |
| 9,10,13-TriHOME | SUS-7 | 0.849531664 | 0.032257724 |
| 9,10,13-TriHOME | SUS-8 | -0.916856809 | 0.010081810 |
| 9,10,13-TriHOME | SUS-9 | -0.911933793 | 0.011291980 |
| 9,10,13-TriHOME | UGP2-2 | -0.90600643 | 0.012836980 |
| 9,10,13-TriHOME | ALDO | 0.920964263 | 0.009123117 |
| 9,10,13-TriHOME | PK-2 | -0.985084638 | 0.000332043 |
| 9,10,13-TriHOME | PDAH | 0.977031316 | 0.000785282 |
| 9,10,13-TriHOME | CS | -0.981359723 | 0.000517952 |
| 9,10,13-TriHOME | ACO-2 | 0.958629017 | 0.002531933 |
| 9,10,13-TriHOME | OGDH | -0.927652025 | 0.007662001 |
| 9,10,13-TriHOME | trpD | -0.882549408 | 0.019881868 |
| 9,10,13-TriHOME | ilvH | -0.977934901 | 0.000724931 |
| 9,10,13-TriHOME | ilvE-3 | 0.900160507 | 0.014454290 |
| 9,10,13-TriHOME | IMDH | 0.850155368 | 0.031997859 |
| 9,10,13-TriHOME | GLTI | 0.980976928 | 0.000539374 |
| 9,10,13-TriHOME | argD | -0.920087321 | 0.009323892 |
| 9,10,13-TriHOME | argE-5 | -0.96214718 | 0.002122136 |
| 9,10,13-TriHOME | LOX2S-2 | 0.814766443 | 0.048289388 |
| 9,10,13-TriHOME | LOX2S-3 | 0.981952388 | 0.000485635 |
| 9,10,13-TriHOME | LOX2S-4 | -0.97758035 | 0.000748327 |
| 13(S)-HOTrE | E2.4.1.67-1 | -0.8784842 | 0.021251978 |
| 13(S)-HOTrE | E2.4.1.67-2 | -0.880217667 | 0.020662404 |
| 13(S)-HOTrE | SUS-1 | 0.888261243 | 0.018030765 |
| 13(S)-HOTrE | SUS-2 | 0.972271197 | 0.001142670 |
| 13(S)-HOTrE | SUS-5 | 0.89620247 | 0.015601737 |
| 13(S)-HOTrE | SUS-6 | 0.873705925 | 0.022918083 |
| 13(S)-HOTrE | SUS-8 | -0.833603232 | 0.039228240 |
| 13(S)-HOTrE | SUS-9 | -0.817280206 | 0.047029595 |
| **Index.1** | **Index.2** | **Correlation** | **P-value** |
| 13(S)-HOTrE | UGP2-2 | -0.950402432 | 0.003628875 |
| 13(S)-HOTrE | PK-2 | -0.879192747 | 0.021010035 |
| 13(S)-HOTrE | PDAH | 0.870862287 | 0.023938038 |
| 13(S)-HOTrE | CS | -0.884510898 | 0.019236418 |
| 13(S)-HOTrE | ACO-2 | 0.818838933 | 0.046256206 |
| 13(S)-HOTrE | OGDH | -0.961527037 | 0.002191780 |
| 13(S)-HOTrE | trpD | -0.895034319 | 0.015948446 |
| 13(S)-HOTrE | ilvH | -0.888321939 | 0.018011560 |
| 13(S)-HOTrE | GLTI | 0.887421778 | 0.018297384 |
| 13(S)-HOTrE | argD | -0.946041309 | 0.004288759 |
| 13(S)-HOTrE | argE-1 | 0.822196017 | 0.044610814 |
| 13(S)-HOTrE | argE-4 | -0.843137667 | 0.034978826 |
| 13(S)-HOTrE | argE-5 | -0.905996493 | 0.012839650 |
| 13(S)-HOTrE | LOX2S-2 | 0.822285379 | 0.044567395 |
| 13(S)-HOTrE | LOX2S-3 | 0.897108388 | 0.015335385 |
| 13(S)-HOTrE | LOX2S-4 | -0.889067204 | 0.017776554 |
| L-Tryptophan | SUS-2 | 0.824693689 | 0.043404671 |
| L-Tryptophan | trpD | -0.917166125 | 0.010007996 |
| L-Tryptophan | argE-4 | -0.972072977 | 0.001158987 |
| L-Valine | UGP2-1 | -0.897604044 | 0.015190591 |
| L-Valine | trpD | -0.910598122 | 0.011631763 |
| L-Valine | argD | -0.823217172 | 0.044115829 |
| L-Valine | argE-4 | -0.896412358 | 0.015539831 |
| L-Saccharopine | E2.4.1.67-1 | -0.832788815 | 0.039601793 |
| L-Saccharopine | E2.4.1.67-2 | -0.830077801 | 0.040857202 |
| L-Saccharopine | SUS-1 | 0.819078715 | 0.046137763 |
| L-Saccharopine | SUS-5 | 0.831824499 | 0.040046245 |
| L-Saccharopine | SUS-7 | 0.922338217 | 0.008812826 |
| L-Saccharopine | SUS-10 | -0.888677446 | 0.017899272 |
| L-Saccharopine | UGP2-2 | -0.894164527 | 0.016208982 |
| L-Saccharopine | PK-2 | -0.830145513 | 0.040825623 |
| L-Saccharopine | CS | -0.819035593 | 0.046159053 |
| L-Saccharopine | OGDH | -0.825286627 | 0.043120602 |
| L-Saccharopine | trpD | -0.820542772 | 0.045417644 |
| L-Saccharopine | ilvH | -0.814306769 | 0.048521429 |
| L-Saccharopine | GLTI | 0.816673073 | 0.047332448 |
| L-Saccharopine | argE-4 | -0.81914268 | 0.046106191 |
| **Index.1** | **Index.2** | **Correlation** | **P-value** |
| L-Saccharopine | LOX2S-3 | 0.829192191 | 0.041271276 |
| L-Alanine | SUS-2 | 0.900336993 | 0.014404110 |
| L-Alanine | SUS-6 | 0.819404847 | 0.045976894 |
| L-Arginine | UGP2-2 | -0.878908608 | 0.021106899 |
| L-Arginine | trpD | -0.860220109 | 0.027942088 |
| L-Arginine | argE-4 | -0.953978228 | 0.003128268 |
